# Supplementary material for: Maternal and child health intervention to promote behaviour change: a population-level cluster-randomised controlled trial in Honduras
Source: BMJ Open. 2024 Jun 10;14(6):e060784. doi: 10.1136/bmjopen-2022-060784 (PMC11168147; doi:10.1136/bmjopen-2022-060784)
Supplement: Supplementary data [file bmjopen-2022-060784supp001.pdf]

ONLINE SUPPLEMENTARY APPENDIX

Maternal and child health intervention to promote behavior change: a population-level cluster-randomized controlled trial in Honduras

William Oles, BS

Marcus Alexander, PhD

Rennie Negron, MPH

Jennifer Nelson, MPH

Emma Iriarte, MD

E. M. Airoldi, PhD

Nicholas A. Christakis, MD, PhD, MPH

Laura Forastiere, PhD

Table of Contents

Pilot work **3**

Field operations **3**

Community engagement **3**

Missing data and attrition **3**

Blocking of villages and randomisation **3**

Assignment of households to treatment/control in each village **4**

Intervention sessions **5**

Statistical methods **5**

Additional analysis **7**

Tables **9**

Table S1. Pooled direct, indirect, and total effect estimates of intervention on maternal, child, and neonatal outcomes 9

Table S2. Pooled total effect estimates of intervention on maternal, child, and neonatal outcomes, mothers responses for practice outcomes only 20

Table S3. Pooled total effect estimates of intervention on maternal, child, and neonatal outcomes, estimated on sub-sample of respondents who did not move between villages during study 22

Table S4. Pooled total effect estimates of intervention on maternal, child, and neonatal outcomes, with adjustment for baseline demographic characteristics 29

Table S5. Pooled total effect estimates of intervention on maternal, child, and neonatal outcomes, with adjustment for baseline outcome 36

Table S6. Pooled total effect estimates of intervention on maternal, child, and neonatal outcomes, with adjustment for baseline demographic characteristics and baseline outcome 43

Table S7. Pooled total effect estimates of intervention on maternal, child, and neonatal outcomes, with adjustment for baseline demographic characteristics and baseline outcome, estimated on sub-sample of respondents who did not move between villages during study 50

Table S8. Pooled total effect estimates of intervention on maternal, child, and neonatal outcomes, intervention module receipt defined as treatment, with adjustment for baseline demographic characteristics 57

Table S9. Absolute changes in primary and secondary outcomes from baseline to endline survey 65

Table S10. Analysis of differences in attrition between baseline and endline surveys 76

References **77**

Pilot work

Prior to the rollout of our community census and baseline survey efforts, we conducted 3 separate pilot surveys. The first pilot survey, conducted in the summer of 2014, was given to 1018 individuals in 2 towns in the Lempira Department, Honduras, of whom we were able to collect network, demographic, and social normative measures from 831. The second pilot survey was conducted in February 2015, and covered 4 small villages in the Copan region, and included 165 individuals with questions on networks, demographics, as well as reproductive, maternal, child and neonatal health knowledge, attitudes, norms, and behaviors. Our final pilot survey was conducted in May 2015, for which we enrolled 577 individuals. After each pilot we carefully analyzed the data for anomalies, including large number of missing responses, no variation in item responses, or responses that seemed irregular in ways suggesting response bias or misunderstanding of questions. The survey was then revised accordingly.

Our final survey instrument included both internally-derived scales revised using aforementioned pilot work as well as additional scales with well-described external validity and reliability. Examples of such scales include Self-Rated Health (SF-1), Patient Health Questionnaire-2 (PHQ-2), Alcohol Use Disorders Identification Test (AUDIT-C), Perceived Neighborhood Safety, and the Latin American and Caribbean Household Food Security Measurement Scale (ELCSA), among others.[1-4]

Field operations

We recruited and trained over 100 local surveyors to perform preliminary data collection and infrastructure development, complete participant recruitment and census enumeration, and conduct the survey interviews. We also established 4 field offices in geographically strategic locations to minimize travel time to and from the study villages. All offices were fully equipped with data collection tablets, netbook computers, printers, high speed internet, and a local server to insure secure download, synchronization and transfer of research data. All surveyors were extensively trained in the use of our survey instruments and software by US-based project managers and supervised by Honduran project coordinators with whom we had daily contact in relation to data collection and other implementation activities.

Community engagement

We developed valuable relationships with local government and health officials, participating in staff meetings for health center personnel and community health workers. We presented project plans and obtained approval of our field operations and data collection procedures from the Honduras Ministry of Health (MOH). The MOH reviewed our study protocols, consents and survey instruments and provided feedback. Our field teams have also met with local community leaders and indigenous council members to present study objectives in all study villages prior to beginning recruitment and data collection.

Missing data and attrition

Of the 32800 estimated eligible individuals in the study villages, 93% agreed to participate in the study, so bias due to failure to recruit participants is likely to be very low. At the follow-up and final stages, we made our best efforts to track study participants enrolled at baseline who discontinued for any reason, and we attempted to continue surveying participants who moved from one study village to another. Follow-up data and attrition are reported for each treatment arm in our comprehensive trial profile shown in supplemental figure S1. Of the 5633 households initially randomized in the trial that were a part of the treatment arms included in the current analysis, 4861 households (86%) had at least one respondent who completed the endline survey. A chi-square test of independence showed that there was no significant association between household treatment status and attrition, X^2 (1, N = 5633) = 0.80, p = .37. Further analysis of differences in attrition is reported in Table S10.

Blocking of villages and randomisation

Villages were assigned to treatment arms in an 8x2 factorial design—8 dosages of households treated per village (0, 0.05, 0.1, 0.2, 0.3, 0.5, 0.75, 1), and 2 targeting strategies (random target, friend nomination target). We ran a re-

randomization procedure that generated 10000 assignments of villages to treatment arms. This procedure generates assignments while controlling balance on the following variables: number of respondents in the village, average number of respondents in the household, latitude, longitude, elevation, time to health center, and time to maternity clinic.

This procedure prioritized matching the means and standard deviations of the 16 treatment arms, but also weights the third moments and cross-moments (to get at covariances). The procedure also assigns a score that summarizes balance. We checked how similar these assignments were. Of the 100 assignments with a better balance summary score, no two contained a pair of treatment arms that overlap in more than 8 elements. We also measured the average maximum overlap, defined as the maximum number of items that any pair of treatment arms have in common. On average, over the 100 assignments with better balance summary score, the maximum overlap was 3.985, indicating that the top 100 assignments of villages to treatment arms were are all quite different.

Focusing on the 100 assignments with better balance summary score, as candidate re-randomizations, we then ran a battery of statistical tests check for statistically significant imbalances. We focused on the following variables and tests quoted in parenthesis: number of respondents in the village (t test), average number of respondents in the household (t test), latitude (t test), longitude (t test), elevation (t test), time to health center (t test), time to maternity clinic (t test), number of households (t test), empirical distribution of household sizes (Kolmogorov-Smirnov test), village can be accessed in when raining (proportions), proportion of indigenous population (t test), time in minutes to main road (t test), village is a coffee producer (proportions), average number of women of reproductive age (t test), average age (t test), and percent male (t test).

For each of the 100 assignments we produced a table with these tests for each of 147 contrasts: 120 contrasts reported the p-values for imbalance in pairs of treatment arms for each of the covariates; 26 contrasts reported the p-values for marginal imbalance in pairs of levels of treatment; 1 contrast reported the p-values for marginal imbalance in the two nomination schemes. We selected assignment number 73, which had only one imbalance at significance 5%. The imbalance was for average age between treatment arms 3 (random target, 10% treated households) and 14 (friend nomination target, 50% treated households), which is an unimportant contrast for the purpose of conducting the analyses proposed in this study.

Assignment of households to treatment/control in each village

Dosage is to be understood as proportion of households treated per village, which was applied to the number of households in each village and the rounded to the nearest whole integer number. We built a social network where a tie meant that there was a nomination in any of the following name generators: personal private matters; spent free time; closest friend. For each village, we generated 10000 assignments of households to treatment in villages in the friend nomination arm. For each of these assignments we then computed balance for the following covariates: number of census respondents, number of women of reproductive age, number of children under the age of 12 who live in this household, existence of handwashing location observed by enumerator, household electricity, separate room in the house that is used as the kitchen, self-reported health status, and network degree centrality of the household. We also considered the following individual variables averaged at the household level: age, sex, and baseline self-reported health status of the individuals in the households.

Here, for each of the 144 villages assigned to a treatment arm in which the percentage of household treated differs from 0% and 100%, we only considered the contrast: treatment vs. control. We then selected the set of assignments of household to treatment and control in each of the 144 villages that minimized the number of tests for imbalance failed at 5%. These balanced sets of assignments constituted the final assignments of households to treatment in villages in the random target arm, but only the initial assignment of households to treatment in villages in the friend nomination arm. For villages in the friend nomination arm, we picked each of the households initially assigned to treatment, in turn, then picked a person in the household, then picked a tie at random among this person’s ties. Whenever we ran into a duplicate, we restarted the choice. This procedure generated a final set of assignments of households to treatment in villages in the friend nomination arm. For the purposes of analyses of direct effects of the intervention, households in villages in the friend nomination arm were excluded since they were targeted nonrandomly to receive the intervention.

Intervention sessions

Our community-based behavioral intervention took course over 22 months (November 2016–August 2018). It was comprised of a series of household-level counseling sessions targeting maternal, child, and neonatal practices using 15 distinct educational modules delivered over a maximum of 22 sessions. Each session was designed to last two hours and was centered around the timed and targeted counseling (ttC) method, which is a bottom-up counseling strategy in which family units are introduced to information about health practices and behaviors through storytelling. Throughout each story, families encounter "problem situations" and "positive stories," after which they reflect on the presentation and negotiate agreements about practices that are appropriate or necessary to improve health outcomes. Sessions also incorporated other forms of media including educational posters, songs, and physical illustrations. In each sessions, members of the family also complete standardized questionnaires for data collection which range from providing information about their home to specific questions related to knowledge, attitudes, and practices of targeted health behaviors.

The 15 educational modules along with the percentage of targeted households randomized to treatment in random-target villages which receiving the module are listed below. Table S8 contains additional analysis which incorporates specific module receipt into our estimation of treatment effects and also describes which modules were paired with primary and secondary outcomes.

**Module**	**Description of module**	**% targeted households receiving the module (at least one session)**
m1	Pregnancy care and prenatal care in first 12 weeks	77.1
m2	Preparing a birth plan	76.3
m3	Facility-based birth	72.9
m4	Importance of family planning	75.2
m5	Care of woman during childbirth and postpartum period and newborn care	74.0
m6	Care of woman during childbirth and postpartum period and newborn care	72.2
m7	Newborn care and danger signs	72.9
m8	Infant care: caring for children between 1–6 months	78.1
m9	Danger signs and seeking medical attention for acute respiratory infections and diarrheal illness	77.5
m10	Reproductive life plan	80.7
m11	Importance of folic acid for mom and newborn	80.1
m12	Importance of preventing teen pregnancy (<18 years old)	78.7
m13	Self-esteem, values, life goals	78.5
m14	Prevention of inter-partner violence; non-violent communication	79.6
m15	Zika prevention	77.3

Statistical methods

The opportunity to answer certain questions on our survey was determined by eligibility encoded into the survey design. For example, only mothers and fathers that had a child since the end of the intervention (September 1, 2018) were eligible to have a response included for the question about whether their child was exclusively breastfed for the first 6 months. For each outcome, we restricted the analysis to the respondents eligible to answer the related question. The respondent denominator for all outcomes analyzed can be found in Table S1. Each outcome variable was coded as binary: 0 if the response was the undesired outcome related to the intervention, and 1 if the response was the desired outcome related to the intervention. Responses of 'Refusal' for any survey question were excluded for the purposes of our analysis. Responses of 'Don't know' were coded as an undesired for outcomes related to knowledge but were otherwise excluded for outcomes about practices or attitudes. The unit of analysis was the individual respondent.

Oles W, *et al. BMJ Open* 2024; 14:e060784. doi: 10.1136/bmjopen-2022-060784

In our randomized experiment, first villages were either randomly assigned to different dosages, or proportion of treated households, or to no intervention, and then household were randomly assigned to the intervention according to the dosage assigned to their village. Here we only consider the villages assigned to random targeting. Because of this, three groups of respondents exist that can be compared: pure controls (those not targeted living in villages where no one is targeted); within-cluster controls (those not targeted living in villages where a nonzero dosage of people is targeted); and treated (those targeted). Due to the presence of interference among households, in order to estimate the effect of receiving the intervention, we needed to correct for the indirect effect affecting the untreated households in a village assigned to random targeting. We make the assumption that interference could be present within villages but not between them (also known as the partial interference assumption), and that a respondent's outcome depends on the proportion of treated household in the village in addition to its own treatment (stratified interference).[5,6] Under this assumption, we denote by Yic(Tic, Pc) the potential outcome of individual i in village c under individual treatment Tic and a proportion of treated households in the village c equal to Pc. We define the following causal effects:

$$TE(p): E[Y(1,p)-Y(0,0)]$$

$$DE(p): E[Y(1,p)-Y(0,p)]$$

$$SE(p): E[Y(0,p)-Y(0,0)]$$

TE(p) is the total effect of receiving the treatment while a proportion of p households is also treated in the village compare to the control condition without any intervention in the village, DE(p) is the average direct effect of receiving the treatment versus not in a village where a proportion of p households are treated, and SE(p) is the indirect (or spillover) effect of having a proportion of p households treated in the village for the untreated individuals. We have that for each p, the total effect TE(p) is the sum of the spillover effect on the untreated SE(p) and the direct effect DE(p). Here we focus on the total and direct effects, while accounting for spillover effects. To this end, we estimate the pooled total effect TE and the pooled direct effect DE averaging across dosages p. We estimated pooled effects by combining observations from all dosages in order to measure an average effect for the entire study population. The pooled total effect can be seen as the effect on the treated due to the intervention, while the pooled direct effect is the effect of directly receiving the treatment controlling for spillover effects.

To estimate these pooled causal effects in presence of interference, we used the following regression model:

$$\text{probit}(Y_{ic}) = \beta_0 + \beta_{total}T_{ic} + \beta_{indirect}S_{ic}$$

where Y_{ic} is the observed outcome of individual i in village c ,β_0 is the population average outcome, β_{total} is the pooled total effect, T_{ic} is the treated status of individual i in village c, $\beta_{indirect}$ is the pooled indirect effect, and S_{ic} is the within-village control status of individual i in village c.[5] Given the estimated coefficients we predicted the pooled potential outcomes for the treated individual, for the within-village control individuals, and for the pure controls. Total and spillover effects were estimated subtracting the corresponding potential outcomes.

Since the pooled total effect is the sum of the pooled indirect effect and the pooled direct effect, the pooled direct effect was computed by subtracting the estimated effects:

$$DE=TE-SE$$

In addition, this estimation of pooled effects required the addition of saturation weights, since targeted individuals are given disproportionately high weight in high-dosage villages and disproportionately low weight in low-dosage villages when observations are pooled. Saturation weights were calculated on the village-level based on the empirical proportion of respondents targeted. The indirect and total effect estimates used robust standard errors clustered on village, and standard errors for the direct effect estimates were calculated using the delta method.

We performed additional sensitivity analysis to test the robustness of our primary findings. Results for our additional analyses are reported in the supplemental tables below. Table S1 reports direct and total effect estimates for all primary and secondary outcomes, and also details the percentage of respondents who reported the desired outcome at baseline. Table S2 reports the direct and total effect estimates for a sub-sample of respondents that was limited to mother of children born after the intervention period, and effects are reported only for the primary outcomes. Table S3 reports the direct and total effect estimates for all primary and secondary outcomes for a sub-sample of respondents that was limited to those who did not move between villages, and therefore treatment arms, during the

study period. Table S4 reports the direct and total effect estimates for all primary and secondary outcomes, adjusted for baseline demographic characteristics (including age, gender, marital status, indigenous status, and household wealth index). Table S5 reports the direct and total effect estimates for all primary and secondary outcomes, adjusted for outcomes at baseline. Table S6 reports the direct and total effect estimates, adjusted for baseline demographic characteristics and baseline outcomes. Table S7 reports the direct and total effect estimates for a sub-sample of respondents that was limited to those who did not move between villages, adjusted for baseline demographic characteristics and baseline outcomes. Table S8 reports the direct and total effect estimates using relevant intervention module receipt as indicators of treatment, adjusted for baseline demographic characteristics.

Additional analysis

When analysis of practices was limited to the responses of mothers, the probability of having the newborn's health checked by a professional in a health facility within three days and of not using a fajero on the umbilical cord were even greater among the targeted, and, additionally, a significant effect on the likelihood that mothers reported that the father was involved with bathing their child emerged (Table S2, appendix). We performed additional sensitivity analyses by excluding respondents that moved between treatment arms during the study, adjusting for the respondent's baseline responses for each outcome, and adjusting for demographic factors including gender, age, education, marital status, indigenous status, and household wealth index (Table S7, appendix). When all of the aforementioned covariates were included, we noted that there were no significant total effects for seeking a professional check-up for a newborn within three days of birth or for immediate breastfeeding, while the total effect estimate for not using a fajero increased. In these analyses, additional significant total effects also emerged for mothers seeking medical care after experiencing a postnatal danger sign and parents providing their children with proper treatment (defined as providing zinc, antibiotics, additional food, or oral rehydration therapy) after their child experienced past-month diarrheal illness. The variation in effects with individual covariate adjustment suggests that the impact of the intervention is heterogenous and that, while we are able to report pooled effects across the study population, it is expected that some individuals will be more likely to respond to the interventions than others.

Figures

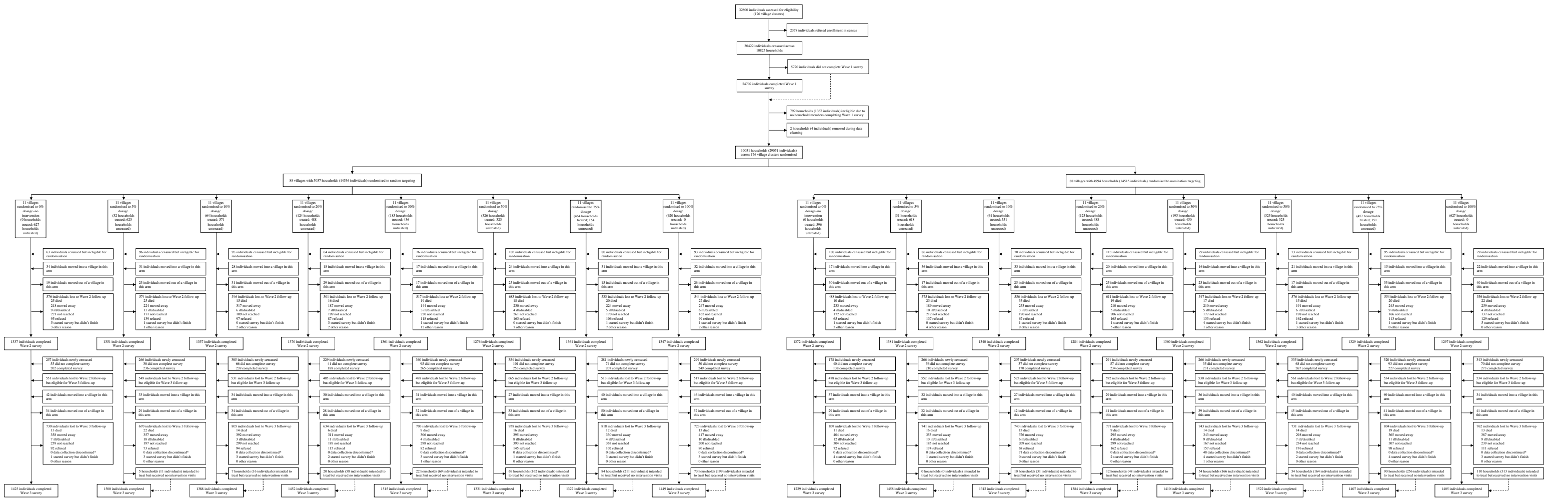

Figure S1. Trial profile for parent study

Tables

Table S1. Pooled direct, indirect, and total effect estimates of intervention on maternal, child, and neonatal outcomes

	Outcome type	Respondent denominator	Pooled direct effect estimate		Pooled indirect effect estimate		Pooled total effect estimate		N	Outcome at baseline survey (%)
			Effect size (95% CI)	p value	Effect size (95% CI)	p value	Effect size (95% CI)	p value		
**Prenatal care**										
Currently taking folic acid tablets (daily in past 7 days)	Practice	All women 15+ with a complete survey	-0·0049 (-0·034 to 0·024)	0·74	0·0053 (-0·036 to 0·047)	0·80	0·0004 (-0·043 to 0·044)	0·98	6311	32
Made a birth plan in preparation for birth	Practice	All respondents with a complete survey and a child born since 2018-09-01 (after the end of the intervention)	0·020 (-0·071 to 0·11)	0·67	0·054 (-0·020 to 0·13)	0·15	0·074 (-0·015 to 0·16)	0·10	619	61
Saved money in preparation for birth	Practice	All respondents with a complete survey and a child born since 2018-09-01 (after the end of the intervention)	0·046 (-0·061 to 0·15)	0·40	-0·065 (-0·15 to 0·023)	0·14	-0·020 (-0·14 to 0·098)	0·74	620	63
Sold animal in preparation for birth	Practice	All respondents with a complete survey and a child born since 2018-09-01 (after the end of the intervention)	-0·011 (-0·079 to 0·058)	0·76	-0·0044 (-0·055 to 0·046)	0·86	-0·015 (-0·080 to 0·049)	0·65	619	NA
Sought prenatal care within 12 weeks	Practice	All respondents with a complete survey and a child born since 2018-09-01 (after the end of the intervention)	-0·042 (-0·17 to 0·090)	0·54	-0·011 (-0·12 to 0·095)	0·84	-0·053 (-0·19 to 0·089)	0·46	597	73
Identifies that women should take folic acid before pregnancy	Knowledge and attitudes	All respondents with a complete survey	0·033 (0·0010 to 0·064)	0·044	0·010 (-0·024 to 0·044)	0·55	0·043 (0·0008 to 0·085)	0·046	10262	73
Identifies that women should seek prenatal care first 12 weeks of pregnancy	Knowledge and attitudes	All respondents with a complete survey	0·019 (-0·0061 to 0·044)	0·14	0·0038 (-0·018 to 0·026)	0·73	0·023 (-0·0046 to 0·050)	0·10	10261	87

Oles W, *et al. BMJ Open* 2024; 14:e060784. doi: 10.1136/bmjopen-2022-060784

Identifies accompanying woman to prenatal care visits as method of support during pregnancy	Knowledge and attitudes	All respondents with a complete survey	0·0051 (-0·025 to 0·036)	0·74	-0·011 (-0·060 to 0·038)	0·66	-0·0058 (-0·059 to 0·048)	0·83	10257	NA
Identifies ensuring that woman eats well as method of support during pregnancy	Knowledge and attitudes	All respondents with a complete survey	-0·0072 (-0·040 to 0·025)	0·66	-0·012 (-0·057 to 0·034)	0·61	-0·019 (-0·071 to 0·032)	0·47	10257	NA
Identifies ensuring that woman rests 1 hour per day well as method of support during pregnancy	Knowledge and attitudes	All respondents with a complete survey	0·0046 (-0·018 to 0·028)	0·69	-0·0012 (-0·034 to 0·031)	0·94	0·0034 (-0·028 to 0·035)	0·83	10257	NA
Identifies avoiding violence as method of support during pregnancy	Knowledge and attitudes	All respondents with a complete survey	-0·014 (-0·032 to 0·0037)	0·12	-0·018 (-0·036 to 0·0003)	0·054	-0·032 (-0·053 to -0·011)	0·0023	10257	NA
Identifies encouraging women to take vitamins as method of support during pregnancy	Knowledge and attitudes	All respondents with a complete survey	-0·0046 (-0·027 to 0·018)	0·68	-0·019 (-0·053 to 0·016)	0·29	-0·023 (-0·060 to 0·013)	0·21	10257	NA
Identifies helping woman with house work/child care as method of support during pregnancy	Knowledge and attitudes	All respondents with a complete survey	0·019 (-0·012 to 0·050)	0·24	0·053 (-0·0039 to 0·11)	0·068	0·072 (0·010 to 0·13)	0·022	10257	NA
Identifies saving animals to sell as method of preparing for birth expenses	Knowledge and attitudes	All respondents with a complete survey	0·017 (-0·0027 to 0·036)	0·093	0·016 (-0·0098 to 0·043)	0·22	0·033 (0·0025 to 0·063)	0·034	10261	NA
Identifies knowing cost of trip to maternal clinic as method of preparing for birth expenses	Knowledge and attitudes	All respondents with a complete survey	-0·0049 (-0·023 to 0·014)	0·61	-0·0019 (-0·031 to 0·027)	0·90	-0·0068 (-0·038 to 0·024)	0·67	10261	NA
Identifies having a savings plan as method of preparing for birth expenses	Knowledge and attitudes	All respondents with a complete survey	0·030 (0·013 to 0·047)	0·0004	0·0013 (-0·025 to 0·027)	0·92	0·031 (0·0022 to 0·060)	0·035	10261	NA
Correctly answered prenatal care knowledge riddle	Intervention knowledge	All respondents with a complete survey	0·044 (0·015 to 0·073)	0·0030	0·032 (0 to 0·065)	0·050	0·076 (0·040 to 0·11)	<0·0001	10261	NA
**Facility based birth**										
Gave birth in health facility	Practice	All respondents with a complete survey and a child born since 2018-09-01 (after the end of the intervention)	0·018 (-0·075 to 0·11)	0·70	-0·031 (-0·17 to 0·11)	0·67	-0·013 (-0·15 to 0·13)	0·86	620	72
Believes health facility is best place to give birth	Knowledge and attitudes	All respondents with a complete survey	0·021 (0·0036 to 0·039)	0·018	-0·013 (-0·050 to 0·024)	0·48	0·0080 (-0·028 to 0·044)	0·66	10262	90
**Pregnancy danger signs**										

Woman sought medical care for pregnancy danger sign	Practice	All respondents with a complete survey and a child born since 2018-09-01 (after the end of the intervention) who experienced a prenatal care danger sign	-0·026 (-0·23 to 0·18)	0·80	-0·081 (-0·24 to 0·076)	0·31	-0·11 (-0·33 to 0·12)	0·35	235	64
Identified bleeding as pregnancy d.s.	Knowledge and attitudes	All respondents with a complete survey	0·018 (-0·015 to 0·052)	0·28	0·021 (-0·017 to 0·058)	0·28	0·039 (-0·0039 to 0·082)	0·075	10260	41
Identified seizure as pregnancy d.s.	Knowledge and attitudes	All respondents with a complete survey	0·0048 (-0·0047 to 0·014)	0·32	-0·0020 (-0·0094 to 0·0054)	0·60	0·0028 (-0·0063 to 0·012)	0·54	10260	6
Identified headache as pregnancy d.s.	Knowledge and attitudes	All respondents with a complete survey	0·027 (-0·012 to 0·066)	0·18	0·0079 (-0·031 to 0·046)	0·69	0·035 (-0·017 to 0·086)	0·18	10260	39
Identified ringing in ears as pregnancy d.s.	Knowledge and attitudes	All respondents with a complete survey	0·0040 (-0·0053 to 0·013)	0·40	0·0010 (-0·0055 to 0·0074)	0·77	0·0050 (-0·0052 to 0·015)	0·34	10260	4
Identified dizziness as pregnancy d.s.	Knowledge and attitudes	All respondents with a complete survey	-0·014 (-0·048 to 0·020)	0·42	0·0031 (-0·042 to 0·048)	0·89	-0·011 (-0·060 to 0·038)	0·66	10260	37
Identified difficulty urinating as pregnancy d.s.	Knowledge and attitudes	All respondents with a complete survey	0·0024 (-0·0033 to 0·0081)	0·41	-0·0025 (-0·0095 to 0·0046)	0·49	-0·0001 (-0·0078 to 0·0077)	0·99	10260	2
Identified reduced or absent fetal movement as pregnancy d.s.	Knowledge and attitudes	All respondents with a complete survey	0·0087 (-0·0087 to 0·026)	0·33	0·0096 (-0·0044 to 0·024)	0·18	0·018 (-0·0048 to 0·042)	0·12	10260	6
Identified water breaking as pregnancy d.s.	Knowledge and attitudes	All respondents with a complete survey	-0·0025 (-0·0088 to 0·0037)	0·43	0·0024 (-0·0037 to 0·0084)	0·45	-0·0002 (-0·0074 to 0·0070)	0·96	10260	2
Identified fever as pregnancy d.s.	Knowledge and attitudes	All respondents with a complete survey	0·012 (-0·012 to 0·036)	0·34	0·027 (-0·0056 to 0·059)	0·11	0·039 (0·0013 to 0·076)	0·042	10260	14
Identified swelling of face/hands/feet as pregnancy d.s.	Knowledge and attitudes	All respondents with a complete survey	0·017 (-0·0095 to 0·044)	0·21	0·017 (-0·0061 to 0·040)	0·15	0·034 (0·0032 to 0·065)	0·030	10260	10
**Postnatal care for mother**										
Mother had health checked by professional within 3 days of birth	Practice	All respondents with a complete survey and a child born since 2018-09-01 (after the end of the intervention)	0·051 (-0·060 to 0·16)	0·37	0·088 (-0·055 to 0·23)	0·23	0·14 (-0·024 to 0·30)	0·094	605	14
Mother had health checked by professional within 7 days of birth	Practice	All respondents with a complete survey and a child born since 2018-09-01 (after the	0·00020 (-0·13 to 0·13)	1·00	0·12 (-0·0002 to 0·25)	0·050	0·12 (-0·037 to 0·28)	0·13	605	27

Oles W, *et al. BMJ Open* 2024; 14:e060784. doi: 10.1136/bmjopen-2022-060784

		end of the intervention)								
Mother sought medical care for postnatal danger sign	Practice	All respondents with a complete survey and a child born since 2018-09-01 (after the end of the intervention)	0·063 (-0·14 to 0·26)	0·54	0·16 (-0·020 to 0·33)	0·082	0·22 (-0·0038 to 0·44)	0·054	194	48
Identifies that mother should receive postnatal medical check-up within 3 days of birth	Knowledge and attitudes	All respondents with a complete survey	0·088 (0·058 to 0·12)	<0·0001	-0·0007 (-0·037 to 0·036)	0·97	0·088 (0·043 to 0·13)	0·0001	10261	9
Identifies that mother should receive postnatal medical check-up within 7 days of birth	Knowledge and attitudes	All respondents with a complete survey	0·061 (0·026 to 0·097)	0·0008	-0·0025 (-0·046 to 0·041)	0·91	0·059 (0·0086 to 0·11)	0·022	10261	24
Identified heavy vaginal bleeding as postnatal d.s.	Knowledge and attitudes	All respondents with a complete survey	0·047 (0·011 to 0·083)	0·010	0·0011 (-0·032 to 0·034)	0·95	0·048 (0·0074 to 0·088)	0·020	10260	43
Identified fever as postnatal d.s.	Knowledge and attitudes	All respondents with a complete survey	0·025 (-0·010 to 0·060)	0·17	0·026 (-0·012 to 0·063)	0·18	0·051 (0·0024 to 0·099)	0·040	10260	38
Identified vaginal discharge as postnatal d.s.	Knowledge and attitudes	All respondents with a complete survey	0·0083 (-0·0022 to 0·019)	0·12	-0·0055 (-0·017 to 0·0060)	0·35	0·0029 (-0·010 to 0·016)	0·67	10260	7
Identified headache as postnatal d.s.	Knowledge and attitudes	All respondents with a complete survey	0·019 (-0·0070 to 0·044)	0·15	0·0073 (-0·027 to 0·041)	0·67	0·026 (-0·013 to 0·065)	0·20	10260	22
Identified convulsions or fits as postnatal d.s.	Knowledge and attitudes	All respondents with a complete survey	0·0021 (-0·0021 to 0·0062)	0·33	-0·0019 (-0·0066 to 0·0028)	0·44	0·0002 (-0·0051 to 0·0056)	0·94	10260	1
**Postnatal care for newborn**										
Newborn had health checked by professional within 3 days of birth	Practice	All respondents with a complete survey and a child born since 2018-09-01 (after the end of the intervention)	0·087 (-0·026 to 0·20)	0·13	0·077 (-0·033 to 0·19)	0·17	0·16 (0·031 to 0·30)	0·016	610	15
Newborn had health checked by professional within 7 days of birth	Practice	All respondents with a complete survey and a child born since 2018-09-01 (after the end of the intervention)	0·079 (-0·050 to 0·21)	0·23	0·049 (-0·047 to 0·14)	0·32	0·13 (-0·014 to 0·27)	0·077	610	29
Newborn experienced health problem in first month, care was sought	Practice	All respondents with a complete survey and a child born since 2018-09-01 (after the	-0·081 (-0·34 to 0·18)	0·54	0·11 (-0·070 to 0·29)	0·23	0·030 (-0·22 to 0·28)	0·81	177	74

Oles W, *et al. BMJ Open* 2024; 14:e060784. doi: 10.1136/bmjopen-2022-060784

		end of the intervention)								
Identified correct ways to provide newborn care[a]	Knowledge and attitudes	All respondents with a complete survey	0·015 (0·0044 to 0·026)	0·0055	0·012 (-0·0023 to 0·026)	0·10	0·027 (0·010 to 0·043)	0·0014	10261	5
Identified fever as newborn d.s.	Knowledge and attitudes	All respondents with a complete survey	-0·023 (-0·062 to 0·016)	0·25	0·028 (-0·011 to 0·067)	0·15	0·0055 (-0·044 to 0·055)	0·83	10260	69
Identified diarrhea as newborn d.s.	Knowledge and attitudes	All respondents with a complete survey	0·0096 (-0·019 to 0·038)	0·51	-0·0002 (-0·038 to 0·037)	0·99	0·0093 (-0·032 to 0·050)	0·65	10260	49
Identified difficulty breathing as newborn d.s.	Knowledge and attitudes	All respondents with a complete survey	0·0099 (-0·010 to 0·030)	0·34	0·0033 (-0·028 to 0·035)	0·84	0·013 (-0·024 to 0·050)	0·48	10260	36
Identified vomiting as newborn d.s.	Knowledge and attitudes	All respondents with a complete survey	0·0018 (-0·018 to 0·021)	0·86	0·0084 (-0·017 to 0·034)	0·52	0·010 (-0·017 to 0·038)	0·47	10260	27
Identified poor appetite as newborn d.s.	Knowledge and attitudes	All respondents with a complete survey	-0·0001 (-0·011 to 0·011)	0·99	-0·0064 (-0·014 to 0·0012)	0·097	-0·0065 (-0·016 to 0·0032)	0·19	10260	6
Identified redness/bleeding around cord as newborn d.s.	Knowledge and attitudes	All respondents with a complete survey	0·0099 (-0·0027 to 0·023)	0·13	0·0047 (-0·014 to 0·024)	0·63	0·015 (-0·0056 to 0·035)	0·16	10260	5
Identified pus in cord as newborn d.s.	Knowledge and attitudes	All respondents with a complete survey	0·013 (0·0016 to 0·024)	0·025	0·0006 (-0·012 to 0·013)	0·93	0·013 (-0·0021 to 0·028)	0·092	10260	5
Identified cold skin as newborn d.s.	Knowledge and attitudes	All respondents with a complete survey	0·0042 (-0·0048 to 0·013)	0·36	-0·0025 (-0·0086 to 0·0037)	0·43	0·0017 (-0·0067 to 0·010)	0·69	10260	2
Identified cough as newborn d.s.	Knowledge and attitudes	All respondents with a complete survey	-0·034 (-0·063 to -0·0054)	0·020	0·015 (-0·026 to 0·057)	0·47	-0·019 (-0·065 to 0·027)	0·42	10260	2
Identified cold as newborn d.s.	Knowledge and attitudes	All respondents with a complete survey	-0·022 (-0·052 to 0·0085)	0·16	0·0028 (-0·045 to 0·050)	0·91	-0·019 (-0·074 to 0·036)	0·50	10260	1
Identified problems latching as newborn d.s.	Knowledge and attitudes	All respondents with a complete survey	0·0009 (-0·0080 to 0·0098)	0·84	-0·0051 (-0·015 to 0·0053)	0·34	-0·0041 (-0·017 to 0·0082)	0·51	10260	0
Identified pneumonia as newborn d.s.	Knowledge and attitudes	All respondents with a complete survey	0·0027 (-0·025 to 0·031)	0·85	-0·0037 (-0·037 to 0·029)	0·82	-0·0010 (-0·037 to 0·035)	0·95	10260	NA
Did not use harmful substances to treat cord stump	Practice	All respondents with a complete survey and a child born since 2018-09-01 (after the end of the intervention)	0·070 (-0·052 to 0·19)	0·26	0·022 (-0·098 to 0·14)	0·72	0·092 (-0·042 to 0·23)	0·18	598	83
Did not wrap fajero around newborn in first 7 days after birth	Practice	All respondents with a complete survey and a child born since 2018-09-01 (after the end of the intervention)	0·15 (0·050 to 0·26)	0·0038	0·043 (-0·100 to 0·19)	0·56	0·20 (0·042 to 0·35)	0·013	616	6

Identified proper cord care methods[b]	Knowledge and attitudes	All respondents with a complete survey	0·089 (0·056 to 0·12)	<0·0001	0·0015 (-0·044 to 0·047)	0·95	0·091 (0·040 to 0·14)	0·0005	10260	47
Correctly answered proper cord care riddle	Intervention knowledge	All respondents with a complete survey	0·17 (0·13 to 0·21)	<0·0001	0·019 (-0·027 to 0·066)	0·42	0·19 (0·14 to 0·24)	<0·0001	10262	NA
Kept newborn wrapped first 7 days after birth	Practice	All respondents with a complete survey and a child born since 2018-09-01 (after the end of the intervention)	-0·044 (-0·095 to 0·0080)	0·098	0·013 (-0·036 to 0·063)	0·59	-0·030 (-0·086 to 0·026)	0·29	619	99
Held newborn skin-to-skin during first month after birth	Practice	All respondents with a complete survey and a child born since 2018-09-01 (after the end of the intervention)	0·095 (-0·041 to 0·23)	0·17	0·032 (-0·080 to 0·14)	0·57	0·13 (-0·014 to 0·27)	0·077	617	100
**Breastfeeding**										
Exclusively breastfed child first 6 months	Practice	All respondents with a complete survey and a child born since 2018-09-01 (after the end of the intervention)	0·053 (-0·085 to 0·19)	0·45	-0·032 (-0·16 to 0·091)	0·61	0·021 (-0·14 to 0·18)	0·80	619	59
Exclusively breastfed child first 6 months without giving chupón	Practice	All respondents with a complete survey and a child born since 2018-09-01 (after the end of the intervention)	0·086 (-0·046 to 0·22)	0·20	0·012 (-0·10 to 0·12)	0·84	0·097 (-0·048 to 0·24)	0·19	619	34
Breastfed immediately after birth	Practice	All respondents with a complete survey and a child born since 2018-09-01 (after the end of the intervention) who reported a natural birth	0·13 (0·047 to 0·21)	0·0023	-0·042 (-0·12 to 0·035)	0·29	0·089 (0·0028 to 0·17)	0·043	493	86
Identifies that newborn should be breastfed immediately after birth	Knowledge and attitudes	All respondents with a complete survey	0·0045 (-0·019 to 0·028)	0·70	0·010 (-0·016 to 0·037)	0·45	0·015 (-0·018 to 0·048)	0·38	10261	82
Identifies that newborns should only be given breast milk during first 6 months	Knowledge and attitudes	All respondents with a complete survey	0·042 (0·017 to 0·067)	0·0008	-0·015 (-0·041 to 0·011)	0·25	0·027 (-0·0048 to 0·059)	0·096	10261	80

Believes newborns should not be given chupón during first 6 months	Knowledge and attitudes	All respondents with a complete survey	0·15 (0·11 to 0·19)	<0·0001	-0·011 (-0·060 to 0·038)	0·67	0·14 (0·084 to 0·20)	<0·0001	10261	23
**Paternal involvement**										
Father accompanied mother to clinic for prenatal care visit at least once	Practice	All respondents with a complete survey and a child born since 2018-09-01 (after the end of the intervention) who received prenatal care	0·018 (-0·12 to 0·15)	0·79	-0·064 (-0·21 to 0·087)	0·41	-0·045 (-0·22 to 0·13)	0·62	595	52
Father accompanied mother to seek medical care for pregnancy danger sign	Practice	All respondents with a complete survey and a child born since 2018-09-01 (after the end of the intervention) who experienced a prenatal care danger sign and sought care	0·010 (-0·15 to 0·17)	0·90	-0·0013 (-0·18 to 0·18)	0·99	0·0091 (-0·21 to 0·23)	0·94	149	65
Father waited at birthplace during labor	Practice	All respondents with a complete survey and a child born since 2018-09-01 (after the end of the intervention)	-0·031 (-0·14 to 0·075)	0·56	0·0083 (-0·12 to 0·13)	0·90	-0·023 (-0·16 to 0·11)	0·74	620	78
Father accompanied mother to seek medical care for postnatal danger sign	Practice	All respondents with a complete survey and a child born since 2018-09-01 (after the end of the intervention) and sought care for postpartum problem	0·18 (-0·032 to 0·39)	0·096	-0·037 (-0·32 to 0·24)	0·80	0·14 (-0·17 to 0·45)	0·37	97	67
Father sought medical care for newborn for postnatal danger sign	Practice	All respondents with a complete survey and a child born since 2018-09-01 (after the end of the intervention) and sought care for a postnatal problem	0·0059 (-0·33 to 0·34)	0·97	-0·099 (-0·37 to 0·17)	0·48	-0·093 (-0·44 to 0·25)	0·60	116	73
Father held child	Practice	All respondents with a complete survey and a child born since 2018-	-0·046 (-0·11 to 0·022)	0·19	0·078 (0·013 to 0·14)	0·020	0·032 (-0·040 to 0·10)	0·38	614	97

		09-01 (after the end of the intervention)								
Father played with child	Practice	All respondents with a complete survey and a child born since 2018-09-01 (after the end of the intervention)	-0·091 (-0·19 to 0·0099)	0·077	0·076 (-0·030 to 0·18)	0·16	-0·015 (-0·13 to 0·10)	0·80	614	90
Father took child to clinic when sick	Practice	All respondents with a complete survey and a child born since 2018-09-01 (after the end of the intervention)	-0·053 (-0·19 to 0·080)	0·44	0·084 (-0·011 to 0·18)	0·082	0·031 (-0·11 to 0·17)	0·66	614	71
Believes father should accompany mother to prenatal care visits	Knowledge and attitudes	All respondents with a complete survey	0·041 (0·022 to 0·060)	<0·0001	0·0024 (-0·022 to 0·027)	0·85	0·043 (0·019 to 0·068)	0·0005	10220	93
Believes father should wait at birth location while mother gives birth	Knowledge and attitudes	All respondents with a complete survey	0·0044 (-0·0078 to 0·017)	0·48	0·0070 (-0·0079 to 0·022)	0·36	0·011 (-0·0062 to 0·029)	0·20	10225	94
Believes father should care for children when sick	Knowledge and attitudes	All respondents with a complete survey	-0·0012 (-0·0069 to 0·0044)	0·67	-0·0015 (-0·0074 to 0·0043)	0·60	-0·0028 (-0·010 to 0·0047)	0·47	10250	99
**Diarrhea management**										
Did not report diarrhea in past 4 weeks	Practice	All respondents with a complete survey	-0·0010 (-0·014 to 0·012)	0·87	-0·0086 (-0·019 to 0·0021)	0·12	-0·0096 (-0·025 to 0·0060)	0·23	10263	97
Used ORT to treat diarrhea in past 4 weeks	Practice	All respondents with a complete survey who had diarrhea in past 4 weeks	-0·14 (-0·30 to 0·022)	0·091	-0·044 (-0·20 to 0·12)	0·59	-0·19 (-0·38 to 0·011)	0·064	266	52
Used appropriate treatment for diarrhea in past 4 weeks[c]	Practice	All respondents with a complete survey who had diarrhea in past 4 weeks	-0·015 (-0·16 to 0·13)	0·84	0·012 (-0·12 to 0·15)	0·86	-0·0027 (-0·16 to 0·16)	0·97	268	28
Child did not experience diarrhea past 4 weeks	Practice	All respondents with a complete survey and a new child <= 5 years old reported at wave 3 who had diarrhea in past 4 weeks	0·0042 (-0·025 to 0·034)	0·78	-0·011 (-0·044 to 0·023)	0·53	-0·0064 (-0·043 to 0·030)	0·73	3333	84
Child experienced diarrhea past 4 weeks, was given appropriate amount of liquids	Practice	All respondents with a complete survey and a new child <= 5 years old reported at	-0·100 (-0·25 to 0·046)	0·18	-0·031 (-0·20 to 0·14)	0·73	-0·13 (-0·31 to 0·050)	0·16	350	50

		wave 3 who had diarrhea in past 4 weeks								
Child experienced diarrhea past 4 weeks, was given appropriate amount of food	Practice	All respondents with a complete survey and a new child <= 5 years old reported at wave 3 who had diarrhea in past 4 weeks	-0·088 (-0·20 to 0·028)	0·14	-0·024 (-0·15 to 0·099)	0·70	-0·11 (-0·25 to 0·024)	0·11	350	35
Child experienced diarrhea past 4 weeks, was given ORT	Practice	All respondents with a complete survey and a new child <= 5 years old reported at wave 3 who had diarrhea in past 4 weeks	0·082 (-0·069 to 0·23)	0·29	0·056 (-0·089 to 0·20)	0·45	0·14 (-0·032 to 0·31)	0·11	347	60
Child experienced diarrhea past 4 weeks, was given appropriate treatment	Practice	All respondents with a complete survey and a new child <= 5 years old reported at wave 3 who had diarrhea in past 4 weeks	0·027 (-0·10 to 0·15)	0·68	0·076 (-0·056 to 0·21)	0·26	0·10 (-0·059 to 0·27)	0·21	348	31
Child experienced diarrhea past 4 weeks, was given zinc 10-14 days	Practice	All respondents with a complete survey and a new child <= 5 years old reported at wave 3 who had diarrhea in past 4 weeks	0·0080 (-0·030 to 0·046)	0·68	0·15 (0·0026 to 0·29)	0·046	0·16 (-0·014 to 0·33)	0·072	311	1
Identified appropriate diarrhea treatment methods[d]	Knowledge and attitudes	All respondents with a complete survey	0·083 (0·045 to 0·12)	<0·0001	0·014 (-0·030 to 0·058)	0·54	0·097 (0·042 to 0·15)	0·0005	10260	41
Identified zinc 10-14 days as diarrhea treatment	Knowledge and attitudes	All respondents with a complete survey	0·0093 (-0·0073 to 0·026)	0·27	0·020 (-0·0005 to 0·040)	0·056	0·029 (0·0079 to 0·050)	0·0072	10261	4
Identified zinc supplement as way to prevent diarrhea	Knowledge and attitudes	All respondents with a complete survey	0·063 (0·035 to 0·090)	<0·0001	-0·0004 (-0·066 to 0·065)	0·99	0·062 (-0·0063 to 0·13)	0·075	10261	45
Identified breastfeeding as way to prevent diarrhea	Knowledge and attitudes	All respondents with a complete survey	0·031 (0·0028 to 0·060)	0·032	0·0070 (-0·058 to 0·072)	0·83	0·038 (-0·028 to 0·10)	0·26	10261	26
Correctly answered diarrhea treatment with zinc riddle	Intervention knowledge	All respondents with a complete survey	0·20 (0·16 to 0·24)	<0·0001	0·019 (-0·035 to 0·074)	0·49	0·22 (0·16 to 0·28)	<0·0001	10261	NA
**Respiratory illness**										

Did not report respiratory illness (coughing) for 2 weeks	Practice	All respondents with a complete survey	0·0005 (-0·022 to 0·023)	0·97	-0·017 (-0·045 to 0·011)	0·23	-0·017 (-0·050 to 0·017)	0·33	10263	79
Child did not have cough past 4 weeks	Practice	All respondents with a complete survey	0·0076 (-0·037 to 0·052)	0·74	-0·070 (-0·11 to -0·029)	0·0009	-0·063 (-0·12 to -0·0051)	0·033	3334	67
Child had cough past 4 weeks, care was sought	Practice	All respondents with a complete survey and a new child <= 5 years old reported at wave 3 who had an illness with a cough in past 4 weeks	-0·041 (-0·14 to 0·055)	0·40	0·070 (-0·033 to 0·17)	0·18	0·029 (-0·092 to 0·15)	0·64	700	4
Identified vaccination as way to prevent respiratory illness	Knowledge and attitudes	All respondents with a complete survey	0·021 (-0·00010 to 0·042)	0·051	-0·042 (-0·080 to -0·0035)	0·033	-0·021 (-0·059 to 0·017)	0·28	10261	51
Identified breastfeeding as way to prevent respiratory illness	Knowledge and attitudes	All respondents with a complete survey	0·015 (-0·013 to 0·042)	0·29	-0·0010 (-0·052 to 0·050)	0·97	0·014 (-0·040 to 0·068)	0·62	10261	34
Identified fever as d.s. for children with respiratory illness	Knowledge and attitudes	All respondents with a complete survey	0·0067 (-0·0067 to 0·020)	0·33	-0·0023 (-0·032 to 0·027)	0·88	0·0044 (-0·028 to 0·037)	0·79	10261	82
Identified difficulty breathing as d.s. for children with respiratory illness	Knowledge and attitudes	All respondents with a complete survey	0·011 (0·0032 to 0·019)	0·0057	-0·0064 (-0·023 to 0·011)	0·46	0·0046 (-0·014 to 0·023)	0·62	10261	89
Identified rapid breathing as d.s. for children with respiratory illness	Knowledge and attitudes	All respondents with a complete survey	0·0073 (0·0012 to 0·013)	0·019	-0·0025 (-0·014 to 0·0087)	0·67	0·0048 (-0·0070 to 0·017)	0·42	10261	85
**Reproductive health**										
Reported ever using birth control to delay or avoid pregnancy	Practice	All respondents with a complete survey	0·015 (-0·021 to 0·052)	0·41	-0·033 (-0·084 to 0·019)	0·21	-0·018 (-0·073 to 0·038)	0·53	10257	33
Reported currently using birth control	Practice	All respondents with a complete survey who are not pregnant and have ever used birth control	0·014 (-0·039 to 0·066)	0·61	0·035 (-0·025 to 0·095)	0·26	0·048 (-0·023 to 0·12)	0·19	3793	55
Believes woman should be at least 18 years of age to have her first child	Knowledge and attitudes	All respondents with a complete survey	0·0054 (-0·0010 to 0·012)	0·096	0·0056 (-0·0022 to 0·014)	0·16	0·011 (0·0021 to 0·020)	0·015	9733	98
**Gender/reproductive norms**										
Birth location chosen either jointly or by woman	Practice	All respondents with a complete survey and a child born since 2018-09-01 (after the	0·0093 (-0·061 to 0·080)	0·80	0·039 (-0·045 to 0·12)	0·36	0·048 (-0·054 to 0·15)	0·36	620	93

		end of the intervention)								

Results based on final sample of N=10263 (household targeted n=3238, household not targeted n=7025). d.s.=Danger sign, NA=Not applicable, ORT=Oral rehydration therapy. Proportions with NA could not be assessed because the outcome was not part of the baseline instrument. Standard errors are clustered at the village level. Robust 95% CIs in parentheses.

[a] 'Identified correct ways to provide newborn care' outcome was coded as correct if the respondent identified holding baby skin to skin, getting their health checked by a professional, immediately putting them to the breast, or making sure their diaper is clean and dry, and did not identify giving purgante, wrapping a fajero, giving a chupon, or bathing them in water right away.
[b] 'Identified proper cord care' outcome was coded as correct if respondent identified applying alcohol, applying iodine, keeping cord clean/dry, or applying peroxide, and did not identify applying oil, applying coffee, applying quina water, wrapping a fajero, applying powder, burning, applying ashes, applying a clamp, or applying thiomersal.
[c] 'Used appropriate treatment for diarrhea in past 4 weeks' outcome was coded as correct if respondent did any of the following: antibiotic, zinc, give extra food, give extra liquid, and did not do any of the following: anti-diarrhea medication, home remedy, laxative, deworming medication, chupon, massage, stop eating foods, stop taking liquids.
[d] 'Identified appropriate diarrhea treatment methods' outcome was coded as correct if respondent identified any of the following: antibiotic, zinc, ORS or Litrosol, give extra food, give extra liquid, get medical attention, and did not identify any of the following: anti-diarrhea medication, laxative, deworming medication, home remedy, chupon, massage, stop giving foods, stop giving liquids, or do nothing.

Table S2. Pooled total effect estimates of intervention on maternal, child, and neonatal outcomes, mothers responses for practice outcomes only

	Pooled total effect estimate		N
	Effect size (95% CI)	p value	
**Prenatal care**			
Currently taking folic acid tablets (daily in past 7 days)	0·0004 (-0·043 to 0·044)	0·98	6311
Made a birth plan in preparation for birth	0·031 (-0·11 to 0·17)	0·66	412
Saved money in preparation for birth	-0·039 (-0·17 to 0·091)	0·56	412
Sold animal in preparation for birth	-0·014 (-0·097 to 0·070)	0·75	412
Sought prenatal care within 12 weeks	-0·031 (-0·19 to 0·12)	0·69	407
**Facility based birth**			
Gave birth in health facility	-0·049 (-0·19 to 0·094)	0·50	412
**Pregnancy danger signs**			
Woman sought medical care for pregnancy danger sign	-0·12 (-0·41 to 0·16)	0·39	152
**Postnatal care for mother**			
Mother had health checked by professional within 3 days of birth	0·14 (-0·038 to 0·32)	0·12	411
Mother had health checked by professional within 7 days of birth	0·11 (-0·058 to 0·27)	0·20	411
Mother sought medical care for postnatal danger sign	0·22 (-0·014 to 0·46)	0·066	131
**Postnatal care for newborn**			
Newborn had health checked by professional within 3 days of birth	0·18 (0·015 to 0·34)	0·032	411
Newborn had health checked by professional within 7 days of birth	0·11 (-0·049 to 0·27)	0·17	411
Newborn experienced health problem in first month, care was sought	-0·0022 (-0·29 to 0·29)	0·99	129
Did not use harmful substances to treat cord stump	0·054 (-0·10 to 0·21)	0·50	410
Did not wrap fajero around newborn in first 7 days after birth	0·24 (0·069 to 0·41)	0·0060	411
Kept newborn wrapped first 7 days after birth	-0·0026 (-0·059 to 0·054)	0·93	412
Held newborn skin-to-skin during first month after birth	0·15 (-0·013 to 0·31)	0·073	411
**Breastfeeding**			
Exclusively breastfed child first 6 months	0·061 (-0·10 to 0·22)	0·47	412
Exclusively breastfed child first 6 months without giving chupón	0·14 (-0·016 to 0·29)	0·079	412
Breastfed immediately after birth	0·10 (-0·0032 to 0·20)	0·057	335

Oles W, *et al. BMJ Open* 2024; 14:e060784. doi: 10.1136/bmjopen-2022-060784

**Paternal involvement**			
Father accompanied mother to clinic for prenatal care visit at least once	-0·060 (-0·26 to 0·14)	0·55	394
Father accompanied mother to seek medical care for pregnancy danger sign	0·11 (-0·15 to 0·38)	0·41	94
Father waited at birthplace during labor	-0·017 (-0·17 to 0·14)	0·83	412
Father accompanied mother to seek medical care for postnatal danger sign	0·22 (-0·10 to 0·54)	0·18	66
Father sought medical care for newborn for postnatal danger sign	-0·028 (-0·42 to 0·37)	0·89	83
Father held child	0·054 (-0·038 to 0·14)	0·25	406
Father played with child	0·025 (-0·13 to 0·18)	0·75	406
Father took child to clinic when sick	0·059 (-0·11 to 0·23)	0·51	406
**Diarrhea management**			
Did not report diarrhea in past 4 weeks	-0·013 (-0·034 to 0·0070)	0·20	6318
Used ORT to treat diarrhea in past 4 weeks	-0·14 (-0·39 to 0·12)	0·29	181
Used appropriate treatment for diarrhea in past 4 weeks[a]	0·069 (-0·16 to 0·30)	0·55	183
Child did not experience diarrhea past 4 weeks	-0·0035 (-0·040 to 0·033)	0·85	2891
Child experienced diarrhea past 4 weeks, was given appropriate amount of liquids	-0·13 (-0·32 to 0·064)	0·19	291
Child experienced diarrhea past 4 weeks, was given appropriate amount of food	-0·11 (-0·25 to 0·032)	0·13	291
Child experienced diarrhea past 4 weeks, was given ORT	0·13 (-0·052 to 0·32)	0·16	289
Child experienced diarrhea past 4 weeks, was given appropriate treatment	0·090 (-0·081 to 0·26)	0·30	291
Child experienced diarrhea past 4 weeks, was given zinc 10-14 days	0·16 (-0·033 to 0·35)	0·10	260
**Respiratory illness**			
Did not report respiratory illness (coughing) for 2 weeks	-0·040 (-0·082 to 0·0022)	0·063	6318
Child did not have cough past 4 weeks	-0·061 (-0·12 to 0·0012)	0·055	2892
Child had cough past 4 weeks, care was sought	0·055 (-0·068 to 0·18)	0·38	616
**Reproductive health**			
Reported ever using birth control to delay or avoid pregnancy	0·020 (-0·041 to 0·081)	0·52	6316
Reported currently using birth control	0·030 (-0·061 to 0·12)	0·52	2570
**Gender/reproductive norms**			
Birth location chosen either jointly or by woman	0·032 (-0·092 to 0·16)	0·62	412

ORT=Oral rehydration therapy. Standard errors are clustered at the village level. Robust 95% CIs in parentheses.

[a] 'Used appropriate treatment for diarrhea in past 4 weeks' outcome was coded as correct if respondent did any of the following: antibiotic, zinc, give extra food, give extra liquid, and did not do any of the following: anti-diarrhea medication, home remedy, laxative, deworming medication, chupon, massage, stop eating foods, stop taking liquids.

Table S3. Pooled total effect estimates of intervention on maternal, child, and neonatal outcomes, estimated on sub-sample of respondents who did not move between villages during study

	Outcome type	Respondent denominator	Pooled total effect estimate		N
			Effect size (95% CI)	p value	
**Prenatal care**					
Currently taking folic acid tablets (daily in past 7 days)	Practice	All women 15+ with a complete survey	-0·0029 (-0·048 to 0·042)	0·90	6035
Made a birth plan in preparation for birth	Practice	All respondents with a complete survey and a child born since 2018-09-01 (after the end of the intervention)	0·070 (-0·022 to 0·16)	0·13	567
Saved money in preparation for birth	Practice	All respondents with a complete survey and a child born since 2018-09-01 (after the end of the intervention)	-0·012 (-0·14 to 0·11)	0·85	568
Sold animal in preparation for birth	Practice	All respondents with a complete survey and a child born since 2018-09-01 (after the end of the intervention)	-0·017 (-0·086 to 0·052)	0·62	567
Sought prenatal care within 12 weeks	Practice	All respondents with a complete survey and a child born since 2018-09-01 (after the end of the intervention)	-0·052 (-0·20 to 0·097)	0·50	547
Identifies that women should take folic acid before pregnancy	Knowledge and attitudes	All respondents with a complete survey	0·042 (-0·0008 to 0·084)	0·054	9917
Identifies that women should seek prenatal care first 12 weeks of pregnancy	Knowledge and attitudes	All respondents with a complete survey	0·020 (-0·0077 to 0·048)	0·16	9917
Identifies accompanying woman to prenatal care visits as method of support during pregnancy	Knowledge and attitudes	All respondents with a complete survey	-0·0039 (-0·058 to 0·051)	0·89	9913
Identifies ensuring that woman eats well as method of support during pregnancy	Knowledge and attitudes	All respondents with a complete survey	-0·016 (-0·069 to 0·037)	0·56	9913
Identifies ensuring that woman rests 1 hour per day well as method of support during pregnancy	Knowledge and attitudes	All respondents with a complete survey	0·0091 (-0·024 to 0·042)	0·59	9913
Identifies avoiding violence as method of support during pregnancy	Knowledge and attitudes	All respondents with a complete survey	-0·034 (-0·055 to -0·013)	0·0018	9913
Identifies encouraging women to take vitamins as method of support during pregnancy	Knowledge and attitudes	All respondents with a complete survey	-0·022 (-0·060 to 0·017)	0·27	9913
Identifies helping woman with house work/child care as method of support during pregnancy	Knowledge and attitudes	All respondents with a complete survey	0·074 (0·0092 to 0·14)	0·025	9913
Identifies saving animals to sell as method of preparing for birth expenses	Knowledge and attitudes	All respondents with a complete survey	0·032 (0·0013 to 0·063)	0·041	9917

Identifies knowing cost of trip to maternal clinic as method of preparing for birth expenses	Knowledge and attitudes	All respondents with a complete survey	-0·0050 (-0·037 to 0·027)	0·76	9917
Identifies having a savings plan as method of preparing for birth expenses	Knowledge and attitudes	All respondents with a complete survey	0·033 (0·0025 to 0·063)	0·034	9917
Correctly answered prenatal care knowledge riddle	Intervention knowledge	All respondents with a complete survey	0·078 (0·039 to 0·12)	0·0001	9916
**Facility based birth**					
Gave birth in health facility	Practice	All respondents with a complete survey and a child born since 2018-09-01 (after the end of the intervention)	-0·0002 (-0·15 to 0·15)	1·00	568
Believes health facility is best place to give birth	Knowledge and attitudes	All respondents with a complete survey	0·0074 (-0·030 to 0·044)	0·69	9917
**Pregnancy danger signs**					
Woman sought medical care for pregnancy danger sign	Practice	All respondents with a complete survey and a child born since 2018-09-01 (after the end of the intervention) who experienced a prenatal care danger sign	-0·12 (-0·36 to 0·12)	0·33	210
Identified bleeding as pregnancy d.s.	Knowledge and attitudes	All respondents with a complete survey	0·040 (-0·0049 to 0·084)	0·081	9916
Identified seizure as pregnancy d.s.	Knowledge and attitudes	All respondents with a complete survey	0·0030 (-0·0064 to 0·012)	0·53	9916
Identified headache as pregnancy d.s.	Knowledge and attitudes	All respondents with a complete survey	0·040 (-0·014 to 0·093)	0·14	9916
Identified ringing in ears as pregnancy d.s.	Knowledge and attitudes	All respondents with a complete survey	0·0053 (-0·0054 to 0·016)	0·33	9916
Identified dizziness as pregnancy d.s.	Knowledge and attitudes	All respondents with a complete survey	-0·013 (-0·064 to 0·038)	0·61	9916
Identified difficulty urinating as pregnancy d.s.	Knowledge and attitudes	All respondents with a complete survey	-0·0002 (-0·0082 to 0·0077)	0·95	9916
Identified reduced or absent fetal movement as pregnancy d.s.	Knowledge and attitudes	All respondents with a complete survey	0·019 (-0·0052 to 0·043)	0·13	9916
Identified water breaking as pregnancy d.s.	Knowledge and attitudes	All respondents with a complete survey	-0·0022 (-0·0091 to 0·0047)	0·53	9916
Identified fever as pregnancy d.s.	Knowledge and attitudes	All respondents with a complete survey	0·036 (-0·0024 to 0·075)	0·066	9916
Identified swelling of face/hands/feet as pregnancy d.s.	Knowledge and attitudes	All respondents with a complete survey	0·035 (0·0030 to 0·067)	0·032	9916
**Postnatal care for mother**					
Mother had health checked by professional within 3 days of birth	Practice	All respondents with a complete survey and a child born since 2018-09-01 (after the end of the intervention)	0·12 (-0·047 to 0·30)	0·15	555

Oles W, *et al. BMJ Open* 2024; 14:e060784. doi: 10.1136/bmjopen-2022-060784

Mother had health checked by professional within 7 days of birth	Practice	All respondents with a complete survey and a child born since 2018-09-01 (after the end of the intervention)	0·10 (-0·072 to 0·27)	0·25	555
Mother sought medical care for postnatal danger sign	Practice	All respondents with a complete survey and a child born since 2018-09-01 (after the end of the intervention)	0·21 (-0·019 to 0·45)	0·071	178
Identifies that mother should receive postnatal medical check-up within 3 days of birth	Knowledge and attitudes	All respondents with a complete survey	0·087 (0·041 to 0·13)	0·0002	9917
Identifies that mother should receive postnatal medical check-up within 7 days of birth	Knowledge and attitudes	All respondents with a complete survey	0·060 (0·0079 to 0·11)	0·024	9917
Identified heavy vaginal bleeding as postnatal d.s.	Knowledge and attitudes	All respondents with a complete survey	0·053 (0·012 to 0·094)	0·011	9915
Identified fever as postnatal d.s.	Knowledge and attitudes	All respondents with a complete survey	0·049 (0·0007 to 0·098)	0·047	9915
Identified vaginal discharge as postnatal d.s.	Knowledge and attitudes	All respondents with a complete survey	0·0026 (-0·010 to 0·015)	0·68	9915
Identified headache as postnatal d.s.	Knowledge and attitudes	All respondents with a complete survey	0·029 (-0·011 to 0·069)	0·16	9915
Identified convulsions or fits as postnatal d.s.	Knowledge and attitudes	All respondents with a complete survey	0·0006 (-0·0050 to 0·0062)	0·84	9915
**Postnatal care for newborn**					
Newborn had health checked by professional within 3 days of birth	Practice	All respondents with a complete survey and a child born since 2018-09-01 (after the end of the intervention)	0·15 (0·016 to 0·29)	0·029	560
Newborn had health checked by professional within 7 days of birth	Practice	All respondents with a complete survey and a child born since 2018-09-01 (after the end of the intervention)	0·11 (-0·036 to 0·26)	0·14	560
Newborn experienced health problem in first month, care was sought	Practice	All respondents with a complete survey and a child born since 2018-09-01 (after the end of the intervention)	0·067 (-0·20 to 0·33)	0·62	160
Identified correct ways to provide newborn care[a]	Knowledge and attitudes	All respondents with a complete survey	0·026 (0·0096 to 0·043)	0·0019	9917
Identified fever as newborn d.s.	Knowledge and attitudes	All respondents with a complete survey	0·0054 (-0·046 to 0·057)	0·84	9915
Identified diarrhea as newborn d.s.	Knowledge and attitudes	All respondents with a complete survey	0·015 (-0·028 to 0·057)	0·50	9915
Identified difficulty breathing as newborn d.s.	Knowledge and attitudes	All respondents with a complete survey	0·0085 (-0·029 to 0·046)	0·66	9915
Identified vomiting as newborn d.s.	Knowledge and attitudes	All respondents with a complete survey	0·011 (-0·017 to 0·038)	0·45	9915
Identified poor appetite as newborn d.s.	Knowledge and attitudes	All respondents with a complete survey	-0·0062 (-0·016 to 0·0039)	0·23	9915
Identified redness/bleeding around cord as newborn d.s.	Knowledge and attitudes	All respondents with a complete survey	0·016 (-0·0053 to 0·037)	0·14	9915
Identified pus in cord as newborn d.s.	Knowledge and attitudes	All respondents with a complete survey	0·013 (-0·0024 to 0·029)	0·097	9915
Identified cold skin as newborn d.s.	Knowledge and attitudes	All respondents with a complete survey	0·0018 (-0·0069 to 0·011)	0·68	9915

Identified cough as newborn d.s.	Knowledge and attitudes	All respondents with a complete survey	-0·018 (-0·065 to 0·029)	0·45	9915
Identified cold as newborn d.s.	Knowledge and attitudes	All respondents with a complete survey	-0·016 (-0·072 to 0·040)	0·58	9915
Identified problems latching as newborn d.s.	Knowledge and attitudes	All respondents with a complete survey	-0·0029 (-0·016 to 0·010)	0·67	9915
Identified pneumonia as newborn d.s.	Knowledge and attitudes	All respondents with a complete survey	-0·0009 (-0·039 to 0·037)	0·96	9915
Did not use harmful substances to treat cord stump	Practice	All respondents with a complete survey and a child born since 2018-09-01 (after the end of the intervention)	0·098 (-0·043 to 0·24)	0·17	546
Did not wrap fajero around newborn in first 7 days after birth	Practice	All respondents with a complete survey and a child born since 2018-09-01 (after the end of the intervention)	0·20 (0·038 to 0·37)	0·016	565
Identified proper cord care methods[b]	Knowledge and attitudes	All respondents with a complete survey	0·097 (0·046 to 0·15)	0·0002	9916
Correctly answered proper cord care riddle	Intervention knowledge	All respondents with a complete survey	0·19 (0·13 to 0·24)	<0·0001	9917
Kept newborn wrapped first 7 days after birth	Practice	All respondents with a complete survey and a child born since 2018-09-01 (after the end of the intervention)	-0·040 (-0·10 to 0·022)	0·21	567
Held newborn skin-to-skin during first month after birth	Practice	All respondents with a complete survey and a child born since 2018-09-01 (after the end of the intervention)	0·15 (-0·0050 to 0·30)	0·058	565
**Breastfeeding**					
Exclusively breastfed child first 6 months	Practice	All respondents with a complete survey and a child born since 2018-09-01 (after the end of the intervention)	0·0034 (-0·16 to 0·17)	0·97	567
Exclusively breastfed child first 6 months without giving chupón	Practice	All respondents with a complete survey and a child born since 2018-09-01 (after the end of the intervention)	0·10 (-0·049 to 0·26)	0·18	567
Breastfed immediately after birth	Practice	All respondents with a complete survey and a child born since 2018-09-01 (after the end of the intervention) who reported a natural birth	0·12 (0·035 to 0·21)	0·0055	452
Identifies that newborn should be breastfed immediately after birth	Knowledge and attitudes	All respondents with a complete survey	0·018 (-0·015 to 0·051)	0·28	9917
Identifies that newborns should only be given breast milk during first 6 months	Knowledge and attitudes	All respondents with a complete survey	0·028 (-0·0042 to 0·061)	0·088	9916
Believes newborns should not be given chupón during first 6 months	Knowledge and attitudes	All respondents with a complete survey	0·14 (0·082 to 0·20)	<0·0001	9917
**Paternal involvement**					
Father accompanied mother to clinic for prenatal care visit at least once	Practice	All respondents with a complete survey and a child born since 2018-09-01 (after the end of the intervention) who received prenatal care	-0·053 (-0·25 to 0·14)	0·60	544
Father accompanied mother to seek medical care for pregnancy danger sign	Practice	All respondents with a complete survey and a child born since 2018-09-01 (after the end of the intervention) who experienced a prenatal care danger sign and sought care	-0·073 (-0·30 to 0·16)	0·53	134

Oles W, *et al. BMJ Open* 2024; 14:e060784. doi: 10.1136/bmjopen-2022-060784

Father waited at birthplace during labor	Practice	All respondents with a complete survey and a child born since 2018-09-01 (after the end of the intervention)	-0·036 (-0·18 to 0·11)	0·63	568
Father accompanied mother to seek medical care for postnatal danger sign	Practice	All respondents with a complete survey and a child born since 2018-09-01 (after the end of the intervention) and sought care for postpartum problem	0·13 (-0·19 to 0·45)	0·43	90
Father sought medical care for newborn for postnatal danger sign	Practice	All respondents with a complete survey and a child born since 2018-09-01 (after the end of the intervention) and sought care for a postnatal problem	-0·060 (-0·43 to 0·31)	0·75	103
Father held child	Practice	All respondents with a complete survey and a child born since 2018-09-01 (after the end of the intervention)	0·033 (-0·044 to 0·11)	0·40	563
Father played with child	Practice	All respondents with a complete survey and a child born since 2018-09-01 (after the end of the intervention)	-0·023 (-0·15 to 0·11)	0·73	563
Father took child to clinic when sick	Practice	All respondents with a complete survey and a child born since 2018-09-01 (after the end of the intervention)	0·014 (-0·13 to 0·16)	0·85	563
Believes father should accompany mother to prenatal care visits	Knowledge and attitudes	All respondents with a complete survey	0·044 (0·019 to 0·069)	0·0006	9876
Believes father should wait at birth location while mother gives birth	Knowledge and attitudes	All respondents with a complete survey	0·011 (-0·0068 to 0·029)	0·22	9881
Believes father should care for children when sick	Knowledge and attitudes	All respondents with a complete survey	-0·0025 (-0·010 to 0·0051)	0·52	9906
**Diarrhea management**					
Did not report diarrhea in past 4 weeks	Practice	All respondents with a complete survey	-0·0097 (-0·026 to 0·0066)	0·24	9918
Used ORT to treat diarrhea in past 4 weeks	Practice	All respondents with a complete survey who had diarrhea in past 4 weeks	-0·19 (-0·39 to 0·0012)	0·051	263
Used appropriate treatment for diarrhea in past 4 weeks[c]	Practice	All respondents with a complete survey who had diarrhea in past 4 weeks	-0·0006 (-0·16 to 0·16)	0·99	265
Child did not experience diarrhea past 4 weeks	Practice	All respondents with a complete survey and a new child <= 5 years old reported at wave 3 who had diarrhea in past 4 weeks	-0·0045 (-0·042 to 0·033)	0·82	3150
Child experienced diarrhea past 4 weeks, was given appropriate amount of liquids	Practice	All respondents with a complete survey and a new child <= 5 years old reported at wave 3 who had diarrhea in past 4 weeks	-0·15 (-0·34 to 0·037)	0·12	327
Child experienced diarrhea past 4 weeks, was given appropriate amount of food	Practice	All respondents with a complete survey and a new child <= 5 years old reported at wave 3 who had diarrhea in past 4 weeks	-0·12 (-0·26 to 0·015)	0·081	327
Child experienced diarrhea past 4 weeks, was given ORT	Practice	All respondents with a complete survey and a new child <= 5 years old reported at wave 3 who had diarrhea in past 4 weeks	0·12 (-0·054 to 0·30)	0·17	324
Child experienced diarrhea past 4 weeks, was given appropriate treatment	Practice	All respondents with a complete survey and a new child <= 5 years old reported at wave 3 who had diarrhea in past 4 weeks	0·12 (-0·056 to 0·29)	0·19	326
Child experienced diarrhea past 4 weeks, was given zinc 10-14 days	Practice	All respondents with a complete survey and a new child <= 5 years old reported at wave 3 who had diarrhea in past 4 weeks	0·17 (-0·014 to 0·35)	0·071	291

Identified appropriate diarrhea treatment methods[d]	Knowledge and attitudes	All respondents with a complete survey	0·097 (0·040 to 0·15)	0·0008	9916
Identified zinc 10-14 days as diarrhea treatment	Knowledge and attitudes	All respondents with a complete survey	0·030 (0·0077 to 0·052)	0·0081	9917
Identified zinc supplement as way to prevent diarrhea	Knowledge and attitudes	All respondents with a complete survey	0·061 (-0·0089 to 0·13)	0·087	9917
Identified breastfeeding as way to prevent diarrhea	Knowledge and attitudes	All respondents with a complete survey	0·036 (-0·032 to 0·10)	0·30	9917
Correctly answered diarrhea treatment with zinc riddle	Intervention knowledge	All respondents with a complete survey	0·23 (0·16 to 0·29)	<0·0001	9916
**Respiratory illness**					
Did not report respiratory illness (coughing) for 2 weeks	Practice	All respondents with a complete survey	-0·015 (-0·049 to 0·019)	0·39	9918
Child did not have cough past 4 weeks	Practice	All respondents with a complete survey	-0·067 (-0·13 to -0·0079)	0·027	3150
Child had cough past 4 weeks, care was sought	Practice	All respondents with a complete survey and a new child <= 5 years old reported at wave 3 who had an illness with a cough in past 4 weeks	0·035 (-0·091 to 0·16)	0·58	661
Identified vaccination as way to prevent respiratory illness	Knowledge and attitudes	All respondents with a complete survey	-0·022 (-0·060 to 0·017)	0·27	9917
Identified breastfeeding as way to prevent respiratory illness	Knowledge and attitudes	All respondents with a complete survey	0·013 (-0·042 to 0·068)	0·65	9917
Identified fever as d.s. for children with respiratory illness	Knowledge and attitudes	All respondents with a complete survey	0·0052 (-0·027 to 0·037)	0·75	9917
Identified difficulty breathing as d.s. for children with respiratory illness	Knowledge and attitudes	All respondents with a complete survey	0·0048 (-0·014 to 0·023)	0·61	9917
Identified rapid breathing as d.s. for children with respiratory illness	Knowledge and attitudes	All respondents with a complete survey	0·0050 (-0·0070 to 0·017)	0·41	9917
**Reproductive health**					
Reported ever using birth control to delay or avoid pregnancy	Practice	All respondents with a complete survey	-0·022 (-0·081 to 0·036)	0·46	9913
Reported currently using birth control	Practice	All respondents with a complete survey who are not pregnant and have ever used birth control	0·047 (-0·028 to 0·12)	0·22	3604
Believes woman should be at least 18 years of age to have her first child	Knowledge and attitudes	All respondents with a complete survey	0·011 (0·0023 to 0·020)	0·014	9407
**Gender/reproductive norms**					

Birth location chosen either jointly or by woman	Practice	All respondents with a complete survey and a child born since 2018-09-01 (after the end of the intervention)	0·048 (-0·054 to 0·15)	0·36	568

Results based on sub-sample of respondents who didn't move villages during the study N=9918 (household targeted n=3129, household not targeted n=6789). d.s.=Danger sign, ORT=Oral rehydration therapy. Standard errors are clustered at the village level. Robust 95% CIs in parentheses.

[a] 'Identified correct ways to provide newborn care' outcome was coded as correct if the respondent identified holding baby skin to skin, getting their health checked by a professional, immediately putting them to the breast, or making sure their diaper is clean and dry, and did not identify giving purgante, wrapping a fajero, giving a chupon, or bathing them in water right away.
[b] 'Identified proper cord care' outcome was coded as correct if respondent identified applying alcohol, applying iodine, keeping cord clean/dry, or applying peroxide, and did not identify applying oil, applying coffee, applying quina water, wrapping a fajero, applying powder, burning, applying ashes, applying a clamp, or applying thiomersal.
[c] 'Used appropriate treatment for diarrhea in past 4 weeks' outcome was coded as correct if respondent did any of the following: antibiotic, zinc, give extra food, give extra liquid, and did not do any of the following: anti-diarrhea medication, home remedy, laxative, deworming medication, chupon, massage, stop eating foods, stop taking liquids.
[d] 'Identified appropriate diarrhea treatment methods' outcome was coded as correct if respondent identified any of the following: antibiotic, zinc, ORS or Litrosol, give extra food, give extra liquid, get medical attention, and did not identify any of the following: anti-diarrhea medication, laxative, deworming medication, home remedy, chupon, massage, stop giving foods, stop giving liquids, or do nothing.

Oles W, *et al. BMJ Open* 2024; 14:e060784. doi: 10.1136/bmjopen-2022-060784

Table S4. Pooled total effect estimates of intervention on maternal, child, and neonatal outcomes, with adjustment for baseline demographic characteristics

	Outcome type	Respondent denominator	Pooled total effect estimate		N
			Effect size (95% CI)	p value	
**Prenatal care**					
Currently taking folic acid tablets (daily in past 7 days)	Practice	All women 15+ with a complete survey	-0·0059 (-0·049 to 0·037)	0·79	5922
Made a birth plan in preparation for birth	Practice	All respondents with a complete survey and a child born since 2018-09-01 (after the end of the intervention)	0·026 (-0·080 to 0·13)	0·63	536
Saved money in preparation for birth	Practice	All respondents with a complete survey and a child born since 2018-09-01 (after the end of the intervention)	-0·090 (-0·22 to 0·039)	0·17	540
Sold animal in preparation for birth	Practice	All respondents with a complete survey and a child born since 2018-09-01 (after the end of the intervention)	-0·013 (-0·078 to 0·051)	0·69	535
Sought prenatal care within 12 weeks	Practice	All respondents with a complete survey and a child born since 2018-09-01 (after the end of the intervention)	-0·0044 (-0·13 to 0·12)	0·94	518
Identifies that women should take folic acid before pregnancy	Knowledge and attitudes	All respondents with a complete survey	0·060 (0·028 to 0·093)	0·0003	9197
Identifies that women should seek prenatal care first 12 weeks of pregnancy	Knowledge and attitudes	All respondents with a complete survey	0·032 (0·011 to 0·053)	0·0033	9196
Identifies accompanying woman to prenatal care visits as method of support during pregnancy	Knowledge and attitudes	All respondents with a complete survey	0·0020 (-0·048 to 0·052)	0·94	9192
Identifies ensuring that woman eats well as method of support during pregnancy	Knowledge and attitudes	All respondents with a complete survey	-0·027 (-0·077 to 0·023)	0·29	9192
Identifies ensuring that woman rests 1 hour per day well as method of support during pregnancy	Knowledge and attitudes	All respondents with a complete survey	0·0095 (-0·022 to 0·041)	0·56	9183
Identifies avoiding violence as method of support during pregnancy	Knowledge and attitudes	All respondents with a complete survey	-0·029 (-0·051 to -0·0079)	0·0073	9192
Identifies encouraging women to take vitamins as method of support during pregnancy	Knowledge and attitudes	All respondents with a complete survey	-0·019 (-0·054 to 0·017)	0·30	9192
Identifies helping woman with house work/child care as method of support during pregnancy	Knowledge and attitudes	All respondents with a complete survey	0·085 (0·030 to 0·14)	0·0022	9192
Identifies saving animals to sell as method of preparing for birth expenses	Knowledge and attitudes	All respondents with a complete survey	0·023 (-0·0023 to 0·048)	0·075	9187

Identifies knowing cost of trip to maternal clinic as method of preparing for birth expenses	Knowledge and attitudes	All respondents with a complete survey	-0·0044 (-0·035 to 0·026)	0·78	9196
Identifies having a savings plan as method of preparing for birth expenses	Knowledge and attitudes	All respondents with a complete survey	0·039 (0·012 to 0·067)	0·0047	9172
Correctly answered prenatal care knowledge riddle	Intervention knowledge	All respondents with a complete survey	0·090 (0·054 to 0·13)	<0·0001	9196
**Facility based birth**					
Gave birth in health facility	Practice	All respondents with a complete survey and a child born since 2018-09-01 (after the end of the intervention)	0·026 (-0·088 to 0·14)	0·66	540
Believes health facility is best place to give birth	Knowledge and attitudes	All respondents with a complete survey	0·024 (-0·00060 to 0·049)	0·056	9188
**Pregnancy danger signs**					
Woman sought medical care for pregnancy danger sign	Practice	All respondents with a complete survey and a child born since 2018-09-01 (after the end of the intervention) who experienced a prenatal care danger sign	-0·040 (-0·25 to 0·17)	0·71	209
Identified bleeding as pregnancy d.s.	Knowledge and attitudes	All respondents with a complete survey	0·051 (0·013 to 0·090)	0·0090	9195
Identified seizure as pregnancy d.s.	Knowledge and attitudes	All respondents with a complete survey	0·0020 (-0·0076 to 0·012)	0·68	9186
Identified headache as pregnancy d.s.	Knowledge and attitudes	All respondents with a complete survey	0·036 (-0·010 to 0·083)	0·13	9195
Identified ringing in ears as pregnancy d.s.	Knowledge and attitudes	All respondents with a complete survey	0·0031 (-0·0064 to 0·013)	0·52	9171
Identified dizziness as pregnancy d.s.	Knowledge and attitudes	All respondents with a complete survey	-0·0026 (-0·049 to 0·044)	0·91	9195
Identified difficulty urinating as pregnancy d.s.	Knowledge and attitudes	All respondents with a complete survey	-0·0018 (-0·0094 to 0·0059)	0·65	9171
Identified reduced or absent fetal movement as pregnancy d.s.	Knowledge and attitudes	All respondents with a complete survey	0·014 (-0·0060 to 0·034)	0·17	9171
Identified water breaking as pregnancy d.s.	Knowledge and attitudes	All respondents with a complete survey	-0·0007 (-0·0079 to 0·0066)	0·86	9171
Identified fever as pregnancy d.s.	Knowledge and attitudes	All respondents with a complete survey	0·040 (0·0012 to 0·079)	0·043	9195
Identified swelling of face/hands/feet as pregnancy d.s.	Knowledge and attitudes	All respondents with a complete survey	0·034 (0·0050 to 0·064)	0·022	9195
**Postnatal care for mother**					
Mother had health checked by professional within 3 days of birth	Practice	All respondents with a complete survey and a child born since 2018-09-01 (after the end of the intervention)	0·13 (-0·024 to 0·29)	0·096	530

Oles W, *et al. BMJ Open* 2024; 14:e060784. doi: 10.1136/bmjopen-2022-060784

Mother had health checked by professional within 7 days of birth	Practice	All respondents with a complete survey and a child born since 2018-09-01 (after the end of the intervention)	0·075 (-0·065 to 0·22)	0·30	530
Mother sought medical care for postnatal danger sign	Practice	All respondents with a complete survey and a child born since 2018-09-01 (after the end of the intervention)	0·12 (-0·072 to 0·31)	0·23	178
Identifies that mother should receive postnatal medical check-up within 3 days of birth	Knowledge and attitudes	All respondents with a complete survey	0·073 (0·031 to 0·11)	0·0006	9196
Identifies that mother should receive postnatal medical check-up within 7 days of birth	Knowledge and attitudes	All respondents with a complete survey	0·040 (-0·0085 to 0·089)	0·11	9196
Identified heavy vaginal bleeding as postnatal d.s.	Knowledge and attitudes	All respondents with a complete survey	0·067 (0·029 to 0·11)	0·0006	9195
Identified fever as postnatal d.s.	Knowledge and attitudes	All respondents with a complete survey	0·060 (0·0098 to 0·11)	0·019	9195
Identified vaginal discharge as postnatal d.s.	Knowledge and attitudes	All respondents with a complete survey	0·0046 (-0·010 to 0·019)	0·54	9180
Identified headache as postnatal d.s.	Knowledge and attitudes	All respondents with a complete survey	0·029 (-0·0086 to 0·066)	0·13	9195
Identified convulsions or fits as postnatal d.s.	Knowledge and attitudes	All respondents with a complete survey	0·00 (-0·0056 to 0·0056)	1·00	8983
**Postnatal care for newborn**					
Newborn had health checked by professional within 3 days of birth	Practice	All respondents with a complete survey and a child born since 2018-09-01 (after the end of the intervention)	0·16 (0·029 to 0·30)	0·017	532
Newborn had health checked by professional within 7 days of birth	Practice	All respondents with a complete survey and a child born since 2018-09-01 (after the end of the intervention)	0·085 (-0·041 to 0·21)	0·19	532
Newborn experienced health problem in first month, care was sought	Practice	All respondents with a complete survey and a child born since 2018-09-01 (after the end of the intervention)	0·0030 (-0·23 to 0·23)	0·98	154
Identified correct ways to provide newborn care[a]	Knowledge and attitudes	All respondents with a complete survey	0·024 (0·010 to 0·037)	0·0005	9172
Identified fever as newborn d.s.	Knowledge and attitudes	All respondents with a complete survey	0·0022 (-0·045 to 0·049)	0·93	9195
Identified diarrhea as newborn d.s.	Knowledge and attitudes	All respondents with a complete survey	0·0035 (-0·037 to 0·044)	0·86	9195
Identified difficulty breathing as newborn d.s.	Knowledge and attitudes	All respondents with a complete survey	0·0099 (-0·025 to 0·045)	0·58	9171
Identified vomiting as newborn d.s.	Knowledge and attitudes	All respondents with a complete survey	0·0090 (-0·021 to 0·039)	0·56	9186
Identified poor appetite as newborn d.s.	Knowledge and attitudes	All respondents with a complete survey	-0·0049 (-0·015 to 0·0047)	0·32	9171
Identified redness/bleeding around cord as newborn d.s.	Knowledge and attitudes	All respondents with a complete survey	0·020 (-0·0017 to 0·041)	0·072	9195
Identified pus in cord as newborn d.s.	Knowledge and attitudes	All respondents with a complete survey	0·015 (-0·0014 to 0·031)	0·074	9186
Identified cold skin as newborn d.s.	Knowledge and attitudes	All respondents with a complete survey	0·0023 (-0·0068 to 0·012)	0·61	9171

Oles W, *et al. BMJ Open* 2024; 14:e060784. doi: 10.1136/bmjopen-2022-060784

Identified cough as newborn d.s.	Knowledge and attitudes	All respondents with a complete survey	-0·019 (-0·069 to 0·031)	0·45	9195
Identified cold as newborn d.s.	Knowledge and attitudes	All respondents with a complete survey	-0·017 (-0·067 to 0·034)	0·52	9195
Identified problems latching as newborn d.s.	Knowledge and attitudes	All respondents with a complete survey	-0·0013 (-0·015 to 0·012)	0·85	9171
Identified pneumonia as newborn d.s.	Knowledge and attitudes	All respondents with a complete survey	0·0061 (-0·030 to 0·042)	0·74	9171
Did not use harmful substances to treat cord stump	Practice	All respondents with a complete survey and a child born since 2018-09-01 (after the end of the intervention)	0·089 (-0·042 to 0·22)	0·18	527
Did not wrap fajero around newborn in first 7 days after birth	Practice	All respondents with a complete survey and a child born since 2018-09-01 (after the end of the intervention)	0·27 (0·11 to 0·42)	0·0006	535
Identified proper cord care methods[b]	Knowledge and attitudes	All respondents with a complete survey	0·095 (0·046 to 0·14)	0·0001	9195
Correctly answered proper cord care riddle	Intervention knowledge	All respondents with a complete survey	0·21 (0·16 to 0·25)	<0·0001	9197
Kept newborn wrapped first 7 days after birth	Practice	All respondents with a complete survey and a child born since 2018-09-01 (after the end of the intervention)	-0·050 (-0·11 to 0·012)	0·12	530
Held newborn skin-to-skin during first month after birth	Practice	All respondents with a complete survey and a child born since 2018-09-01 (after the end of the intervention)	0·11 (-0·019 to 0·24)	0·095	537
**Breastfeeding**					
Exclusively breastfed child first 6 months	Practice	All respondents with a complete survey and a child born since 2018-09-01 (after the end of the intervention)	-0·0046 (-0·14 to 0·14)	0·95	539
Exclusively breastfed child first 6 months without giving chupón	Practice	All respondents with a complete survey and a child born since 2018-09-01 (after the end of the intervention)	0·061 (-0·082 to 0·20)	0·40	539
Breastfed immediately after birth	Practice	All respondents with a complete survey and a child born since 2018-09-01 (after the end of the intervention) who reported a natural birth	0·090 (-0·0078 to 0·19)	0·071	428
Identifies that newborn should be breastfed immediately after birth	Knowledge and attitudes	All respondents with a complete survey	0·016 (-0·018 to 0·050)	0·35	9196
Identifies that newborns should only be given breast milk during first 6 months	Knowledge and attitudes	All respondents with a complete survey	0·039 (0·0073 to 0·070)	0·015	9196
Believes newborns should not be given chupón during first 6 months	Knowledge and attitudes	All respondents with a complete survey	0·16 (0·10 to 0·21)	<0·0001	9196
**Paternal involvement**					
Father accompanied mother to clinic for prenatal care visit at least once	Practice	All respondents with a complete survey and a child born since 2018-09-01 (after the end of the intervention) who received prenatal care	-0·016 (-0·20 to 0·17)	0·87	519
Father accompanied mother to seek medical care for pregnancy danger sign	Practice	All respondents with a complete survey and a child born since 2018-09-01 (after the end of the intervention) who experienced a prenatal care danger sign and sought care	0·16 (-0·081 to 0·41)	0·19	134

Oles W, *et al. BMJ Open* 2024; 14:e060784. doi: 10.1136/bmjopen-2022-060784

Father waited at birthplace during labor	Practice	All respondents with a complete survey and a child born since 2018-09-01 (after the end of the intervention)	-0·022 (-0·15 to 0·11)	0·74	540
Father accompanied mother to seek medical care for postnatal danger sign	Practice	All respondents with a complete survey and a child born since 2018-09-01 (after the end of the intervention) and sought care for postpartum problem	0·099 (-0·16 to 0·36)	0·46	93
Father sought medical care for newborn for postnatal danger sign	Practice	All respondents with a complete survey and a child born since 2018-09-01 (after the end of the intervention) and sought care for a postnatal problem	-0·010 (-0·30 to 0·28)	0·95	98
Father held child	Practice	All respondents with a complete survey and a child born since 2018-09-01 (after the end of the intervention)	0·045 (-0·028 to 0·12)	0·23	535
Father played with child	Practice	All respondents with a complete survey and a child born since 2018-09-01 (after the end of the intervention)	0·013 (-0·10 to 0·13)	0·83	535
Father took child to clinic when sick	Practice	All respondents with a complete survey and a child born since 2018-09-01 (after the end of the intervention)	0·091 (-0·040 to 0·22)	0·17	535
Believes father should accompany mother to prenatal care visits	Knowledge and attitudes	All respondents with a complete survey	0·038 (0·015 to 0·061)	0·0014	9158
Believes father should wait at birth location while mother gives birth	Knowledge and attitudes	All respondents with a complete survey	0·014 (-0·0044 to 0·032)	0·14	9156
Believes father should care for children when sick	Knowledge and attitudes	All respondents with a complete survey	0·0001 (-0·0059 to 0·0062)	0·97	9177
**Diarrhea management**					
Did not report diarrhea in past 4 weeks	Practice	All respondents with a complete survey	-0·0095 (-0·025 to 0·0063)	0·24	9182
Used ORT to treat diarrhea in past 4 weeks	Practice	All respondents with a complete survey who had diarrhea in past 4 weeks	-0·17 (-0·35 to 0·0073)	0·060	241
Used appropriate treatment for diarrhea in past 4 weeks[c]	Practice	All respondents with a complete survey who had diarrhea in past 4 weeks	-0·0029 (-0·14 to 0·14)	0·97	243
Child did not experience diarrhea past 4 weeks	Practice	All respondents with a complete survey and a new child <= 5 years old reported at wave 3 who had diarrhea in past 4 weeks	-0·0049 (-0·042 to 0·032)	0·80	3033
Child experienced diarrhea past 4 weeks, was given appropriate amount of liquids	Practice	All respondents with a complete survey and a new child <= 5 years old reported at wave 3 who had diarrhea in past 4 weeks	-0·16 (-0·32 to 0·0046)	0·057	310
Child experienced diarrhea past 4 weeks, was given appropriate amount of food	Practice	All respondents with a complete survey and a new child <= 5 years old reported at wave 3 who had diarrhea in past 4 weeks	-0·14 (-0·27 to -0·0099)	0·035	313
Child experienced diarrhea past 4 weeks, was given ORT	Practice	All respondents with a complete survey and a new child <= 5 years old reported at wave 3 who had diarrhea in past 4 weeks	0·14 (-0·035 to 0·32)	0·12	310
Child experienced diarrhea past 4 weeks, was given appropriate treatment	Practice	All respondents with a complete survey and a new child <= 5 years old reported at wave 3 who had diarrhea in past 4 weeks	0·13 (-0·038 to 0·29)	0·13	312
Child experienced diarrhea past 4 weeks, was given zinc 10-14 days	Practice	All respondents with a complete survey and a new child <= 5 years old reported at wave 3 who had diarrhea in past 4 weeks	..	..	..

Identified appropriate diarrhea treatment methods[d]	Knowledge and attitudes	All respondents with a complete survey	0·11 (0·056 to 0·16)	<0·0001	9195
Identified zinc 10-14 days as diarrhea treatment	Knowledge and attitudes	All respondents with a complete survey	0·033 (0·0097 to 0·056)	0·0053	9181
Identified zinc supplement as way to prevent diarrhea	Knowledge and attitudes	All respondents with a complete survey	0·073 (0·0073 to 0·14)	0·029	9196
Identified breastfeeding as way to prevent diarrhea	Knowledge and attitudes	All respondents with a complete survey	0·040 (-0·021 to 0·100)	0·20	9196
Correctly answered diarrhea treatment with zinc riddle	Intervention knowledge	All respondents with a complete survey	0·24 (0·19 to 0·30)	<0·0001	9197
**Respiratory illness**					
Did not report respiratory illness (coughing) for 2 weeks	Practice	All respondents with a complete survey	-0·013 (-0·046 to 0·020)	0·44	9188
Child did not have cough past 4 weeks	Practice	All respondents with a complete survey	-0·060 (-0·12 to -0·0046)	0·034	3034
Child had cough past 4 weeks, care was sought	Practice	All respondents with a complete survey and a new child <= 5 years old reported at wave 3 who had an illness with a cough in past 4 weeks	0·042 (-0·085 to 0·17)	0·52	647
Identified vaccination as way to prevent respiratory illness	Knowledge and attitudes	All respondents with a complete survey	-0·0017 (-0·039 to 0·036)	0·93	9196
Identified breastfeeding as way to prevent respiratory illness	Knowledge and attitudes	All respondents with a complete survey	0·030 (-0·021 to 0·081)	0·25	9196
Identified fever as d.s. for children with respiratory illness	Knowledge and attitudes	All respondents with a complete survey	0·015 (-0·012 to 0·043)	0·27	9187
Identified difficulty breathing as d.s. for children with respiratory illness	Knowledge and attitudes	All respondents with a complete survey	0·011 (-0·0049 to 0·027)	0·18	9187
Identified rapid breathing as d.s. for children with respiratory illness	Knowledge and attitudes	All respondents with a complete survey	0·0082 (-0·0024 to 0·019)	0·13	9187
**Reproductive health**					
Reported ever using birth control to delay or avoid pregnancy	Practice	All respondents with a complete survey	-0·0030 (-0·055 to 0·049)	0·91	9191
Reported currently using birth control	Practice	All respondents with a complete survey who are not pregnant and have ever used birth control	0·035 (-0·027 to 0·097)	0·27	3420
Believes woman should be at least 18 years of age to have her first child	Knowledge and attitudes	All respondents with a complete survey	0·012 (0·0048 to 0·019)	0·0011	8713
**Gender/reproductive norms**					
Birth location chosen either jointly or by woman	Practice	All respondents with a complete survey and a child born since 2018-09-01 (after the end of the intervention)	0·049 (-0·040 to 0·14)	0·28	540

Oles W, *et al. BMJ Open* 2024; 14:e060784. doi: 10.1136/bmjopen-2022-060784

Results based on sub-sample that completed baseline survey, N=9285 (household targeted n=2919, household not targeted n=6366). Baseline covariates included age, gender, education, marital status, indigenous status, and household wealth index. d.s.=Danger sign, ORT=Oral rehydration therapy. ··Model did not converge. Covariates were dropped sequentially if they predicted outcome perfectly. Standard errors are clustered at the village level. Robust 95% CIs in parentheses.

[a] 'Identified correct ways to provide newborn care' outcome was coded as correct if the respondent identified holding baby skin to skin, getting their health checked by a professional, immediately putting them to the breast, or making sure their diaper is clean and dry, and did not identify giving purgante, wrapping a fajero, giving a chupon, or bathing them in water right away.
[b] 'Identified proper cord care' outcome was coded as correct if respondent identified applying alcohol, applying iodine, keeping cord clean/dry, or applying peroxide, and did not identify applying oil, applying coffee, applying quina water, wrapping a fajero, applying powder, burning, applying ashes, applying a clamp, or applying thiomersal.
[c] 'Used appropriate treatment for diarrhea in past 4 weeks' outcome was coded as correct if respondent did any of the following: antibiotic, zinc, give extra food, give extra liquid, and did not do any of the following: anti-diarrhea medication, home remedy, laxative, deworming medication, chupon, massage, stop eating foods, stop taking liquids.
[d] 'Identified appropriate diarrhea treatment methods' outcome was coded as correct if respondent identified any of the following: antibiotic, zinc, ORS or Litrosol, give extra food, give extra liquid, get medical attention, and did not identify any of the following: anti-diarrhea medication, laxative, deworming medication, home remedy, chupon, massage, stop giving foods, stop giving liquids, or do nothing.

Table S5. Pooled total effect estimates of intervention on maternal, child, and neonatal outcomes, with adjustment for baseline outcome

	Outcome type	Respondent denominator	Pooled total effect estimate		N
			Effect size (95% CI)	p value	
**Prenatal care**					
Currently taking folic acid tablets (daily in past 7 days)	Practice	All women 15+ with a complete survey	0·0086 (-0·033 to 0·050)	0·69	5552
Made a birth plan in preparation for birth	Practice	All respondents with a complete survey and a child born since 2018-09-01 (after the end of the intervention)	-0·075 (-0·22 to 0·074)	0·32	299
Saved money in preparation for birth	Practice	All respondents with a complete survey and a child born since 2018-09-01 (after the end of the intervention)	0·044 (-0·092 to 0·18)	0·53	299
Sold animal in preparation for birth	Practice	All respondents with a complete survey and a child born since 2018-09-01 (after the end of the intervention)	NA	NA	NA
Sought prenatal care within 12 weeks	Practice	All respondents with a complete survey and a child born since 2018-09-01 (after the end of the intervention)	0·0028 (-0·16 to 0·16)	0·97	285
Identifies that women should take folic acid before pregnancy	Knowledge and attitudes	All respondents with a complete survey	0·045 (0·0062 to 0·083)	0·023	9283
Identifies that women should seek prenatal care first 12 weeks of pregnancy	Knowledge and attitudes	All respondents with a complete survey	0·024 (-0·0017 to 0·050)	0·067	9282
Identifies accompanying woman to prenatal care visits as method of support during pregnancy	Knowledge and attitudes	All respondents with a complete survey	NA	NA	NA
Identifies ensuring that woman eats well as method of support during pregnancy	Knowledge and attitudes	All respondents with a complete survey	NA	NA	NA
Identifies ensuring that woman rests 1 hour per day well as method of support during pregnancy	Knowledge and attitudes	All respondents with a complete survey	NA	NA	NA
Identifies avoiding violence as method of support during pregnancy	Knowledge and attitudes	All respondents with a complete survey	NA	NA	NA
Identifies encouraging women to take vitamins as method of support during pregnancy	Knowledge and attitudes	All respondents with a complete survey	NA	NA	NA
Identifies helping woman with house work/child care as method of support during pregnancy	Knowledge and attitudes	All respondents with a complete survey	NA	NA	NA
Identifies saving animals to sell as method of preparing for birth expenses	Knowledge and attitudes	All respondents with a complete survey	NA	NA	NA

Identifies knowing cost of trip to maternal clinic as method of preparing for birth expenses	Knowledge and attitudes	All respondents with a complete survey	NA	NA	NA
Identifies having a savings plan as method of preparing for birth expenses	Knowledge and attitudes	All respondents with a complete survey	NA	NA	NA
Correctly answered prenatal care knowledge riddle	Intervention knowledge	All respondents with a complete survey	NA	NA	NA
**Facility based birth**					
Gave birth in health facility	Practice	All respondents with a complete survey and a child born since 2018-09-01 (after the end of the intervention)	-0·055 (-0·20 to 0·092)	0·46	299
Believes health facility is best place to give birth	Knowledge and attitudes	All respondents with a complete survey	0·0065 (-0·023 to 0·036)	0·66	9283
**Pregnancy danger signs**					
Woman sought medical care for pregnancy danger sign	Practice	All respondents with a complete survey and a child born since 2018-09-01 (after the end of the intervention) who experienced a prenatal care danger sign	-0·34 (-0·81 to 0·13)	0·15	57
Identified bleeding as pregnancy d.s.	Knowledge and attitudes	All respondents with a complete survey	0·048 (0·0048 to 0·091)	0·030	9279
Identified seizure as pregnancy d.s.	Knowledge and attitudes	All respondents with a complete survey	0·0026 (-0·0071 to 0·012)	0·60	9279
Identified headache as pregnancy d.s.	Knowledge and attitudes	All respondents with a complete survey	0·045 (-0·0051 to 0·094)	0·079	9279
Identified ringing in ears as pregnancy d.s.	Knowledge and attitudes	All respondents with a complete survey	0·0050 (-0·0062 to 0·016)	0·38	9279
Identified dizziness as pregnancy d.s.	Knowledge and attitudes	All respondents with a complete survey	-0·0068 (-0·056 to 0·043)	0·79	9279
Identified difficulty urinating as pregnancy d.s.	Knowledge and attitudes	All respondents with a complete survey	-0·0001 (-0·0085 to 0·0084)	0·99	9279
Identified reduced or absent fetal movement as pregnancy d.s.	Knowledge and attitudes	All respondents with a complete survey	0·014 (-0·0055 to 0·034)	0·16	9279
Identified water breaking as pregnancy d.s.	Knowledge and attitudes	All respondents with a complete survey	-0·0006 (-0·0083 to 0·0071)	0·89	9279
Identified fever as pregnancy d.s.	Knowledge and attitudes	All respondents with a complete survey	0·047 (0·010 to 0·084)	0·013	9279
Identified swelling of face/hands/feet as pregnancy d.s.	Knowledge and attitudes	All respondents with a complete survey	0·037 (0·0039 to 0·070)	0·028	9279
**Postnatal care for mother**					
Mother had health checked by professional within 3 days of birth	Practice	All respondents with a complete survey and a child born since 2018-09-01 (after the end of the intervention)	0·024 (-0·18 to 0·23)	0·82	293

Mother had health checked by professional within 7 days of birth	Practice	All respondents with a complete survey and a child born since 2018-09-01 (after the end of the intervention)	0·0051 (-0·22 to 0·24)	0·97	293
Mother sought medical care for postnatal danger sign	Practice	All respondents with a complete survey and a child born since 2018-09-01 (after the end of the intervention)	0·21 (-0·15 to 0·57)	0·25	34
Identifies that mother should receive postnatal medical check-up within 3 days of birth	Knowledge and attitudes	All respondents with a complete survey	0·092 (0·045 to 0·14)	0·0001	9279
Identifies that mother should receive postnatal medical check-up within 7 days of birth	Knowledge and attitudes	All respondents with a complete survey	0·054 (0·0031 to 0·11)	0·038	9279
Identified heavy vaginal bleeding as postnatal d.s.	Knowledge and attitudes	All respondents with a complete survey	0·060 (0·021 to 0·098)	0·0024	9279
Identified fever as postnatal d.s.	Knowledge and attitudes	All respondents with a complete survey	0·059 (0·0096 to 0·11)	0·019	9279
Identified vaginal discharge as postnatal d.s.	Knowledge and attitudes	All respondents with a complete survey	0·0032 (-0·012 to 0·018)	0·67	9279
Identified headache as postnatal d.s.	Knowledge and attitudes	All respondents with a complete survey	0·031 (-0·0088 to 0·071)	0·13	9279
Identified convulsions or fits as postnatal d.s.	Knowledge and attitudes	All respondents with a complete survey	0·0003 (-0·0055 to 0·0062)	0·91	9279
**Postnatal care for newborn**					
Newborn had health checked by professional within 3 days of birth	Practice	All respondents with a complete survey and a child born since 2018-09-01 (after the end of the intervention)	0·091 (-0·095 to 0·28)	0·34	297
Newborn had health checked by professional within 7 days of birth	Practice	All respondents with a complete survey and a child born since 2018-09-01 (after the end of the intervention)	0·043 (-0·18 to 0·27)	0·71	297
Newborn experienced health problem in first month, care was sought	Practice	All respondents with a complete survey and a child born since 2018-09-01 (after the end of the intervention)	-0·031 (-0·57 to 0·51)	0·91	25
Identified correct ways to provide newborn care[a]	Knowledge and attitudes	All respondents with a complete survey	0·026 (0·010 to 0·042)	0·0011	9282
Identified fever as newborn d.s.	Knowledge and attitudes	All respondents with a complete survey	0·015 (-0·033 to 0·062)	0·54	9281
Identified diarrhea as newborn d.s.	Knowledge and attitudes	All respondents with a complete survey	0·020 (-0·023 to 0·062)	0·36	9281
Identified difficulty breathing as newborn d.s.	Knowledge and attitudes	All respondents with a complete survey	0·011 (-0·026 to 0·049)	0·55	9281
Identified vomiting as newborn d.s.	Knowledge and attitudes	All respondents with a complete survey	0·013 (-0·017 to 0·042)	0·39	9281
Identified poor appetite as newborn d.s.	Knowledge and attitudes	All respondents with a complete survey	-0·0039 (-0·014 to 0·0067)	0·47	9281
Identified redness/bleeding around cord as newborn d.s.	Knowledge and attitudes	All respondents with a complete survey	0·017 (-0·0038 to 0·039)	0·11	9281
Identified pus in cord as newborn d.s.	Knowledge and attitudes	All respondents with a complete survey	0·015 (-0·0014 to 0·031)	0·074	9281
Identified cold skin as newborn d.s.	Knowledge and attitudes	All respondents with a complete survey	0·0018 (-0·0076 to 0·011)	0·70	9281

Identified cough as newborn d.s.	Knowledge and attitudes	All respondents with a complete survey	-0·018 (-0·066 to 0·031)	0·48	9281
Identified cold as newborn d.s.	Knowledge and attitudes	All respondents with a complete survey	-0·025 (-0·080 to 0·030)	0·37	9281
Identified problems latching as newborn d.s.	Knowledge and attitudes	All respondents with a complete survey	-0·0025 (-0·017 to 0·012)	0·72	9279
Identified pneumonia as newborn d.s.	Knowledge and attitudes	All respondents with a complete survey	NA	NA	NA
Did not use harmful substances to treat cord stump	Practice	All respondents with a complete survey and a child born since 2018-09-01 (after the end of the intervention)	0·099 (-0·082 to 0·28)	0·29	289
Did not wrap fajero around newborn in first 7 days after birth	Practice	All respondents with a complete survey and a child born since 2018-09-01 (after the end of the intervention)	0·25 (0·043 to 0·46)	0·018	298
Identified proper cord care methods[b]	Knowledge and attitudes	All respondents with a complete survey	0·094 (0·047 to 0·14)	0·0001	9281
Correctly answered proper cord care riddle	Intervention knowledge	All respondents with a complete survey	NA	NA	NA
Kept newborn wrapped first 7 days after birth	Practice	All respondents with a complete survey and a child born since 2018-09-01 (after the end of the intervention)	-0·041 (-0·12 to 0·038)	0·31	293
Held newborn skin-to-skin during first month after birth	Practice	All respondents with a complete survey and a child born since 2018-09-01 (after the end of the intervention)	0·21 (-0·099 to 0·51)	0·19	72
**Breastfeeding**					
Exclusively breastfed child first 6 months	Practice	All respondents with a complete survey and a child born since 2018-09-01 (after the end of the intervention)	-0·15 (-0·33 to 0·035)	0·11	298
Exclusively breastfed child first 6 months without giving chupón	Practice	All respondents with a complete survey and a child born since 2018-09-01 (after the end of the intervention)	-0·079 (-0·29 to 0·13)	0·46	299
Breastfed immediately after birth	Practice	All respondents with a complete survey and a child born since 2018-09-01 (after the end of the intervention) who reported a natural birth	-0·017 (-0·15 to 0·11)	0·80	230
Identifies that newborn should be breastfed immediately after birth	Knowledge and attitudes	All respondents with a complete survey	0·0088 (-0·027 to 0·044)	0·63	9282
Identifies that newborns should only be given breast milk during first 6 months	Knowledge and attitudes	All respondents with a complete survey	0·038 (0·0086 to 0·068)	0·012	9282
Believes newborns should not be given chupón during first 6 months	Knowledge and attitudes	All respondents with a complete survey	0·14 (0·079 to 0·19)	<0·0001	9282
**Paternal involvement**					
Father accompanied mother to clinic for prenatal care visit at least once	Practice	All respondents with a complete survey and a child born since 2018-09-01 (after the end of the intervention) who received prenatal care	0·16 (-0·049 to 0·36)	0·14	276
Father accompanied mother to seek medical care for pregnancy danger sign	Practice	All respondents with a complete survey and a child born since 2018-09-01 (after the end of the intervention) who experienced a prenatal care danger sign and sought care	-0·017 (-0·71 to 0·67)	0·96	25

Oles W, *et al. BMJ Open* 2024; 14:e060784. doi: 10.1136/bmjopen-2022-060784

Father waited at birthplace during labor	Practice	All respondents with a complete survey and a child born since 2018-09-01 (after the end of the intervention)	-0·013 (-0·17 to 0·14)	0·87	299
Father accompanied mother to seek medical care for postnatal danger sign	Practice	All respondents with a complete survey and a child born since 2018-09-01 (after the end of the intervention) and sought care for postpartum problem	0·28 (-0·061 to 0·63)	0·11	14
Father sought medical care for newborn for postnatal danger sign	Practice	All respondents with a complete survey and a child born since 2018-09-01 (after the end of the intervention) and sought care for a postnatal problem	-0·062 (-0·64 to 0·51)	0·83	16
Father held child	Practice	All respondents with a complete survey and a child born since 2018-09-01 (after the end of the intervention)	-0·0032 (-0·12 to 0·11)	0·96	269
Father played with child	Practice	All respondents with a complete survey and a child born since 2018-09-01 (after the end of the intervention)	0·036 (-0·10 to 0·17)	0·61	275
Father took child to clinic when sick	Practice	All respondents with a complete survey and a child born since 2018-09-01 (after the end of the intervention)	0·068 (-0·11 to 0·25)	0·46	275
Believes father should accompany mother to prenatal care visits	Knowledge and attitudes	All respondents with a complete survey	0·043 (0·020 to 0·066)	0·00020	9214
Believes father should wait at birth location while mother gives birth	Knowledge and attitudes	All respondents with a complete survey	0·014 (-0·0049 to 0·032)	0·15	9225
Believes father should care for children when sick	Knowledge and attitudes	All respondents with a complete survey	-0·0010 (-0·0082 to 0·0061)	0·78	9266
**Diarrhea management**					
Did not report diarrhea in past 4 weeks	Practice	All respondents with a complete survey	-0·0099 (-0·026 to 0·0065)	0·24	9282
Used ORT to treat diarrhea in past 4 weeks	Practice	All respondents with a complete survey who had diarrhea in past 4 weeks	0·21 (-0·28 to 0·70)	0·40	23
Used appropriate treatment for diarrhea in past 4 weeks[c]	Practice	All respondents with a complete survey who had diarrhea in past 4 weeks	-0·24 (-0·48 to 0·0082)	0·058	23
Child did not experience diarrhea past 4 weeks	Practice	All respondents with a complete survey and a new child <= 5 years old reported at wave 3 who had diarrhea in past 4 weeks	-0·011 (-0·053 to 0·031)	0·60	2377
Child experienced diarrhea past 4 weeks, was given appropriate amount of liquids	Practice	All respondents with a complete survey and a new child <= 5 years old reported at wave 3 who had diarrhea in past 4 weeks	1·9 (1·5 to 2·3)	<0·0001	49
Child experienced diarrhea past 4 weeks, was given appropriate amount of food	Practice	All respondents with a complete survey and a new child <= 5 years old reported at wave 3 who had diarrhea in past 4 weeks	1·2 (0·67 to 1·7)	<0·0001	50
Child experienced diarrhea past 4 weeks, was given ORT	Practice	All respondents with a complete survey and a new child <= 5 years old reported at wave 3 who had diarrhea in past 4 weeks	-0·029 (-0·58 to 0·52)	0·92	50
Child experienced diarrhea past 4 weeks, was given appropriate treatment	Practice	All respondents with a complete survey and a new child <= 5 years old reported at wave 3 who had diarrhea in past 4 weeks	1·1 (0·60 to 1·5)	<0·0001	55
Child experienced diarrhea past 4 weeks, was given zinc 10-14 days	Practice	All respondents with a complete survey and a new child <= 5 years old reported at wave 3 who had diarrhea in past 4 weeks	..	..	..

Identified appropriate diarrhea treatment methods[d]	Knowledge and attitudes	All respondents with a complete survey	0·093 (0·038 to 0·15)	0·0009	9280
Identified zinc 10-14 days as diarrhea treatment	Knowledge and attitudes	All respondents with a complete survey	0·034 (0·010 to 0·057)	0·0046	9257
Identified zinc supplement as way to prevent diarrhea	Knowledge and attitudes	All respondents with a complete survey	0·070 (0·0004 to 0·14)	0·049	9282
Identified breastfeeding as way to prevent diarrhea	Knowledge and attitudes	All respondents with a complete survey	0·041 (-0·026 to 0·11)	0·23	9282
Correctly answered diarrhea treatment with zinc riddle	Intervention knowledge	All respondents with a complete survey	NA	NA	NA
**Respiratory illness**					
Did not report respiratory illness (coughing) for 2 weeks	Practice	All respondents with a complete survey	-0·021 (-0·057 to 0·014)	0·24	9283
Child did not have cough past 4 weeks	Practice	All respondents with a complete survey	-0·033 (-0·097 to 0·031)	0·31	2379
Child had cough past 4 weeks, care was sought	Practice	All respondents with a complete survey and a new child <= 5 years old reported at wave 3 who had an illness with a cough in past 4 weeks	0·0043 (-0·24 to 0·24)	0·97	196
Identified vaccination as way to prevent respiratory illness	Knowledge and attitudes	All respondents with a complete survey	-0·0075 (-0·047 to 0·032)	0·71	9282
Identified breastfeeding as way to prevent respiratory illness	Knowledge and attitudes	All respondents with a complete survey	0·027 (-0·028 to 0·081)	0·34	9282
Identified fever as d.s. for children with respiratory illness	Knowledge and attitudes	All respondents with a complete survey	0·0090 (-0·021 to 0·039)	0·56	9282
Identified difficulty breathing as d.s. for children with respiratory illness	Knowledge and attitudes	All respondents with a complete survey	0·0066 (-0·0098 to 0·023)	0·43	9282
Identified rapid breathing as d.s. for children with respiratory illness	Knowledge and attitudes	All respondents with a complete survey	0·0069 (-0·0044 to 0·018)	0·23	9282
**Reproductive health**					
Reported ever using birth control to delay or avoid pregnancy	Practice	All respondents with a complete survey	0·011 (-0·038 to 0·060)	0·67	8529
Reported currently using birth control	Practice	All respondents with a complete survey who are not pregnant and have ever used birth control	0·060 (-0·038 to 0·16)	0·23	1948
Believes woman should be at least 18 years of age to have her first child	Knowledge and attitudes	All respondents with a complete survey	0·012 (0·0029 to 0·020)	0·0087	8675
**Gender/reproductive norms**					
Birth location chosen either jointly or by woman	Practice	All respondents with a complete survey and a child born since 2018-09-01 (after the end of the intervention)	0·042 (-0·10 to 0·19)	0·57	299

Results based on sub-sample that completed baseline survey, N=9285 (household targeted n=2919, household not targeted n=6366). Models included covariate for the related outcome assessed at baseline survey. d.s.=Danger sign, NA=Not applicable, ORT=Oral rehydration therapy. ··Model did not converge. Results with NA could not be assessed because outcome was not included in baseline survey. Standard errors are clustered at the village level. Robust 95% CIs in parentheses.

[a] ‘Identified correct ways to provide newborn care’ outcome was coded as correct if the respondent identified holding baby skin to skin, getting their health checked by a professional, immediately putting them to the breast, or making sure their diaper is clean and dry, and did not identify giving purgante, wrapping a fajero, giving a chupon, or bathing them in water right away.
[b] ‘Identified proper cord care’ outcome was coded as correct if respondent identified applying alcohol, applying iodine, keeping cord clean/dry, or applying peroxide, and did not identify applying oil, applying coffee, applying quina water, wrapping a fajero, applying powder, burning, applying ashes, applying a clamp, or applying thiomersal.
[c] ‘Used appropriate treatment for diarrhea in past 4 weeks’ outcome was coded as correct if respondent did any of the following: antibiotic, zinc, give extra food, give extra liquid, and did not do any of the following: anti-diarrhea medication, home remedy, laxative, deworming medication, chupon, massage, stop eating foods, stop taking liquids.
[d] ‘Identified appropriate diarrhea treatment methods’ outcome was coded as correct if respondent identified any of the following: antibiotic, zinc, ORS or Litrosol, give extra food, give extra liquid, get medical attention, and did not identify any of the following: anti-diarrhea medication, laxative, deworming medication, home remedy, chupon, massage, stop giving foods, stop giving liquids, or do nothing.

Table S6. Pooled total effect estimates of intervention on maternal, child, and neonatal outcomes, with adjustment for baseline demographic characteristics and baseline outcome

	Outcome type	Respondent denominator	Pooled total effect estimate		N
			Effect size (95% CI)	p value	
**Prenatal care**					
Currently taking folic acid tablets (daily in past 7 days)	Practice	All women 15+ with a complete survey	-0·0013 (-0·041 to 0·039)	0·95	5492
Made a birth plan in preparation for birth	Practice	All respondents with a complete survey and a child born since 2018-09-01 (after the end of the intervention)	-0·083 (-0·22 to 0·051)	0·22	291
Saved money in preparation for birth	Practice	All respondents with a complete survey and a child born since 2018-09-01 (after the end of the intervention)	-0·0003 (-0·13 to 0·13)	1·00	295
Sold animal in preparation for birth	Practice	All respondents with a complete survey and a child born since 2018-09-01 (after the end of the intervention)	NA	NA	NA
Sought prenatal care within 12 weeks	Practice	All respondents with a complete survey and a child born since 2018-09-01 (after the end of the intervention)	0·030 (-0·11 to 0·17)	0·67	277
Identifies that women should take folic acid before pregnancy	Knowledge and attitudes	All respondents with a complete survey	0·058 (0·026 to 0·091)	0·0005	9174
Identifies that women should seek prenatal care first 12 weeks of pregnancy	Knowledge and attitudes	All respondents with a complete survey	0·031 (0·0090 to 0·054)	0·0059	9173
Identifies accompanying woman to prenatal care visits as method of support during pregnancy	Knowledge and attitudes	All respondents with a complete survey	NA	NA	NA
Identifies ensuring that woman eats well as method of support during pregnancy	Knowledge and attitudes	All respondents with a complete survey	NA	NA	NA
Identifies ensuring that woman rests 1 hour per day well as method of support during pregnancy	Knowledge and attitudes	All respondents with a complete survey	NA	NA	NA
Identifies avoiding violence as method of support during pregnancy	Knowledge and attitudes	All respondents with a complete survey	NA	NA	NA
Identifies encouraging women to take vitamins as method of support during pregnancy	Knowledge and attitudes	All respondents with a complete survey	NA	NA	NA
Identifies helping woman with house work/child care as method of support during pregnancy	Knowledge and attitudes	All respondents with a complete survey	NA	NA	NA
Identifies saving animals to sell as method of preparing for birth expenses	Knowledge and attitudes	All respondents with a complete survey	NA	NA	NA

Identifies knowing cost of trip to maternal clinic as method of preparing for birth expenses	Knowledge and attitudes	All respondents with a complete survey	NA	NA	NA
Identifies having a savings plan as method of preparing for birth expenses	Knowledge and attitudes	All respondents with a complete survey	NA	NA	NA
Correctly answered prenatal care knowledge riddle	Intervention knowledge	All respondents with a complete survey	NA	NA	NA
**Facility based birth**					
Gave birth in health facility	Practice	All respondents with a complete survey and a child born since 2018-09-01 (after the end of the intervention)	0·0030 (-0·13 to 0·14)	0·97	295
Believes health facility is best place to give birth	Knowledge and attitudes	All respondents with a complete survey	0·018 (-0·0034 to 0·040)	0·099	9165
**Pregnancy danger signs**					
Woman sought medical care for pregnancy danger sign	Practice	All respondents with a complete survey and a child born since 2018-09-01 (after the end of the intervention) who experienced a prenatal care danger sign	-0·26 (-0·76 to 0·24)	0·30	55
Identified bleeding as pregnancy d.s.	Knowledge and attitudes	All respondents with a complete survey	0·051 (0·012 to 0·090)	0·0099	9170
Identified seizure as pregnancy d.s.	Knowledge and attitudes	All respondents with a complete survey	0·0020 (-0·0077 to 0·012)	0·69	9161
Identified headache as pregnancy d.s.	Knowledge and attitudes	All respondents with a complete survey	0·038 (-0·0085 to 0·084)	0·11	9170
Identified ringing in ears as pregnancy d.s.	Knowledge and attitudes	All respondents with a complete survey	0·0031 (-0·0064 to 0·013)	0·52	9146
Identified dizziness as pregnancy d.s.	Knowledge and attitudes	All respondents with a complete survey	-0·0030 (-0·050 to 0·044)	0·90	9170
Identified difficulty urinating as pregnancy d.s.	Knowledge and attitudes	All respondents with a complete survey	-0·0018 (-0·0094 to 0·0059)	0·65	9146
Identified reduced or absent fetal movement as pregnancy d.s.	Knowledge and attitudes	All respondents with a complete survey	0·014 (-0·0060 to 0·034)	0·17	9146
Identified water breaking as pregnancy d.s.	Knowledge and attitudes	All respondents with a complete survey	-0·0006 (-0·0079 to 0·0066)	0·87	9146
Identified fever as pregnancy d.s.	Knowledge and attitudes	All respondents with a complete survey	0·041 (0·0031 to 0·079)	0·034	9170
Identified swelling of face/hands/feet as pregnancy d.s.	Knowledge and attitudes	All respondents with a complete survey	0·032 (0·0030 to 0·061)	0·031	9170
**Postnatal care for mother**					
Mother had health checked by professional within 3 days of birth	Practice	All respondents with a complete survey and a child born since 2018-09-01 (after the end of the intervention)	-0·035 (-0·22 to 0·15)	0·70	289

Mother had health checked by professional within 7 days of birth	Practice	All respondents with a complete survey and a child born since 2018-09-01 (after the end of the intervention)	-0·032 (-0·23 to 0·17)	0·75	289
Mother sought medical care for postnatal danger sign	Practice	All respondents with a complete survey and a child born since 2018-09-01 (after the end of the intervention)	0·38 (0·062 to 0·69)	0·019	33
Identifies that mother should receive postnatal medical check-up within 3 days of birth	Knowledge and attitudes	All respondents with a complete survey	0·072 (0·030 to 0·11)	0·0007	9170
Identifies that mother should receive postnatal medical check-up within 7 days of birth	Knowledge and attitudes	All respondents with a complete survey	0·037 (-0·011 to 0·085)	0·13	9170
Identified heavy vaginal bleeding as postnatal d.s.	Knowledge and attitudes	All respondents with a complete survey	0·066 (0·028 to 0·10)	0·0006	9170
Identified fever as postnatal d.s.	Knowledge and attitudes	All respondents with a complete survey	0·060 (0·011 to 0·11)	0·017	9170
Identified vaginal discharge as postnatal d.s.	Knowledge and attitudes	All respondents with a complete survey	0·0045 (-0·010 to 0·019)	0·55	9155
Identified headache as postnatal d.s.	Knowledge and attitudes	All respondents with a complete survey	0·029 (-0·0085 to 0·066)	0·13	9170
Identified convulsions or fits as postnatal d.s.	Knowledge and attitudes	All respondents with a complete survey	0·00 (-0·0056 to 0·0056)	0·99	8958
**Postnatal care for newborn**					
Newborn had health checked by professional within 3 days of birth	Practice	All respondents with a complete survey and a child born since 2018-09-01 (after the end of the intervention)	0·028 (-0·13 to 0·18)	0·72	293
Newborn had health checked by professional within 7 days of birth	Practice	All respondents with a complete survey and a child born since 2018-09-01 (after the end of the intervention)	0·022 (-0·16 to 0·20)	0·81	293
Newborn experienced health problem in first month, care was sought	Practice	All respondents with a complete survey and a child born since 2018-09-01 (after the end of the intervention)	-0·014 (-0·014 to -0·014)	<0·0001	23
Identified correct ways to provide newborn care[a]	Knowledge and attitudes	All respondents with a complete survey	0·023 (0·0099 to 0·037)	0·0006	9149
Identified fever as newborn d.s.	Knowledge and attitudes	All respondents with a complete survey	0·0033 (-0·043 to 0·049)	0·89	9172
Identified diarrhea as newborn d.s.	Knowledge and attitudes	All respondents with a complete survey	0·0039 (-0·036 to 0·043)	0·85	9172
Identified difficulty breathing as newborn d.s.	Knowledge and attitudes	All respondents with a complete survey	0·0097 (-0·025 to 0·045)	0·59	9148
Identified vomiting as newborn d.s.	Knowledge and attitudes	All respondents with a complete survey	0·0085 (-0·021 to 0·038)	0·58	9163
Identified poor appetite as newborn d.s.	Knowledge and attitudes	All respondents with a complete survey	-0·0049 (-0·015 to 0·0048)	0·32	9148
Identified redness/bleeding around cord as newborn d.s.	Knowledge and attitudes	All respondents with a complete survey	0·020 (-0·0008 to 0·042)	0·059	9172
Identified pus in cord as newborn d.s.	Knowledge and attitudes	All respondents with a complete survey	0·015 (-0·0014 to 0·031)	0·073	9163
Identified cold skin as newborn d.s.	Knowledge and attitudes	All respondents with a complete survey	0·0023 (-0·0069 to 0·011)	0·63	9148

Identified cough as newborn d.s.	Knowledge and attitudes	All respondents with a complete survey	-0·019 (-0·069 to 0·031)	0·45	9172
Identified cold as newborn d.s.	Knowledge and attitudes	All respondents with a complete survey	-0·016 (-0·067 to 0·035)	0·55	9172
Identified problems latching as newborn d.s.	Knowledge and attitudes	All respondents with a complete survey	-0·0013 (-0·015 to 0·012)	0·85	9146
Identified pneumonia as newborn d.s.	Knowledge and attitudes	All respondents with a complete survey	NA	NA	NA
Did not use harmful substances to treat cord stump	Practice	All respondents with a complete survey and a child born since 2018-09-01 (after the end of the intervention)	0·11 (-0·061 to 0·29)	0·20	285
Did not wrap fajero around newborn in first 7 days after birth	Practice	All respondents with a complete survey and a child born since 2018-09-01 (after the end of the intervention)	0·30 (0·11 to 0·48)	0·0018	290
Identified proper cord care methods[b]	Knowledge and attitudes	All respondents with a complete survey	0·094 (0·048 to 0·14)	0·0001	9172
Correctly answered proper cord care riddle	Intervention knowledge	All respondents with a complete survey	NA	NA	NA
Kept newborn wrapped first 7 days after birth	Practice	All respondents with a complete survey and a child born since 2018-09-01 (after the end of the intervention)	..	..	..
Held newborn skin-to-skin during first month after birth	Practice	All respondents with a complete survey and a child born since 2018-09-01 (after the end of the intervention)	0·051 (-0·21 to 0·31)	0·70	70
**Breastfeeding**					
Exclusively breastfed child first 6 months	Practice	All respondents with a complete survey and a child born since 2018-09-01 (after the end of the intervention)	-0·12 (-0·26 to 0·014)	0·079	294
Exclusively breastfed child first 6 months without giving chupón	Practice	All respondents with a complete survey and a child born since 2018-09-01 (after the end of the intervention)	-0·062 (-0·23 to 0·11)	0·47	295
Breastfed immediately after birth	Practice	All respondents with a complete survey and a child born since 2018-09-01 (after the end of the intervention) who reported a natural birth	-0·049 (-0·18 to 0·085)	0·47	217
Identifies that newborn should be breastfed immediately after birth	Knowledge and attitudes	All respondents with a complete survey	0·011 (-0·023 to 0·045)	0·53	9173
Identifies that newborns should only be given breast milk during first 6 months	Knowledge and attitudes	All respondents with a complete survey	0·038 (0·0075 to 0·069)	0·015	9173
Believes newborns should not be given chupón during first 6 months	Knowledge and attitudes	All respondents with a complete survey	0·16 (0·10 to 0·21)	<0·0001	9173
**Paternal involvement**					
Father accompanied mother to clinic for prenatal care visit at least once	Practice	All respondents with a complete survey and a child born since 2018-09-01 (after the end of the intervention) who received prenatal care	0·079 (-0·10 to 0·26)	0·39	272
Father accompanied mother to seek medical care for pregnancy danger sign	Practice	All respondents with a complete survey and a child born since 2018-09-01 (after the end of the intervention) who experienced a prenatal care danger sign and sought care	..	..	..

Oles W, *et al. BMJ Open* 2024; 14:e060784. doi: 10.1136/bmjopen-2022-060784

Father waited at birthplace during labor	Practice	All respondents with a complete survey and a child born since 2018-09-01 (after the end of the intervention)	-0·021 (-0·18 to 0·13)	0·79	295
Father accompanied mother to seek medical care for postnatal danger sign	Practice	All respondents with a complete survey and a child born since 2018-09-01 (after the end of the intervention) and sought care for postpartum problem	..	..	..
Father sought medical care for newborn for postnatal danger sign	Practice	All respondents with a complete survey and a child born since 2018-09-01 (after the end of the intervention) and sought care for a postnatal problem	..	..	..
Father held child	Practice	All respondents with a complete survey and a child born since 2018-09-01 (after the end of the intervention)	0·058 (-0·042 to 0·16)	0·25	265
Father played with child	Practice	All respondents with a complete survey and a child born since 2018-09-01 (after the end of the intervention)	0·077 (-0·037 to 0·19)	0·18	271
Father took child to clinic when sick	Practice	All respondents with a complete survey and a child born since 2018-09-01 (after the end of the intervention)	0·093 (-0·077 to 0·26)	0·28	271
Believes father should accompany mother to prenatal care visits	Knowledge and attitudes	All respondents with a complete survey	0·039 (0·017 to 0·061)	0·0006	9105
Believes father should wait at birth location while mother gives birth	Knowledge and attitudes	All respondents with a complete survey	0·014 (-0·0044 to 0·032)	0·14	9108
Believes father should care for children when sick	Knowledge and attitudes	All respondents with a complete survey	0·0001 (-0·0059 to 0·0062)	0·97	9149
**Diarrhea management**					
Did not report diarrhea in past 4 weeks	Practice	All respondents with a complete survey	-0·0095 (-0·025 to 0·0062)	0·24	9160
Used ORT to treat diarrhea in past 4 weeks	Practice	All respondents with a complete survey who had diarrhea in past 4 weeks	0·036 (0·036 to 0·036)	<0·0001	23
Used appropriate treatment for diarrhea in past 4 weeks[c]	Practice	All respondents with a complete survey who had diarrhea in past 4 weeks	..	..	..
Child did not experience diarrhea past 4 weeks	Practice	All respondents with a complete survey and a new child <= 5 years old reported at wave 3 who had diarrhea in past 4 weeks	-0·0093 (-0·052 to 0·034)	0·67	2346
Child experienced diarrhea past 4 weeks, was given appropriate amount of liquids	Practice	All respondents with a complete survey and a new child <= 5 years old reported at wave 3 who had diarrhea in past 4 weeks	1·9 (1·2 to 2·6)	<0·0001	43
Child experienced diarrhea past 4 weeks, was given appropriate amount of food	Practice	All respondents with a complete survey and a new child <= 5 years old reported at wave 3 who had diarrhea in past 4 weeks	0·95 (0·52 to 1·4)	<0·0001	43
Child experienced diarrhea past 4 weeks, was given ORT	Practice	All respondents with a complete survey and a new child <= 5 years old reported at wave 3 who had diarrhea in past 4 weeks	1·3 (0·73 to 1·8)	<0·0001	43
Child experienced diarrhea past 4 weeks, was given appropriate treatment	Practice	All respondents with a complete survey and a new child <= 5 years old reported at wave 3 who had diarrhea in past 4 weeks	0·59 (0·19 to 0·98)	0·0034	48
Child experienced diarrhea past 4 weeks, was given zinc 10-14 days	Practice	All respondents with a complete survey and a new child <= 5 years old reported at wave 3 who had diarrhea in past 4 weeks	..	..	..

Identified appropriate diarrhea treatment methods[d]	Knowledge and attitudes	All respondents with a complete survey	0·11 (0·055 to 0·16)	0·0001	9171
Identified zinc 10-14 days as diarrhea treatment	Knowledge and attitudes	All respondents with a complete survey	0·032 (0·0095 to 0·055)	0·0056	9133
Identified zinc supplement as way to prevent diarrhea	Knowledge and attitudes	All respondents with a complete survey	0·073 (0·0071 to 0·14)	0·030	9173
Identified breastfeeding as way to prevent diarrhea	Knowledge and attitudes	All respondents with a complete survey	0·039 (-0·021 to 0·100)	0·20	9173
Correctly answered diarrhea treatment with zinc riddle	Intervention knowledge	All respondents with a complete survey	NA	NA	NA
**Respiratory illness**					
Did not report respiratory illness (coughing) for 2 weeks	Practice	All respondents with a complete survey	-0·017 (-0·050 to 0·017)	0·33	9167
Child did not have cough past 4 weeks	Practice	All respondents with a complete survey	-0·038 (-0·10 to 0·026)	0·25	2348
Child had cough past 4 weeks, care was sought	Practice	All respondents with a complete survey and a new child <= 5 years old reported at wave 3 who had an illness with a cough in past 4 weeks	-0·0071 (-0·24 to 0·23)	0·95	195
Identified vaccination as way to prevent respiratory illness	Knowledge and attitudes	All respondents with a complete survey	-0·0012 (-0·039 to 0·036)	0·95	9173
Identified breastfeeding as way to prevent respiratory illness	Knowledge and attitudes	All respondents with a complete survey	0·031 (-0·020 to 0·081)	0·24	9173
Identified fever as d.s. for children with respiratory illness	Knowledge and attitudes	All respondents with a complete survey	0·016 (-0·012 to 0·043)	0·26	9164
Identified difficulty breathing as d.s. for children with respiratory illness	Knowledge and attitudes	All respondents with a complete survey	0·011 (-0·0047 to 0·027)	0·17	9164
Identified rapid breathing as d.s. for children with respiratory illness	Knowledge and attitudes	All respondents with a complete survey	0·0084 (-0·0023 to 0·019)	0·12	9164
**Reproductive health**					
Reported ever using birth control to delay or avoid pregnancy	Practice	All respondents with a complete survey	0·0068 (-0·040 to 0·054)	0·78	8433
Reported currently using birth control	Practice	All respondents with a complete survey who are not pregnant and have ever used birth control	0·051 (-0·039 to 0·14)	0·27	1926
Believes woman should be at least 18 years of age to have her first child	Knowledge and attitudes	All respondents with a complete survey	0·012 (0·0046 to 0·019)	0·0015	8551
**Gender/reproductive norms**					
Birth location chosen either jointly or by woman	Practice	All respondents with a complete survey and a child born since 2018-09-01 (after the end of the intervention)	0·046 (-0·086 to 0·18)	0·49	291

Results based on sub-sample that completed baseline survey, N=9285 (household targeted n=2919, household not targeted n=6366). Covariates included baseline age, gender, education, marital status, indigenous status, household wealth index, and the related outcome. d.s.=Danger sign, NA=Not applicable, ORT=Oral

rehydration therapy. ··Model did not converge. Results with NA could not be assessed because outcome was not included in baseline survey. Covariates were dropped sequentially if they predicted outcome perfectly. Standard errors are clustered at the village level. Robust 95% CIs in parentheses.

[a] 'Identified correct ways to provide newborn care' outcome was coded as correct if the respondent identified holding baby skin to skin, getting their health checked by a professional, immediately putting them to the breast, or making sure their diaper is clean and dry, and did not identify giving purgante, wrapping a fajero, giving a chupon, or bathing them in water right away.
[b] 'Identified proper cord care' outcome was coded as correct if respondent identified applying alcohol, applying iodine, keeping cord clean/dry, or applying peroxide, and did not identify applying oil, applying coffee, applying quina water, wrapping a fajero, applying powder, burning, applying ashes, applying a clamp, or applying thiomersal.
[c] 'Used appropriate treatment for diarrhea in past 4 weeks' outcome was coded as correct if respondent did any of the following: antibiotic, zinc, give extra food, give extra liquid, and did not do any of the following: anti-diarrhea medication, home remedy, laxative, deworming medication, chupon, massage, stop eating foods, stop taking liquids.
[d] 'Identified appropriate diarrhea treatment methods' outcome was coded as correct if respondent identified any of the following: antibiotic, zinc, ORS or Litrosol, give extra food, give extra liquid, get medical attention, and did not identify any of the following: anti-diarrhea medication, laxative, deworming medication, home remedy, chupon, massage, stop giving foods, stop giving liquids, or do nothing.

Table S7. Pooled total effect estimates of intervention on maternal, child, and neonatal outcomes, with adjustment for baseline demographic characteristics and baseline outcome, estimated on sub-sample of respondents who did not move between villages during study

	Outcome type	Respondent denominator	Pooled total effect estimate		N
			Effect size (95% CI)	p value	
**Prenatal care**					
Currently taking folic acid tablets (daily in past 7 days)	Practice	All women 15+ with a complete survey	0·0014 (-0·040 to 0·043)	0·95	5284
Made a birth plan in preparation for birth	Practice	All respondents with a complete survey and a child born since 2018-09-01 (after the end of the intervention)	-0·082 (-0·22 to 0·055)	0·24	278
Saved money in preparation for birth	Practice	All respondents with a complete survey and a child born since 2018-09-01 (after the end of the intervention)	-0·0050 (-0·14 to 0·13)	0·94	281
Sold animal in preparation for birth	Practice	All respondents with a complete survey and a child born since 2018-09-01 (after the end of the intervention)	NA	NA	NA
Sought prenatal care within 12 weeks	Practice	All respondents with a complete survey and a child born since 2018-09-01 (after the end of the intervention)	0·033 (-0·11 to 0·17)	0·64	264
Identifies that women should take folic acid before pregnancy	Knowledge and attitudes	All respondents with a complete survey	0·058 (0·026 to 0·090)	0·0004	8875
Identifies that women should seek prenatal care first 12 weeks of pregnancy	Knowledge and attitudes	All respondents with a complete survey	0·030 (0·0068 to 0·052)	0·011	8875
Identifies accompanying woman to prenatal care visits as method of support during pregnancy	Knowledge and attitudes	All respondents with a complete survey	NA	NA	NA
Identifies ensuring that woman eats well as method of support during pregnancy	Knowledge and attitudes	All respondents with a complete survey	NA	NA	NA
Identifies ensuring that woman rests 1 hour per day well as method of support during pregnancy	Knowledge and attitudes	All respondents with a complete survey	NA	NA	NA
Identifies avoiding violence as method of support during pregnancy	Knowledge and attitudes	All respondents with a complete survey	NA	NA	NA
Identifies encouraging women to take vitamins as method of support during pregnancy	Knowledge and attitudes	All respondents with a complete survey	NA	NA	NA
Identifies helping woman with house work/child care as method of support during pregnancy	Knowledge and attitudes	All respondents with a complete survey	NA	NA	NA
Identifies saving animals to sell as method of preparing for birth expenses	Knowledge and attitudes	All respondents with a complete survey	NA	NA	NA

Oles W, *et al. BMJ Open* 2024; 14:e060784. doi: 10.1136/bmjopen-2022-060784

Identifies knowing cost of trip to maternal clinic as method of preparing for birth expenses	Knowledge and attitudes	All respondents with a complete survey	NA	NA	NA
Identifies having a savings plan as method of preparing for birth expenses	Knowledge and attitudes	All respondents with a complete survey	NA	NA	NA
Correctly answered prenatal care knowledge riddle	Intervention knowledge	All respondents with a complete survey	NA	NA	NA
**Facility based birth**					
Gave birth in health facility	Practice	All respondents with a complete survey and a child born since 2018-09-01 (after the end of the intervention)	0·029 (-0·10 to 0·16)	0·66	281
Believes health facility is best place to give birth	Knowledge and attitudes	All respondents with a complete survey	0·017 (-0·0050 to 0·039)	0·13	8866
**Pregnancy danger signs**					
Woman sought medical care for pregnancy danger sign	Practice	All respondents with a complete survey and a child born since 2018-09-01 (after the end of the intervention) who experienced a prenatal care danger sign	-0·29 (-0·82 to 0·24)	0·29	51
Identified bleeding as pregnancy d.s.	Knowledge and attitudes	All respondents with a complete survey	0·054 (0·013 to 0·095)	0·010	8872
Identified seizure as pregnancy d.s.	Knowledge and attitudes	All respondents with a complete survey	0·0022 (-0·0078 to 0·012)	0·67	8863
Identified headache as pregnancy d.s.	Knowledge and attitudes	All respondents with a complete survey	0·042 (-0·0059 to 0·090)	0·086	8872
Identified ringing in ears as pregnancy d.s.	Knowledge and attitudes	All respondents with a complete survey	0·0025 (-0·0073 to 0·012)	0·62	8848
Identified dizziness as pregnancy d.s.	Knowledge and attitudes	All respondents with a complete survey	-0·0061 (-0·055 to 0·043)	0·81	8872
Identified difficulty urinating as pregnancy d.s.	Knowledge and attitudes	All respondents with a complete survey	-0·0023 (-0·010 to 0·0055)	0·57	8848
Identified reduced or absent fetal movement as pregnancy d.s.	Knowledge and attitudes	All respondents with a complete survey	0·014 (-0·0064 to 0·034)	0·18	8848
Identified water breaking as pregnancy d.s.	Knowledge and attitudes	All respondents with a complete survey	-0·0031 (-0·0098 to 0·0035)	0·36	8848
Identified fever as pregnancy d.s.	Knowledge and attitudes	All respondents with a complete survey	0·039 (0·0002 to 0·077)	0·049	8872
Identified swelling of face/hands/feet as pregnancy d.s.	Knowledge and attitudes	All respondents with a complete survey	0·032 (0·0017 to 0·063)	0·038	8872
**Postnatal care for mother**					
Mother had health checked by professional within 3 days of birth	Practice	All respondents with a complete survey and a child born since 2018-09-01 (after the end of the intervention)	-0·058 (-0·24 to 0·13)	0·54	275

Mother had health checked by professional within 7 days of birth	Practice	All respondents with a complete survey and a child born since 2018-09-01 (after the end of the intervention)	-0·060 (-0·27 to 0·15)	0·57	272
Mother sought medical care for postnatal danger sign	Practice	All respondents with a complete survey and a child born since 2018-09-01 (after the end of the intervention)	0·32 (-0·015 to 0·66)	0·061	29
Identifies that mother should receive postnatal medical check-up within 3 days of birth	Knowledge and attitudes	All respondents with a complete survey	0·071 (0·028 to 0·11)	0·0011	8872
Identifies that mother should receive postnatal medical check-up within 7 days of birth	Knowledge and attitudes	All respondents with a complete survey	0·039 (-0·012 to 0·090)	0·13	8872
Identified heavy vaginal bleeding as postnatal d.s.	Knowledge and attitudes	All respondents with a complete survey	0·070 (0·032 to 0·11)	0·0003	8871
Identified fever as postnatal d.s.	Knowledge and attitudes	All respondents with a complete survey	0·057 (0·0076 to 0·11)	0·024	8871
Identified vaginal discharge as postnatal d.s.	Knowledge and attitudes	All respondents with a complete survey	0·0035 (-0·011 to 0·018)	0·62	8856
Identified headache as postnatal d.s.	Knowledge and attitudes	All respondents with a complete survey	0·031 (-0·0079 to 0·069)	0·12	8871
Identified convulsions or fits as postnatal d.s.	Knowledge and attitudes	All respondents with a complete survey	0·0004 (-0·0054 to 0·0063)	0·88	8673
**Postnatal care for newborn**					
Newborn had health checked by professional within 3 days of birth	Practice	All respondents with a complete survey and a child born since 2018-09-01 (after the end of the intervention)	0·0038 (-0·15 to 0·16)	0·96	279
Newborn had health checked by professional within 7 days of birth	Practice	All respondents with a complete survey and a child born since 2018-09-01 (after the end of the intervention)	-0·013 (-0·20 to 0·17)	0·89	276
Newborn experienced health problem in first month, care was sought	Practice	All respondents with a complete survey and a child born since 2018-09-01 (after the end of the intervention)	-0·011 (-0·011 to -0·011)	<0·0001	21
Identified correct ways to provide newborn care[a]	Knowledge and attitudes	All respondents with a complete survey	0·022 (0·0089 to 0·035)	0·0010	8851
Identified fever as newborn d.s.	Knowledge and attitudes	All respondents with a complete survey	0·0037 (-0·044 to 0·051)	0·88	8873
Identified diarrhea as newborn d.s.	Knowledge and attitudes	All respondents with a complete survey	0·0081 (-0·034 to 0·050)	0·71	8873
Identified difficulty breathing as newborn d.s.	Knowledge and attitudes	All respondents with a complete survey	0·0061 (-0·030 to 0·042)	0·74	8849
Identified vomiting as newborn d.s.	Knowledge and attitudes	All respondents with a complete survey	0·0089 (-0·021 to 0·039)	0·56	8864
Identified poor appetite as newborn d.s.	Knowledge and attitudes	All respondents with a complete survey	-0·0045 (-0·015 to 0·0054)	0·37	8849
Identified redness/bleeding around cord as newborn d.s.	Knowledge and attitudes	All respondents with a complete survey	0·021 (-0·0012 to 0·043)	0·064	8873
Identified pus in cord as newborn d.s.	Knowledge and attitudes	All respondents with a complete survey	0·015 (-0·0020 to 0·031)	0·085	8864
Identified cold skin as newborn d.s.	Knowledge and attitudes	All respondents with a complete survey	0·0024 (-0·0069 to 0·012)	0·61	8849

Oles W, *et al. BMJ Open* 2024; 14:e060784. doi: 10.1136/bmjopen-2022-060784

Identified cough as newborn d.s.	Knowledge and attitudes	All respondents with a complete survey	-0·020 (-0·070 to 0·031)	0·45	8873
Identified cold as newborn d.s.	Knowledge and attitudes	All respondents with a complete survey	-0·014 (-0·066 to 0·038)	0·61	8873
Identified problems latching as newborn d.s.	Knowledge and attitudes	All respondents with a complete survey	0·0001 (-0·014 to 0·014)	0·99	8847
Identified pneumonia as newborn d.s.	Knowledge and attitudes	All respondents with a complete survey	NA	NA	NA
Did not use harmful substances to treat cord stump	Practice	All respondents with a complete survey and a child born since 2018-09-01 (after the end of the intervention)	0·11 (-0·068 to 0·29)	0·23	271
Did not wrap fajero around newborn in first 7 days after birth	Practice	All respondents with a complete survey and a child born since 2018-09-01 (after the end of the intervention)	0·29 (0·099 to 0·48)	0·0029	277
Identified proper cord care methods[b]	Knowledge and attitudes	All respondents with a complete survey	0·10 (0·053 to 0·15)	<0·0001	8874
Correctly answered proper cord care riddle	Intervention knowledge	All respondents with a complete survey	NA	NA	NA
Kept newborn wrapped first 7 days after birth	Practice	All respondents with a complete survey and a child born since 2018-09-01 (after the end of the intervention)	..	..	..
Held newborn skin-to-skin during first month after birth	Practice	All respondents with a complete survey and a child born since 2018-09-01 (after the end of the intervention)	0·051 (-0·21 to 0·31)	0·70	68
**Breastfeeding**					
Exclusively breastfed child first 6 months	Practice	All respondents with a complete survey and a child born since 2018-09-01 (after the end of the intervention)	-0·12 (-0·26 to 0·012)	0·073	280
Exclusively breastfed child first 6 months without giving chupón	Practice	All respondents with a complete survey and a child born since 2018-09-01 (after the end of the intervention)	-0·060 (-0·24 to 0·12)	0·51	281
Breastfed immediately after birth	Practice	All respondents with a complete survey and a child born since 2018-09-01 (after the end of the intervention) who reported a natural birth	-0·0011 (-0·12 to 0·11)	0·98	210
Identifies that newborn should be breastfed immediately after birth	Knowledge and attitudes	All respondents with a complete survey	0·016 (-0·019 to 0·050)	0·38	8875
Identifies that newborns should only be given breast milk during first 6 months	Knowledge and attitudes	All respondents with a complete survey	0·039 (0·0080 to 0·069)	0·014	8874
Believes newborns should not be given chupón during first 6 months	Knowledge and attitudes	All respondents with a complete survey	0·16 (0·10 to 0·22)	<0·0001	8875
**Paternal involvement**					
Father accompanied mother to clinic for prenatal care visit at least once	Practice	All respondents with a complete survey and a child born since 2018-09-01 (after the end of the intervention) who received prenatal care	0·074 (-0·11 to 0·26)	0·44	259
Father accompanied mother to seek medical care for pregnancy danger sign	Practice	All respondents with a complete survey and a child born since 2018-09-01 (after the end of the intervention) who experienced a prenatal care danger sign and sought care	..	..	..

Oles W, *et al. BMJ Open* 2024; 14:e060784. doi: 10.1136/bmjopen-2022-060784

Father waited at birthplace during labor	Practice	All respondents with a complete survey and a child born since 2018-09-01 (after the end of the intervention)	-0·031 (-0·19 to 0·13)	0·70	281
Father accompanied mother to seek medical care for postnatal danger sign	Practice	All respondents with a complete survey and a child born since 2018-09-01 (after the end of the intervention) and sought care for postpartum problem	..	..	..
Father sought medical care for newborn for postnatal danger sign	Practice	All respondents with a complete survey and a child born since 2018-09-01 (after the end of the intervention) and sought care for a postnatal problem	..	..	..
Father held child	Practice	All respondents with a complete survey and a child born since 2018-09-01 (after the end of the intervention)	0·057 (-0·046 to 0·16)	0·28	258
Father played with child	Practice	All respondents with a complete survey and a child born since 2018-09-01 (after the end of the intervention)	0·073 (-0·043 to 0·19)	0·22	262
Father took child to clinic when sick	Practice	All respondents with a complete survey and a child born since 2018-09-01 (after the end of the intervention)	0·089 (-0·086 to 0·26)	0·32	262
Believes father should accompany mother to prenatal care visits	Knowledge and attitudes	All respondents with a complete survey	0·039 (0·017 to 0·061)	0·0006	8807
Believes father should wait at birth location while mother gives birth	Knowledge and attitudes	All respondents with a complete survey	0·013 (-0·0057 to 0·032)	0·17	8810
Believes father should care for children when sick	Knowledge and attitudes	All respondents with a complete survey	0·0004 (-0·0058 to 0·0065)	0·90	8851
**Diarrhea management**					
Did not report diarrhea in past 4 weeks	Practice	All respondents with a complete survey	-0·0095 (-0·026 to 0·0068)	0·26	8861
Used ORT to treat diarrhea in past 4 weeks	Practice	All respondents with a complete survey who had diarrhea in past 4 weeks	..	..	..
Used appropriate treatment for diarrhea in past 4 weeks[c]	Practice	All respondents with a complete survey who had diarrhea in past 4 weeks	..	..	..
Child did not experience diarrhea past 4 weeks	Practice	All respondents with a complete survey and a new child <= 5 years old reported at wave 3 who had diarrhea in past 4 weeks	-0·0094 (-0·054 to 0·035)	0·68	2257
Child experienced diarrhea past 4 weeks, was given appropriate amount of liquids	Practice	All respondents with a complete survey and a new child <= 5 years old reported at wave 3 who had diarrhea in past 4 weeks	1·9 (1·4 to 2·4)	<0·0001	41
Child experienced diarrhea past 4 weeks, was given appropriate amount of food	Practice	All respondents with a complete survey and a new child <= 5 years old reported at wave 3 who had diarrhea in past 4 weeks	1·2 (0·74 to 1·6)	<0·0001	36
Child experienced diarrhea past 4 weeks, was given ORT	Practice	All respondents with a complete survey and a new child <= 5 years old reported at wave 3 who had diarrhea in past 4 weeks	1·0 (0·62 to 1·4)	<0·0001	41
Child experienced diarrhea past 4 weeks, was given appropriate treatment	Practice	All respondents with a complete survey and a new child <= 5 years old reported at wave 3 who had diarrhea in past 4 weeks	0·58 (0·16 to 1·0)	0·0071	46
Child experienced diarrhea past 4 weeks, was given zinc 10-14 days	Practice	All respondents with a complete survey and a new child <= 5 years old reported at wave 3 who had diarrhea in past 4 weeks	..	..	..
Identified appropriate diarrhea treatment methods[d]	Knowledge and attitudes	All respondents with a complete survey	0·10 (0·051 to 0·16)	0·0001	8873
Identified zinc 10-14 days as diarrhea treatment	Knowledge and attitudes	All respondents with a complete survey	0·033 (0·0091 to 0·057)	0·0068	8835

Identified zinc supplement as way to prevent diarrhea	Knowledge and attitudes	All respondents with a complete survey	0·071 (0·0039 to 0·14)	0·038	8875
Identified breastfeeding as way to prevent diarrhea	Knowledge and attitudes	All respondents with a complete survey	0·038 (-0·024 to 0·099)	0·23	8875
Correctly answered diarrhea treatment with zinc riddle	Intervention knowledge	All respondents with a complete survey	NA	NA	NA
**Respiratory illness**					
Did not report respiratory illness (coughing) for 2 weeks	Practice	All respondents with a complete survey	-0·015 (-0·048 to 0·019)	0·39	8868
Child did not have cough past 4 weeks	Practice	All respondents with a complete survey	-0·044 (-0·11 to 0·022)	0·19	2257
Child had cough past 4 weeks, care was sought	Practice	All respondents with a complete survey and a new child <= 5 years old reported at wave 3 who had an illness with a cough in past 4 weeks	0·0005 (-0·24 to 0·24)	1·00	187
Identified vaccination as way to prevent respiratory illness	Knowledge and attitudes	All respondents with a complete survey	-0·0026 (-0·040 to 0·035)	0·89	8875
Identified breastfeeding as way to prevent respiratory illness	Knowledge and attitudes	All respondents with a complete survey	0·029 (-0·023 to 0·081)	0·27	8875
Identified fever as d.s. for children with respiratory illness	Knowledge and attitudes	All respondents with a complete survey	0·017 (-0·011 to 0·044)	0·23	8866
Identified difficulty breathing as d.s. for children with respiratory illness	Knowledge and attitudes	All respondents with a complete survey	0·011 (-0·0048 to 0·028)	0·17	8866
Identified rapid breathing as d.s. for children with respiratory illness	Knowledge and attitudes	All respondents with a complete survey	0·0086 (-0·0022 to 0·019)	0·12	8866
**Reproductive health**					
Reported ever using birth control to delay or avoid pregnancy	Practice	All respondents with a complete survey	0·0035 (-0·045 to 0·052)	0·89	8172
Reported currently using birth control	Practice	All respondents with a complete survey who are not pregnant and have ever used birth control	0·058 (-0·035 to 0·15)	0·22	1868
Believes woman should be at least 18 years of age to have her first child	Knowledge and attitudes	All respondents with a complete survey	0·012 (0·0043 to 0·019)	0·0018	8276
**Gender/reproductive norms**					
Birth location chosen either jointly or by woman	Practice	All respondents with a complete survey and a child born since 2018-09-01 (after the end of the intervention)	0·063 (-0·067 to 0·19)	0·34	277

Results based on sub-sample of respondents who didn't move villages during the study who also completed baseline survey, N=8981 (household targeted n=2831, household not targeted n=6150). Covariates included baseline age, gender, education, marital status, indigenous status, household wealth index, and the related outcome. d.s.=Danger sign, NA=Not applicable, ORT=Oral rehydration therapy. ··Model did not converge. Results with NA could not be assessed

Oles W, *et al. BMJ Open* 2024; 14:e060784. doi: 10.1136/bmjopen-2022-060784

because outcome was not included in baseline survey. Covariates were dropped sequentially if they predicted outcome perfectly. Standard errors are clustered at the village level. Robust 95% CIs in parentheses.

[a] ‘Identified correct ways to provide newborn care’ outcome was coded as correct if the respondent identified holding baby skin to skin, getting their health checked by a professional, immediately putting them to the breast, or making sure their diaper is clean and dry, and did not identify giving purgante, wrapping a fajero, giving a chupon, or bathing them in water right away.
[b] ‘Identified proper cord care’ outcome was coded as correct if respondent identified applying alcohol, applying iodine, keeping cord clean/dry, or applying peroxide, and did not identify applying oil, applying coffee, applying quina water, wrapping a fajero, applying powder, burning, applying ashes, applying a clamp, or applying thiomersal.
[c] ‘Used appropriate treatment for diarrhea in past 4 weeks’ outcome was coded as correct if respondent did any of the following: antibiotic, zinc, give extra food, give extra liquid, and did not do any of the following: anti-diarrhea medication, home remedy, laxative, deworming medication, chupon, massage, stop eating foods, stop taking liquids.
[d] ‘Identified appropriate diarrhea treatment methods’ outcome was coded as correct if respondent identified any of the following: antibiotic, zinc, ORS or Litrosol, give extra food, give extra liquid, get medical attention, and did not identify any of the following: anti-diarrhea medication, laxative, deworming medication, home remedy, chupon, massage, stop giving foods, stop giving liquids, or do nothing.

Table S8. Pooled total effect estimates of intervention on maternal, child, and neonatal outcomes, intervention module receipt defined as treatment, with adjustment for baseline demographic characteristics

	Outcome type	Respondent denominator	Intervention module(s)	Pooled total effect estimate		N
				Effect size (95% CI)	p value	
**Prenatal care**						
Currently taking folic acid tablets (daily in past 7 days)	Practice	All women 15+ with a complete survey	m11	0·017 (-0·035 to 0·070)	0·52	5922
Made a birth plan in preparation for birth	Practice	All respondents with a complete survey and a child born since 2018-09-01 (after the end of the intervention)	m2	0·021 (-0·12 to 0·16)	0·77	536
Saved money in preparation for birth	Practice	All respondents with a complete survey and a child born since 2018-09-01 (after the end of the intervention)	m1	-0·061 (-0·19 to 0·073)	0·37	540
Sold animal in preparation for birth	Practice	All respondents with a complete survey and a child born since 2018-09-01 (after the end of the intervention)	m1	-0·0070 (-0·090 to 0·076)	0·87	535
Sought prenatal care within 12 weeks	Practice	All respondents with a complete survey and a child born since 2018-09-01 (after the end of the intervention)	m1	-0·037 (-0·20 to 0·13)	0·67	518
Identifies that women should take folic acid before pregnancy	Knowledge and attitudes	All respondents with a complete survey	m11	0·097 (0·063 to 0·13)	<0·0001	9197
Identifies that women should seek prenatal care first 12 weeks of pregnancy	Knowledge and attitudes	All respondents with a complete survey	m1	0·060 (0·040 to 0·081)	<0·0001	9196
Identifies accompanying woman to prenatal care visits as method of support during pregnancy	Knowledge and attitudes	All respondents with a complete survey	m1	0·0012 (-0·057 to 0·060)	0·97	9192
Identifies ensuring that woman eats well as method of support during pregnancy	Knowledge and attitudes	All respondents with a complete survey	m1	-0·022 (-0·084 to 0·039)	0·48	9192
Identifies ensuring that woman rests 1 hour per day well as method of support during pregnancy	Knowledge and attitudes	All respondents with a complete survey	m1	0·0053 (-0·038 to 0·048)	0·81	9183
Identifies avoiding violence as method of support during pregnancy	Knowledge and attitudes	All respondents with a complete survey	m1	-0·032 (-0·054 to -0·0097)	0·0049	9192
Identifies encouraging women to take vitamins as method of support during pregnancy	Knowledge and attitudes	All respondents with a complete survey	m1	-0·038 (-0·074 to -0·0016)	0·041	9192

Oles W, *et al. BMJ Open* 2024; 14:e060784. doi: 10.1136/bmjopen-2022-060784

Identifies helping woman with house work/child care as method of support during pregnancy	Knowledge and attitudes	All respondents with a complete survey	m1	0·099 (0·032 to 0·17)	0·0037	9192
Identifies saving animals to sell as method of preparing for birth expenses	Knowledge and attitudes	All respondents with a complete survey	m1	0·026 (-0·0042 to 0·057)	0·091	9187
Identifies knowing cost of trip to maternal clinic as method of preparing for birth expenses	Knowledge and attitudes	All respondents with a complete survey	m1	0·0057 (-0·036 to 0·047)	0·79	9196
Identifies having a savings plan as method of preparing for birth expenses	Knowledge and attitudes	All respondents with a complete survey	m1	0·059 (0·036 to 0·083)	<0·0001	9172
Correctly answered prenatal care knowledge riddle	Intervention knowledge	All respondents with a complete survey	m1	0·12 (0·079 to 0·17)	<0·0001	9196
**Facility based birth**						
Gave birth in health facility	Practice	All respondents with a complete survey and a child born since 2018-09-01 (after the end of the intervention)	m3	-0·039 (-0·18 to 0·10)	0·58	540
Believes health facility is best place to give birth	Knowledge and attitudes	All respondents with a complete survey	m3	0·034 (0·0069 to 0·061)	0·014	9188
**Pregnancy danger signs**						
Woman sought medical care for pregnancy danger sign	Practice	All respondents with a complete survey and a child born since 2018-09-01 (after the end of the intervention) who experienced a prenatal care danger sign	m1	0·025 (-0·22 to 0·27)	0·84	209
Identified bleeding as pregnancy d.s.	Knowledge and attitudes	All respondents with a complete survey	m1	0·070 (0·023 to 0·12)	0·0035	9195
Identified seizure as pregnancy d.s.	Knowledge and attitudes	All respondents with a complete survey	m1	-0·0032 (-0·013 to 0·0066)	0·53	9186
Identified headache as pregnancy d.s.	Knowledge and attitudes	All respondents with a complete survey	m1	0·059 (0·0056 to 0·11)	0·030	9195
Identified ringing in ears as pregnancy d.s.	Knowledge and attitudes	All respondents with a complete survey	m1	0·0044 (-0·0074 to 0·016)	0·46	9171
Identified dizziness as pregnancy d.s.	Knowledge and attitudes	All respondents with a complete survey	m1	-0·025 (-0·085 to 0·036)	0·43	9195
Identified difficulty urinating as pregnancy d.s.	Knowledge and attitudes	All respondents with a complete survey	m1	-0·0066 (-0·014 to 0·0008)	0·079	9171
Identified reduced or absent fetal movement as pregnancy d.s.	Knowledge and attitudes	All respondents with a complete survey	m1	0·032 (0·0006 to 0·062)	0·046	9171
Identified water breaking as pregnancy d.s.	Knowledge and attitudes	All respondents with a complete survey	m1	0·0017 (-0·0078 to 0·011)	0·72	9171
Identified fever as pregnancy d.s.	Knowledge and attitudes	All respondents with a complete survey	m1	0·066 (0·015 to 0·12)	0·012	9195

Identified swelling of face/hands/feet as pregnancy d.s.	Knowledge and attitudes	All respondents with a complete survey	m1	0·064 (0·022 to 0·11)	0·0028	9195
**Postnatal care for mother**						
Mother had health checked by professional within 3 days of birth	Practice	All respondents with a complete survey and a child born since 2018-09-01 (after the end of the intervention)	m5, m6	0·22 (0·034 to 0·40)	0·020	530
Mother had health checked by professional within 7 days of birth	Practice	All respondents with a complete survey and a child born since 2018-09-01 (after the end of the intervention)	m5, m6	0·15 (-0·0047 to 0·31)	0·057	530
Mother sought medical care for postnatal danger sign	Practice	All respondents with a complete survey and a child born since 2018-09-01 (after the end of the intervention)	m5, m6	0·20 (-0·027 to 0·42)	0·084	178
Identifies that mother should receive postnatal medical check-up within 3 days of birth	Knowledge and attitudes	All respondents with a complete survey	m5, m6	0·13 (0·075 to 0·18)	<0·0001	9196
Identifies that mother should receive postnatal medical check-up within 7 days of birth	Knowledge and attitudes	All respondents with a complete survey	m5, m6	0·100 (0·048 to 0·15)	0·00010	9196
Identified heavy vaginal bleeding as postnatal d.s.	Knowledge and attitudes	All respondents with a complete survey	m5, m6	0·12 (0·068 to 0·16)	<0·0001	9195
Identified fever as postnatal d.s.	Knowledge and attitudes	All respondents with a complete survey	m5, m6	0·095 (0·036 to 0·15)	0·0016	9195
Identified vaginal discharge as postnatal d.s.	Knowledge and attitudes	All respondents with a complete survey	m5, m6	0·0062 (-0·014 to 0·026)	0·54	9180
Identified headache as postnatal d.s.	Knowledge and attitudes	All respondents with a complete survey	m5, m6	0·018 (-0·026 to 0·061)	0·42	9195
Identified convulsions or fits as postnatal d.s.	Knowledge and attitudes	All respondents with a complete survey	m5, m6	0·0050 (-0·0054 to 0·016)	0·35	8983
**Postnatal care for newborn**						
Newborn had health checked by professional within 3 days of birth	Practice	All respondents with a complete survey and a child born since 2018-09-01 (after the end of the intervention)	m5, m6	0·30 (0·13 to 0·47)	0·0006	532
Newborn had health checked by professional within 7 days of birth	Practice	All respondents with a complete survey and a child born since 2018-09-01 (after the end of the intervention)	m5, m6	0·22 (0·064 to 0·37)	0·0055	532
Newborn experienced health problem in first month, care was sought	Practice	All respondents with a complete survey and a child born since 2018-09-01 (after the end of the intervention)	m5, m6	0·25 (0·049 to 0·45)	0·015	154
Identified correct ways to provide newborn care[a]	Knowledge and attitudes	All respondents with a complete survey	m5, m6, m7	0·029 (0·010 to 0·047)	0·0021	9172
Identified fever as newborn d.s.	Knowledge and attitudes	All respondents with a complete survey	m7	-0·0012 (-0·054 to 0·052)	0·96	9195

Oles W, *et al. BMJ Open* 2024; 14:e060784. doi: 10.1136/bmjopen-2022-060784

Identified diarrhea as newborn d.s.	Knowledge and attitudes	All respondents with a complete survey	m7	0·0025 (-0·049 to 0·054)	0·92	9195
Identified difficulty breathing as newborn d.s.	Knowledge and attitudes	All respondents with a complete survey	m7	0·028 (-0·013 to 0·070)	0·18	9171
Identified vomiting as newborn d.s.	Knowledge and attitudes	All respondents with a complete survey	m7	0·023 (-0·018 to 0·064)	0·27	9186
Identified poor appetite as newborn d.s.	Knowledge and attitudes	All respondents with a complete survey	m7	0·0060 (-0·013 to 0·025)	0·54	9171
Identified redness/bleeding around cord as newborn d.s.	Knowledge and attitudes	All respondents with a complete survey	m7	0·032 (0·0034 to 0·060)	0·028	9195
Identified pus in cord as newborn d.s.	Knowledge and attitudes	All respondents with a complete survey	m7	0·032 (0·0051 to 0·060)	0·020	9186
Identified cold skin as newborn d.s.	Knowledge and attitudes	All respondents with a complete survey	m7	0·012 (-0·0079 to 0·033)	0·23	9171
Identified cough as newborn d.s.	Knowledge and attitudes	All respondents with a complete survey	m7	-0·033 (-0·087 to 0·021)	0·23	9195
Identified cold as newborn d.s.	Knowledge and attitudes	All respondents with a complete survey	m7	-0·029 (-0·090 to 0·031)	0·34	9195
Identified problems latching as newborn d.s.	Knowledge and attitudes	All respondents with a complete survey	m7	0·0028 (-0·016 to 0·021)	0·77	9171
Identified pneumonia as newborn d.s.	Knowledge and attitudes	All respondents with a complete survey	m7	0·028 (-0·016 to 0·072)	0·22	9171
Did not use harmful substances to treat cord stump	Practice	All respondents with a complete survey and a child born since 2018-09-01 (after the end of the intervention)	m5, m6, m7	0·11 (-0·045 to 0·26)	0·17	527
Did not wrap fajero around newborn in first 7 days after birth	Practice	All respondents with a complete survey and a child born since 2018-09-01 (after the end of the intervention)	m5, m6, m7	0·30 (0·13 to 0·47)	0·0005	535
Identified proper cord care methods[b]	Knowledge and attitudes	All respondents with a complete survey	m5, m6, m7	0·16 (0·10 to 0·22)	<0·0001	9195
Correctly answered proper cord care riddle	Intervention knowledge	All respondents with a complete survey	m5, m6, m7	0·30 (0·25 to 0·34)	<0·0001	9197
Kept newborn wrapped first 7 days after birth	Practice	All respondents with a complete survey and a child born since 2018-09-01 (after the end of the intervention)	m5, m6, m7	-0·0064 (-0·059 to 0·047)	0·81	530
Held newborn skin-to-skin during first month after birth	Practice	All respondents with a complete survey and a child born since 2018-09-01 (after the end of the intervention)	m5, m6, m7	0·22 (0·081 to 0·36)	0·0020	537
**Breastfeeding**						
Exclusively breastfed child first 6 months	Practice	All respondents with a complete survey and a child born since 2018-09-01 (after the end of the intervention)	m5, m6, m7, m8	0·11 (-0·031 to 0·25)	0·13	539
Exclusively breastfed child first 6 months without giving chupón	Practice	All respondents with a complete survey and a child born since 2018-09-01 (after the end of the intervention)	m5, m6, m7, m8	0·20 (0·058 to 0·35)	0·0060	539

Breastfed immediately after birth	Practice	All respondents with a complete survey and a child born since 2018-09-01 (after the end of the intervention) who reported a natural birth	m5, m6, m7, m8	0·071 (-0·033 to 0·18)	0·18	428
Identifies that newborn should be breastfed immediately after birth	Knowledge and attitudes	All respondents with a complete survey	m5, m6, m7, m8	0·037 (0·0018 to 0·072)	0·040	9196
Identifies that newborns should only be given breast milk during first 6 months	Knowledge and attitudes	All respondents with a complete survey	m5, m6, m7, m8	0·061 (0·030 to 0·092)	0·0001	9196
Believes newborns should not be given chupón during first 6 months	Knowledge and attitudes	All respondents with a complete survey	m5, m6, m7, m8	0·22 (0·16 to 0·28)	<0·0001	9196
**Paternal involvement**						
Father accompanied mother to clinic for prenatal care visit at least once	Practice	All respondents with a complete survey and a child born since 2018-09-01 (after the end of the intervention) who received prenatal care	m5, m6, m7	-0·071 (-0·30 to 0·16)	0·54	519
Father accompanied mother to seek medical care for pregnancy danger sign	Practice	All respondents with a complete survey and a child born since 2018-09-01 (after the end of the intervention) who experienced a prenatal care danger sign and sought care	m5, m6, m7	0·073 (-0·24 to 0·39)	0·65	134
Father waited at birthplace during labor	Practice	All respondents with a complete survey and a child born since 2018-09-01 (after the end of the intervention)	m5, m6, m7	-0·019 (-0·16 to 0·12)	0·79	540
Father accompanied mother to seek medical care for postnatal danger sign	Practice	All respondents with a complete survey and a child born since 2018-09-01 (after the end of the intervention) and sought care for postpartum problem	m5, m6, m7	0·070 (-0·26 to 0·40)	0·68	93
Father sought medical care for newborn for postnatal danger sign	Practice	All respondents with a complete survey and a child born since 2018-09-01 (after the end of the intervention) and sought care for a postnatal problem	m5, m6, m7	0·044 (-0·24 to 0·33)	0·76	98
Father held child	Practice	All respondents with a complete survey and a child born since 2018-09-01 (after the end of the intervention)	m5, m6, m7	-0·010 (-0·11 to 0·091)	0·85	535
Father played with child	Practice	All respondents with a complete survey and a child born since 2018-09-01 (after the end of the intervention)	m5, m6, m7	-0·045 (-0·20 to 0·11)	0·56	535
Father took child to clinic when sick	Practice	All respondents with a complete survey and a child born since 2018-09-01 (after the end of the intervention)	m5, m6, m7	-0·086 (-0·24 to 0·072)	0·29	535
Believes father should accompany mother to prenatal care visits	Knowledge and attitudes	All respondents with a complete survey	m5, m6, m7	0·044 (0·021 to 0·067)	0·0002	9158
Believes father should wait at birth location while mother gives birth	Knowledge and attitudes	All respondents with a complete survey	m5, m6, m7	0·018 (-0·0006 to 0·037)	0·058	9156

Oles W, *et al. BMJ Open* 2024; 14:e060784. doi: 10.1136/bmjopen-2022-060784

Believes father should care for children when sick	Knowledge and attitudes	All respondents with a complete survey	m5, m6, m7	0·0005 (-0·0077 to 0·0086)	0·91	9177
**Diarrhea management**						
Did not report diarrhea in past 4 weeks	Practice	All respondents with a complete survey	m9	-0·0069 (-0·025 to 0·011)	0·46	9182
Used ORT to treat diarrhea in past 4 weeks	Practice	All respondents with a complete survey who had diarrhea in past 4 weeks	m9	-0·098 (-0·28 to 0·087)	0·30	241
Used appropriate treatment for diarrhea in past 4 weeks[c]	Practice	All respondents with a complete survey who had diarrhea in past 4 weeks	m9	-0·021 (-0·16 to 0·12)	0·78	243
Child did not experience diarrhea past 4 weeks	Practice	All respondents with a complete survey and a new child <= 5 years old reported at wave 3 who had diarrhea in past 4 weeks	m9	-0·023 (-0·070 to 0·024)	0·34	3033
Child experienced diarrhea past 4 weeks, was given appropriate amount of liquids	Practice	All respondents with a complete survey and a new child <= 5 years old reported at wave 3 who had diarrhea in past 4 weeks	m9	-0·15 (-0·35 to 0·050)	0·14	310
Child experienced diarrhea past 4 weeks, was given appropriate amount of food	Practice	All respondents with a complete survey and a new child <= 5 years old reported at wave 3 who had diarrhea in past 4 weeks	m9	-0·12 (-0·26 to 0·015)	0·081	313
Child experienced diarrhea past 4 weeks, was given ORT	Practice	All respondents with a complete survey and a new child <= 5 years old reported at wave 3 who had diarrhea in past 4 weeks	m9	0·15 (-0·041 to 0·35)	0·12	310
Child experienced diarrhea past 4 weeks, was given appropriate treatment	Practice	All respondents with a complete survey and a new child <= 5 years old reported at wave 3 who had diarrhea in past 4 weeks	m9	0·17 (-0·020 to 0·36)	0·080	312
Child experienced diarrhea past 4 weeks, was given zinc 10-14 days	Practice	All respondents with a complete survey and a new child <= 5 years old reported at wave 3 who had diarrhea in past 4 weeks	m9	..	..	..
Identified appropriate diarrhea treatment methods[d]	Knowledge and attitudes	All respondents with a complete survey	m9	0·13 (0·069 to 0·19)	<0·0001	9195
Identified zinc 10-14 days as diarrhea treatment	Knowledge and attitudes	All respondents with a complete survey	m9	0·054 (0·021 to 0·087)	0·0013	9181
Identified zinc supplement as way to prevent diarrhea	Knowledge and attitudes	All respondents with a complete survey	m9	0·071 (0·0038 to 0·14)	0·038	9196
Identified breastfeeding as way to prevent diarrhea	Knowledge and attitudes	All respondents with a complete survey	m9	0·038 (-0·023 to 0·099)	0·23	9196
Correctly answered diarrhea treatment with zinc riddle	Intervention knowledge	All respondents with a complete survey	m9	0·35 (0·30 to 0·40)	<0·0001	9197
**Respiratory illness**						
Did not report respiratory illness (coughing) for 2 weeks	Practice	All respondents with a complete survey	m9	-0·012 (-0·051 to 0·028)	0·56	9188
Child did not have cough past 4 weeks	Practice	All respondents with a complete survey	m9	-0·058 (-0·12 to 0·0070)	0·081	3034

Child had cough past 4 weeks, care was sought	Practice	All respondents with a complete survey and a new child <= 5 years old reported at wave 3 who had an illness with a cough in past 4 weeks	m9	0·064 (-0·090 to 0·22)	0·41	647
Identified vaccination as way to prevent respiratory illness	Knowledge and attitudes	All respondents with a complete survey	m9	-0·0053 (-0·046 to 0·036)	0·80	9196
Identified breastfeeding as way to prevent respiratory illness	Knowledge and attitudes	All respondents with a complete survey	m9	0·013 (-0·045 to 0·070)	0·66	9196
Identified fever as d.s. for children with respiratory illness	Knowledge and attitudes	All respondents with a complete survey	m9	0·021 (-0·0036 to 0·046)	0·093	9187
Identified difficulty breathing as d.s. for children with respiratory illness	Knowledge and attitudes	All respondents with a complete survey	m9	0·011 (-0·0046 to 0·026)	0·17	9187
Identified rapid breathing as d.s. for children with respiratory illness	Knowledge and attitudes	All respondents with a complete survey	m9	0·0096 (-0·0010 to 0·020)	0·075	9187
**Reproductive health**						
Reported ever using birth control to delay or avoid pregnancy	Practice	All respondents with a complete survey	m4, m10, m12	-0·017 (-0·069 to 0·036)	0·53	9191
Reported currently using birth control	Practice	All respondents with a complete survey who are not pregnant and have ever used birth control	m4, m10, m12	0·033 (-0·035 to 0·10)	0·34	3420
Believes woman should be at least 18 years of age to have her first child	Knowledge and attitudes	All respondents with a complete survey	m4, m10, m12	0·017 (0·0098 to 0·025)	<0·0001	8713
**Gender/reproductive norms**						
Birth location chosen either jointly or by woman	Practice	All respondents with a complete survey and a child born since 2018-09-01 (after the end of the intervention)	m14	0·055 (-0·036 to 0·15)	0·24	540

Results based on sub-sample that completed baseline survey, N=9285 (household targeted n=2919, household not targeted n=6366). Baseline covariates included age, gender, education, marital status, indigenous status, and household wealth index. d.s.=Danger sign, ORT=Oral rehydration therapy. ··Model did not converge. Covariates were dropped sequentially if they predicted outcome perfectly. Standard errors are clustered at the village level. Robust 95% CIs in parentheses.

[a] 'Identified correct ways to provide newborn care' outcome was coded as correct if the respondent identified holding baby skin to skin, getting their health checked by a professional, immediately putting them to the breast, or making sure their diaper is clean and dry, and did not identify giving purgante, wrapping a fajero, giving a chupon, or bathing them in water right away.
[b] 'Identified proper cord care' outcome was coded as correct if respondent identified applying alcohol, applying iodine, keeping cord clean/dry, or applying peroxide, and did not identify applying oil, applying coffee, applying quina water, wrapping a fajero, applying powder, burning, applying ashes, applying a clamp, or applying thiomersal.

[c] 'Used appropriate treatment for diarrhea in past 4 weeks' outcome was coded as correct if respondent did any of the following: antibiotic, zinc, give extra food, give extra liquid, and did not do any of the following: anti-diarrhea medication, home remedy, laxative, deworming medication, chupon, massage, stop eating foods, stop taking liquids.
[d] 'Identified appropriate diarrhea treatment methods' outcome was coded as correct if respondent identified any of the following: antibiotic, zinc, ORS or Litrosol, give extra food, give extra liquid, get medical attention, and did not identify any of the following: anti-diarrhea medication, laxative, deworming medication, home remedy, chupon, massage, stop giving foods, stop giving liquids, or do nothing.

Table S9. Absolute changes in primary and secondary outcomes from baseline to endline survey

	Outcome type	Respondent denominator	Baseline			Endline			Change in outcome %, untargeted	Change in outcome %, targeted
			Outcome %, untargeted	Outcome %, targeted	p value	Outcome %, untargeted	Outcome %, targeted	p value		
**Prenatal care**										
Currently taking folic acid tablets (daily in past 7 days)	Practice	All women 15+ with a complete survey	30·7	32·3	0·255	20·3	21·0	0·562	-10·4	-11·3
Made a birth plan in preparation for birth	Practice	All respondents with a complete survey and a child born since 2018-09-01 (after the end of the intervention)	63·7	58·9	0·011	78·2	77·1	0·834	+14·5	+18·2
Saved money in preparation for birth	Practice	All respondents with a complete survey and a child born since 2018-09-01 (after the end of the intervention)	64·5	62·9	0·391	79·7	77·3	0·545	+15·2	+14·4
Sold animal in preparation for birth	Practice	All respondents with a complete survey and a child born since 2018-09-01 (after the end of the intervention)	NA	NA	NA	9·6	9·0	0·937	NA	NA
Sought prenatal care within 12 weeks	Practice	All respondents with a complete survey and a child born since 2018-09-01 (after the end of the intervention)	73·0	73·8	0·666	78·2	75·4	0·502	+5·2	+1·6
Identifies that women should take folic acid before pregnancy	Knowledge and attitudes	All respondents with a complete survey	73·7	73·3	0·728	76·0	82·0	<0·001	+2·3	+8·7
Identifies that women should seek prenatal care first 12 weeks of pregnancy	Knowledge and attitudes	All respondents with a complete survey	86·1	86·5	0·665	85·4	88·0	<0·001	-0·7	+1·5
Identifies accompanying woman to prenatal care visits as method of support during pregnancy	Knowledge and attitudes	All respondents with a complete survey	NA	NA	NA	30·5	33·0	0·013	NA	NA

Oles W, *et al. BMJ Open* 2024; 14:e060784. doi: 10.1136/bmjopen-2022-060784

Identifies ensuring that woman eats well as method of support during pregnancy	Knowledge and attitudes	All respondents with a complete survey	NA	NA	NA	29·4	26·6	0·004	NA	NA
Identifies ensuring that woman rests 1 hour per day well as method of support during pregnancy	Knowledge and attitudes	All respondents with a complete survey	NA	NA	NA	11·3	11·3	0·998	NA	NA
Identifies avoiding violence as method of support during pregnancy	Knowledge and attitudes	All respondents with a complete survey	NA	NA	NA	7·2	5·8	0·011	NA	NA
Identifies encouraging women to take vitamins as method of support during pregnancy	Knowledge and attitudes	All respondents with a complete survey	NA	NA	NA	12·2	10·5	0·016	NA	NA
Identifies helping woman with house work/child care as method of support during pregnancy	Knowledge and attitudes	All respondents with a complete survey	NA	NA	NA	55·2	56·8	0·124	NA	NA
Identifies saving animals to sell as method of preparing for birth expenses	Knowledge and attitudes	All respondents with a complete survey	NA	NA	NA	3·7	5·2	<0·001	NA	NA
Identifies knowing cost of trip to maternal clinic as method of preparing for birth expenses	Knowledge and attitudes	All respondents with a complete survey	NA	NA	NA	8·0	7·1	0·125	NA	NA
Identifies having a savings plan as method of preparing for birth expenses	Knowledge and attitudes	All respondents with a complete survey	NA	NA	NA	89·4	92·2	<0·001	NA	NA
Correctly answered prenatal care knowledge riddle	Intervention knowledge	All respondents with a complete survey	NA	NA	NA	11·2	16·1	<0·001	NA	NA
**Facility based birth**										
Gave birth in health facility	Practice	All respondents with a complete survey and a child born since 2018-09-01 (after the end of the intervention)	74·8	72·3	0·142	84·4	81·0	0·352	+9·6	+8·7
Believes health facility is best place to give birth	Knowledge and attitudes	All respondents with a complete survey	91·1	90·2	0·19	92·8	95·0	<0·001	+1·7	+4·8
**Pregnancy danger signs**										
Woman sought medical care for pregnancy danger sign	Practice	All respondents with a complete survey and a child born since 2018-	64·7	63·9	0·852	63·2	62·7	1	-1·5	-1·2

		09-01 (after the end of the intervention) who experienced a prenatal care danger sign							
Identified bleeding as pregnancy d.s.	Knowledge and attitudes	All respondents with a complete survey	41·5	41·4	0·952	27·5	29·5	0·034	-14·0	-11·9
Identified seizure as pregnancy d.s.	Knowledge and attitudes	All respondents with a complete survey	6·3	5·4	0·103	1·5	1·9	0·229	-4·8	-3·5
Identified headache as pregnancy d.s.	Knowledge and attitudes	All respondents with a complete survey	38·1	39·2	0·32	37·0	41·4	<0·001	-1·1	+2·2
Identified ringing in ears as pregnancy d.s.	Knowledge and attitudes	All respondents with a complete survey	3·9	3·8	0·789	1·5	1·5	1	-2·4	-2·3
Identified dizziness as pregnancy d.s.	Knowledge and attitudes	All respondents with a complete survey	36·2	36·9	0·521	37·1	36·6	0·623	+0·9	-0·3
Identified difficulty urinating as pregnancy d.s.	Knowledge and attitudes	All respondents with a complete survey	1·9	2·2	0·555	1·0	1·1	0·668	-0·9	-1·1
Identified reduced or absent fetal movement as pregnancy d.s.	Knowledge and attitudes	All respondents with a complete survey	5·9	6·1	0·662	4·6	4·5	0·906	-1·3	-1·6
Identified water breaking as pregnancy d.s.	Knowledge and attitudes	All respondents with a complete survey	2·1	2·4	0·415	1·2	1·1	0·779	-0·9	-1·3
Identified fever as pregnancy d.s.	Knowledge and attitudes	All respondents with a complete survey	13·7	13·6	0·907	17·1	19·6	0·002	+3·4	+6·0
Identified swelling of face/hands/feet as pregnancy d.s.	Knowledge and attitudes	All respondents with a complete survey	9·3	9·7	0·489	14·0	16·5	0·001	+4·7	+6·8
**Postnatal care for mother**									
Mother had health checked by professional within 3 days of birth	Practice	All respondents with a complete survey and a child born since 2018-09-01 (after the end of the intervention)	14·6	14·1	0·744	22·9	27·4	0·263	+8·3	+13·3
Mother had health checked by professional within 7 days of birth	Practice	All respondents with a complete survey and a child born since 2018-09-01 (after the end of the intervention)	27·2	27·2	1	43·1	43·8	0·942	+15·9	+16·6

Mother sought medical care for postnatal danger sign	Practice	All respondents with a complete survey and a child born since 2018-09-01 (after the end of the intervention)	51·2	46·6	0·214	47·5	51·3	0·706	-3·7	+4·7
Identifies that mother should receive postnatal medical check-up within 3 days of birth	Knowledge and attitudes	All respondents with a complete survey	9·1	10·0	0·17	16·0	24·4	<0·001	+6·9	+14·4
Identifies that mother should receive postnatal medical check-up within 7 days of birth	Knowledge and attitudes	All respondents with a complete survey	23·9	24·5	0·542	33·8	40·8	<0·001	+9·9	+16·3
Identified heavy vaginal bleeding as postnatal d.s.	Knowledge and attitudes	All respondents with a complete survey	42·4	43·6	0·312	37·6	41·4	<0·001	-4·8	-2·2
Identified fever as postnatal d.s.	Knowledge and attitudes	All respondents with a complete survey	37·4	36·2	0·283	26·1	28·5	0·013	-11·3	-7·7
Identified vaginal discharge as postnatal d.s.	Knowledge and attitudes	All respondents with a complete survey	6·8	6·8	1	3·6	3·7	0·695	-3·2	-3·1
Identified headache as postnatal d.s.	Knowledge and attitudes	All respondents with a complete survey	22·1	23·5	0·141	17·0	19·5	0·002	-5·1	-4·0
Identified convulsions or fits as postnatal d.s.	Knowledge and attitudes	All respondents with a complete survey	1·0	1·1	0·944	0·7	0·8	0·591	-0·3	-0·3
**Postnatal care for newborn**										
Newborn had health checked by professional within 3 days of birth	Practice	All respondents with a complete survey and a child born since 2018-09-01 (after the end of the intervention)	16·6	15·9	0·65	22·4	29·8	0·056	+5·8	+13·9
Newborn had health checked by professional within 7 days of birth	Practice	All respondents with a complete survey and a child born since 2018-09-01 (after the end of the intervention)	29·7	28·4	0·456	43·0	48·1	0·271	+13·3	+19·7
Newborn experienced health problem in first month, care was sought	Practice	All respondents with a complete survey and a child born since 2018-09-01 (after the end of the intervention)	74·5	70·0	0·175	65·5	55·2	0·242	-9·0	-14·8

Oles W, *et al. BMJ Open* 2024; 14:e060784. doi: 10.1136/bmjopen-2022-060784

Identified correct ways to provide newborn care[a]	Knowledge and attitudes	All respondents with a complete survey	4·6	6·1	0·003	2·1	3·5	<0·001	-2·5	-2·6
Identified fever as newborn d.s.	Knowledge and attitudes	All respondents with a complete survey	68·2	66·8	0·201	57·7	56·8	0·379	-10·5	-10·0
Identified diarrhea as newborn d.s.	Knowledge and attitudes	All respondents with a complete survey	48·7	49·2	0·694	32·5	35·3	0·005	-16·2	-13·9
Identified difficulty breathing as newborn d.s.	Knowledge and attitudes	All respondents with a complete survey	35·9	36·3	0·721	9·8	11·0	0·062	-26·1	-25·3
Identified vomiting as newborn d.s.	Knowledge and attitudes	All respondents with a complete survey	27·6	28·2	0·588	11·5	11·8	0·7	-16·1	-16·4
Identified poor appetite as newborn d.s.	Knowledge and attitudes	All respondents with a complete survey	5·5	6·8	0·018	2·2	2·0	0·509	-3·3	-4·8
Identified redness/bleeding around cord as newborn d.s.	Knowledge and attitudes	All respondents with a complete survey	4·9	5·0	0·823	4·4	5·9	0·001	-0·5	+0·9
Identified pus in cord as newborn d.s.	Knowledge and attitudes	All respondents with a complete survey	4·5	5·6	0·02	2·9	4·5	<0·001	-1·6	-1·1
Identified cold skin as newborn d.s.	Knowledge and attitudes	All respondents with a complete survey	1·2	1·7	0·036	0·8	0·6	0·283	-0·4	-1·1
Identified cough as newborn d.s.	Knowledge and attitudes	All respondents with a complete survey	1·6	0·9	0·009	37·5	35·7	0·084	+35·9	+34·8
Identified cold as newborn d.s.	Knowledge and attitudes	All respondents with a complete survey	1·3	1·1	0·679	36·2	32·5	<0·001	+34·9	+31·4
Identified problems latching as newborn d.s.	Knowledge and attitudes	All respondents with a complete survey	0·0	0·0	0·844	2·6	2·7	1	+2·6	+2·7
Identified pneumonia as newborn d.s.	Knowledge and attitudes	All respondents with a complete survey	NA	NA	NA	14·0	14·4	0·62	NA	NA
Did not use harmful substances to treat cord stump	Practice	All respondents with a complete survey and a child born since 2018-09-01 (after the end of the intervention)	84·4	81·7	0·066	44·1	53·2	0·042	-40·3	-28·5
Did not wrap fajero around newborn in first 7 days after birth	Practice	All respondents with a complete survey and a child born since 2018-09-01 (after the end of the intervention)	6·1	5·2	0·376	22·2	43·1	<0·001	+16·1	+37·9
Identified proper cord care methods[b]	Knowledge and attitudes	All respondents with a complete survey	46·0	46·4	0·731	38·7	49·0	<0·001	-7·3	+2·6

Correctly answered proper cord care riddle	Intervention knowledge	All respondents with a complete survey	NA	NA	NA	21·5	39·4	<0·001	NA	NA
Kept newborn wrapped first 7 days after birth	Practice	All respondents with a complete survey and a child born since 2018-09-01 (after the end of the intervention)	98·7	98·0	0·182	97·5	92·9	0·01	-1·2	-5·1
Held newborn skin-to-skin during first month after birth	Practice	All respondents with a complete survey and a child born since 2018-09-01 (after the end of the intervention)	100·0	100·0	NA	31·0	40·5	0·023	-69·0	-59·5
**Breastfeeding**										
Exclusively breastfed child first 6 months	Practice	All respondents with a complete survey and a child born since 2018-09-01 (after the end of the intervention)	57·8	57·9	0·99	56·4	65·9	0·028	-1·4	+8·0
Exclusively breastfed child first 6 months without giving chupón	Practice	All respondents with a complete survey and a child born since 2018-09-01 (after the end of the intervention)	35·1	31·4	0·042	41·7	52·1	0·017	+6·6	+20·7
Breastfed immediately after birth	Practice	All respondents with a complete survey and a child born since 2018-09-01 (after the end of the intervention) who reported a natural birth	86·5	83·8	0·071	82·4	88·9	0·073	-4·1	+5·1
Identifies that newborn should be breastfed immediately after birth	Knowledge and attitudes	All respondents with a complete survey	81·0	81·7	0·469	80·5	82·0	0·096	-0·5	+0·3
Identifies that newborns should only be given breast milk during first 6 months	Knowledge and attitudes	All respondents with a complete survey	80·5	81·2	0·495	81·9	85·4	<0·001	+1·4	+4·2
Believes newborns should not be given chupón during first 6 months	Knowledge and attitudes	All respondents with a complete survey	24·3	22·4	0·048	26·7	44·9	<0·001	+2·4	+22·5

**Paternal involvement**										
Father accompanied mother to clinic for prenatal care visit at least once	Practice	All respondents with a complete survey and a child born since 2018-09-01 (after the end of the intervention) who received prenatal care	52·5	52·8	0·906	58·8	55·9	0·565	+6·3	+3·1
Father accompanied mother to seek medical care for pregnancy danger sign	Practice	All respondents with a complete survey and a child born since 2018-09-01 (after the end of the intervention) who experienced a prenatal care danger sign and sought care	69·7	60·9	0·019	67·7	71·7	0·749	-2·0	+10·8
Father waited at birthplace during labor	Practice	All respondents with a complete survey and a child born since 2018-09-01 (after the end of the intervention)	76·6	76·7	0·987	80·7	73·5	0·05	+4·1	-3·2
Father accompanied mother to seek medical care for postnatal danger sign	Practice	All respondents with a complete survey and a child born since 2018-09-01 (after the end of the intervention) and sought care for postpartum problem	68·8	69·7	0·923	66·1	76·3	0·398	-2·7	+6·6
Father sought medical care for newborn for postnatal danger sign	Practice	All respondents with a complete survey and a child born since 2018-09-01 (after the end of the intervention) and sought care for a postnatal problem	76·2	72·5	0·352	67·5	66·7	1	-8·7	-5·8
Father held child	Practice	All respondents with a complete survey and a child born since 2018-09-01 (after the end of the intervention)	97·3	96·9	0·61	90·1	88·6	0·654	-7·2	-8·3

Oles W, *et al. BMJ Open* 2024; 14:e060784. doi: 10.1136/bmjopen-2022-060784

Father played with child	Practice	All respondents with a complete survey and a child born since 2018-09-01 (after the end of the intervention)	90·6	91·0	0·793	84·7	79·0	0·103	-5·9	-12·0
Father took child to clinic when sick	Practice	All respondents with a complete survey and a child born since 2018-09-01 (after the end of the intervention)	73·2	69·6	0·049	63·9	62·9	0·875	-9·3	-6·7
Believes father should accompany mother to prenatal care visits	Knowledge and attitudes	All respondents with a complete survey	92·3	92·0	0·569	92·4	96·0	<0·001	+0·1	+4·0
Believes father should wait at birth location while mother gives birth	Knowledge and attitudes	All respondents with a complete survey	93·9	94·2	0·522	94·3	94·8	0·387	+0·4	+0·6
Believes father should care for children when sick	Knowledge and attitudes	All respondents with a complete survey	99·3	99·2	0·91	99·1	99·0	0·687	-0·2	-0·2
**Diarrhea management**										
Did not report diarrhea in past 4 weeks	Practice	All respondents with a complete survey	97·6	97·4	0·516	97·5	97·2	0·355	-0·1	-0·2
Used ORT to treat diarrhea in past 4 weeks	Practice	All respondents with a complete survey who had diarrhea in past 4 weeks	61·6	42·9	0·011	41·5	37·8	0·653	-20·1	-5·1
Used appropriate treatment for diarrhea in past 4 weeks[c]	Practice	All respondents with a complete survey who had diarrhea in past 4 weeks	25·7	26·0	1	19·9	19·6	1	-5·8	-6·4
Child did not experience diarrhea past 4 weeks	Practice	All respondents with a complete survey and a new child <= 5 years old reported at wave 3 who had diarrhea in past 4 weeks	84·2	82·3	0·189	89·5	89·5	1	+5·3	+7·2
Child experienced diarrhea past 4 weeks, was given appropriate amount of liquids	Practice	All respondents with a complete survey and a new child <= 5 years old reported at wave 3 who had diarrhea in past 4 weeks	52·4	54·7	0·685	51·1	41·6	0·123	-1·3	-13·1

Oles W, *et al. BMJ Open* 2024; 14:e060784. doi: 10.1136/bmjopen-2022-060784

Child experienced diarrhea past 4 weeks, was given appropriate amount of food	Practice	All respondents with a complete survey and a new child <= 5 years old reported at wave 3 who had diarrhea in past 4 weeks	36·8	36·1	0·958	30·0	21·2	0·113	-6·8	-14·9
Child experienced diarrhea past 4 weeks, was given ORT	Practice	All respondents with a complete survey and a new child <= 5 years old reported at wave 3 who had diarrhea in past 4 weeks	66·3	58·0	0·079	49·2	55·0	0·372	-17·1	-3·0
Child experienced diarrhea past 4 weeks, was given appropriate treatment	Practice	All respondents with a complete survey and a new child <= 5 years old reported at wave 3 who had diarrhea in past 4 weeks	27·3	29·7	0·614	20·3	28·6	0·117	-7·0	-1·1
Child experienced diarrhea past 4 weeks, was given zinc 10-14 days	Practice	All respondents with a complete survey and a new child <= 5 years old reported at wave 3 who had diarrhea in past 4 weeks	1·6	0·6	0·581	1·4	2·0	1	-0·2	+1·4
Identified appropriate diarrhea treatment methods[d]	Knowledge and attitudes	All respondents with a complete survey	41·3	41·2	0·963	35·4	45·0	<0·001	-5·9	+3·8
Identified zinc 10-14 days as diarrhea treatment	Knowledge and attitudes	All respondents with a complete survey	4·1	4·2	0·727	7·3	8·3	0·084	+3·2	+4·1
Identified zinc supplement as way to prevent diarrhea	Knowledge and attitudes	All respondents with a complete survey	45·4	45·5	0·994	67·2	73·5	<0·001	+21·8	+28·0
Identified breastfeeding as way to prevent diarrhea	Knowledge and attitudes	All respondents with a complete survey	26·2	26·0	0·837	65·3	67·5	0·028	+39·1	+41·5
Correctly answered diarrhea treatment with zinc riddle	Intervention knowledge	All respondents with a complete survey	NA	NA	NA	18·9	41·0	<0·001	NA	NA
**Respiratory illness**										
Did not report respiratory illness (coughing) for 2 weeks	Practice	All respondents with a complete survey	79·2	79·6	0·633	85·5	83·5	0·011	+6·3	+3·9

Child did not have cough past 4 weeks	Practice	All respondents with a complete survey	68·0	63·2	0·008	79·2	78·6	0·741	+11·2	+15·4
Child had cough past 4 weeks, care was sought	Practice	All respondents with a complete survey and a new child <= 5 years old reported at wave 3 who had an illness with a cough in past 4 weeks	4·1	2·9	0·447	34·3	33·5	0·905	+30·2	+30·6
Identified vaccination as way to prevent respiratory illness	Knowledge and attitudes	All respondents with a complete survey	50·7	50·8	0·939	83·9	84·8	0·236	+33·2	+34·0
Identified breastfeeding as way to prevent respiratory illness	Knowledge and attitudes	All respondents with a complete survey	34·0	33·6	0·728	70·9	72·0	0·262	+36·9	+38·4
Identified fever as d.s. for children with respiratory illness	Knowledge and attitudes	All respondents with a complete survey	81·6	80·8	0·392	94·1	95·3	0·022	+12·5	+14·5
Identified difficulty breathing as d.s. for children with respiratory illness	Knowledge and attitudes	All respondents with a complete survey	88·6	88·3	0·691	96·7	97·6	0·018	+8·1	+9·3
Identified rapid breathing as d.s. for children with respiratory illness	Knowledge and attitudes	All respondents with a complete survey	85·0	85·2	0·853	97·5	98·1	0·079	+12·5	+12·9
**Reproductive health**										
Reported ever using birth control to delay or avoid pregnancy	Practice	All respondents with a complete survey	33·9	31·7	0·056	38·2	40·0	0·082	+4·3	+8·3
Reported currently using birth control	Practice	All respondents with a complete survey who are not pregnant and have ever used birth control	56·0	57·2	0·584	54·7	59·4	0·007	-1·3	+2·2
Believes woman should be at least 18 years of age to have her first child	Knowledge and attitudes	All respondents with a complete survey	98·4	97·9	0·112	98·3	99·0	0·02	-0·1	+1·1
**Gender/reproductive norms**										
Birth location chosen either jointly or by woman	Practice	All respondents with a complete survey and a child born since 2018-09-01 (after the end of the intervention)	92·4	91·8	0·593	84·6	85·8	0·785	-7·8	-6·0

Results based on final sample of N=10263 (household targeted n=3238, household not targeted n=7025). d.s.=Danger sign, NA=Not applicable, ORT=Oral rehydration therapy. Proportions with NA could not be assessed because the outcome was not part of the baseline instrument. Proportion comparisons against a null hypothesis that there is no difference in outcomes between those targeted and untargeted were performed using a two-sided t-test.

[a] ‘Identified correct ways to provide newborn care’ outcome was coded as correct if the respondent identified holding baby skin to skin, getting their health checked by a professional, immediately putting them to the breast, or making sure their diaper is clean and dry, and did not identify giving purgante, wrapping a fajero, giving a chupon, or bathing them in water right away.
[b] ‘Identified proper cord care’ outcome was coded as correct if respondent identified applying alcohol, applying iodine, keeping cord clean/dry, or applying peroxide, and did not identify applying oil, applying coffee, applying quina water, wrapping a fajero, applying powder, burning, applying ashes, applying a clamp, or applying thiomersal.
[c] ‘Used appropriate treatment for diarrhea in past 4 weeks’ outcome was coded as correct if respondent did any of the following: antibiotic, zinc, give extra food, give extra liquid, and did not do any of the following: anti-diarrhea medication, home remedy, laxative, deworming medication, chupon, massage, stop eating foods, stop taking liquids.
[d] ‘Identified appropriate diarrhea treatment methods’ outcome was coded as correct if respondent identified any of the following: antibiotic, zinc, ORS or Litrosol, give extra food, give extra liquid, get medical attention, and did not identify any of the following: anti-diarrhea medication, laxative, deworming medication, home remedy, chupon, massage, stop giving foods, stop giving liquids, or do nothing.

Table S10. Analysis of differences in attrition between baseline and endline surveys

	Lost to follow-up (n=6038)	Completed endline survey (n=10263)	Test statistic	p value
Target status (households), *n (%)*				
Not targeted	3971 (66%)	7025 (68%)	0·87	0·351
Targeted	2067 (34%)	3238 (32%)		
Age, *mean (SD)*	28·7 (15·9)	35·0 (17·5)	-21·49	<0·001
Sex, *n (%)*				
Female	2382 (39%)	6318 (62%)	746·79	<0·001
Male	3656 (61%)	3945 (38%)		
Education, *n (%)*				
Less than primary	2625 (57%)	5964 (64%)	67·02	<0·001
Primary or greater	1985 (43%)	3339 (36%)		
Marital status, *n (%)*				
Single	2719 (45%)	3186 (31%)	335·74	<0·001
Married or Civil Union	3056 (51%)	6348 (62%)		
Separated/Divorced/Widowed	263 (4%)	729 (7%)		
Indigenous status, *n (%)*				
No	4168 (90%)	8061 (87%)	40·36	<0·001
Yes, Maya Chorti	438 (10%)	1229 (13%)		
Yes, other indigenous group	3 (<1%)	6 (<1%)		
Self-rated physical health, *mean (SD)*	3·1 (1·1)	3·3 (1·0)	-8·99	<0·001
Self-rated mental health, *mean (SD)*	3·0 (1·1)	3·2 (1·1)	-8·70	<0·001
Household wealth index, *n (%)*				
Quintile 1	940 (17%)	1674 (17%)	4·10	0·393
Quintile 2	1094 (18%)	1931 (19%)		
Quintile 3	1191 (20%)	1980 (19%)		
Quintile 4	1264 (21%)	2194 (22%)		
Quintile 5	1426 (24%)	2336 (23%)		

Analysis based on subgroup of households/respondents who were randomized in the trial (intention to treat analysis, n=16301). Pearson's chi-squared test was used to assess differences in target status, sex, education, marital status, indigenous status, and household wealth index. Welch two-sample t-test was used to assess age, self-rated physical health, and self-rated mental health.

References

1. DeSalvo KB, Bloser N, Reynolds K, et al. Mortality prediction with a single general self-rated health question: A meta-analysis. Journal of General Internal Medicine 2006;21:267–75. doi: 10.1111/j.1525-1497.2005.00291.x

2. Kroenke K, Spitzer RL, Williams JB. The Patient Health Questionnaire-2: Validity of a Two-Item Depression Screener. Medical Care 2003;41:1284–92. doi: 10.1097/01.MLR.0000093487.78664.3C

3. Bush K, Kivlahan DR, McDonell MB, et al. The AUDIT alcohol consumption questions (AUDIT-C): an effective brief screening test for problem drinking. Arch Intern Med 1998;158(16):1789–95. doi: 10.1001/archinte.158.16.1789.

4. Pérez-Escamilla R, Dessalines M, Finnigan M, et al. Household food insecurity is associated with childhood malaria in rural Haiti. Journal of Nutrition 2009;139(11):2132–8. doi: 10.3945/jn.109.108852

5. Baird S, Bohren JA, McIntosh C, *et al*. Optimal Design of Experiments in the Presence of Interference. The Review of Economics and Statistics 2018;100:844–60. doi: 10.1162/rest_a_00716

6. Hudgens MG, Halloran ME. Toward Causal Inference With Interference. Journal of the American Statistical Association 2008;103:832–42. doi: 10.1198/016214508000000292
