# Supplementary material for: Maternal and child health intervention to promote behaviour change: a population-level cluster-randomised controlled trial in Honduras
Source: BMJ Open. 2024 Jun 10;14(6):e060784. doi: 10.1136/bmjopen-2022-060784 (PMC11168147; doi:10.1136/bmjopen-2022-060784)
Supplement: Author's manuscript [file bmjopen-2022-060784.draft_revisions.pdf]

BMJ Open

BMJ Open is committed to open peer review. As part of this commitment we make the peer review history of every article we publish publicly available.

When an article is published we post the peer reviewers' comments and the authors' responses online. We also post the versions of the paper that were used during peer review. These are the versions that the peer review comments apply to.

The versions of the paper that follow are the versions that were submitted during the peer review process. They are not the versions of record or the final published versions. They should not be cited or distributed as the published version of this manuscript.

BMJ Open is an open access journal and the full, final, typeset and author-corrected version of record of the manuscript is available on our site with no access controls, subscription charges or pay-per-view fees (http://bmjopen.bmj.com).

If you have any questions on BMJ Open's open peer review process please email info.bmjopen@bmj.com

Maternal and child health intervention to promote behavior change: a population-level cluster-randomized controlled trial in Honduras

Journal:	*BMJ Open*
Manuscript ID	bmjopen-2022-060784
Article Type:	Original research
Date Submitted by the Author:	09-Apr-2022

Complete List of Authors:	Alexander, Marcus; Yale University, Yale Institute for Network Science<br>Oles, William; Yale University<br>Negron , Rennie; Yale University, Yale Institute for Network Science<br>Nelson , Jennifer ; Inter-American Development Bank<br>Iriarte, Emma; Salud Mesoamérica 2015 / Inter-American Development Bank<br>Airoldi, E; Temple University Fox School of Business and Management<br>Christakis, Nicholas; Yale University, Yale Institute for Network Science; Yale University, Department of Sociology and Medicine<br>Forastiere, Laura; Yale University, Yale Institute for Network Science; Yale University, Department of Biostatistics

Keywords:	Public health < INFECTIOUS DISEASES, Community child health < PAEDIATRICS, STATISTICS & RESEARCH METHODS

SCHOLARONE™
Manuscripts

I, the Submitting Author has the right to grant and does grant on behalf of all authors of the Work (as defined in the below author licence), an exclusive licence and/or a non-exclusive licence for contributions from authors who are: i) UK Crown employees; ii) where BMJ has agreed a CC-BY licence shall apply, and/or iii) in accordance with the terms applicable for US Federal Government officers or employees acting as part of their official duties; on a worldwide, perpetual, irrevocable, royalty-free basis to BMJ Publishing Group Ltd ("BMJ") its licensees and where the relevant Journal is co-owned by BMJ to the co-owners of the Journal, to publish the Work in this journal and any other BMJ products and to exploit all rights, as set out in our licence.

The Submitting Author accepts and understands that any supply made under these terms is made by BMJ to the Submitting Author unless you are acting as an employee on behalf of your employer or a postgraduate student of an affiliated institution which is paying any applicable article publishing charge ("APC") for Open Access articles. Where the Submitting Author wishes to make the Work available on an Open Access basis (and intends to pay the relevant APC), the terms of reuse of such Open Access shall be governed by a Creative Commons licence – details of these licences and which Creative Commons licence will apply to this Work are set out in our licence referred to above.

Other than as permitted in any relevant BMJ Author's Self Archiving Policies, I confirm this Work has not been accepted for publication elsewhere, is not being considered for publication elsewhere and does not duplicate material already published. I confirm all authors consent to publication of this Work and authorise the granting of this licence.

For peer review only

Maternal and child health intervention to promote behavior change: a population-level cluster-randomized controlled trial in Honduras

Marcus Alexander,[1] William Oles,[1] Rennie Negron,[1] Jennifer Nelson,[2] Emma Iriarte,[2] E. M. Airoldi,[3] Nicholas A. Christakis,[1,4] Laura Forastiere[1,5]*

[1]Yale Institute for Network Science, Yale University, New Haven, CT, USA;
[2]Inter-American Development Bank, Washington, DC, USA;
[3]Fox School of Business, Temple University, Philadelphia, PA, USA;
[4]Departments of Sociology and Medicine, Yale University, New Haven, CT, USA; and
[5]Department of Biostatistics, Yale School of Public Health, Yale University, New Haven, CT, USA (L Forastiere PhD)

*Correspondence to: Professor Laura Forastiere, Department of Biostatistics, Yale School of Public Health, 350 George St, New Haven, CT, laura.forastiere@yale.edu

For peer review only

Abstract

Objectives:
We assessed the efficacy of a sustained educational intervention to change behaviors with respect to a variety of outcomes across the pregnancy and infancy timeline.

Setting:
We conducted a multi-arm cluster-randomized controlled trial in a population of 16,301 people in 5,633 households in 99 villages in the Copán region of Honduras, from October, 2015, to December, 2019.

Participants:
All village residents 12 and older were eligible, 93% of the population participated in the photographic census, 13,881 of whom completed baseline surveys and 10,263 of whom completed the endline survey.

Intervention:
A household-based counseling intervention was administered for 22 months to improve practices, knowledge, and attitudes related to maternal, neonatal, and child health.

Primary and secondary outcome measures:
Primary outcomes were prenatal care, facility births, postnatal care for mother and newborn, exclusive breastfeeding, parental involvement, treatment of diarrhea and respiratory illness, reproductive health, and gender/reproductive norms. Secondary outcomes were knowledge and attitudes related to the primary outcomes.

Results
Parents targeted for the intervention were 16·4% (95% CI 3·1%–29·8%, p=0·016) more likely to have their newborn's health checked by a professional in a health facility within three days of birth; 19·6% (95% CI 4·2%–35·1%, p=0·013) more likely to not wrap a fajero around the umbilical cord in the first week after birth; and 8·9% (95% CI 0·3%–17·5%, p=0·043) more likely report that the mother breastfed immediately after birth. Changes in knowledge and attitudes related to these primary outcomes were also observed. Significant spillover effects were estimated among untreated individuals as well (e.g., related to fathers holding their child and treatment of diarrheal illness).

Conclusion
A sustained counseling intervention delivered in the home setting by community health workers can meaningfully change practices, knowledge, and attitudes related to proper newborn care following birth, including professional care-seeking, umbilical cord care, and breastfeeding.

Trial registration:
ClinicalTrials.gov, NCT02694679.

For peer review only - http://bmjopen.bmj.com/site/about/guidelines.xhtml

Strengths and limitations of this study

- Two-stage randomization and different treatment dosages allowed estimation of pooled spillover effects, a feature uncommon to other large-scale interventions.
- High-quality data acquisition over four years using a modified version of Timed and Targeted Counseling methodology included dialogue counseling methods and interactive story-telling to assess current needs and practices of intervention recipients and negotiate on progressive improvement.
- A wider array of outcomes was collected than other trials of its kind, determining the impact of the intervention on various points of the pregnancy and infancy timeline.
- Limitations are that the study area was subject to co-interventions; that the intervention modules were delivered at different times and frequencies, depending on household composition; and that the outcomes were assessed using self-report.

For peer review only

Introduction

Despite significant global strides in many measures of maternal and under-5 child health over the past 30 years, the burden of maternal and child morbidity and mortality remains large, particularly in resource-limited settings across Sub-Saharan Africa and Latin America. Globally, neonatal deaths make up nearly 50% of all under-5 child deaths,[1] and preterm birth complications, pneumonia, and intrapartum-related complications account for the majority of these deaths.[2] These causes suggest that poor maternal outcomes often underlie poor infant and child outcomes; consequently, large-scale interventions to improve resources, knowledge, and practices at every point in the pregnancy and infancy timeline have become a central part of the global health agenda. Interventions targeting maternal and child health outcomes are largely founded on evidence-based WHO guidelines, which emphasize birth preparedness, complication readiness, and care-seeking for the mother, as well as immediate stimulation, skin-to-skin contact, immediate and exclusive breastfeeding, umbilical cord care, thermal care and medical check-ups for the newborn, among others.[3,4]

Although many neonatal, child, and maternal deaths can be prevented through clinical services, emerging evidence has shown that reductions in poor health outcomes in these areas are possible by complementing them with low-cost interventions in the home and community setting.[5,6] This branch of intervention packages focuses on demand-side conditions, building on the idea that improvement in behaviors related to maternal and child health outcomes not only depends on the accessibility or provision of health services, but also on the community-level demand for services and practices.

For peer review only

Behaviors which affect maternal and child health outcomes are often socially reinforced or otherwise influenced by community norms and therefore difficult to change, making the on-ground implementation of interventions increasingly important. There is a large body of evidence that points to the efficacy of counseling interventions,[7-11] which often utilize interpersonal interactions with community health workers during home visits and may be implemented on their own or as one of many domains in a multidimensional intervention package.

Despite the considerable progress that Honduras has made towards its population health goals, it still lags behind many other low-to-middle-income countries in Latin America. In Honduras in 2019, the under-5 mortality rate was 16.8 per 1000 live births while the neonatal mortality rate was for 9.2 per 1000 live births.[6] Of neonatal deaths, 40% are attributable to premature labor, while another 40% are attributable to asphyxia or infection.[12] According to data from the most recent Honduras Demographic and Health Survey, nearly one quarter of Honduran girls aged 15-19 were either pregnant or a mother,[13] and in 2017, the World Health Organization estimated that maternal mortality accounted for 3% of all deaths among women of reproductive age in Honduras.[14] Nationwide, 74% of live births occur in a medical facility,[15] while 57% of live births occur in rural regions which likely face higher barriers to perinatal care resource access, given that Honduras already has the lowest physician density in Latin America.[16,17]

For peer review only - http://bmjopen.bmj.com/site/about/guidelines.xhtml

Few if any studies have combined an at-scale counseling intervention with randomized assessment methods in Latin America. Here, we completed a randomized trial in the western highlands of Honduras in which households received a two-year intervention package that consisted of household visits by community health workers (CHWs). CHWs were trained to use validated counseling methods to promote practices, knowledges, and attitudes related to desired maternal and child health outcomes. We assessed a wide range of outcomes across the pregnancy and infancy timeline that aligned with the intervention counseling topics, including prenatal care, facility-based births, postnatal care for mother and child, danger signs and care-seeking, breastfeeding, paternal involvement, diarrhea and respiratory illness prevention and management in children 5 and under, and reproductive health. Pertinently, the underlying framework of the intervention here also differed from similar efforts in other settings in that it targeted the broader community in addition to parents themselves.

The aim of our randomized assessment is thus to evaluate the effects of a large-scale, household-based counseling intervention on outcomes related to maternal, neonatal, and child health in rural villages in Honduras.

Methods

Study design and participants

We conducted a multi-arm cluster-randomized controlled trial in the Department of Copán in western Honduras comprising the municipalities of Copán Ruinas, Santa Rita, Cabanas, and San Jeronimo. Copán is a predominantly rural, mountainous, coffee-

For peer review only - http://bmjopen.bmj.com/site/about/guidelines.xhtml

growing region and is characterized by high rates of neonatal and maternal morbidity and mortality, with many rural villages facing barriers to healthcare which often leave perinatal care insufficient or unsafe.[12,16] The trial was a partnership between the Yale Human Nature Lab (HNL), the Inter-American Development Bank (IDB), and the Ministry of Health of Honduras, with implementing partners World Vision Honduras and Dimagi. Of the 238 villages in this area, 176 were selected for a parent trial evaluating a novel social network targeting technique. Factors such as population size, geographic diversity, accessibility, and safety were considered when selecting villages, and our selection process and profiling of the study area is described further in our study protocol, which has been published elsewhere.[17] Of these 176 villages, 99 form part of this assessment (we excluded villages in which subjects were not assigned to treatment at random, but rather via an alternative, network-based assignment mechanism irrelevant to our objective here).

All individuals aged 12 years or older and who lived in a study village were eligible to participate. We conducted a complete photographic census among individuals who agreed to enroll in the study which covered 93% of the eligible population. Any household with at least one eligible individual who agreed to enroll in the study and who completed the baseline survey was eligible for randomization.

Ethics Approval

All participants provided informed consent at the time of data collection before randomization. Ethics approval was obtained from the Institutional Review Board

For peer review only - http://bmjopen.bmj.com/site/about/guidelines.xhtml

(Protocol #1506016012) at Yale University in New Haven, Connecticut, USA and the Ministry of Health of Honduras.

Randomization and masking

The trial used a two-stage factorial design with a first-stage randomization at the village level and a second-stage randomization at the household level. In the first stage, we randomly assigned villages to a dosage, or proportion of households targeted for intervention per village (0, 0.05, 0.1, 0.2, 0.3, 0.5, 0.75, 1). In the second stage, households were randomly assigned to the intervention according to the village's dosage. We used a covariate-constrained randomization procedure than ensured balance at the village and household levels between arms. Our full randomization procedure is described in our published protocol.[17]

The current randomized assessment focuses on the 99 villages that were assigned to the random targeting strategy with a proportion of targeted households greater than 0 or the control arm with a proportion equal to 0. In other words, we had two kinds of 'control' subjects: 1) untreated people in untreated villages, and 2) untreated people in treated villages. This is important, because, when assessing whether subjects respond to receiving an educational intervention, one should adjust for whether, by chance, other members of their village also got the same intervention, thereby potentially reinforcing the impact of the educational intervention (we note that this important detail of possible reinforcement of treatment response because of treatment of co-villagers is typically overlooked in most field trials of such interventions). Households in the 22 villages with

For peer review only - http://bmjopen.bmj.com/site/about/guidelines.xhtml

a proportion of households targeted equal to 0 did not receive any intervention and were used as our pure control, or comparison, villages.

The intervention assessment reported here establishes if there were changes in practices, knowledge, and attitudes related to target outcomes among those actually receiving the intervention. Due to the nature of the intervention, masking of participants and community health workers, termed Community Change Agents (CCAs) in the current study, who delivered the intervention was not possible. However, both CCAs and surveyors were blinded to the methods used to select the intervention households assigned to them.

Procedures

We and our local partners introduced the project to village leaders, secured local approvals, and managed local implementation of the study and intervention. CCAs delivered up to 22 1–2 hour counseling sessions across 15 modules to targeted households at monthly intervals between November, 2016, and August, 2018. The intervention was designed by World Vision Honduras (who also hired, paid and trained CCAs) and was named Proyecto Redes: Con Amor y Cuidados Madres y Bebés Sanos (With Love and Care, Healthy Moms and Babies).

CCAs made home visits and spoke to families regarding several health topics tailored to the family's current circumstances and based on a modified version of the Timed and Targeted Counseling (ttC) methodology, which has been implemented in 20 countries

For peer review only

For peer review only - http://bmjopen.bmj.com/site/about/guidelines.xhtml

worldwide by World Vision.[18] Additionally, the behavior change communication strategy was designed with the P Process tool, which uses narrative and negotiation to reach agreements with families to try new practices and has been used for more than 30 years for planning health-communication programs.[19] The educational modules delivered during home visits were designed to address maternal, neonatal, and child health, and included topics such as preparation of birth plan, facility-based birth, mother and newborn care, and folic acid importance (see appendix for full list of educational modules). Sessions were also designed to include discussion of relevant regional practices that were identified during formative work in our pilot study as being potentially dangerous to newborns. Two such practices were the use of chupones during feeding––which are cloth materials dampened with saliva, honey, or plant waters—and the placement of a fajero, or an abdominal cloth wrap, around a newborn's umbilical cord site.[17,20]

CCAs used tablets during intervention delivery, which served as a visual and audio aid for educational materials (i.e., families could see stories in video and listen to songs/riddles); as a prompt for CCAs to standardize intervention implementation; and as a medium to collect data.[21] Intervention delivery differed from other community-based approaches in that it was randomized to any household regardless of household composition (not just delivered to expectant mothers); it was comprised of long home visits and did not include additional outside messaging (i.e., pamphlets, radio messages); and it did not include the use of community action groups. However, it should be noted that both intervention and control villages may also have received other

For peer review only - http://bmjopen.bmj.com/site/about/guidelines.xhtml

interventions simultaneously, including both traditional behavior change interventions through the Government of Honduras and supply-side interventions through IDB and the Salud Mesoamerica Initiative.[22]

We developed a structured survey instrument comprised of both validated scales and internally developed items based on extensive literature review, expert consultation, input from the Ministry of Health of Honduras, and qualitative research with local residents and village leaders. Our survey instrument was primarily designed to capture target outcomes related to maternal, neonatal, and child health, in addition to demographic information. Upon study enrollment, participants completed a photographic census and baseline survey from June, 2015, to June, 2016, using our publicly-available social network data collection software “Trellis”.[23] An additional photographic census was conducted from January, 2019, to December, 2019, at which point participants also completed the endline survey. For more details on our pilot work and field operations see the study’s published protocol.[17] No important changes to methods were made after trial commencement.

For peer review only

Outcomes

The primary outcomes can be grouped into the following practices: (1) prenatal care for the mother, including folic acid use, creating a birth plan, and receipt of prenatal care in first trimester; (2) giving birth in a health facility; (3) care-seeking for danger signs experienced by both mother and newborn; (4) postnatal care for mother, including preventative check-ups with health professionals; (5) postnatal care for newborn,

For peer review only - http://bmjopen.bmj.com/site/about/guidelines.xhtml

including preventative check-ups with health professionals, proper thermal care, and proper cord care (including withholding use of fajero); (6) immediate and exclusive breastfeeding for infants under 6 months (including withholding use of chupones); (7) paternal involvement in pregnancy and child care; (8) proper treatment of diarrhea and respiratory illness in children 5 and under; (9) reproductive health (including contraceptive use); and (10) gender/reproductive norms (see appendix for full list of outcomes). Secondary outcomes were knowledge or attitudes related to the primary outcomes. All outcomes were measured via our endline survey instrument. The inclusion of certain responses to our survey questions was determined by eligibility encoded into the survey design, which was termed “denominator” and can be found in the appendix for each outcome (appendix, table S1). For example, only mothers and fathers who had a child since the end of the intervention (September 1, 2018) were eligible to have a response included for the endline survey question about whether their child was exclusively breastfed for the first 6 months.

Statistical analysis

Given that our intervention was designed to be delivered at the household-level and that its behavior change strategies are embedded in ideas of addressing community norms, we cannot rule out the presence of between-participant interference within villages. In particular, we assume that there could be interference within villages but not between villages (partial interference), and that a respondent’s outcome may be affected by the proportion of treated household in the village in addition to its own treatment (stratified interference).[24,25] This assumption is substantiated by an analysis in prior work in a

For peer review only

different part of Honduras that showed enhanced adoption of a nutritional intervention consistent with social magnification.[26]

In this analysis, we use a two-stage randomization design (in which we randomized village clusters to a treatment arm and then further randomized households to receive the intervention) in order to estimate the total effect of the intervention on the treated (the effect of receiving the treatment in an intervention village compared to a control village); the direct effects on the treated (which are effects attributable to receiving the intervention after adjusting for spillover effects); and finally the spillover effects on the untreated who lived in the treated villages and may have been affected by others who received the intervention.[24] We mainly focus on the first effect.

Under an intent-to-treat framework, we used multivariate logistic regression fitted to individual-level data for analysis of binary outcomes. We pooled responses across all treatment arms to obtain an average effect across all village-level treatment dosages, adding sampling weights to adjust for differential probability of treatment assignment. Clustered heteroskedasticity-robust standard errors that allowed for intragroup correlation were estimated at the level of villages, which was the unit of randomization in the first stage. Further information about our analysis strategy can be found in the appendix. Significance was defined as $p<0.05$. All analyses were performed using R version 4.0.4 and Stata version 13. This trial is registered at ClinicalTrials.gov, NCT02694679.

For peer review only - http://bmjopen.bmj.com/site/about/guidelines.xhtml

Patient and Public Involvement

Community leaders of each of the villages were involved as partners in this study, and both the intervention team and the survey data collection team worked with local communities at each step of the study design process to ensure feasibility and incorporate feedback obtained through focus groups, interviews, pilot surveys, and other outreach.

Results

Trial participants 12 and older were recruited and completed a baseline survey between June, 2015, and June, 2016. A total of 16,301 individuals across 5,633 households were randomized, 13,881 of whom completed baseline surveys. 10,263 individuals completed the endline survey between January, 2019, and December, 2019, approximately 24 months after intervention delivery began (of 6,038 individuals lost to endline, 4% were lost to death, 50% to out-migration, 12% to refusal to continue in the study, 32% due to not being reached, and 2% to other reasons). A total of 77 villages with 4,410 households were randomized to the random targeting strategy and 22 villages with 1,223 households were randomized to the control arm with no treated households. Among the 77 villages assigned to the random targeting strategy, 11 villages were randomly assigned to each treatment dosage, or proportion of households targeted (figure 1, see appendix figure S1 for parent trial). Across all treatment dosages, a total of 1,815 households with 5,305 individuals were randomized to receive the intervention. Villages had between 48 and 571 participants each, and households

For peer review only

assigned to treatment received the intervention's educational household visits between November, 2016, and August, 2018.

At baseline, 8,700 out of 16,301 (53%) participants were women. The mean age was 32·9 years (SD 17·2); 9,404 (58%) respondents were married or in a civil union; and 8,589 out of 13,881 (62%) participants who completed baseline surveys had less than a primary education. Of the parents that completed an endline survey, 630 reported having a child after the intervention delivery period (September 1, 2018) and 1,133 reported having a child that was five or under at baseline. Descriptive statistics of the sample arranged by treatment arm are reported in table 1. Village and household characteristics were balanced across treatment arms at baseline.

For peer review only

Table 1. Baseline characteristics of the intention-to-treat population in the pure control and random-assignment arms

	Pure control group (n=3600)	Random-assignment group (n=12701)	
		Within-cluster control (n=7396)	Targeted (n=5305)
Household characteristics			
**Wealth index**			
Quintile 1	524 (15%)	1194 (17%)	896 (17%)
Quintile 2	558 (16%)	1394 (19%)	1073 (21%)
Quintile 3	592 (17%)	1596 (22%)	983 (19%)
Quintile 4	819 (22%)	1487 (21%)	1152 (22%)
Quintile 5	1080 (30%)	1566 (21%)	1116 (21%)
**Households**	1223	2595	1815
**Household size***	4·28 (2·1)	4·35 (2·1)	4·52 (2·2)
Child characteristics			
**Youngest child's age, years***	6·04 (5·2)	5·44 (4·8)	5·51 (4·9)
**Past month illness (child 5 or under)***			
Respiratory illness	281 (30%)	674 (33%)	527 (36%)
Diarrheal illness	124 (13%)	338 (16%)	251 (17%)
Respondent characteristics			
**Gender**			
Female	1908 (53%)	3964 (54%)	2828 (53%)
Male	1692 (47%)	3432 (46%)	2477 (47%)
**Age**	33·2 (17·4)	32·8 (17·1)	32·8 (17·2)
**Education***			
Less than primary	1845 (60%)	3907 (62%)	2836 (63%)
Primary or greater	1206 (40%)	2438 (38%)	1680 (37%)
**Marital status**			
Single	1274 (35%)	2672 (36%)	1959 (37%)
Married or Civil Union	2120 (59%)	4254 (58%)	3030 (57%)
Separated/Divorced/Widowed	206 (6%)	470 (6%)	316 (6%)
**Indigenous status***			
No	2807 (92%)	5489 (87%)	3932 (87%)
Yes, Maya Chorti	243 (8%)	851 (13%)	573 (13%)
Yes, other indigenous group	1 (0%)	4 (0%)	2 (0%)
**Health***			
Self-rated physical health, 1 (excellent)–5 (poor)†	3·21 (1·1)	3·25 (1·1)	3·24 (1·1)
Self-rated mental health, 1 (excellent)–5 (poor)†	3·08 (1·1)	3·12 (1·1)	3·14 (1·1)

Data are n, n (%), or mean (SD). *Information only available from respondents who completed baseline survey (n=13881). †Health questions from single item assessing general self-rated health (SF-1), DeSalvo KB, Bloser N, Reynolds K, He J, Muntner P. Mortality prediction with a single general self-rated health question. A meta-analysis. *Journal of general internal medicine* 2006; **21**(3).

For peer review only - http://bmjopen.bmj.com/site/about/guidelines.xhtml

oSignificant total effects of the intervention on all primary and secondary outcomes are displayed in figure 2. Direct, indirect, and total effects estimated from outcomes at endline as well as baseline levels for each outcome are reported in Table S1 (appendix). We focus here on total effects of the intervention pooled across all dosages, which account for both the direct effect of the intervention and any spillover effects across each village (i.e., the effect of having other treated individuals in the same village upon a person who is treated with the intervention). Although all three effects are estimated, the total effects are emphasized as the best average estimate of the intervention's impact in the study population and as the metric most analogous to treatment effects reported from other comparable educational intervention trials.

Compared to parents of children born after the intervention period that didn't receive the intervention, parents of children who received it showed significant improvements in primary outcome practices related to postnatal care, proper umbilical cord care, and breastfeeding. Specifically, the total effect of the intervention led to a 16·4% (95% CI 3·1%–29·8%, p=0·016) increase in the probability that parents had their newborn's health checked by a professional in a health facility within three days of birth; a 19·6% (95% CI 4·2%–35·1%, p=0·013) increase in the probability that parents did not wrap a fajero around the umbilical cord in the first week after birth; and a 8·9% (95% CI 0·3%–17·5%, p=0·043) increase in the probability that the mother breastfed immediately after birth (figure 2; Table S1, appendix). Among parents of children born after the intervention period, we found no significant total effect of the intervention on practices related to preparing a birth plan, seeking care for pregnancy danger signs, facility based

For peer review only - http://bmjopen.bmj.com/site/about/guidelines.xhtml

birth, mothers seeking postnatal check-up or care for postnatal danger signs, keeping the newborn wrapped or holding them skin-to-skin after birth, exclusively breastfeeding for six months, or father involvement in newborn care. Additionally, we found no significant total effects on diarrhea or respiratory illness treatment in children five and under, folic acid use among women 15 and over, or contraceptive use among all respondents.

In addition to the changes in actual behaviors related to maternal and child health, we also assessed changes in the knowledge and attitudes related to those behaviors as secondary outcomes. Changes in knowledge or attitudes may precede changes in practices, and we analyzed responses of all participants regardless of whether they had a child since the intervention. We noted improvements in knowledge or attitudes related to the significant practices in the areas of postnatal care, proper umbilical cord care, and breastfeeding among the treated. Specifically, the total effect of the intervention was a 2·7% (95% CI 1·0%–4·3%, p=0·0014) increase in the probability that a participant identified correct ways to provide newborn care, which included getting their health checked by a medical professional; a 9·1% (95% CI 4·0%–14·2%, p=0·0005) increase in the probability that a participant identified proper cord care methods, which included not wrapping a fajero; and a 14·0% (95% CI 8·4%–19·6%, p<0·0001) increase in the probability a participant believed newborns should not be given chupones when breastfeeding in the first six months (figure 2; Table S1, appendix).

For peer review only - http://bmjopen.bmj.com/site/about/guidelines.xhtml

Additionally, we found significant total effects for knowledge and attitudes related to folic acid use before pregnancy; methods of savings as part of the birth plan; select pregnancy danger signs (i.e., fever, swelling of hands/face/feet); fathers accompanying mothers to prenatal care visits; mothers receiving postnatal check-ups within three days of birth; select postnatal danger signs for mother (i.e., heavy vaginal bleeding, fever); identifying appropriate diarrhea treatment methods including correct use of zinc; and delaying pregnancy until 18 years or older (figure 2; Table S1, appendix). We found no significant total effect of the intervention on knowledge or attitudes related to seeking prenatal care in the first trimester; facility-based birth; the majority of the pregnancy danger signs; the majority of the postnatal and newborn danger signs; immediate breastfeeding; father waiting at birth location or caring for children when they are sick; diarrheal illness prevention; or respiratory illness prevention and danger signs (Table S1, appendix).

No adverse events associated with the intervention were found. We performed additional analyses as a check for robustness of our results, which included looking exclusively at the response of the mothers, adjusting for demographic characteristics of respondents, adjusting for baseline behaviors, and excluding those who moved between treatment arms during the study (Table S2–Table S7, appendix).

Finally, we note that two outcomes were associated with significant spillover effects on those who were untreated, which are effects of having others in one's village receive the intervention: father holding the child, and child with past-month diarrheal illness being

For peer review only - http://bmjopen.bmj.com/site/about/guidelines.xhtml

treated with zinc (Table S1, appendix). Our study design, which included variation in the fraction of people treated per village and the presence of a pure control group of wholly untreated villages allowed us to perform such an analysis. Further investigation of how spillover effects may depend on treatment dosage and effects specific to a village's social network structure are the subject of other work.

Quality assessment of intervention delivery

Monitoring data on session implementation showed that the intervention was delivered as intended, with up to 22 monthly sessions planned per intervention village. Of the 1,815 households initially targeted for intervention at baseline, 1,225 (67%) households received modules for all 15 intervention topics as designed. Of the targeted households that completed endline surveys, the average number of sessions delivered per household was 15·2 (SD 7·4), with an average group size of 1·1 (SD 0·7) in attendance. Of the parents of children born after the intervention period who completed endline surveys, mothers were present for an average of 9·0 educational modules (SD 7·8) while fathers were present for an average of 2·4 educational modules (SD 2·8). Finally, as a method of assessing intervention delivery quality, we administered three survey questions designed as riddles for which the answer was based specifically on information covered in a session visit. We found significant (p<0·0001) effects of the intervention on answers to all quality-check questions (Table S8, appendix).

For peer review only - http://bmjopen.bmj.com/site/about/guidelines.xhtml

Discussion

In this multi-arm cluster-randomized controlled trial, we implemented an intervention targeting health outcomes for mothers, neonates, and children which featured validated counseling and negotiation techniques delivered by community health workers to households in rural Honduras over the course of 22 months. Our results show improvements in many (but not all) practices related to postnatal care, proper umbilical cord care, and breastfeeding. Parents with children born after receiving the intervention were 16% more likely to report that their newborn's health was checked by a professional in a health facility within three days of birth; 20% more likely to report not using a fajero on the newborn's umbilical cord; and 9% more likely to report their child was breastfed immediately. Importantly, whenever we noted a significant effect on behavior, we also saw an effect on the corresponding knowledge or attitude. We generally noted that our intervention seemed to have more impact on knowledge and attitudes than on practices, which is consistent with the theory that changes in knowledge and attitudes may be required first in order to seed changes in behavior and that such changes are easier to affect.

Although the focus of this paper was on the pooled total effect of treatment, we report two significant pooled spillover effects on the untreated, which were effects due to others in one's village being treated: father holding the child and child with past-month diarrheal illness being treated with zinc. Such effects suggest that some practices, knowledge, and attitudes may be particularly prone to effects of social influence or community structure. Additionally, although our study design randomized villages to

For peer review only - http://bmjopen.bmj.com/site/about/guidelines.xhtml

different dosages, or proportions of households treated, we estimate pooled effects across all dosages. The primary contribution of our findings, therefore, is that we provide estimates of average treatment effects. Such estimates represent the expectation policy-makers can have on average in a setting where different proportions of the community are targeted for intervention (including observational settings in which the intervention is delivered non-randomly). Overall, however, the majority of pooled spillover effect estimates are negligible. Spillover effects may be heterogenous by dosage of households treated, social network structure, or individual treatment status. Further investigation of spillover effects in this study remains the subject of other work.

The design and implementation of this trial had several strengths. The experimental design was robust and involved a two-stage randomization in which we assigned village clusters to treatment dosages and then further assigned households within those clusters to treatment. Similar groups were attained through balancing across a number of village- and household-level covariates captured at the baseline stages of the trial. In varying the dosage of households treated and randomizing some villages to a pure control design in which no households are treated, we were able to estimate pooled spillover effects among those not treated, a feature uncommon to other large-scale public health intervention evaluation. Careful monitoring and training of CCAs through partnerships with IDB resulted in unbiased, high-quality data acquisition across four years from baseline to endline. The selected behavior change strategy was a modified version of the Timed and Targeted Counseling methodology[18] with the P Process tool,[19] which together allowed CCAs to use dialogue counseling methods and interactive story-

For peer review only - http://bmjopen.bmj.com/site/about/guidelines.xhtml

telling to assess current needs and practices of intervention recipients and negotiate on progressive improvement. The approach has been documented as effective in changing a variety of behaviors aligned with the UN's Sustainable Development Goals[8,9,27] and was successfully tailored to the conditions of the current study. Finally, this study assessed a wider array of outcomes than other trials of its kind, which helped to determine the impact of the intervention on various points of the pregnancy and infancy timeline.

The study had a number of limitations. The main aim of the study was to evaluate the effects of a large-scale, household-based counseling intervention on outcomes related to maternal, neonatal, and child health in rural villages in Honduras. In a traditional behavior change communication intervention, households would be targeted based on where pregnant women or young children live, and community members would be exposed to the intervention messages as many times as possible (e.g., in-person meetings, radio messages, flyers, etc.). Neither of these were possible given our randomization scheme (and the parent trial's larger goal of measuring spread of information and behavior via social networks). Additionally, our study area was subject to co-intervention, and it should be noted that both traditional behavior change interventions through the Government of Honduras and supply-side interventions through IDB and the Salud Mesoamerica Initiative were being implemented in both intervention and control villages at the time of study and among both the treated and untreated individuals in our treated villages.[22] However, we took care to ensure that the intervention measured had unique and specific messaging that was evaluated through

For peer review only

this study. Similarly, because the composition of targeted households could vary widely, the intervention delivery was designed to be flexible such that CCAs could discuss educational modules at different times and at different frequencies depending on an individual family's circumstances. Finally, while we tried to use objective measures whenever possible, the primary and secondary outcomes of the study were assessed using self-report.

Many of our findings are consistent with similar home-based intervention packages targeting maternal, neonatal and child health through behavior change communication strategies. Our review of the literature suggests that this is one of the largest RCTs of a maternal and child health intervention covering the full range of outcomes to take place in the Americas. Three studies reporting interventions across rural communities in Southern Ethiopia, India, Cambodia, Kenya, and Zambia which used the ttC counseling strategy found a 10% improvement in immediate breastfeeding,[8] a 25% improvement in exclusive breastfeeding within first six months,[8] a doubling in family planning,[9] and a 7.5-fold increase in skilled birth attendance.[10] One intervention also used Community Scorecards as a mechanism of social accountability,[10] which was not used in the present study, and all used fewer home visits overall. The evaluation of breastfeeding in the current trial was largely based on the Alive & Thrive initiative, which relies on interpersonal communication and community mobilization for home-visit counseling to improve breastfeeding and complementary infant feeding practices in low-income regions.[11] Alive & Thrive's most recent trial in rural Burkina Faso used messaging about feeding through both conversation and physical materials at health center consultations,

For peer review only - http://bmjopen.bmj.com/site/about/guidelines.xhtml

women's support groups, home visits, and public community events, and targeted pregnant or breastfeeding women and their family members. Our findings of impacts on knowledge and attitudes about exclusive breastfeeding are in agreement with their trial results, with their trial showing additional effects on exclusive breastfeeding practices up to 39%.[11]

Even among trials like Alive & Thrive targeting similar outcomes with similar behavior change communication strategies, almost no other study to our knowledge had exclusive messaging by CHWs during in-person home visits nor a targeting strategy that selected households regardless of current or recently pregnant mother status, like the current trial. Two quasi-experimental interventions in rural Indian and Ugandan villages—the Integrated Nutrition and Health Programme (INHP)[28] and MANIFEST intervention,[29] respectively—which used home visits by CHWs to promote prenatal, delivery, and newborn care practices, each reported significant improvements in outcomes related to prenatal check-ups, facility delivery, umbilical cord care, thermal care, and breastfeeding. However, the INHP was designed as an non-governmental organization facilitation of a government program with the primary outcome being the socioeconomic equity of changes in the targeted behaviors, while the MANIFEST intervention involved media communication and health care capacity-building components.[28,29] Additionally, almost no interventions included discussion of regional practices that may have cultural significance but be potentially harmful to the mother or newborn. Our study suggested that inclusion of such practices in our communication

For peer review only - http://bmjopen.bmj.com/site/about/guidelines.xhtml

strategy was effective in impacting knowledge, attitudes, and practices related to fajero use, as well as knowledge and attitudes about the use of chupones.

While our randomization scheme and large sample size increased our confidence that observed differences in outcomes, or lack thereof, would be due to the intervention, further investigation into why change may or may not occur is warranted. For example, many of the primary outcomes were prevalent at baseline, including avoiding harmful substances around umbilical cords (83%), keeping the newborn warm and clothed after birth (99%), father holding child (97%), and father playing with child (90%) (Table S1, appendix). An even larger swath of secondary knowledge and attitudes outcomes were prevalent at the 80% level or above at baseline (Table S1, appendix). High rates of practices, knowledge, and attitudes that align with public health goals clearly decrease the potential for impact by intervention, and more work is needed to further clarify how educational intervention packages can be tailored to meet national and local needs given the existing prevalence of behaviors which impact maternal and child health.

Additionally, the intervention was designed for behavior change and was therefore aimed at the demand-side conditions of health behaviors. In many cases, however, said behaviors may not only rely on prior practices, knowledge, and attitudes, but also on supply-side conditions such as the accessibility of healthcare resources or the quality of local health centers. For example, outcomes evaluated in the current study but which depend on supply-side conditions include folic acid use, preventative check-ups, care-seeking from medical professionals due to the experience of either postnatal or

For peer review only - http://bmjopen.bmj.com/site/about/guidelines.xhtml

newborn danger signs, delivery in a health facility, and zinc treatment for diarrheal illness. Other outcomes were not as dependent on supply-side conditions and were therefore considered in the household's control and potentially more able to be impacted by a counseling intervention, such as preparing a birth plan, immediate and exclusive breastfeeding, withholding chupones and fajeros, keeping the newborn warm and clothed, and skin-to-skin contact with newborn.

Overall, a novel large-scale household-based counseling intervention package randomized at the village and household levels and delivered in a rural setting resulted in meaningful change in practices, knowledge, and attitudes related to preventative care-seeking for newborns, proper umbilical cord care, and immediate breastfeeding. Additional studies are needed to understand the underlying reasons for change in some but not all outcomes. In our opinion, the results of the trial are generalizable to similar communities in this region. Further research is also needed to assess the cost-effectiveness of this approach and its generalizability to other areas or practices. Finally, our two-stage randomization allowed us to identify distinct treatment effects, suggesting that there are factors such as social network ties and community structure which can influence the uptake of behavior change. Further evaluation of the role of social network ties is needed to understand how, when people are given public health education, their response depends on how others around them are coping with similar challenges.

For peer review only

Contributors

Marcus Alexander, Nicholas A. Christakis, and Laura Forastiere formulated the hypothesis and conceptualized this study. World Vision Honduras directed the design of the intervention content, supervised by IDB, and the content was then reviewed by HNL to ensure that it met study criteria. Nicholas A. Christakis, Rennie Negron, Jennifer Nelson, and Emma Iriarte were involved in survey design. Rennie Negron trained data collection teams and managed study implementation, and Jennifer Nelson and Emma Iriarte oversaw intervention delivery. Nicholas A. Christakis and Rennie Negron managed data collection. William Oles, Marcus Alexander and Laura Forastiere analyzed the data, and Marcus Alexander, William Oles, Laura Forastiere and Nicholas A. Christakis interpreted the analyses. William Oles did the literature search. William Oles, Marcus Alexander and Laura Forastiere produced the figures and tables, and, with Rennie Negron and Nicholas A. Christakis wrote the first draft. William Oles, Laura Forastiere, and Rennie Negron wrote the supplementary appendix. All authors reviewed and provided input to the final draft. Nicholas A. Christakis and Laura Forastiere had final responsibility for the decision to submit for publication.

Declaration of interests

Nicholas A. Christakis reports grants from the Bill and Melinda Gates Foundation and the Tata-Yale Alliance. The Bill and Melinda Gates Foundation funded the intervention through a grant to IDB supervised by Jennifer Nelson and Emma Iriarte. All authors declare no competing interests.

For peer review only

Data sharing

The de-identified datasets generated during the study along with the statistical plan and analytic code will be available from the corresponding author on reasonable request at the end of the project after all planned manuscripts have been accepted for publication. We will make the data without identifiers available to users only under a data-sharing agreement that provides for: (1) a commitment to using the data only for research purposes and not to make the attempt of identifying any individual participant; (2) a commitment to securing the data in case there are still some sensitive variables after the identifiers have been removed, by using appropriate computer technology; (3) a commitment to destroying or returning the data after analyses are complete; and (4) a commitment to not publish any information that is not treated at the aggregate level so that no specific characteristics can be linked to small communities.

Acknowledgements

We acknowledge our partners at the Bill and Melinda Gates Foundation and all staff of the Human Nature Lab at Yale University, including: HNL Lab and Project Director Thomas Keegan; Data Scientist Liza Nicoll; software developers Mark McKnight and Wyatt Israel; research coordinators Jai Broome, Catherine Henry, Kevin Garcia, Petergaye Murray, Samy Galvez, Maria Vassimon de Assis; as well as research assistants John Sang Won Lee, Selena Lee, and Drew Prinster. We acknowledge our staff at IDB including Dr. Hugo Godoy, IDB Health Specialist in Honduras, the Salud Mesoamerica Initiative Team, especially Maria Paola Zuniga, Mauricio Perez Calvo, Jose Ignacio Mata, and María Elena Ordóñez, IDB consultant, and partners from the

For peer review only

World Vision Honduras Team including Karen Ramos, Lesbia Garcia, Gertrudis Medrano, and the Dimagi Team who supported the development of the community health application used during the intervention. We also acknowledge our partners at the Ministry of Health Honduras and all our local partners and field data collection team, and we thank all study participants.

Role of the funding source

The trial was an investigator-initiated study supported by grants from the Bill & Melinda Gates Foundation and the Tata Group. The funding sources did not have any role in the design, conduct, or analysis of the study, the writing of the report, or the decision to submit it for publication. All authors had full access to all the data in the study. NAC and LF had final responsibility for the decision to submit for publication.

For peer review only

References

1. Collaborators GCM. Global, regional, national, and selected subnational levels of stillbirths, neonatal, infant, and under-5 mortality, 1980–2015: a systematic analysis for the Global Burden of Disease Study 2015. *The Lancet* 2016; **388**(10053): 1725-74.

2. Liu L, Oza S, Hogan D, et al. Global, regional, and national causes of child mortality in 2000-13, with projections to inform post-2015 priorities: an updated systematic analysis. *Lancet (London, England)* 2015; **385**(9966).

3. Organization WH. WHO recommendations on health promotion interventions for maternal and newborn health 2015, 2015.

4. Organization WH. WHO recommendations on newborn health: guidelines approved by the WHO Guidelines Review Committee., 2017.

5. Kumar V, Mohanty S, Kumar A, et al. Effect of community-based behaviour change management on neonatal mortality in Shivgarh, Uttar Pradesh, India: a cluster-randomised controlled trial. *Lancet (London, England)* 2008; **372**(9644).

6. IGME. UN Inter-agency Group for Child Mortality Estimation: Honduras (2019). 2021. https://childmortality.org/data/Honduras (accessed March 3 2021).

7. Gilmore B, McAuliffe E. Effectiveness of community health workers delivering preventive interventions for maternal and child health in low- and middle-income countries: a systematic review. *BMC public health* 2013; **13**.

8. Beyero M, Reider K, Mekonnen Y. Effect of timed and targeted counseling (ttC) by peer mothers in changing infant and young child feeding practices among mothers of children 0 to 18 months in four districts in Southern Ethiopia. *Federation of American Societies for Experimental Biology* 2013; **27**(1): 620.

9. Vision W. Timed and Targeted Counseling (TTC): A Service Package of the CHW Project Model, 2020.

10. Edward A, Krishnan A, Ettyang G, et al. Can people-centered community-oriented interventions improve skilled birth attendance? Evidence from a quasi-experimental study in rural communities of Cambodia, Kenya, and Zambia. *BMC pregnancy and childbirth* 2020; **20**(1).

11. Cresswell J, Ganaba R, Sarrassat S, et al. The effect of the Alive & Thrive initiative on exclusive breastfeeding in rural Burkina Faso: a repeated cross-sectional cluster randomised controlled trial. *The Lancet Global health* 2019; **7**(3).

12. UNICEF. Honduras (HND) - Demographics, Health & Infant Mortality. 2011-2012. https://data.unicef.org/country/hnd/ (accessed March 2 2021).

13. USAID. STATcompiler: The DHS Program. 2011-2012. https://www.statcompiler.com/en/ (accessed March 2 2021).

14. MMEIG. Internationally comparable MMR estimates by the Maternal Mortality Estimation Inter-Agency Group (MMEIG)–WHO, UNICEF, UNFPA, World Bank Group and the United Nations Population Division: Maternal mortality in 2017 (Honduras). 2017. https://www.who.int/gho/maternal_health/countries/hnd.pdf?ua=1 (accessed March 3 2021).

15. PAHO/WHO. Health Situation in the Americas: Core Indicators 2018. 2018. https://iris.paho.org/bitstream/handle/10665.2/49511/CoreIndicators2018_eng.pdf?sequence=1&isAllowed=y (accessed June 4 2021).

For peer review only - http://bmjopen.bmj.com/site/about/guidelines.xhtml

16. La Forgia GM. Health System Innovations in Central America: Lessons and Impact of New Approaches. 2005; **57**.
17. Shakya HB, Stafford D, Hughes DA, et al. Exploiting social influence to magnify population-level behaviour change in maternal and child health: study protocol for a randomised controlled trial of network targeting algorithms in rural Honduras. 2017.
18. International WV. Timed and Targeted Counseling (ttC). 2016. https://www.wvi.org/health/timed-and-targeted-counseling-ttc (accessed April 13 2021).
19. Collaborative HCC. The P Process: Five Steps to Strategic Communication. Baltimore: Johns Hopkins Bloomberg School of Public Health Center for Communication Programs, 2013.
20. Vision W, IADB. Project REDES: Qualitative Evaluation. 2019. https://socialdigital.iadb.org/sites/default/files/2019-10/Evaluaci%C3%B3n%20Cualitativa%20EV_0.pdf (accessed June 4 2021).
21. IADB. ttC: Timed and Targeted Counseling App for Community Health Workers. 2019. https://socialdigital.iadb.org/en/sph/solutions/ttc-timed-and-targeted-counseling-app-community-health-workers (accessed June 4 2021).
22. AH M, EB P, P Z-B, et al. Supply-side interventions to improve health: Findings from the Salud Mesoamérica Initiative. *PloS one* 2018; **13**(4).
23. A L, M M, R N, W M, NA C, NS C. Using Trellis software to enhance high-quality large-scale network data collection in the field. *Social networks* 2021; **66**.
24. Baird S, Bohren JA, McIntosh C, Özler B. Optimal Design of Experiments in the Presence of Interference. *https://doiorg/101162/rest_a_00716* 2018.
25. Hudgens M, Halloran M. Toward Causal Inference With Interference. *Journal of the American Statistical Association* 2008; **103**(482).
26. Kim D, Hwong A, Stafford D, et al. Social network targeting to maximise population behaviour change: a cluster randomised controlled trial. *Lancet (London, England)* 2015; **386**(9989).
27. Yun Y. Family Member Participation In Community Health Worker Intervention and Maternal Health Behavior: a Study of World Vision's Timed and Targeted Counseling from Cambodia, Guatemala, Kenya, and Zambia [dissertation or thesis]. eCommons: Cornell University; 2018.
28. Baqui A, Rosecrans A, Williams E, et al. NGO facilitation of a government community-based maternal and neonatal health programme in rural India: improvements in equity. *Health policy and planning* 2008; **23**(4).
29. Ekirapa-Kiracho E, Kananura R, Tetui M, et al. Effect of a participatory multisectoral maternal and newborn intervention on maternal health service utilization and newborn care practices: a quasi-experimental study in three rural Ugandan districts. *Global health action* 2017; **10**(sup4).

For peer review only - http://bmjopen.bmj.com/site/about/guidelines.xhtml

Figure captions:

Figure 1: Trial profile: A population-level cluster-randomized controlled trial of maternal and child health intervention to promote behavior change in Honduras.

Figure 2: Direct effects of the RCT of maternal and child health intervention to promote behavior change in Honduras.

For peer review only

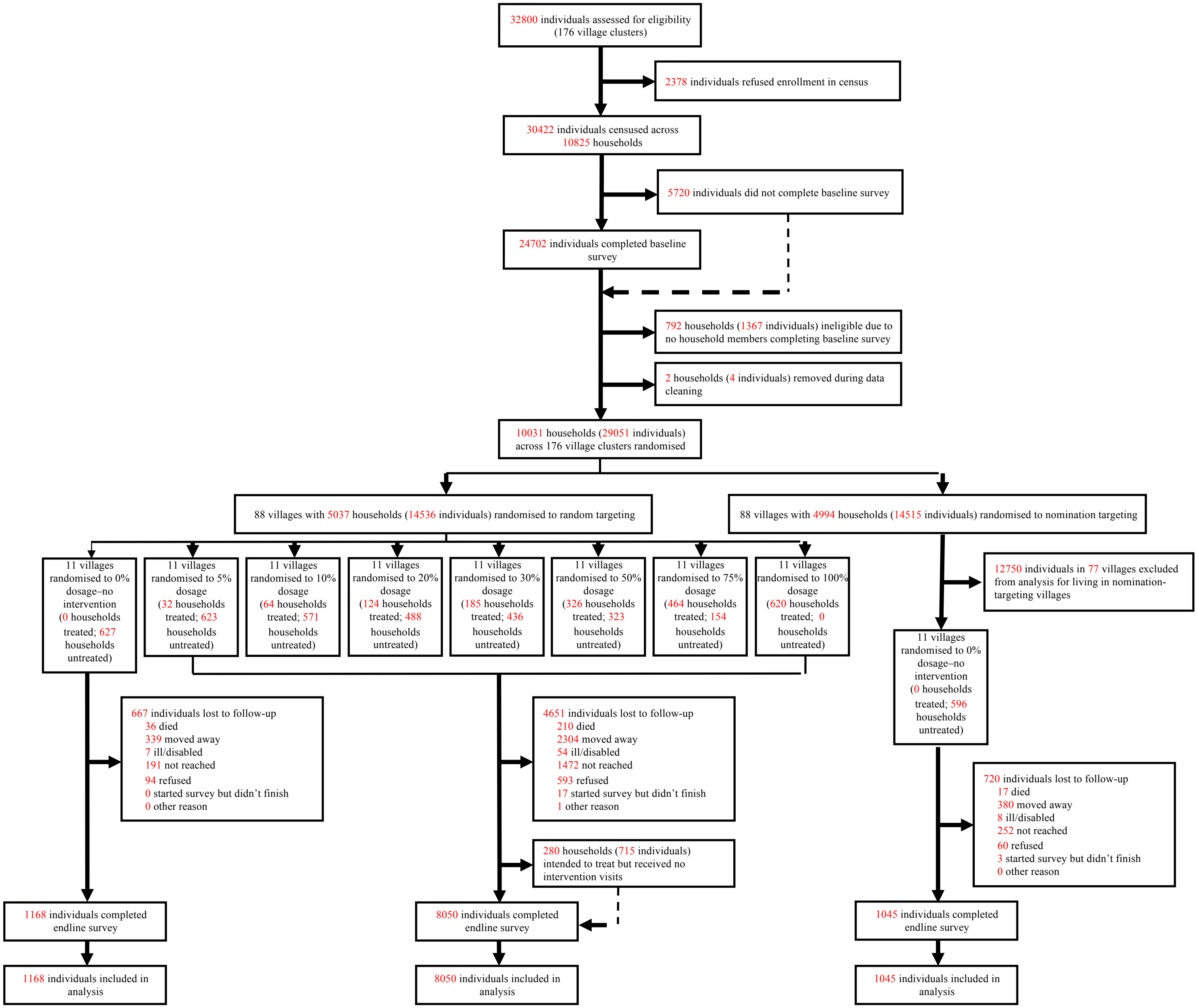
32800 individuals assessed for eligibility (176 village clusters)
2378 individuals refused enrollment in census
30422 individuals censused across 10825 households
5720 individuals did not complete baseline survey
24702 individuals completed baseline survey
792 households (1367 individuals) ineligible due to no household members completing baseline survey
2 households (4 individuals) removed during data cleaning
10031 households (29051 individuals) across 176 village clusters randomised
88 villages with 5037 households (14536 individuals) randomised to random targeting
88 villages with 4994 households (14515 individuals) randomised to nomination targeting
11 villages randomised to 0% dosage–no intervention (0 households treated; 627 households untreated)
11 villages randomised to 5% dosage (32 households treated; 623 households untreated)
11 villages randomised to 10% dosage (64 households treated; 571 households untreated)
11 villages randomised to 20% dosage (124 households treated; 488 households untreated)
11 villages randomised to 30% dosage (185 households treated; 436 households untreated)
11 villages randomised to 50% dosage (326 households treated; 323 households untreated)
11 villages randomised to 75% dosage (464 households treated; 154 households untreated)
11 villages randomised to 100% dosage (620 households treated; 0 households untreated)
12750 individuals in 77 villages excluded from analysis for living in nomination-targeting villages
11 villages randomised to 0% dosage–no intervention (0 households treated; 596 households untreated)
667 individuals lost to follow-up
36 died
339 moved away
7 ill/disabled
191 not reached
94 refused
0 started survey but didn't finish
0 other reason
4651 individuals lost to follow-up
210 died
2304 moved away
54 ill/disabled
1472 not reached
593 refused
17 started survey but didn't finish
1 other reason
280 households (715 individuals) intended to treat but received no intervention visits
720 individuals lost to follow-up
17 died
380 moved away
8 ill/disabled
252 not reached
60 refused
3 started survey but didn't finish
0 other reason
1168 individuals completed endline survey
8050 individuals completed endline survey
1045 individuals completed endline survey
1168 individuals included in analysis
8050 individuals included in analysis
1045 individuals included in analysis

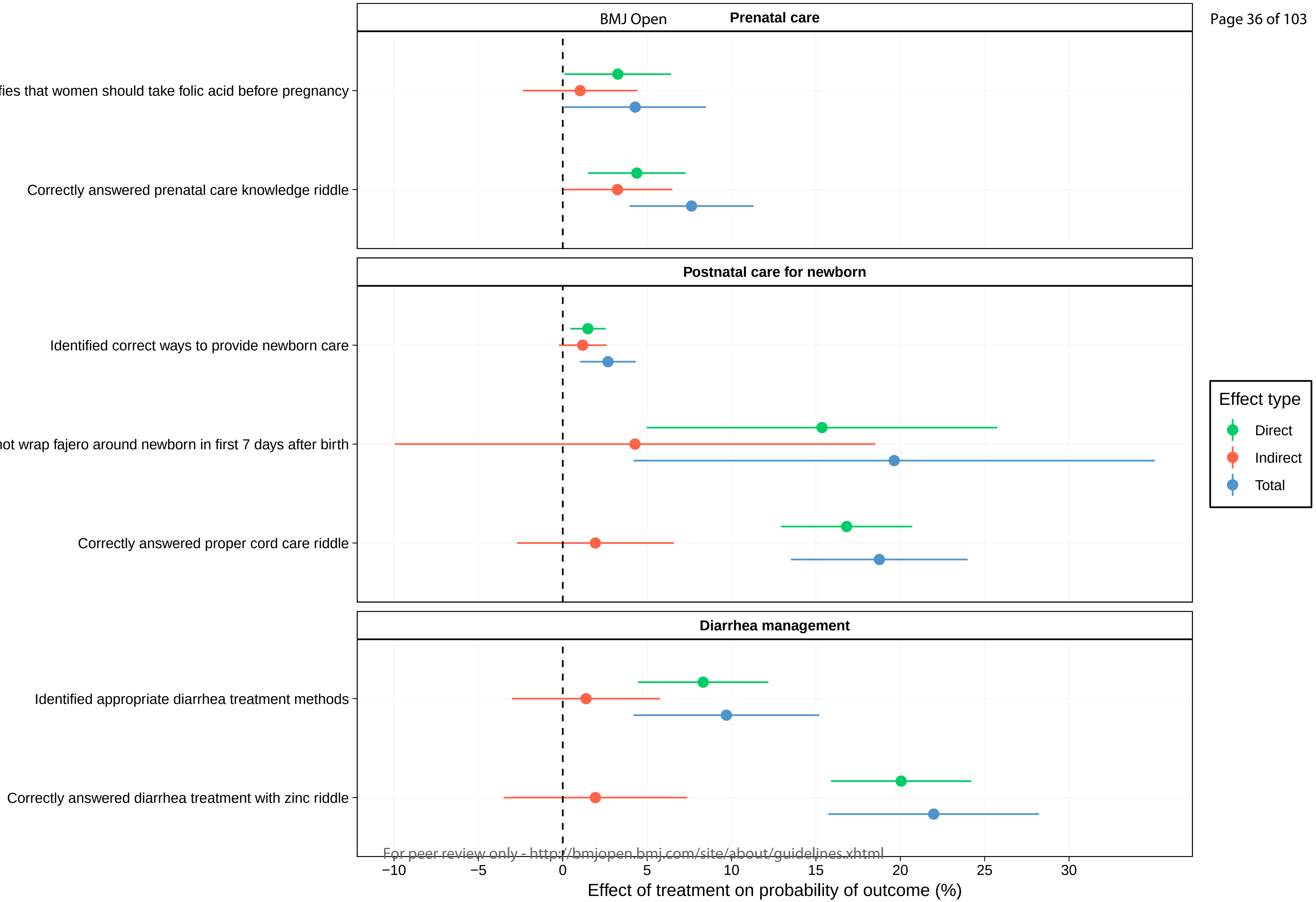

For peer review only - http://bmjopen.bmj.com/site/about/guidelines.xhtml

ONLINE SUPPLEMENTARY APPENDIX

Maternal and child health intervention to promote behavior change: a population-level cluster-randomized controlled trial in Honduras

Marcus Alexander, PhD

William Oles, BS

Rennie Negron, MPH

Jennifer Nelson, MPH

Emma Iriarte, MD

E. M. Airoldi, PhD

Nicholas A. Christakis, MD, PhD, MPH

Laura Forastiere, PhD

For peer review only

Table of Contents

Pilot work 3

Field operations 3

Community engagement 3

Missing data and attrition 3

Blocking of villages and randomisation 3

Assignment of households to treatment/control in each village 4

Intervention sessions 4

Statistical methods 5

Additional analysis 6

Tables 9

Table S1. Pooled direct, indirect, and total effect estimates of intervention on maternal, child, and neonatal outcomes 9

Table S2. Pooled total effect estimates of intervention on maternal, child, and neonatal outcomes, mothers responses for practice outcomes only 20

Table S3. Pooled total effect estimates of intervention on maternal, child, and neonatal outcomes, estimated on sub-sample of respondents who did not move between villages during study 22

Table S4. Pooled total effect estimates of intervention on maternal, child, and neonatal outcomes, with adjustment for baseline demographic characteristics 29

Table S5. Pooled total effect estimates of intervention on maternal, child, and neonatal outcomes, with adjustment for baseline outcome 36

Table S6. Pooled total effect estimates of intervention on maternal, child, and neonatal outcomes, with adjustment for baseline demographic characteristics and baseline outcome 43

Table S7. Pooled total effect estimates of intervention on maternal, child, and neonatal outcomes, with adjustment for baseline demographic characteristics and baseline outcome, estimated on sub-sample of respondents who did not move between villages during study 50

Table S8. Pooled total effect estimates of intervention on maternal, child, and neonatal outcomes, intervention module receipt defined as treatment, with adjustment for baseline demographic characteristics 57

References 65

For peer review only

For peer review only - http://bmjopen.bmj.com/site/about/guidelines.xhtml

Pilot work

Prior to the rollout of our community census and baseline survey efforts, we conducted 3 separate pilot surveys. The first pilot survey, conducted in the summer of 2014, was given to 1018 individuals in 2 towns in the Lempira Department, Honduras, of whom we were able to collect network, demographic, and social normative measures from 831. The second pilot survey was conducted in February 2015, and covered 4 small villages in the Copan region, and included 165 individuals with questions on networks, demographics, as well as reproductive, maternal, child and neonatal health knowledge, attitudes, norms, and behaviors. Our final pilot survey was conducted in May 2015, for which we enrolled 577 individuals. After each pilot we carefully analyzed the data for anomalies, including large number of missing responses, no variation in item responses, or responses that seemed irregular in ways suggesting response bias or misunderstanding of questions. The survey was then revised accordingly.

Field operations

We recruited and trained over 100 local surveyors to perform preliminary data collection and infrastructure development, complete participant recruitment and census enumeration, and conduct the survey interviews. We also established 4 field offices in geographically strategic locations to minimize travel time to and from the study villages. All offices were fully equipped with data collection tablets, netbook computers, printers, high speed internet, and a local server to insure secure download, synchronization and transfer of research data. All surveyors were extensively trained in the use of our survey instruments and software by US-based project managers and supervised by Honduran project coordinators with whom we had daily contact in relation to data collection and other implementation activities.

Community engagement

We developed valuable relationships with local government and health officials, participating in staff meetings for health center personnel and community health workers. We presented project plans and obtained approval of our field operations and data collection procedures from the Honduras Ministry of Health (MOH). The MOH reviewed our study protocols, consents and survey instruments and provided feedback. Our field teams have also met with local community leaders and indigenous council members to present study objectives in all study villages prior to beginning recruitment and data collection.

Missing data and attrition

Of the 32800 estimated eligible individuals in the study villages, 93% agreed to participate in the study, so bias due to failure to recruit participants is likely to be very low. At the follow-up and final stages, we made our best efforts to track study participants enrolled at baseline who discontinued for any reason, and we attempted to continue surveying participants who moved from one study village to another. Follow-up data and attrition are reported for each treatment arm in our comprehensive trial profile shown in supplemental figure S1. Of the 5633 households initially randomized in the trial that were a part of the treatment arms included in the current analysis, 4861 households (86%) had at least one respondent who completed the endline survey. A chi-square test of independence showed that there was no significant association between household treatment status and attrition, X^2 (1, N = 5633) = 0.80, p = .37.

Blocking of villages and randomisation

Villages were assigned to treatment arms in an 8x2 factorial design—8 dosages of households treated per village (0, 0.05, 0.1, 0.2, 0.3, 0.5, 0.75, 1), and 2 targeting strategies (random target, friend nomination target). We ran a re-randomization procedure that generated 10000 assignments of villages to treatment arms. This procedure generates assignments while controlling balance on the following variables: number of respondents in the village, average number of respondents in the household, latitude, longitude, elevation, time to health center, and time to maternity clinic.

For peer review only - http://bmjopen.bmj.com/site/about/guidelines.xhtml

This procedure prioritized matching the means and standard deviations of the 16 treatment arms, but also weights the third moments and cross-moments (to get at covariances). The procedure also assigns a score that summarizes balance. We checked how similar these assignments were. Of the 100 assignments with a better balance summary score, no two contained a pair of treatment arms that overlap in more than 8 elements. We also measured the average maximum overlap, defined as the maximum number of items that any pair of treatment arms have in common. On average, over the 100 assignments with better balance summary score, the maximum overlap was 3.985, indicating that the top 100 assignments of villages to treatment arms were are all quite different.

Focusing on the 100 assignments with better balance summary score, as candidate re-randomizations, we then ran a battery of statistical tests check for statistically significant imbalances. We focused on the following variables and tests quoted in parenthesis: number of respondents in the village (t test), average number of respondents in the household (t test), latitude (t test), longitude (t test), elevation (t test), time to health center (t test), time to maternity clinic (t test), number of households (t test), empirical distribution of household sizes (Kolmogorov-Smirnov test), village can be accessed in when raining (proportions), proportion of indigenous population (t test), time in minutes to main road (t test), village is a coffee producer (proportions), average number of women of reproductive age (t test), average age (t test), and percent male (t test).

For each of the 100 assignments we produced a table with these tests for each of 147 contrasts: 120 contrasts reported the p-values for imbalance in pairs of treatment arms for each of the covariates; 26 contrasts reported the p-values for marginal imbalance in pairs of levels of treatment; 1 contrast reported the p-values for marginal imbalance in the two nomination schemes. We selected assignment number 73, which had only one imbalance at significance 5%. The imbalance was for average age between treatment arms 3 (random target, 10% treated households) and 14 (friend nomination target, 50% treated households), which is an unimportant contrast for the purpose of conducting the analyses proposed in this study.

Assignment of households to treatment/control in each village

Dosage is to be understood as proportion of households treated per village, which was applied to the number of households in each village and the rounded to the nearest whole integer number. We built a social network where a tie meant that there was a nomination in any of the following name generators: personal private matters; spent free time; closest friend. For each village, we generated 10000 assignments of households to treatment in villages in the friend nomination arm. For each of these assignments we then computed balance for the following covariates: number of census respondents, number of women of reproductive age, number of children under the age of 12 who live in this household, existence of handwashing location observed by enumerator, household electricity, separate room in the house that is used as the kitchen, self-reported health status, and network degree centrality of the household. We also considered the following individual variables averaged at the household level: age, sex, and baseline self-reported health status of the individuals in the households.

Here, for each of the 144 villages assigned to a treatment arm in which the percentage of household treated differs from 0% and 100%, we only considered the contrast: treatment vs. control. We then selected the set of assignments of household to treatment and control in each of the 144 villages that minimized the number of tests for imbalance failed at 5%. These balanced sets of assignments constituted the final assignments of households to treatment in villages in the random target arm, but only the initial assignment of households to treatment in villages in the friend nomination arm. For villages in the friend nomination arm, we picked each of the households initially assigned to treatment, in turn, then picked a person in the household, then picked a tie at random among this person's ties. Whenever we ran into a duplicate, we restarted the choice. This procedure generated a final set of assignments of households to treatment in villages in the friend nomination arm. For the purposes of analyses of direct effects of the intervention, households in villages in the friend nomination arm were excluded since they were targeted nonrandomly to receive the intervention.

Intervention sessions

Our community-based behavioral intervention took course over 22 months (November 2016–August 2018). It was comprised of a series of household-level counseling sessions targeting maternal, child, and neonatal practices using 15 distinct educational modules delivered over a maximum of 22 sessions. The 15 educational modules along with

For peer review only - http://bmjopen.bmj.com/site/about/guidelines.xhtml

the percentage of targeted households randomized to treatment in random-target villages which receiving the module are listed below. Table S8 contains additional analysis which incorporates specific module receipt into our estimation of treatment effects and also describes which modules were paired with primary and secondary outcomes.

Module	Description of module	% targeted households receiving the module (at least one session)
m1	Pregnancy care and prenatal care in first 12 weeks	77.1
m2	Preparing a birth plan	76.3
m3	Facility-based birth	72.9
m4	Importance of family planning	75.2
m5	Care of woman during childbirth and postpartum period and newborn care	74.0
m6	Care of woman during childbirth and postpartum period and newborn care	72.2
m7	Newborn care and danger signs	72.9
m8	Infant care: caring for children between 1–6 months	78.1
m9	Danger signs and seeking medical attention for acute respiratory infections and diarrheal illness	77.5
m10	Reproductive life plan	80.7
m11	Importance of folic acid for mom and newborn	80.1
m12	Importance of preventing teen pregnancy (<18 years old)	78.7
m13	Self-esteem, values, life goals	78.5
m14	Prevention of inter-partner violence; non-violent communication	79.6
m15	Zika prevention	77.3

Statistical methods

The opportunity to answer certain questions on our survey was determined by eligibility encoded into the survey design. For example, only mothers and fathers that had a child since the end of the intervention (September 1, 2018) were eligible to have a response included for the question about whether their child was exclusively breastfed for the first 6 months. For each outcome, we restricted the analysis to the respondents eligible to answer the related question. The respondent denominator for all outcomes analyzed can be found in Table S1. Each outcome variable was coded as binary: 0 if the response was the undesired outcome related to the intervention, and 1 if the response was the desired outcome related to the intervention. Responses of 'Refusal' for any survey question were excluded for the purposes of our analysis. Responses of 'Don't know' were coded as an undesired for outcomes related to knowledge but were otherwise excluded for outcomes about practices or attitudes. The unit of analysis was the individual respondent.

In our randomized experiment, first villages were either randomly assigned to different dosages, or proportion of treated households, or to no intervention, and then household were randomly assigned to the intervention according to the dosage assigned to their village. Here we only consider the villages assigned to random targeting. Because of this, three groups of respondents exist that can be compared: pure controls (those not targeted living in villages where no one is targeted); within-cluster controls (those not targeted living in villages where a nonzero dosage of people is targeted); and treated (those targeted). Due to the presence of interference among households, in order to estimate the effect of receiving the intervention, we needed to correct for the indirect effect affecting the untreated households in a village assigned to random targeting. We make the assumption that interference could be present within villages but not between them (also known as the partial interference assumption), and that a respondent's outcome depends on the proportion of treated household in the village in addition to its own treatment (stratified interference).[1,2] Under this assumption, we denote by Yic(Tic, Pc) the potential outcome of individual i in village c under individual treatment Tic and a proportion of treated households in the village c equal to Pc. We define the following causal effects:

$$TE(p): E[Y(1,p)-Y(0,0)]$$

For peer review only - http://bmjopen.bmj.com/site/about/guidelines.xhtml

$$DE(p): E[Y(1,p)-Y(0,p)]$$

$$SE(p):E[Y(0,p)-Y(0,0)]$$

TE(p) is the total effect of receiving the treatment while a proportion of p households is also treated in the village compare to the control condition without any intervention in the village, DE(p) is the average direct effect of receiving the treatment versus not in a village where a proportion of p households are treated, and SE(p) is the indirect (or spillover) effect of having a proportion of p households treated in the village for the untreated individuals. We have that for each p, the total effect TE(p) is the sum of the spillover effect on the untreated SE(p) and the direct effect DE(p). Here we focus on the total and direct effects, while accounting for spillover effects. To this end, we estimate the pooled total effect TE and the pooled direct effect DE averaging across dosages p. We estimated pooled effects by combining observations from all dosages in order to measure an average effect for the entire study population. The pooled total effect can be seen as the effect on the treated due to the intervention, while the pooled direct effect is the effect of directly receiving the treatment controlling for spillover effects.

To estimate these pooled causal effects in presence of interference[1], we used the following regression model:

$$\text{probit}(Y_{ic}) = \beta_0 + \beta_{total} T_{ic} + \beta_{indirect} S_{ic}$$

where Y_{ic} is the observed outcome of individual i in village c ,β_0 is the population average outcome, β_{total} is the pooled total effect, T_{ic} is the treated status of individual i in village c, $\beta_{indirect}$ is the pooled indirect effect, and S_{ic} is the within-village control status of individual i in village c. Given the estimated coefficients we predicted the pooled potential outcomes for the treated individual, for the within-village control individuals, and for the pure controls. total and spillover effects were estimated subtracting the corresponding potential outcomes.

Since the pooled total effect is the sum of the pooled indirect effect and the pooled direct effect, the pooled direct effect was computed by subtracting the estimated effects:

$$DE=TE-SE$$

In addition, this estimation of pooled effects required the addition of saturation weights, since targeted individuals are given disproportionately high weight in high-dosage villages and disproportionately low weight in low-dosage villages when observations are pooled. Saturation weights were calculated on the village-level based on the empirical proportion of respondents targeted. The indirect and total effect estimates used robust standard errors clustered on village, and standard errors for the direct effect estimates were calculated using the delta method.

We performed additional sensitivity analysis to test the robustness of our primary findings. Results for our additional analyses are reported in the supplemental tables below. Table S1 reports direct and total effect estimates for all primary and secondary outcomes, and also details the percentage of respondents who reported the desired outcome at baseline. Table S2 reports the direct and total effect estimates for a sub-sample of respondents that was limited to mother of children born after the intervention period, and effects are reported only for the primary outcomes. Table S3 reports the direct and total effect estimates for all primary and secondary outcomes for a sub-sample of respondents that was limited to those who did not move between villages, and therefore treatment arms, during the study period. Table S4 reports the direct and total effect estimates for all primary and secondary outcomes, adjusted for baseline demographic characteristics (including age, gender, marital status, indigenous status, and household wealth index). Table S5 reports the direct and total effect estimates for all primary and secondary outcomes, adjusted for outcomes at baseline. Table S6 reports the direct and total effect estimates, adjusted for baseline demographic characteristics and baseline outcomes. Table S7 reports the direct and total effect estimates for a sub-sample of respondents that was limited to those who did not move between villages, adjusted for baseline demographic characteristics and baseline outcomes. Table S8 reports the direct and total effect estimates using relevant intervention module receipt as indicators of treatment, adjusted for baseline demographic characteristics.

Additional analysis

When analysis of practices was limited to the responses of mothers, the probability of having the newborn's health checked by a professional in a health facility within three days and of not using a fajero on the umbilical cord were even greater among the targeted, and, additionally, a significant effect on the likelihood that mothers reported that the father was involved with bathing their child emerged (Table S2, appendix). We performed additional sensitivity

For peer review only - http://bmjopen.bmj.com/site/about/guidelines.xhtml

analyses by excluding respondents that moved between treatment arms during the study, adjusting for the respondent's baseline responses for each outcome, and adjusting for demographic factors including gender, age, education, marital status, indigenous status, and household wealth index (Table S7, appendix). When all of the aforementioned covariates were included, we noted that there were no significant total effects for seeking a professional check-up for a newborn within three days of birth or for immediate breastfeeding, while the total effect estimate for not using a fajero increased. In these analyses, additional significant total effects also emerged for mothers seeking medical care after experiencing a postnatal danger sign and parents providing their children with proper treatment (defined as providing zinc, antibiotics, additional food, or oral rehydration therapy) after their child experienced past-month diarrheal illness. The variation in effects with individual covariate adjustment suggests that the impact of the intervention is heterogenous and that, while we are able to report pooled effects across the study population, it is expected that some individuals will be more likely to respond to the interventions than others.

For peer review only

For peer review only - http://bmjopen.bmj.com/site/about/guidelines.xhtml

Figures

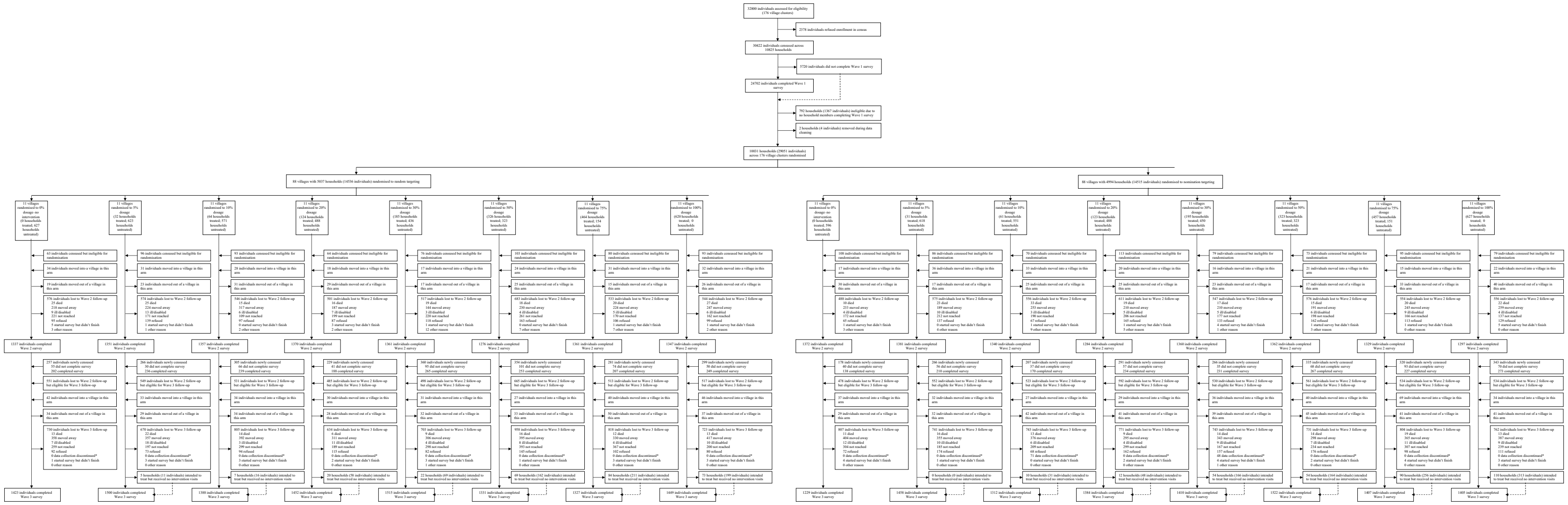

Figure S1. Trial profile for parent study

8

For peer review only - http://bmjopen.bmj.com/site/about/guidelines.xhtml

Tables

Table S1. Pooled direct, indirect, and total effect estimates of intervention on maternal, child, and neonatal outcomes

	Outcome type	Respondent denominator	Pooled direct effect estimate		Pooled indirect effect estimate		Pooled total effect estimate		N	Outcome at baseline survey (%)
			Effect size (95% CI)	p value	Effect size (95% CI)	p value	Effect size (95% CI)	p value		
**Prenatal care**										
Currently taking folic acid tablets (daily in past 7 days)	Practice	All women 15+ with a complete survey	-0·0049 (-0·034 to 0·024)	0·74	0·0053 (-0·036 to 0·047)	0·80	0·0004 (-0·043 to 0·044)	0·98	6311	32
Made a birth plan in preparation for birth	Practice	All respondents with a complete survey and a child born since 2018-09-01 (after the end of the intervention)	0·020 (-0·071 to 0·11)	0·67	0·054 (-0·020 to 0·13)	0·15	0·074 (-0·015 to 0·16)	0·10	619	61
Saved money in preparation for birth	Practice	All respondents with a complete survey and a child born since 2018-09-01 (after the end of the intervention)	0·046 (-0·061 to 0·15)	0·40	-0·065 (-0·15 to 0·023)	0·14	-0·020 (-0·14 to 0·098)	0·74	620	63
Sold animal in preparation for birth	Practice	All respondents with a complete survey and a child born since 2018-09-01 (after the end of the intervention)	-0·011 (-0·079 to 0·058)	0·76	-0·0044 (-0·055 to 0·046)	0·86	-0·015 (-0·080 to 0·049)	0·65	619	NA
Sought prenatal care within 12 weeks	Practice	All respondents with a complete survey and a child born since 2018-09-01 (after the end of the intervention)	-0·042 (-0·17 to 0·090)	0·54	-0·011 (-0·12 to 0·095)	0·84	-0·053 (-0·19 to 0·089)	0·46	597	73
Identifies that women should take folic acid before pregnancy	Knowledge and attitudes	All respondents with a complete survey	0·033 (0·0010 to 0·064)	0·044	0·010 (-0·024 to 0·044)	0·55	0·043 (0·0008 to 0·085)	0·046	10262	73
Identifies that women should seek prenatal care first 12 weeks of pregnancy	Knowledge and attitudes	All respondents with a complete survey	0·019 (-0·0061 to 0·044)	0·14	0·0038 (-0·018 to 0·026)	0·73	0·023 (-0·0046 to 0·050)	0·10	10261	87

For peer review only - http://bmjopen.bmj.com/site/about/guidelines.xhtml

For peer review only

Identifies accompanying woman to prenatal care visits as method of support during pregnancy	Knowledge and attitudes	All respondents with a complete survey	0·0051 (-0·025 to 0·036)	0·74	-0·011 (-0·060 to 0·038)	0·66	-0·0058 (-0·059 to 0·048)	0·83	10257	NA
Identifies ensuring that woman eats well as method of support during pregnancy	Knowledge and attitudes	All respondents with a complete survey	-0·0072 (-0·040 to 0·025)	0·66	-0·012 (-0·057 to 0·034)	0·61	-0·019 (-0·071 to 0·032)	0·47	10257	NA
Identifies ensuring that woman rests 1 hour per day well as method of support during pregnancy	Knowledge and attitudes	All respondents with a complete survey	0·0046 (-0·018 to 0·028)	0·69	-0·0012 (-0·034 to 0·031)	0·94	0·0034 (-0·028 to 0·035)	0·83	10257	NA
Identifies avoiding violence as method of support during pregnancy	Knowledge and attitudes	All respondents with a complete survey	-0·014 (-0·032 to 0·0037)	0·12	-0·018 (-0·036 to 0·0003)	0·054	-0·032 (-0·053 to -0·011)	0·0023	10257	NA
Identifies encouraging women to take vitamins as method of support during pregnancy	Knowledge and attitudes	All respondents with a complete survey	-0·0046 (-0·027 to 0·018)	0·68	-0·019 (-0·053 to 0·016)	0·29	-0·023 (-0·060 to 0·013)	0·21	10257	NA
Identifies helping woman with house work/child care as method of support during pregnancy	Knowledge and attitudes	All respondents with a complete survey	0·019 (-0·012 to 0·050)	0·24	0·053 (-0·0039 to 0·11)	0·068	0·072 (0·010 to 0·13)	0·022	10257	NA
Identifies saving animals to sell as method of preparing for birth expenses	Knowledge and attitudes	All respondents with a complete survey	0·017 (-0·0027 to 0·036)	0·093	0·016 (-0·0098 to 0·043)	0·22	0·033 (0·0025 to 0·063)	0·034	10261	NA
Identifies knowing cost of trip to maternal clinic as method of preparing for birth expenses	Knowledge and attitudes	All respondents with a complete survey	-0·0049 (-0·023 to 0·014)	0·61	-0·0019 (-0·031 to 0·027)	0·90	-0·0068 (-0·038 to 0·024)	0·67	10261	NA
Identifies having a savings plan as method of preparing for birth expenses	Knowledge and attitudes	All respondents with a complete survey	0·030 (0·013 to 0·047)	0·0004	0·0013 (-0·025 to 0·027)	0·92	0·031 (0·0022 to 0·060)	0·035	10261	NA
Correctly answered prenatal care knowledge riddle	Intervention knowledge	All respondents with a complete survey	0·044 (0·015 to 0·073)	0·0030	0·032 (0 to 0·065)	0·050	0·076 (0·040 to 0·11)	<0·0001	10261	NA
**Facility based birth**										
Gave birth in health facility	Practice	All respondents with a complete survey and a child born since 2018-09-01 (after the end of the intervention)	0·018 (-0·075 to 0·11)	0·70	-0·031 (-0·17 to 0·11)	0·67	-0·013 (-0·15 to 0·13)	0·86	620	72
Believes health facility is best place to give birth	Knowledge and attitudes	All respondents with a complete survey	0·021 (0·0036 to 0·039)	0·018	-0·013 (-0·050 to 0·024)	0·48	0·0080 (-0·028 to 0·044)	0·66	10262	90
**Pregnancy danger signs**										

For peer review only - http://bmjopen.bmj.com/site/about/guidelines.xhtml

Woman sought medical care for pregnancy danger sign	Practice	All respondents with a complete survey and a child born since 2018-09-01 (after the end of the intervention) who experienced a prenatal care danger sign	-0·026 (-0·23 to 0·18)	0·80	-0·081 (-0·24 to 0·076)	0·31	-0·11 (-0·33 to 0·12)	0·35	235	64
Identified bleeding as pregnancy d.s.	Knowledge and attitudes	All respondents with a complete survey	0·018 (-0·015 to 0·052)	0·28	0·021 (-0·017 to 0·058)	0·28	0·039 (-0·0039 to 0·082)	0·075	10260	41
Identified seizure as pregnancy d.s.	Knowledge and attitudes	All respondents with a complete survey	0·0048 (-0·0047 to 0·014)	0·32	-0·0020 (-0·0094 to 0·0054)	0·60	0·0028 (-0·0063 to 0·012)	0·54	10260	6
Identified headache as pregnancy d.s.	Knowledge and attitudes	All respondents with a complete survey	0·027 (-0·012 to 0·066)	0·18	0·0079 (-0·031 to 0·046)	0·69	0·035 (-0·017 to 0·086)	0·18	10260	39
Identified ringing in ears as pregnancy d.s.	Knowledge and attitudes	All respondents with a complete survey	0·0040 (-0·0053 to 0·013)	0·40	0·0010 (-0·0055 to 0·0074)	0·77	0·0050 (-0·0052 to 0·015)	0·34	10260	4
Identified dizziness as pregnancy d.s.	Knowledge and attitudes	All respondents with a complete survey	-0·014 (-0·048 to 0·020)	0·42	0·0031 (-0·042 to 0·048)	0·89	-0·011 (-0·060 to 0·038)	0·66	10260	37
Identified difficulty urinating as pregnancy d.s.	Knowledge and attitudes	All respondents with a complete survey	0·0024 (-0·0033 to 0·0081)	0·41	-0·0025 (-0·0095 to 0·0046)	0·49	-0·0001 (-0·0078 to 0·0077)	0·99	10260	2
Identified reduced or absent fetal movement as pregnancy d.s.	Knowledge and attitudes	All respondents with a complete survey	0·0087 (-0·0087 to 0·026)	0·33	0·0096 (-0·0044 to 0·024)	0·18	0·018 (-0·0048 to 0·042)	0·12	10260	6
Identified water breaking as pregnancy d.s.	Knowledge and attitudes	All respondents with a complete survey	-0·0025 (-0·0088 to 0·0037)	0·43	0·0024 (-0·0037 to 0·0084)	0·45	-0·0002 (-0·0074 to 0·0070)	0·96	10260	2
Identified fever as pregnancy d.s.	Knowledge and attitudes	All respondents with a complete survey	0·012 (-0·012 to 0·036)	0·34	0·027 (-0·0056 to 0·059)	0·11	0·039 (0·0013 to 0·076)	0·042	10260	14
Identified swelling of face/hands/feet as pregnancy d.s.	Knowledge and attitudes	All respondents with a complete survey	0·017 (-0·0095 to 0·044)	0·21	0·017 (-0·0061 to 0·040)	0·15	0·034 (0·0032 to 0·065)	0·030	10260	10
**Postnatal care for mother**										
Mother had health checked by professional within 3 days of birth	Practice	All respondents with a complete survey and a child born since 2018-09-01 (after the end of the intervention)	0·051 (-0·060 to 0·16)	0·37	0·088 (-0·055 to 0·23)	0·23	0·14 (-0·024 to 0·30)	0·094	605	14
Mother had health checked by professional within 7 days of birth	Practice	All respondents with a complete survey and a child born since 2018-09-01 (after the	0·00020 (-0·13 to 0·13)	1·00	0·12 (-0·0002 to 0·25)	0·050	0·12 (-0·037 to 0·28)	0·13	605	27

For peer review only - http://bmjopen.bmj.com/site/about/guidelines.xhtml

		end of the intervention)								
Mother sought medical care for postnatal danger sign	Practice	All respondents with a complete survey and a child born since 2018-09-01 (after the end of the intervention)	0·063 (-0·14 to 0·26)	0·54	0·16 (-0·020 to 0·33)	0·082	0·22 (-0·0038 to 0·44)	0·054	194	48
Identifies that mother should receive postnatal medical check-up within 3 days of birth	Knowledge and attitudes	All respondents with a complete survey	0·088 (0·058 to 0·12)	<0·0001	-0·0007 (-0·037 to 0·036)	0·97	0·088 (0·043 to 0·13)	0·0001	10261	9
Identifies that mother should receive postnatal medical check-up within 7 days of birth	Knowledge and attitudes	All respondents with a complete survey	0·061 (0·026 to 0·097)	0·0008	-0·0025 (-0·046 to 0·041)	0·91	0·059 (0·0086 to 0·11)	0·022	10261	24
Identified heavy vaginal bleeding as postnatal d.s.	Knowledge and attitudes	All respondents with a complete survey	0·047 (0·011 to 0·083)	0·010	0·0011 (-0·032 to 0·034)	0·95	0·048 (0·0074 to 0·088)	0·020	10260	43
Identified fever as postnatal d.s.	Knowledge and attitudes	All respondents with a complete survey	0·025 (-0·010 to 0·060)	0·17	0·026 (-0·012 to 0·063)	0·18	0·051 (0·0024 to 0·099)	0·040	10260	38
Identified vaginal discharge as postnatal d.s.	Knowledge and attitudes	All respondents with a complete survey	0·0083 (-0·0022 to 0·019)	0·12	-0·0055 (-0·017 to 0·0060)	0·35	0·0029 (-0·010 to 0·016)	0·67	10260	7
Identified headache as postnatal d.s.	Knowledge and attitudes	All respondents with a complete survey	0·019 (-0·0070 to 0·044)	0·15	0·0073 (-0·027 to 0·041)	0·67	0·026 (-0·013 to 0·065)	0·20	10260	22
Identified convulsions or fits as postnatal d.s.	Knowledge and attitudes	All respondents with a complete survey	0·0021 (-0·0021 to 0·0062)	0·33	-0·0019 (-0·0066 to 0·0028)	0·44	0·0002 (-0·0051 to 0·0056)	0·94	10260	1
**Postnatal care for newborn**										
Newborn had health checked by professional within 3 days of birth	Practice	All respondents with a complete survey and a child born since 2018-09-01 (after the end of the intervention)	0·087 (-0·026 to 0·20)	0·13	0·077 (-0·033 to 0·19)	0·17	0·16 (0·031 to 0·30)	0·016	610	15
Newborn had health checked by professional within 7 days of birth	Practice	All respondents with a complete survey and a child born since 2018-09-01 (after the end of the intervention)	0·079 (-0·050 to 0·21)	0·23	0·049 (-0·047 to 0·14)	0·32	0·13 (-0·014 to 0·27)	0·077	610	29
Newborn experienced health problem in first month, care was sought	Practice	All respondents with a complete survey and a child born since 2018-09-01 (after the	-0·081 (-0·34 to 0·18)	0·54	0·11 (-0·070 to 0·29)	0·23	0·030 (-0·22 to 0·28)	0·81	177	74

For peer review only - http://bmjopen.bmj.com/site/about/guidelines.xhtml

		end of the intervention)								
Identified correct ways to provide newborn care[a]	Knowledge and attitudes	All respondents with a complete survey	0·015 (0·0044 to 0·026)	0·0055	0·012 (-0·0023 to 0·026)	0·10	0·027 (0·010 to 0·043)	0·0014	10261	5
Identified fever as newborn d.s.	Knowledge and attitudes	All respondents with a complete survey	-0·023 (-0·062 to 0·016)	0·25	0·028 (-0·011 to 0·067)	0·15	0·0055 (-0·044 to 0·055)	0·83	10260	69
Identified diarrhea as newborn d.s.	Knowledge and attitudes	All respondents with a complete survey	0·0096 (-0·019 to 0·038)	0·51	-0·0002 (-0·038 to 0·037)	0·99	0·0093 (-0·032 to 0·050)	0·65	10260	49
Identified difficulty breathing as newborn d.s.	Knowledge and attitudes	All respondents with a complete survey	0·0099 (-0·010 to 0·030)	0·34	0·0033 (-0·028 to 0·035)	0·84	0·013 (-0·024 to 0·050)	0·48	10260	36
Identified vomiting as newborn d.s.	Knowledge and attitudes	All respondents with a complete survey	0·0018 (-0·018 to 0·021)	0·86	0·0084 (-0·017 to 0·034)	0·52	0·010 (-0·017 to 0·038)	0·47	10260	27
Identified poor appetite as newborn d.s.	Knowledge and attitudes	All respondents with a complete survey	-0·0001 (-0·011 to 0·011)	0·99	-0·0064 (-0·014 to 0·0012)	0·097	-0·0065 (-0·016 to 0·0032)	0·19	10260	6
Identified redness/bleeding around cord as newborn d.s.	Knowledge and attitudes	All respondents with a complete survey	0·0099 (-0·0027 to 0·023)	0·13	0·0047 (-0·014 to 0·024)	0·63	0·015 (-0·0056 to 0·035)	0·16	10260	5
Identified pus in cord as newborn d.s.	Knowledge and attitudes	All respondents with a complete survey	0·013 (0·0016 to 0·024)	0·025	0·0006 (-0·012 to 0·013)	0·93	0·013 (-0·0021 to 0·028)	0·092	10260	5
Identified cold skin as newborn d.s.	Knowledge and attitudes	All respondents with a complete survey	0·0042 (-0·0048 to 0·013)	0·36	-0·0025 (-0·0086 to 0·0037)	0·43	0·0017 (-0·0067 to 0·010)	0·69	10260	2
Identified cough as newborn d.s.	Knowledge and attitudes	All respondents with a complete survey	-0·034 (-0·063 to -0·0054)	0·020	0·015 (-0·026 to 0·057)	0·47	-0·019 (-0·065 to 0·027)	0·42	10260	2
Identified cold as newborn d.s.	Knowledge and attitudes	All respondents with a complete survey	-0·022 (-0·052 to 0·0085)	0·16	0·0028 (-0·045 to 0·050)	0·91	-0·019 (-0·074 to 0·036)	0·50	10260	1
Identified problems latching as newborn d.s.	Knowledge and attitudes	All respondents with a complete survey	0·0009 (-0·0080 to 0·0098)	0·84	-0·0051 (-0·015 to 0·0053)	0·34	-0·0041 (-0·017 to 0·0082)	0·51	10260	0
Identified pneumonia as newborn d.s.	Knowledge and attitudes	All respondents with a complete survey	0·0027 (-0·025 to 0·031)	0·85	-0·0037 (-0·037 to 0·029)	0·82	-0·0010 (-0·037 to 0·035)	0·95	10260	NA
Did not use harmful substances to treat cord stump	Practice	All respondents with a complete survey and a child born since 2018-09-01 (after the end of the intervention)	0·070 (-0·052 to 0·19)	0·26	0·022 (-0·098 to 0·14)	0·72	0·092 (-0·042 to 0·23)	0·18	598	83
Did not wrap fajero around newborn in first 7 days after birth	Practice	All respondents with a complete survey and a child born since 2018-09-01 (after the end of the intervention)	0·15 (0·050 to 0·26)	0·0038	0·043 (-0·100 to 0·19)	0·56	0·20 (0·042 to 0·35)	0·013	616	6

For peer review only - http://bmjopen.bmj.com/site/about/guidelines.xhtml

Identified proper cord care methods[b]	Knowledge and attitudes	All respondents with a complete survey	0·089 (0·056 to 0·12)	<0·0001	0·0015 (-0·044 to 0·047)	0·95	0·091 (0·040 to 0·14)	0·0005	10260	47
Correctly answered proper cord care riddle	Intervention knowledge	All respondents with a complete survey	0·17 (0·13 to 0·21)	<0·0001	0·019 (-0·027 to 0·066)	0·42	0·19 (0·14 to 0·24)	<0·0001	10262	NA
Kept newborn wrapped first 7 days after birth	Practice	All respondents with a complete survey and a child born since 2018-09-01 (after the end of the intervention)	-0·044 (-0·095 to 0·0080)	0·098	0·013 (-0·036 to 0·063)	0·59	-0·030 (-0·086 to 0·026)	0·29	619	99
Held newborn skin-to-skin during first month after birth	Practice	All respondents with a complete survey and a child born since 2018-09-01 (after the end of the intervention)	0·095 (-0·041 to 0·23)	0·17	0·032 (-0·080 to 0·14)	0·57	0·13 (-0·014 to 0·27)	0·077	617	100
**Breastfeeding**										
Exclusively breastfed child first 6 months	Practice	All respondents with a complete survey and a child born since 2018-09-01 (after the end of the intervention)	0·053 (-0·085 to 0·19)	0·45	-0·032 (-0·16 to 0·091)	0·61	0·021 (-0·14 to 0·18)	0·80	619	59
Exclusively breastfed child first 6 months without giving chupón	Practice	All respondents with a complete survey and a child born since 2018-09-01 (after the end of the intervention)	0·086 (-0·046 to 0·22)	0·20	0·012 (-0·10 to 0·12)	0·84	0·097 (-0·048 to 0·24)	0·19	619	34
Breastfed immediately after birth	Practice	All respondents with a complete survey and a child born since 2018-09-01 (after the end of the intervention) who reported a natural birth	0·13 (0·047 to 0·21)	0·0023	-0·042 (-0·12 to 0·035)	0·29	0·089 (0·0028 to 0·17)	0·043	493	86
Identifies that newborn should be breastfed immediately after birth	Knowledge and attitudes	All respondents with a complete survey	0·0045 (-0·019 to 0·028)	0·70	0·010 (-0·016 to 0·037)	0·45	0·015 (-0·018 to 0·048)	0·38	10261	82
Identifies that newborns should only be given breast milk during first 6 months	Knowledge and attitudes	All respondents with a complete survey	0·042 (0·017 to 0·067)	0·0008	-0·015 (-0·041 to 0·011)	0·25	0·027 (-0·0048 to 0·059)	0·096	10261	80

For peer review only

For peer review only - http://bmjopen.bmj.com/site/about/guidelines.xhtml

Believes newborns should not be given chupón during first 6 months	Knowledge and attitudes	All respondents with a complete survey	0·15 (0·11 to 0·19)	<0·0001	-0·011 (-0·060 to 0·038)	0·67	0·14 (0·084 to 0·20)	<0·0001	10261	23
**Paternal involvement**										
Father accompanied mother to clinic for prenatal care visit at least once	Practice	All respondents with a complete survey and a child born since 2018-09-01 (after the end of the intervention) who received prenatal care	0·018 (-0·12 to 0·15)	0·79	-0·064 (-0·21 to 0·087)	0·41	-0·045 (-0·22 to 0·13)	0·62	595	52
Father accompanied mother to seek medical care for pregnancy danger sign	Practice	All respondents with a complete survey and a child born since 2018-09-01 (after the end of the intervention) who experienced a prenatal care danger sign and sought care	0·010 (-0·15 to 0·17)	0·90	-0·0013 (-0·18 to 0·18)	0·99	0·0091 (-0·21 to 0·23)	0·94	149	65
Father waited at birthplace during labor	Practice	All respondents with a complete survey and a child born since 2018-09-01 (after the end of the intervention)	-0·031 (-0·14 to 0·075)	0·56	0·0083 (-0·12 to 0·13)	0·90	-0·023 (-0·16 to 0·11)	0·74	620	78
Father accompanied mother to seek medical care for postnatal danger sign	Practice	All respondents with a complete survey and a child born since 2018-09-01 (after the end of the intervention) and sought care for postpartum problem	0·18 (-0·032 to 0·39)	0·096	-0·037 (-0·32 to 0·24)	0·80	0·14 (-0·17 to 0·45)	0·37	97	67
Father sought medical care for newborn for postnatal danger sign	Practice	All respondents with a complete survey and a child born since 2018-09-01 (after the end of the intervention) and sought care for a postnatal problem	0·0059 (-0·33 to 0·34)	0·97	-0·099 (-0·37 to 0·17)	0·48	-0·093 (-0·44 to 0·25)	0·60	116	73
Father held child	Practice	All respondents with a complete survey and a child born since 2018-	-0·046 (-0·11 to 0·022)	0·19	0·078 (0·013 to 0·14)	0·020	0·032 (-0·040 to 0·10)	0·38	614	97

For peer review only - http://bmjopen.bmj.com/site/about/guidelines.xhtml

		09-01 (after the end of the intervention)								
Father played with child	Practice	All respondents with a complete survey and a child born since 2018-09-01 (after the end of the intervention)	-0·091 (-0·19 to 0·0099)	0·077	0·076 (-0·030 to 0·18)	0·16	-0·015 (-0·13 to 0·10)	0·80	614	90
Father took child to clinic when sick	Practice	All respondents with a complete survey and a child born since 2018-09-01 (after the end of the intervention)	-0·053 (-0·19 to 0·080)	0·44	0·084 (-0·011 to 0·18)	0·082	0·031 (-0·11 to 0·17)	0·66	614	71
Believes father should accompany mother to prenatal care visits	Knowledge and attitudes	All respondents with a complete survey	0·041 (0·022 to 0·060)	<0·0001	0·0024 (-0·022 to 0·027)	0·85	0·043 (0·019 to 0·068)	0·0005	10220	93
Believes father should wait at birth location while mother gives birth	Knowledge and attitudes	All respondents with a complete survey	0·0044 (-0·0078 to 0·017)	0·48	0·0070 (-0·0079 to 0·022)	0·36	0·011 (-0·0062 to 0·029)	0·20	10225	94
Believes father should care for children when sick	Knowledge and attitudes	All respondents with a complete survey	-0·0012 (-0·0069 to 0·0044)	0·67	-0·0015 (-0·0074 to 0·0043)	0·60	-0·0028 (-0·010 to 0·0047)	0·47	10250	99
**Diarrhea management**										
Did not report diarrhea in past 4 weeks	Practice	All respondents with a complete survey	-0·0010 (-0·014 to 0·012)	0·87	-0·0086 (-0·019 to 0·0021)	0·12	-0·0096 (-0·025 to 0·0060)	0·23	10263	97
Used ORT to treat diarrhea in past 4 weeks	Practice	All respondents with a complete survey who had diarrhea in past 4 weeks	-0·14 (-0·30 to 0·022)	0·091	-0·044 (-0·20 to 0·12)	0·59	-0·19 (-0·38 to 0·011)	0·064	266	52
Used appropriate treatment for diarrhea in past 4 weeks[c]	Practice	All respondents with a complete survey who had diarrhea in past 4 weeks	-0·015 (-0·16 to 0·13)	0·84	0·012 (-0·12 to 0·15)	0·86	-0·0027 (-0·16 to 0·16)	0·97	268	28
Child did not experience diarrhea past 4 weeks	Practice	All respondents with a complete survey and a new child <= 5 years old reported at wave 3 who had diarrhea in past 4 weeks	0·0042 (-0·025 to 0·034)	0·78	-0·011 (-0·044 to 0·023)	0·53	-0·0064 (-0·043 to 0·030)	0·73	3333	84
Child experienced diarrhea past 4 weeks, was given appropriate amount of liquids	Practice	All respondents with a complete survey and a new child <= 5 years old reported at	-0·100 (-0·25 to 0·046)	0·18	-0·031 (-0·20 to 0·14)	0·73	-0·13 (-0·31 to 0·050)	0·16	350	50

For peer review only

For peer review only - http://bmjopen.bmj.com/site/about/guidelines.xhtml

		wave 3 who had diarrhea in past 4 weeks								
Child experienced diarrhea past 4 weeks, was given appropriate amount of food	Practice	All respondents with a complete survey and a new child <= 5 years old reported at wave 3 who had diarrhea in past 4 weeks	-0·088 (-0·20 to 0·028)	0·14	-0·024 (-0·15 to 0·099)	0·70	-0·11 (-0·25 to 0·024)	0·11	350	35
Child experienced diarrhea past 4 weeks, was given ORT	Practice	All respondents with a complete survey and a new child <= 5 years old reported at wave 3 who had diarrhea in past 4 weeks	0·082 (-0·069 to 0·23)	0·29	0·056 (-0·089 to 0·20)	0·45	0·14 (-0·032 to 0·31)	0·11	347	60
Child experienced diarrhea past 4 weeks, was given appropriate treatment	Practice	All respondents with a complete survey and a new child <= 5 years old reported at wave 3 who had diarrhea in past 4 weeks	0·027 (-0·10 to 0·15)	0·68	0·076 (-0·056 to 0·21)	0·26	0·10 (-0·059 to 0·27)	0·21	348	31
Child experienced diarrhea past 4 weeks, was given zinc 10-14 days	Practice	All respondents with a complete survey and a new child <= 5 years old reported at wave 3 who had diarrhea in past 4 weeks	0·0080 (-0·030 to 0·046)	0·68	0·15 (0·0026 to 0·29)	0·046	0·16 (-0·014 to 0·33)	0·072	311	1
Identified appropriate diarrhea treatment methods[d]	Knowledge and attitudes	All respondents with a complete survey	0·083 (0·045 to 0·12)	<0·0001	0·014 (-0·030 to 0·058)	0·54	0·097 (0·042 to 0·15)	0·0005	10260	41
Identified zinc 10-14 days as diarrhea treatment	Knowledge and attitudes	All respondents with a complete survey	0·0093 (-0·0073 to 0·026)	0·27	0·020 (-0·0005 to 0·040)	0·056	0·029 (0·0079 to 0·050)	0·0072	10261	4
Identified zinc supplement as way to prevent diarrhea	Knowledge and attitudes	All respondents with a complete survey	0·063 (0·035 to 0·090)	<0·0001	-0·0004 (-0·066 to 0·065)	0·99	0·062 (-0·0063 to 0·13)	0·075	10261	45
Identified breastfeeding as way to prevent diarrhea	Knowledge and attitudes	All respondents with a complete survey	0·031 (0·0028 to 0·060)	0·032	0·0070 (-0·058 to 0·072)	0·83	0·038 (-0·028 to 0·10)	0·26	10261	26
Correctly answered diarrhea treatment with zinc riddle	Intervention knowledge	All respondents with a complete survey	0·20 (0·16 to 0·24)	<0·0001	0·019 (-0·035 to 0·074)	0·49	0·22 (0·16 to 0·28)	<0·0001	10261	NA
**Respiratory illness**										

For peer review only - http://bmjopen.bmj.com/site/about/guidelines.xhtml

Did not report respiratory illness (coughing) for 2 weeks	Practice	All respondents with a complete survey	0·0005 (-0·022 to 0·023)	0·97	-0·017 (-0·045 to 0·011)	0·23	-0·017 (-0·050 to 0·017)	0·33	10263	79
Child did not have cough past 4 weeks	Practice	All respondents with a complete survey	0·0076 (-0·037 to 0·052)	0·74	-0·070 (-0·11 to -0·029)	0·0009	-0·063 (-0·12 to -0·0051)	0·033	3334	67
Child had cough past 4 weeks, care was sought	Practice	All respondents with a complete survey and a new child <= 5 years old reported at wave 3 who had an illness with a cough in past 4 weeks	-0·041 (-0·14 to 0·055)	0·40	0·070 (-0·033 to 0·17)	0·18	0·029 (-0·092 to 0·15)	0·64	700	4
Identified vaccination as way to prevent respiratory illness	Knowledge and attitudes	All respondents with a complete survey	0·021 (-0·00010 to 0·042)	0·051	-0·042 (-0·080 to -0·0035)	0·033	-0·021 (-0·059 to 0·017)	0·28	10261	51
Identified breastfeeding as way to prevent respiratory illness	Knowledge and attitudes	All respondents with a complete survey	0·015 (-0·013 to 0·042)	0·29	-0·0010 (-0·052 to 0·050)	0·97	0·014 (-0·040 to 0·068)	0·62	10261	34
Identified fever as d.s. for children with respiratory illness	Knowledge and attitudes	All respondents with a complete survey	0·0067 (-0·0067 to 0·020)	0·33	-0·0023 (-0·032 to 0·027)	0·88	0·0044 (-0·028 to 0·037)	0·79	10261	82
Identified difficulty breathing as d.s. for children with respiratory illness	Knowledge and attitudes	All respondents with a complete survey	0·011 (0·0032 to 0·019)	0·0057	-0·0064 (-0·023 to 0·011)	0·46	0·0046 (-0·014 to 0·023)	0·62	10261	89
Identified rapid breathing as d.s. for children with respiratory illness	Knowledge and attitudes	All respondents with a complete survey	0·0073 (0·0012 to 0·013)	0·019	-0·0025 (-0·014 to 0·0087)	0·67	0·0048 (-0·0070 to 0·017)	0·42	10261	85
**Reproductive health**										
Reported ever using birth control to delay or avoid pregnancy	Practice	All respondents with a complete survey	0·015 (-0·021 to 0·052)	0·41	-0·033 (-0·084 to 0·019)	0·21	-0·018 (-0·073 to 0·038)	0·53	10257	33
Reported currently using birth control	Practice	All respondents with a complete survey who are not pregnant and have ever used birth control	0·014 (-0·039 to 0·066)	0·61	0·035 (-0·025 to 0·095)	0·26	0·048 (-0·023 to 0·12)	0·19	3793	55
Believes woman should be at least 18 years of age to have her first child	Knowledge and attitudes	All respondents with a complete survey	0·0054 (-0·0010 to 0·012)	0·096	0·0056 (-0·0022 to 0·014)	0·16	0·011 (0·0021 to 0·020)	0·015	9733	98
**Gender/reproductive norms**										
Birth location chosen either jointly or by woman	Practice	All respondents with a complete survey and a child born since 2018-09-01 (after the	0·0093 (-0·061 to 0·080)	0·80	0·039 (-0·045 to 0·12)	0·36	0·048 (-0·054 to 0·15)	0·36	620	93

For peer review only - http://bmjopen.bmj.com/site/about/guidelines.xhtml

		end of the intervention)								

Results based on final sample of N=10263 (household targeted n=3238, household not targeted n=7025). d.s.=Danger sign, NA=Not applicable, ORT=Oral rehydration therapy. Proportions with NA could not be assessed because the outcome was not part of the baseline instrument. Standard errors are clustered at the village level. Robust 95% CIs in parentheses.

[a] 'Identified correct ways to provide newborn care' outcome was coded as correct if the respondent identified holding baby skin to skin, getting their health checked by a professional, immediately putting them to the breast, or making sure their diaper is clean and dry, and did not identify giving purgante, wrapping a fajero, giving a chupon, or bathing them in water right away.
[b] 'Identified proper cord care' outcome was coded as correct if respondent identified applying alcohol, applying iodine, keeping cord clean/dry, or applying peroxide, and did not identify applying oil, applying coffee, applying quina water, wrapping a fajero, applying powder, burning, applying ashes, applying a clamp, or applying thiomersal.
[c] 'Used appropriate treatment for diarrhea in past 4 weeks' outcome was coded as correct if respondent did any of the following: antibiotic, zinc, give extra food, give extra liquid, and did not do any of the following: anti-diarrhea medication, home remedy, laxative, deworming medication, chupon, massage, stop eating foods, stop taking liquids.
[d] 'Identified appropriate diarrhea treatment methods' outcome was coded as correct if respondent identified any of the following: antibiotic, zinc, ORS or Litrosol, give extra food, give extra liquid, get medical attention, and did not identify any of the following: anti-diarrhea medication, laxative, deworming medication, home remedy, chupon, massage, stop giving foods, stop giving liquids, or do nothing.

For peer review only

Table S2. Pooled total effect estimates of intervention on maternal, child, and neonatal outcomes, mothers responses for practice outcomes only

	Pooled total effect estimate		N
	Effect size (95% CI)	p value	
**Prenatal care**			
Currently taking folic acid tablets (daily in past 7 days)	0·0004 (-0·043 to 0·044)	0·98	6311
Made a birth plan in preparation for birth	0·031 (-0·11 to 0·17)	0·66	412
Saved money in preparation for birth	-0·039 (-0·17 to 0·091)	0·56	412
Sold animal in preparation for birth	-0·014 (-0·097 to 0·070)	0·75	412
Sought prenatal care within 12 weeks	-0·031 (-0·19 to 0·12)	0·69	407
**Facility based birth**			
Gave birth in health facility	-0·049 (-0·19 to 0·094)	0·50	412
**Pregnancy danger signs**			
Woman sought medical care for pregnancy danger sign	-0·12 (-0·41 to 0·16)	0·39	152
**Postnatal care for mother**			
Mother had health checked by professional within 3 days of birth	0·14 (-0·038 to 0·32)	0·12	411
Mother had health checked by professional within 7 days of birth	0·11 (-0·058 to 0·27)	0·20	411
Mother sought medical care for postnatal danger sign	0·22 (-0·014 to 0·46)	0·066	131
**Postnatal care for newborn**			
Newborn had health checked by professional within 3 days of birth	0·18 (0·015 to 0·34)	0·032	411
Newborn had health checked by professional within 7 days of birth	0·11 (-0·049 to 0·27)	0·17	411
Newborn experienced health problem in first month, care was sought	-0·0022 (-0·29 to 0·29)	0·99	129
Did not use harmful substances to treat cord stump	0·054 (-0·10 to 0·21)	0·50	410
Did not wrap fajero around newborn in first 7 days after birth	0·24 (0·069 to 0·41)	0·0060	411
Kept newborn wrapped first 7 days after birth	-0·0026 (-0·059 to 0·054)	0·93	412
Held newborn skin-to-skin during first month after birth	0·15 (-0·013 to 0·31)	0·073	411
**Breastfeeding**			
Exclusively breastfed child first 6 months	0·061 (-0·10 to 0·22)	0·47	412
Exclusively breastfed child first 6 months without giving chupón	0·14 (-0·016 to 0·29)	0·079	412
Breastfed immediately after birth	0·10 (-0·0032 to 0·20)	0·057	335

For peer review only - http://bmjopen.bmj.com/site/about/guidelines.xhtml

For peer review only

**Paternal involvement**			
Father accompanied mother to clinic for prenatal care visit at least once	-0·060 (-0·26 to 0·14)	0·55	394
Father accompanied mother to seek medical care for pregnancy danger sign	0·11 (-0·15 to 0·38)	0·41	94
Father waited at birthplace during labor	-0·017 (-0·17 to 0·14)	0·83	412
Father accompanied mother to seek medical care for postnatal danger sign	0·22 (-0·10 to 0·54)	0·18	66
Father sought medical care for newborn for postnatal danger sign	-0·028 (-0·42 to 0·37)	0·89	83
Father held child	0·054 (-0·038 to 0·14)	0·25	406
Father played with child	0·025 (-0·13 to 0·18)	0·75	406
Father took child to clinic when sick	0·059 (-0·11 to 0·23)	0·51	406
**Diarrhea management**			
Did not report diarrhea in past 4 weeks	-0·013 (-0·034 to 0·0070)	0·20	6318
Used ORT to treat diarrhea in past 4 weeks	-0·14 (-0·39 to 0·12)	0·29	181
Used appropriate treatment for diarrhea in past 4 weeks[a]	0·069 (-0·16 to 0·30)	0·55	183
Child did not experience diarrhea past 4 weeks	-0·0035 (-0·040 to 0·033)	0·85	2891
Child experienced diarrhea past 4 weeks, was given appropriate amount of liquids	-0·13 (-0·32 to 0·064)	0·19	291
Child experienced diarrhea past 4 weeks, was given appropriate amount of food	-0·11 (-0·25 to 0·032)	0·13	291
Child experienced diarrhea past 4 weeks, was given ORT	0·13 (-0·052 to 0·32)	0·16	289
Child experienced diarrhea past 4 weeks, was given appropriate treatment	0·090 (-0·081 to 0·26)	0·30	291
Child experienced diarrhea past 4 weeks, was given zinc 10-14 days	0·16 (-0·033 to 0·35)	0·10	260
**Respiratory illness**			
Did not report respiratory illness (coughing) for 2 weeks	-0·040 (-0·082 to 0·0022)	0·063	6318
Child did not have cough past 4 weeks	-0·061 (-0·12 to 0·0012)	0·055	2892
Child had cough past 4 weeks, care was sought	0·055 (-0·068 to 0·18)	0·38	616
**Reproductive health**			
Reported ever using birth control to delay or avoid pregnancy	0·020 (-0·041 to 0·081)	0·52	6316
Reported currently using birth control	0·030 (-0·061 to 0·12)	0·52	2570
**Gender/reproductive norms**			
Birth location chosen either jointly or by woman	0·032 (-0·092 to 0·16)	0·62	412

ORT=Oral rehydration therapy. Standard errors are clustered at the village level. Robust 95% CIs in parentheses.

[a] 'Used appropriate treatment for diarrhea in past 4 weeks' outcome was coded as correct if respondent did any of the following: antibiotic, zinc, give extra food, give extra liquid, and did not do any of the following: anti-diarrhea medication, home remedy, laxative, deworming medication, chupon, massage, stop eating foods, stop taking liquids.

For peer review only - http://bmjopen.bmj.com/site/about/guidelines.xhtml

For peer review only

Table S3. Pooled total effect estimates of intervention on maternal, child, and neonatal outcomes, estimated on sub-sample of respondents who did not move between villages during study

	Outcome type	Respondent denominator	Pooled total effect estimate		N
			Effect size (95% CI)	p value	
**Prenatal care**					
Currently taking folic acid tablets (daily in past 7 days)	Practice	All women 15+ with a complete survey	-0·0029 (-0·048 to 0·042)	0·90	6035
Made a birth plan in preparation for birth	Practice	All respondents with a complete survey and a child born since 2018-09-01 (after the end of the intervention)	0·070 (-0·022 to 0·16)	0·13	567
Saved money in preparation for birth	Practice	All respondents with a complete survey and a child born since 2018-09-01 (after the end of the intervention)	-0·012 (-0·14 to 0·11)	0·85	568
Sold animal in preparation for birth	Practice	All respondents with a complete survey and a child born since 2018-09-01 (after the end of the intervention)	-0·017 (-0·086 to 0·052)	0·62	567
Sought prenatal care within 12 weeks	Practice	All respondents with a complete survey and a child born since 2018-09-01 (after the end of the intervention)	-0·052 (-0·20 to 0·097)	0·50	547
Identifies that women should take folic acid before pregnancy	Knowledge and attitudes	All respondents with a complete survey	0·042 (-0·0008 to 0·084)	0·054	9917
Identifies that women should seek prenatal care first 12 weeks of pregnancy	Knowledge and attitudes	All respondents with a complete survey	0·020 (-0·0077 to 0·048)	0·16	9917
Identifies accompanying woman to prenatal care visits as method of support during pregnancy	Knowledge and attitudes	All respondents with a complete survey	-0·0039 (-0·058 to 0·051)	0·89	9913
Identifies ensuring that woman eats well as method of support during pregnancy	Knowledge and attitudes	All respondents with a complete survey	-0·016 (-0·069 to 0·037)	0·56	9913
Identifies ensuring that woman rests 1 hour per day well as method of support during pregnancy	Knowledge and attitudes	All respondents with a complete survey	0·0091 (-0·024 to 0·042)	0·59	9913
Identifies avoiding violence as method of support during pregnancy	Knowledge and attitudes	All respondents with a complete survey	-0·034 (-0·055 to -0·013)	0·0018	9913
Identifies encouraging women to take vitamins as method of support during pregnancy	Knowledge and attitudes	All respondents with a complete survey	-0·022 (-0·060 to 0·017)	0·27	9913
Identifies helping woman with house work/child care as method of support during pregnancy	Knowledge and attitudes	All respondents with a complete survey	0·074 (0·0092 to 0·14)	0·025	9913
Identifies saving animals to sell as method of preparing for birth expenses	Knowledge and attitudes	All respondents with a complete survey	0·032 (0·0013 to 0·063)	0·041	9917

For peer review only - http://bmjopen.bmj.com/site/about/guidelines.xhtml

For peer review only

Identifies knowing cost of trip to maternal clinic as method of preparing for birth expenses	Knowledge and attitudes	All respondents with a complete survey	-0·0050 (-0·037 to 0·027)	0·76	9917
Identifies having a savings plan as method of preparing for birth expenses	Knowledge and attitudes	All respondents with a complete survey	0·033 (0·0025 to 0·063)	0·034	9917
Correctly answered prenatal care knowledge riddle	Intervention knowledge	All respondents with a complete survey	0·078 (0·039 to 0·12)	0·0001	9916
**Facility based birth**					
Gave birth in health facility	Practice	All respondents with a complete survey and a child born since 2018-09-01 (after the end of the intervention)	-0·0002 (-0·15 to 0·15)	1·00	568
Believes health facility is best place to give birth	Knowledge and attitudes	All respondents with a complete survey	0·0074 (-0·030 to 0·044)	0·69	9917
**Pregnancy danger signs**					
Woman sought medical care for pregnancy danger sign	Practice	All respondents with a complete survey and a child born since 2018-09-01 (after the end of the intervention) who experienced a prenatal care danger sign	-0·12 (-0·36 to 0·12)	0·33	210
Identified bleeding as pregnancy d.s.	Knowledge and attitudes	All respondents with a complete survey	0·040 (-0·0049 to 0·084)	0·081	9916
Identified seizure as pregnancy d.s.	Knowledge and attitudes	All respondents with a complete survey	0·0030 (-0·0064 to 0·012)	0·53	9916
Identified headache as pregnancy d.s.	Knowledge and attitudes	All respondents with a complete survey	0·040 (-0·014 to 0·093)	0·14	9916
Identified ringing in ears as pregnancy d.s.	Knowledge and attitudes	All respondents with a complete survey	0·0053 (-0·0054 to 0·016)	0·33	9916
Identified dizziness as pregnancy d.s.	Knowledge and attitudes	All respondents with a complete survey	-0·013 (-0·064 to 0·038)	0·61	9916
Identified difficulty urinating as pregnancy d.s.	Knowledge and attitudes	All respondents with a complete survey	-0·0002 (-0·0082 to 0·0077)	0·95	9916
Identified reduced or absent fetal movement as pregnancy d.s.	Knowledge and attitudes	All respondents with a complete survey	0·019 (-0·0052 to 0·043)	0·13	9916
Identified water breaking as pregnancy d.s.	Knowledge and attitudes	All respondents with a complete survey	-0·0022 (-0·0091 to 0·0047)	0·53	9916
Identified fever as pregnancy d.s.	Knowledge and attitudes	All respondents with a complete survey	0·036 (-0·0024 to 0·075)	0·066	9916
Identified swelling of face/hands/feet as pregnancy d.s.	Knowledge and attitudes	All respondents with a complete survey	0·035 (0·0030 to 0·067)	0·032	9916
**Postnatal care for mother**					
Mother had health checked by professional within 3 days of birth	Practice	All respondents with a complete survey and a child born since 2018-09-01 (after the end of the intervention)	0·12 (-0·047 to 0·30)	0·15	555

For peer review only - http://bmjopen.bmj.com/site/about/guidelines.xhtml

Mother had health checked by professional within 7 days of birth	Practice	All respondents with a complete survey and a child born since 2018-09-01 (after the end of the intervention)	0·10 (-0·072 to 0·27)	0·25	555
Mother sought medical care for postnatal danger sign	Practice	All respondents with a complete survey and a child born since 2018-09-01 (after the end of the intervention)	0·21 (-0·019 to 0·45)	0·071	178
Identifies that mother should receive postnatal medical check-up within 3 days of birth	Knowledge and attitudes	All respondents with a complete survey	0·087 (0·041 to 0·13)	0·0002	9917
Identifies that mother should receive postnatal medical check-up within 7 days of birth	Knowledge and attitudes	All respondents with a complete survey	0·060 (0·0079 to 0·11)	0·024	9917
Identified heavy vaginal bleeding as postnatal d.s.	Knowledge and attitudes	All respondents with a complete survey	0·053 (0·012 to 0·094)	0·011	9915
Identified fever as postnatal d.s.	Knowledge and attitudes	All respondents with a complete survey	0·049 (0·0007 to 0·098)	0·047	9915
Identified vaginal discharge as postnatal d.s.	Knowledge and attitudes	All respondents with a complete survey	0·0026 (-0·010 to 0·015)	0·68	9915
Identified headache as postnatal d.s.	Knowledge and attitudes	All respondents with a complete survey	0·029 (-0·011 to 0·069)	0·16	9915
Identified convulsions or fits as postnatal d.s.	Knowledge and attitudes	All respondents with a complete survey	0·0006 (-0·0050 to 0·0062)	0·84	9915
**Postnatal care for newborn**					
Newborn had health checked by professional within 3 days of birth	Practice	All respondents with a complete survey and a child born since 2018-09-01 (after the end of the intervention)	0·15 (0·016 to 0·29)	0·029	560
Newborn had health checked by professional within 7 days of birth	Practice	All respondents with a complete survey and a child born since 2018-09-01 (after the end of the intervention)	0·11 (-0·036 to 0·26)	0·14	560
Newborn experienced health problem in first month, care was sought	Practice	All respondents with a complete survey and a child born since 2018-09-01 (after the end of the intervention)	0·067 (-0·20 to 0·33)	0·62	160
Identified correct ways to provide newborn care[a]	Knowledge and attitudes	All respondents with a complete survey	0·026 (0·0096 to 0·043)	0·0019	9917
Identified fever as newborn d.s.	Knowledge and attitudes	All respondents with a complete survey	0·0054 (-0·046 to 0·057)	0·84	9915
Identified diarrhea as newborn d.s.	Knowledge and attitudes	All respondents with a complete survey	0·015 (-0·028 to 0·057)	0·50	9915
Identified difficulty breathing as newborn d.s.	Knowledge and attitudes	All respondents with a complete survey	0·0085 (-0·029 to 0·046)	0·66	9915
Identified vomiting as newborn d.s.	Knowledge and attitudes	All respondents with a complete survey	0·011 (-0·017 to 0·038)	0·45	9915
Identified poor appetite as newborn d.s.	Knowledge and attitudes	All respondents with a complete survey	-0·0062 (-0·016 to 0·0039)	0·23	9915
Identified redness/bleeding around cord as newborn d.s.	Knowledge and attitudes	All respondents with a complete survey	0·016 (-0·0053 to 0·037)	0·14	9915
Identified pus in cord as newborn d.s.	Knowledge and attitudes	All respondents with a complete survey	0·013 (-0·0024 to 0·029)	0·097	9915
Identified cold skin as newborn d.s.	Knowledge and attitudes	All respondents with a complete survey	0·0018 (-0·0069 to 0·011)	0·68	9915

For peer review only - http://bmjopen.bmj.com/site/about/guidelines.xhtml

Identified cough as newborn d.s.	Knowledge and attitudes	All respondents with a complete survey	-0·018 (-0·065 to 0·029)	0·45	9915
Identified cold as newborn d.s.	Knowledge and attitudes	All respondents with a complete survey	-0·016 (-0·072 to 0·040)	0·58	9915
Identified problems latching as newborn d.s.	Knowledge and attitudes	All respondents with a complete survey	-0·0029 (-0·016 to 0·010)	0·67	9915
Identified pneumonia as newborn d.s.	Knowledge and attitudes	All respondents with a complete survey	-0·0009 (-0·039 to 0·037)	0·96	9915
Did not use harmful substances to treat cord stump	Practice	All respondents with a complete survey and a child born since 2018-09-01 (after the end of the intervention)	0·098 (-0·043 to 0·24)	0·17	546
Did not wrap fajero around newborn in first 7 days after birth	Practice	All respondents with a complete survey and a child born since 2018-09-01 (after the end of the intervention)	0·20 (0·038 to 0·37)	0·016	565
Identified proper cord care methods[b]	Knowledge and attitudes	All respondents with a complete survey	0·097 (0·046 to 0·15)	0·0002	9916
Correctly answered proper cord care riddle	Intervention knowledge	All respondents with a complete survey	0·19 (0·13 to 0·24)	<0·0001	9917
Kept newborn wrapped first 7 days after birth	Practice	All respondents with a complete survey and a child born since 2018-09-01 (after the end of the intervention)	-0·040 (-0·10 to 0·022)	0·21	567
Held newborn skin-to-skin during first month after birth	Practice	All respondents with a complete survey and a child born since 2018-09-01 (after the end of the intervention)	0·15 (-0·0050 to 0·30)	0·058	565
**Breastfeeding**					
Exclusively breastfed child first 6 months	Practice	All respondents with a complete survey and a child born since 2018-09-01 (after the end of the intervention)	0·0034 (-0·16 to 0·17)	0·97	567
Exclusively breastfed child first 6 months without giving chupón	Practice	All respondents with a complete survey and a child born since 2018-09-01 (after the end of the intervention)	0·10 (-0·049 to 0·26)	0·18	567
Breastfed immediately after birth	Practice	All respondents with a complete survey and a child born since 2018-09-01 (after the end of the intervention) who reported a natural birth	0·12 (0·035 to 0·21)	0·0055	452
Identifies that newborn should be breastfed immediately after birth	Knowledge and attitudes	All respondents with a complete survey	0·018 (-0·015 to 0·051)	0·28	9917
Identifies that newborns should only be given breast milk during first 6 months	Knowledge and attitudes	All respondents with a complete survey	0·028 (-0·0042 to 0·061)	0·088	9916
Believes newborns should not be given chupón during first 6 months	Knowledge and attitudes	All respondents with a complete survey	0·14 (0·082 to 0·20)	<0·0001	9917
**Paternal involvement**					
Father accompanied mother to clinic for prenatal care visit at least once	Practice	All respondents with a complete survey and a child born since 2018-09-01 (after the end of the intervention) who received prenatal care	-0·053 (-0·25 to 0·14)	0·60	544
Father accompanied mother to seek medical care for pregnancy danger sign	Practice	All respondents with a complete survey and a child born since 2018-09-01 (after the end of the intervention) who experienced a prenatal care danger sign and sought care	-0·073 (-0·30 to 0·16)	0·53	134

For peer review only - http://bmjopen.bmj.com/site/about/guidelines.xhtml

Father waited at birthplace during labor	Practice	All respondents with a complete survey and a child born since 2018-09-01 (after the end of the intervention)	-0·036 (-0·18 to 0·11)	0·63	568
Father accompanied mother to seek medical care for postnatal danger sign	Practice	All respondents with a complete survey and a child born since 2018-09-01 (after the end of the intervention) and sought care for postpartum problem	0·13 (-0·19 to 0·45)	0·43	90
Father sought medical care for newborn for postnatal danger sign	Practice	All respondents with a complete survey and a child born since 2018-09-01 (after the end of the intervention) and sought care for a postnatal problem	-0·060 (-0·43 to 0·31)	0·75	103
Father held child	Practice	All respondents with a complete survey and a child born since 2018-09-01 (after the end of the intervention)	0·033 (-0·044 to 0·11)	0·40	563
Father played with child	Practice	All respondents with a complete survey and a child born since 2018-09-01 (after the end of the intervention)	-0·023 (-0·15 to 0·11)	0·73	563
Father took child to clinic when sick	Practice	All respondents with a complete survey and a child born since 2018-09-01 (after the end of the intervention)	0·014 (-0·13 to 0·16)	0·85	563
Believes father should accompany mother to prenatal care visits	Knowledge and attitudes	All respondents with a complete survey	0·044 (0·019 to 0·069)	0·0006	9876
Believes father should wait at birth location while mother gives birth	Knowledge and attitudes	All respondents with a complete survey	0·011 (-0·0068 to 0·029)	0·22	9881
Believes father should care for children when sick	Knowledge and attitudes	All respondents with a complete survey	-0·0025 (-0·010 to 0·0051)	0·52	9906
**Diarrhea management**					
Did not report diarrhea in past 4 weeks	Practice	All respondents with a complete survey	-0·0097 (-0·026 to 0·0066)	0·24	9918
Used ORT to treat diarrhea in past 4 weeks	Practice	All respondents with a complete survey who had diarrhea in past 4 weeks	-0·19 (-0·39 to 0·0012)	0·051	263
Used appropriate treatment for diarrhea in past 4 weeks[c]	Practice	All respondents with a complete survey who had diarrhea in past 4 weeks	-0·0006 (-0·16 to 0·16)	0·99	265
Child did not experience diarrhea past 4 weeks	Practice	All respondents with a complete survey and a new child <= 5 years old reported at wave 3 who had diarrhea in past 4 weeks	-0·0045 (-0·042 to 0·033)	0·82	3150
Child experienced diarrhea past 4 weeks, was given appropriate amount of liquids	Practice	All respondents with a complete survey and a new child <= 5 years old reported at wave 3 who had diarrhea in past 4 weeks	-0·15 (-0·34 to 0·037)	0·12	327
Child experienced diarrhea past 4 weeks, was given appropriate amount of food	Practice	All respondents with a complete survey and a new child <= 5 years old reported at wave 3 who had diarrhea in past 4 weeks	-0·12 (-0·26 to 0·015)	0·081	327
Child experienced diarrhea past 4 weeks, was given ORT	Practice	All respondents with a complete survey and a new child <= 5 years old reported at wave 3 who had diarrhea in past 4 weeks	0·12 (-0·054 to 0·30)	0·17	324
Child experienced diarrhea past 4 weeks, was given appropriate treatment	Practice	All respondents with a complete survey and a new child <= 5 years old reported at wave 3 who had diarrhea in past 4 weeks	0·12 (-0·056 to 0·29)	0·19	326
Child experienced diarrhea past 4 weeks, was given zinc 10-14 days	Practice	All respondents with a complete survey and a new child <= 5 years old reported at wave 3 who had diarrhea in past 4 weeks	0·17 (-0·014 to 0·35)	0·071	291

For peer review only - http://bmjopen.bmj.com/site/about/guidelines.xhtml

Identified appropriate diarrhea treatment methods[d]	Knowledge and attitudes	All respondents with a complete survey	0·097 (0·040 to 0·15)	0·0008	9916
Identified zinc 10-14 days as diarrhea treatment	Knowledge and attitudes	All respondents with a complete survey	0·030 (0·0077 to 0·052)	0·0081	9917
Identified zinc supplement as way to prevent diarrhea	Knowledge and attitudes	All respondents with a complete survey	0·061 (-0·0089 to 0·13)	0·087	9917
Identified breastfeeding as way to prevent diarrhea	Knowledge and attitudes	All respondents with a complete survey	0·036 (-0·032 to 0·10)	0·30	9917
Correctly answered diarrhea treatment with zinc riddle	Intervention knowledge	All respondents with a complete survey	0·23 (0·16 to 0·29)	<0·0001	9916
**Respiratory illness**					
Did not report respiratory illness (coughing) for 2 weeks	Practice	All respondents with a complete survey	-0·015 (-0·049 to 0·019)	0·39	9918
Child did not have cough past 4 weeks	Practice	All respondents with a complete survey	-0·067 (-0·13 to -0·0079)	0·027	3150
Child had cough past 4 weeks, care was sought	Practice	All respondents with a complete survey and a new child <= 5 years old reported at wave 3 who had an illness with a cough in past 4 weeks	0·035 (-0·091 to 0·16)	0·58	661
Identified vaccination as way to prevent respiratory illness	Knowledge and attitudes	All respondents with a complete survey	-0·022 (-0·060 to 0·017)	0·27	9917
Identified breastfeeding as way to prevent respiratory illness	Knowledge and attitudes	All respondents with a complete survey	0·013 (-0·042 to 0·068)	0·65	9917
Identified fever as d.s. for children with respiratory illness	Knowledge and attitudes	All respondents with a complete survey	0·0052 (-0·027 to 0·037)	0·75	9917
Identified difficulty breathing as d.s. for children with respiratory illness	Knowledge and attitudes	All respondents with a complete survey	0·0048 (-0·014 to 0·023)	0·61	9917
Identified rapid breathing as d.s. for children with respiratory illness	Knowledge and attitudes	All respondents with a complete survey	0·0050 (-0·0070 to 0·017)	0·41	9917
**Reproductive health**					
Reported ever using birth control to delay or avoid pregnancy	Practice	All respondents with a complete survey	-0·022 (-0·081 to 0·036)	0·46	9913
Reported currently using birth control	Practice	All respondents with a complete survey who are not pregnant and have ever used birth control	0·047 (-0·028 to 0·12)	0·22	3604
Believes woman should be at least 18 years of age to have her first child	Knowledge and attitudes	All respondents with a complete survey	0·011 (0·0023 to 0·020)	0·014	9407
**Gender/reproductive norms**					

For peer review only

Birth location chosen either jointly or by woman	Practice	All respondents with a complete survey and a child born since 2018-09-01 (after the end of the intervention)	0·048 (-0·054 to 0·15)	0·36	568

Results based on sub-sample of respondents who didn't move villages during the study N=9918 (household targeted n=3129, household not targeted n=6789). d.s.=Danger sign, ORT=Oral rehydration therapy. Standard errors are clustered at the village level. Robust 95% CIs in parentheses.

[a] 'Identified correct ways to provide newborn care' outcome was coded as correct if the respondent identified holding baby skin to skin, getting their health checked by a professional, immediately putting them to the breast, or making sure their diaper is clean and dry, and did not identify giving purgante, wrapping a fajero, giving a chupon, or bathing them in water right away.
[b] 'Identified proper cord care' outcome was coded as correct if respondent identified applying alcohol, applying iodine, keeping cord clean/dry, or applying peroxide, and did not identify applying oil, applying coffee, applying quina water, wrapping a fajero, applying powder, burning, applying ashes, applying a clamp, or applying thiomersal.
[c] 'Used appropriate treatment for diarrhea in past 4 weeks' outcome was coded as correct if respondent did any of the following: antibiotic, zinc, give extra food, give extra liquid, and did not do any of the following: anti-diarrhea medication, home remedy, laxative, deworming medication, chupon, massage, stop eating foods, stop taking liquids.
[d] 'Identified appropriate diarrhea treatment methods' outcome was coded as correct if respondent identified any of the following: antibiotic, zinc, ORS or Litrosol, give extra food, give extra liquid, get medical attention, and did not identify any of the following: anti-diarrhea medication, laxative, deworming medication, home remedy, chupon, massage, stop giving foods, stop giving liquids, or do nothing.

For peer review only

For peer review only - http://bmjopen.bmj.com/site/about/guidelines.xhtml

Table S4. Pooled total effect estimates of intervention on maternal, child, and neonatal outcomes, with adjustment for baseline demographic characteristics

	Outcome type	Respondent denominator	Pooled total effect estimate		N
			Effect size (95% CI)	p value	
**Prenatal care**					
Currently taking folic acid tablets (daily in past 7 days)	Practice	All women 15+ with a complete survey	-0·0059 (-0·049 to 0·037)	0·79	5922
Made a birth plan in preparation for birth	Practice	All respondents with a complete survey and a child born since 2018-09-01 (after the end of the intervention)	0·026 (-0·080 to 0·13)	0·63	536
Saved money in preparation for birth	Practice	All respondents with a complete survey and a child born since 2018-09-01 (after the end of the intervention)	-0·090 (-0·22 to 0·039)	0·17	540
Sold animal in preparation for birth	Practice	All respondents with a complete survey and a child born since 2018-09-01 (after the end of the intervention)	-0·013 (-0·078 to 0·051)	0·69	535
Sought prenatal care within 12 weeks	Practice	All respondents with a complete survey and a child born since 2018-09-01 (after the end of the intervention)	-0·0044 (-0·13 to 0·12)	0·94	518
Identifies that women should take folic acid before pregnancy	Knowledge and attitudes	All respondents with a complete survey	0·060 (0·028 to 0·093)	0·0003	9197
Identifies that women should seek prenatal care first 12 weeks of pregnancy	Knowledge and attitudes	All respondents with a complete survey	0·032 (0·011 to 0·053)	0·0033	9196
Identifies accompanying woman to prenatal care visits as method of support during pregnancy	Knowledge and attitudes	All respondents with a complete survey	0·0020 (-0·048 to 0·052)	0·94	9192
Identifies ensuring that woman eats well as method of support during pregnancy	Knowledge and attitudes	All respondents with a complete survey	-0·027 (-0·077 to 0·023)	0·29	9192
Identifies ensuring that woman rests 1 hour per day well as method of support during pregnancy	Knowledge and attitudes	All respondents with a complete survey	0·0095 (-0·022 to 0·041)	0·56	9183
Identifies avoiding violence as method of support during pregnancy	Knowledge and attitudes	All respondents with a complete survey	-0·029 (-0·051 to -0·0079)	0·0073	9192
Identifies encouraging women to take vitamins as method of support during pregnancy	Knowledge and attitudes	All respondents with a complete survey	-0·019 (-0·054 to 0·017)	0·30	9192
Identifies helping woman with house work/child care as method of support during pregnancy	Knowledge and attitudes	All respondents with a complete survey	0·085 (0·030 to 0·14)	0·0022	9192
Identifies saving animals to sell as method of preparing for birth expenses	Knowledge and attitudes	All respondents with a complete survey	0·023 (-0·0023 to 0·048)	0·075	9187

For peer review only - http://bmjopen.bmj.com/site/about/guidelines.xhtml

Identifies knowing cost of trip to maternal clinic as method of preparing for birth expenses	Knowledge and attitudes	All respondents with a complete survey	-0·0044 (-0·035 to 0·026)	0·78	9196
Identifies having a savings plan as method of preparing for birth expenses	Knowledge and attitudes	All respondents with a complete survey	0·039 (0·012 to 0·067)	0·0047	9172
Correctly answered prenatal care knowledge riddle	Intervention knowledge	All respondents with a complete survey	0·090 (0·054 to 0·13)	<0·0001	9196
**Facility based birth**					
Gave birth in health facility	Practice	All respondents with a complete survey and a child born since 2018-09-01 (after the end of the intervention)	0·026 (-0·088 to 0·14)	0·66	540
Believes health facility is best place to give birth	Knowledge and attitudes	All respondents with a complete survey	0·024 (-0·00060 to 0·049)	0·056	9188
**Pregnancy danger signs**					
Woman sought medical care for pregnancy danger sign	Practice	All respondents with a complete survey and a child born since 2018-09-01 (after the end of the intervention) who experienced a prenatal care danger sign	-0·040 (-0·25 to 0·17)	0·71	209
Identified bleeding as pregnancy d.s.	Knowledge and attitudes	All respondents with a complete survey	0·051 (0·013 to 0·090)	0·0090	9195
Identified seizure as pregnancy d.s.	Knowledge and attitudes	All respondents with a complete survey	0·0020 (-0·0076 to 0·012)	0·68	9186
Identified headache as pregnancy d.s.	Knowledge and attitudes	All respondents with a complete survey	0·036 (-0·010 to 0·083)	0·13	9195
Identified ringing in ears as pregnancy d.s.	Knowledge and attitudes	All respondents with a complete survey	0·0031 (-0·0064 to 0·013)	0·52	9171
Identified dizziness as pregnancy d.s.	Knowledge and attitudes	All respondents with a complete survey	-0·0026 (-0·049 to 0·044)	0·91	9195
Identified difficulty urinating as pregnancy d.s.	Knowledge and attitudes	All respondents with a complete survey	-0·0018 (-0·0094 to 0·0059)	0·65	9171
Identified reduced or absent fetal movement as pregnancy d.s.	Knowledge and attitudes	All respondents with a complete survey	0·014 (-0·0060 to 0·034)	0·17	9171
Identified water breaking as pregnancy d.s.	Knowledge and attitudes	All respondents with a complete survey	-0·0007 (-0·0079 to 0·0066)	0·86	9171
Identified fever as pregnancy d.s.	Knowledge and attitudes	All respondents with a complete survey	0·040 (0·0012 to 0·079)	0·043	9195
Identified swelling of face/hands/feet as pregnancy d.s.	Knowledge and attitudes	All respondents with a complete survey	0·034 (0·0050 to 0·064)	0·022	9195
**Postnatal care for mother**					
Mother had health checked by professional within 3 days of birth	Practice	All respondents with a complete survey and a child born since 2018-09-01 (after the end of the intervention)	0·13 (-0·024 to 0·29)	0·096	530

For peer review only - http://bmjopen.bmj.com/site/about/guidelines.xhtml

Mother had health checked by professional within 7 days of birth	Practice	All respondents with a complete survey and a child born since 2018-09-01 (after the end of the intervention)	0·075 (-0·065 to 0·22)	0·30	530
Mother sought medical care for postnatal danger sign	Practice	All respondents with a complete survey and a child born since 2018-09-01 (after the end of the intervention)	0·12 (-0·072 to 0·31)	0·23	178
Identifies that mother should receive postnatal medical check-up within 3 days of birth	Knowledge and attitudes	All respondents with a complete survey	0·073 (0·031 to 0·11)	0·0006	9196
Identifies that mother should receive postnatal medical check-up within 7 days of birth	Knowledge and attitudes	All respondents with a complete survey	0·040 (-0·0085 to 0·089)	0·11	9196
Identified heavy vaginal bleeding as postnatal d.s.	Knowledge and attitudes	All respondents with a complete survey	0·067 (0·029 to 0·11)	0·0006	9195
Identified fever as postnatal d.s.	Knowledge and attitudes	All respondents with a complete survey	0·060 (0·0098 to 0·11)	0·019	9195
Identified vaginal discharge as postnatal d.s.	Knowledge and attitudes	All respondents with a complete survey	0·0046 (-0·010 to 0·019)	0·54	9180
Identified headache as postnatal d.s.	Knowledge and attitudes	All respondents with a complete survey	0·029 (-0·0086 to 0·066)	0·13	9195
Identified convulsions or fits as postnatal d.s.	Knowledge and attitudes	All respondents with a complete survey	0·00 (-0·0056 to 0·0056)	1·00	8983
**Postnatal care for newborn**					
Newborn had health checked by professional within 3 days of birth	Practice	All respondents with a complete survey and a child born since 2018-09-01 (after the end of the intervention)	0·16 (0·029 to 0·30)	0·017	532
Newborn had health checked by professional within 7 days of birth	Practice	All respondents with a complete survey and a child born since 2018-09-01 (after the end of the intervention)	0·085 (-0·041 to 0·21)	0·19	532
Newborn experienced health problem in first month, care was sought	Practice	All respondents with a complete survey and a child born since 2018-09-01 (after the end of the intervention)	0·0030 (-0·23 to 0·23)	0·98	154
Identified correct ways to provide newborn care[a]	Knowledge and attitudes	All respondents with a complete survey	0·024 (0·010 to 0·037)	0·0005	9172
Identified fever as newborn d.s.	Knowledge and attitudes	All respondents with a complete survey	0·0022 (-0·045 to 0·049)	0·93	9195
Identified diarrhea as newborn d.s.	Knowledge and attitudes	All respondents with a complete survey	0·0035 (-0·037 to 0·044)	0·86	9195
Identified difficulty breathing as newborn d.s.	Knowledge and attitudes	All respondents with a complete survey	0·0099 (-0·025 to 0·045)	0·58	9171
Identified vomiting as newborn d.s.	Knowledge and attitudes	All respondents with a complete survey	0·0090 (-0·021 to 0·039)	0·56	9186
Identified poor appetite as newborn d.s.	Knowledge and attitudes	All respondents with a complete survey	-0·0049 (-0·015 to 0·0047)	0·32	9171
Identified redness/bleeding around cord as newborn d.s.	Knowledge and attitudes	All respondents with a complete survey	0·020 (-0·0017 to 0·041)	0·072	9195
Identified pus in cord as newborn d.s.	Knowledge and attitudes	All respondents with a complete survey	0·015 (-0·0014 to 0·031)	0·074	9186
Identified cold skin as newborn d.s.	Knowledge and attitudes	All respondents with a complete survey	0·0023 (-0·0068 to 0·012)	0·61	9171

For peer review only - http://bmjopen.bmj.com/site/about/guidelines.xhtml

Identified cough as newborn d.s.	Knowledge and attitudes	All respondents with a complete survey	-0·019 (-0·069 to 0·031)	0·45	9195
Identified cold as newborn d.s.	Knowledge and attitudes	All respondents with a complete survey	-0·017 (-0·067 to 0·034)	0·52	9195
Identified problems latching as newborn d.s.	Knowledge and attitudes	All respondents with a complete survey	-0·0013 (-0·015 to 0·012)	0·85	9171
Identified pneumonia as newborn d.s.	Knowledge and attitudes	All respondents with a complete survey	0·0061 (-0·030 to 0·042)	0·74	9171
Did not use harmful substances to treat cord stump	Practice	All respondents with a complete survey and a child born since 2018-09-01 (after the end of the intervention)	0·089 (-0·042 to 0·22)	0·18	527
Did not wrap fajero around newborn in first 7 days after birth	Practice	All respondents with a complete survey and a child born since 2018-09-01 (after the end of the intervention)	0·27 (0·11 to 0·42)	0·0006	535
Identified proper cord care methods[b]	Knowledge and attitudes	All respondents with a complete survey	0·095 (0·046 to 0·14)	0·0001	9195
Correctly answered proper cord care riddle	Intervention knowledge	All respondents with a complete survey	0·21 (0·16 to 0·25)	<0·0001	9197
Kept newborn wrapped first 7 days after birth	Practice	All respondents with a complete survey and a child born since 2018-09-01 (after the end of the intervention)	-0·050 (-0·11 to 0·012)	0·12	530
Held newborn skin-to-skin during first month after birth	Practice	All respondents with a complete survey and a child born since 2018-09-01 (after the end of the intervention)	0·11 (-0·019 to 0·24)	0·095	537
**Breastfeeding**					
Exclusively breastfed child first 6 months	Practice	All respondents with a complete survey and a child born since 2018-09-01 (after the end of the intervention)	-0·0046 (-0·14 to 0·14)	0·95	539
Exclusively breastfed child first 6 months without giving chupón	Practice	All respondents with a complete survey and a child born since 2018-09-01 (after the end of the intervention)	0·061 (-0·082 to 0·20)	0·40	539
Breastfed immediately after birth	Practice	All respondents with a complete survey and a child born since 2018-09-01 (after the end of the intervention) who reported a natural birth	0·090 (-0·0078 to 0·19)	0·071	428
Identifies that newborn should be breastfed immediately after birth	Knowledge and attitudes	All respondents with a complete survey	0·016 (-0·018 to 0·050)	0·35	9196
Identifies that newborns should only be given breast milk during first 6 months	Knowledge and attitudes	All respondents with a complete survey	0·039 (0·0073 to 0·070)	0·015	9196
Believes newborns should not be given chupón during first 6 months	Knowledge and attitudes	All respondents with a complete survey	0·16 (0·10 to 0·21)	<0·0001	9196
**Paternal involvement**					
Father accompanied mother to clinic for prenatal care visit at least once	Practice	All respondents with a complete survey and a child born since 2018-09-01 (after the end of the intervention) who received prenatal care	-0·016 (-0·20 to 0·17)	0·87	519
Father accompanied mother to seek medical care for pregnancy danger sign	Practice	All respondents with a complete survey and a child born since 2018-09-01 (after the end of the intervention) who experienced a prenatal care danger sign and sought care	0·16 (-0·081 to 0·41)	0·19	134

For peer review only - http://bmjopen.bmj.com/site/about/guidelines.xhtml

Father waited at birthplace during labor	Practice	All respondents with a complete survey and a child born since 2018-09-01 (after the end of the intervention)	-0·022 (-0·15 to 0·11)	0·74	540
Father accompanied mother to seek medical care for postnatal danger sign	Practice	All respondents with a complete survey and a child born since 2018-09-01 (after the end of the intervention) and sought care for postpartum problem	0·099 (-0·16 to 0·36)	0·46	93
Father sought medical care for newborn for postnatal danger sign	Practice	All respondents with a complete survey and a child born since 2018-09-01 (after the end of the intervention) and sought care for a postnatal problem	-0·010 (-0·30 to 0·28)	0·95	98
Father held child	Practice	All respondents with a complete survey and a child born since 2018-09-01 (after the end of the intervention)	0·045 (-0·028 to 0·12)	0·23	535
Father played with child	Practice	All respondents with a complete survey and a child born since 2018-09-01 (after the end of the intervention)	0·013 (-0·10 to 0·13)	0·83	535
Father took child to clinic when sick	Practice	All respondents with a complete survey and a child born since 2018-09-01 (after the end of the intervention)	0·091 (-0·040 to 0·22)	0·17	535
Believes father should accompany mother to prenatal care visits	Knowledge and attitudes	All respondents with a complete survey	0·038 (0·015 to 0·061)	0·0014	9158
Believes father should wait at birth location while mother gives birth	Knowledge and attitudes	All respondents with a complete survey	0·014 (-0·0044 to 0·032)	0·14	9156
Believes father should care for children when sick	Knowledge and attitudes	All respondents with a complete survey	0·0001 (-0·0059 to 0·0062)	0·97	9177
**Diarrhea management**					
Did not report diarrhea in past 4 weeks	Practice	All respondents with a complete survey	-0·0095 (-0·025 to 0·0063)	0·24	9182
Used ORT to treat diarrhea in past 4 weeks	Practice	All respondents with a complete survey who had diarrhea in past 4 weeks	-0·17 (-0·35 to 0·0073)	0·060	241
Used appropriate treatment for diarrhea in past 4 weeks[c]	Practice	All respondents with a complete survey who had diarrhea in past 4 weeks	-0·0029 (-0·14 to 0·14)	0·97	243
Child did not experience diarrhea past 4 weeks	Practice	All respondents with a complete survey and a new child <= 5 years old reported at wave 3 who had diarrhea in past 4 weeks	-0·0049 (-0·042 to 0·032)	0·80	3033
Child experienced diarrhea past 4 weeks, was given appropriate amount of liquids	Practice	All respondents with a complete survey and a new child <= 5 years old reported at wave 3 who had diarrhea in past 4 weeks	-0·16 (-0·32 to 0·0046)	0·057	310
Child experienced diarrhea past 4 weeks, was given appropriate amount of food	Practice	All respondents with a complete survey and a new child <= 5 years old reported at wave 3 who had diarrhea in past 4 weeks	-0·14 (-0·27 to -0·0099)	0·035	313
Child experienced diarrhea past 4 weeks, was given ORT	Practice	All respondents with a complete survey and a new child <= 5 years old reported at wave 3 who had diarrhea in past 4 weeks	0·14 (-0·035 to 0·32)	0·12	310
Child experienced diarrhea past 4 weeks, was given appropriate treatment	Practice	All respondents with a complete survey and a new child <= 5 years old reported at wave 3 who had diarrhea in past 4 weeks	0·13 (-0·038 to 0·29)	0·13	312
Child experienced diarrhea past 4 weeks, was given zinc 10-14 days	Practice	All respondents with a complete survey and a new child <= 5 years old reported at wave 3 who had diarrhea in past 4 weeks	..	..	..

For peer review only - http://bmjopen.bmj.com/site/about/guidelines.xhtml

Identified appropriate diarrhea treatment methods[d]	Knowledge and attitudes	All respondents with a complete survey	0·11 (0·056 to 0·16)	<0·0001	9195
Identified zinc 10-14 days as diarrhea treatment	Knowledge and attitudes	All respondents with a complete survey	0·033 (0·0097 to 0·056)	0·0053	9181
Identified zinc supplement as way to prevent diarrhea	Knowledge and attitudes	All respondents with a complete survey	0·073 (0·0073 to 0·14)	0·029	9196
Identified breastfeeding as way to prevent diarrhea	Knowledge and attitudes	All respondents with a complete survey	0·040 (-0·021 to 0·100)	0·20	9196
Correctly answered diarrhea treatment with zinc riddle	Intervention knowledge	All respondents with a complete survey	0·24 (0·19 to 0·30)	<0·0001	9197
**Respiratory illness**					
Did not report respiratory illness (coughing) for 2 weeks	Practice	All respondents with a complete survey	-0·013 (-0·046 to 0·020)	0·44	9188
Child did not have cough past 4 weeks	Practice	All respondents with a complete survey	-0·060 (-0·12 to -0·0046)	0·034	3034
Child had cough past 4 weeks, care was sought	Practice	All respondents with a complete survey and a new child <= 5 years old reported at wave 3 who had an illness with a cough in past 4 weeks	0·042 (-0·085 to 0·17)	0·52	647
Identified vaccination as way to prevent respiratory illness	Knowledge and attitudes	All respondents with a complete survey	-0·0017 (-0·039 to 0·036)	0·93	9196
Identified breastfeeding as way to prevent respiratory illness	Knowledge and attitudes	All respondents with a complete survey	0·030 (-0·021 to 0·081)	0·25	9196
Identified fever as d.s. for children with respiratory illness	Knowledge and attitudes	All respondents with a complete survey	0·015 (-0·012 to 0·043)	0·27	9187
Identified difficulty breathing as d.s. for children with respiratory illness	Knowledge and attitudes	All respondents with a complete survey	0·011 (-0·0049 to 0·027)	0·18	9187
Identified rapid breathing as d.s. for children with respiratory illness	Knowledge and attitudes	All respondents with a complete survey	0·0082 (-0·0024 to 0·019)	0·13	9187
**Reproductive health**					
Reported ever using birth control to delay or avoid pregnancy	Practice	All respondents with a complete survey	-0·0030 (-0·055 to 0·049)	0·91	9191
Reported currently using birth control	Practice	All respondents with a complete survey who are not pregnant and have ever used birth control	0·035 (-0·027 to 0·097)	0·27	3420
Believes woman should be at least 18 years of age to have her first child	Knowledge and attitudes	All respondents with a complete survey	0·012 (0·0048 to 0·019)	0·0011	8713
**Gender/reproductive norms**					
Birth location chosen either jointly or by woman	Practice	All respondents with a complete survey and a child born since 2018-09-01 (after the end of the intervention)	0·049 (-0·040 to 0·14)	0·28	540

For peer review only - http://bmjopen.bmj.com/site/about/guidelines.xhtml

Results based on sub-sample that completed baseline survey, N=9285 (household targeted n=2919, household not targeted n=6366). Baseline covariates included age, gender, education, marital status, indigenous status, and household wealth index. d.s.=Danger sign, ORT=Oral rehydration therapy. ··Model did not converge. Covariates were dropped sequentially if they predicted outcome perfectly. Standard errors are clustered at the village level. Robust 95% CIs in parentheses.

[a] 'Identified correct ways to provide newborn care' outcome was coded as correct if the respondent identified holding baby skin to skin, getting their health checked by a professional, immediately putting them to the breast, or making sure their diaper is clean and dry, and did not identify giving purgante, wrapping a fajero, giving a chupon, or bathing them in water right away.
[b] 'Identified proper cord care' outcome was coded as correct if respondent identified applying alcohol, applying iodine, keeping cord clean/dry, or applying peroxide, and did not identify applying oil, applying coffee, applying quina water, wrapping a fajero, applying powder, burning, applying ashes, applying a clamp, or applying thiomersal.
[c] 'Used appropriate treatment for diarrhea in past 4 weeks' outcome was coded as correct if respondent did any of the following: antibiotic, zinc, give extra food, give extra liquid, and did not do any of the following: anti-diarrhea medication, home remedy, laxative, deworming medication, chupon, massage, stop eating foods, stop taking liquids.
[d] 'Identified appropriate diarrhea treatment methods' outcome was coded as correct if respondent identified any of the following: antibiotic, zinc, ORS or Litrosol, give extra food, give extra liquid, get medical attention, and did not identify any of the following: anti-diarrhea medication, laxative, deworming medication, home remedy, chupon, massage, stop giving foods, stop giving liquids, or do nothing.

For peer review only

For peer review only - http://bmjopen.bmj.com/site/about/guidelines.xhtml

Table S5. Pooled total effect estimates of intervention on maternal, child, and neonatal outcomes, with adjustment for baseline outcome

	Outcome type	Respondent denominator	Pooled total effect estimate		N
			Effect size (95% CI)	p value	
**Prenatal care**					
Currently taking folic acid tablets (daily in past 7 days)	Practice	All women 15+ with a complete survey	0·0086 (-0·033 to 0·050)	0·69	5552
Made a birth plan in preparation for birth	Practice	All respondents with a complete survey and a child born since 2018-09-01 (after the end of the intervention)	-0·075 (-0·22 to 0·074)	0·32	299
Saved money in preparation for birth	Practice	All respondents with a complete survey and a child born since 2018-09-01 (after the end of the intervention)	0·044 (-0·092 to 0·18)	0·53	299
Sold animal in preparation for birth	Practice	All respondents with a complete survey and a child born since 2018-09-01 (after the end of the intervention)	NA	NA	NA
Sought prenatal care within 12 weeks	Practice	All respondents with a complete survey and a child born since 2018-09-01 (after the end of the intervention)	0·0028 (-0·16 to 0·16)	0·97	285
Identifies that women should take folic acid before pregnancy	Knowledge and attitudes	All respondents with a complete survey	0·045 (0·0062 to 0·083)	0·023	9283
Identifies that women should seek prenatal care first 12 weeks of pregnancy	Knowledge and attitudes	All respondents with a complete survey	0·024 (-0·0017 to 0·050)	0·067	9282
Identifies accompanying woman to prenatal care visits as method of support during pregnancy	Knowledge and attitudes	All respondents with a complete survey	NA	NA	NA
Identifies ensuring that woman eats well as method of support during pregnancy	Knowledge and attitudes	All respondents with a complete survey	NA	NA	NA
Identifies ensuring that woman rests 1 hour per day well as method of support during pregnancy	Knowledge and attitudes	All respondents with a complete survey	NA	NA	NA
Identifies avoiding violence as method of support during pregnancy	Knowledge and attitudes	All respondents with a complete survey	NA	NA	NA
Identifies encouraging women to take vitamins as method of support during pregnancy	Knowledge and attitudes	All respondents with a complete survey	NA	NA	NA
Identifies helping woman with house work/child care as method of support during pregnancy	Knowledge and attitudes	All respondents with a complete survey	NA	NA	NA
Identifies saving animals to sell as method of preparing for birth expenses	Knowledge and attitudes	All respondents with a complete survey	NA	NA	NA

For peer review only - http://bmjopen.bmj.com/site/about/guidelines.xhtml

Identifies knowing cost of trip to maternal clinic as method of preparing for birth expenses	Knowledge and attitudes	All respondents with a complete survey	NA	NA	NA
Identifies having a savings plan as method of preparing for birth expenses	Knowledge and attitudes	All respondents with a complete survey	NA	NA	NA
Correctly answered prenatal care knowledge riddle	Intervention knowledge	All respondents with a complete survey	NA	NA	NA
**Facility based birth**					
Gave birth in health facility	Practice	All respondents with a complete survey and a child born since 2018-09-01 (after the end of the intervention)	-0·055 (-0·20 to 0·092)	0·46	299
Believes health facility is best place to give birth	Knowledge and attitudes	All respondents with a complete survey	0·0065 (-0·023 to 0·036)	0·66	9283
**Pregnancy danger signs**					
Woman sought medical care for pregnancy danger sign	Practice	All respondents with a complete survey and a child born since 2018-09-01 (after the end of the intervention) who experienced a prenatal care danger sign	-0·34 (-0·81 to 0·13)	0·15	57
Identified bleeding as pregnancy d.s.	Knowledge and attitudes	All respondents with a complete survey	0·048 (0·0048 to 0·091)	0·030	9279
Identified seizure as pregnancy d.s.	Knowledge and attitudes	All respondents with a complete survey	0·0026 (-0·0071 to 0·012)	0·60	9279
Identified headache as pregnancy d.s.	Knowledge and attitudes	All respondents with a complete survey	0·045 (-0·0051 to 0·094)	0·079	9279
Identified ringing in ears as pregnancy d.s.	Knowledge and attitudes	All respondents with a complete survey	0·0050 (-0·0062 to 0·016)	0·38	9279
Identified dizziness as pregnancy d.s.	Knowledge and attitudes	All respondents with a complete survey	-0·0068 (-0·056 to 0·043)	0·79	9279
Identified difficulty urinating as pregnancy d.s.	Knowledge and attitudes	All respondents with a complete survey	-0·0001 (-0·0085 to 0·0084)	0·99	9279
Identified reduced or absent fetal movement as pregnancy d.s.	Knowledge and attitudes	All respondents with a complete survey	0·014 (-0·0055 to 0·034)	0·16	9279
Identified water breaking as pregnancy d.s.	Knowledge and attitudes	All respondents with a complete survey	-0·0006 (-0·0083 to 0·0071)	0·89	9279
Identified fever as pregnancy d.s.	Knowledge and attitudes	All respondents with a complete survey	0·047 (0·010 to 0·084)	0·013	9279
Identified swelling of face/hands/feet as pregnancy d.s.	Knowledge and attitudes	All respondents with a complete survey	0·037 (0·0039 to 0·070)	0·028	9279
**Postnatal care for mother**					
Mother had health checked by professional within 3 days of birth	Practice	All respondents with a complete survey and a child born since 2018-09-01 (after the end of the intervention)	0·024 (-0·18 to 0·23)	0·82	293

For peer review only - http://bmjopen.bmj.com/site/about/guidelines.xhtml

Mother had health checked by professional within 7 days of birth	Practice	All respondents with a complete survey and a child born since 2018-09-01 (after the end of the intervention)	0·0051 (-0·22 to 0·24)	0·97	293
Mother sought medical care for postnatal danger sign	Practice	All respondents with a complete survey and a child born since 2018-09-01 (after the end of the intervention)	0·21 (-0·15 to 0·57)	0·25	34
Identifies that mother should receive postnatal medical check-up within 3 days of birth	Knowledge and attitudes	All respondents with a complete survey	0·092 (0·045 to 0·14)	0·0001	9279
Identifies that mother should receive postnatal medical check-up within 7 days of birth	Knowledge and attitudes	All respondents with a complete survey	0·054 (0·0031 to 0·11)	0·038	9279
Identified heavy vaginal bleeding as postnatal d.s.	Knowledge and attitudes	All respondents with a complete survey	0·060 (0·021 to 0·098)	0·0024	9279
Identified fever as postnatal d.s.	Knowledge and attitudes	All respondents with a complete survey	0·059 (0·0096 to 0·11)	0·019	9279
Identified vaginal discharge as postnatal d.s.	Knowledge and attitudes	All respondents with a complete survey	0·0032 (-0·012 to 0·018)	0·67	9279
Identified headache as postnatal d.s.	Knowledge and attitudes	All respondents with a complete survey	0·031 (-0·0088 to 0·071)	0·13	9279
Identified convulsions or fits as postnatal d.s.	Knowledge and attitudes	All respondents with a complete survey	0·0003 (-0·0055 to 0·0062)	0·91	9279
**Postnatal care for newborn**					
Newborn had health checked by professional within 3 days of birth	Practice	All respondents with a complete survey and a child born since 2018-09-01 (after the end of the intervention)	0·091 (-0·095 to 0·28)	0·34	297
Newborn had health checked by professional within 7 days of birth	Practice	All respondents with a complete survey and a child born since 2018-09-01 (after the end of the intervention)	0·043 (-0·18 to 0·27)	0·71	297
Newborn experienced health problem in first month, care was sought	Practice	All respondents with a complete survey and a child born since 2018-09-01 (after the end of the intervention)	-0·031 (-0·57 to 0·51)	0·91	25
Identified correct ways to provide newborn care[a]	Knowledge and attitudes	All respondents with a complete survey	0·026 (0·010 to 0·042)	0·0011	9282
Identified fever as newborn d.s.	Knowledge and attitudes	All respondents with a complete survey	0·015 (-0·033 to 0·062)	0·54	9281
Identified diarrhea as newborn d.s.	Knowledge and attitudes	All respondents with a complete survey	0·020 (-0·023 to 0·062)	0·36	9281
Identified difficulty breathing as newborn d.s.	Knowledge and attitudes	All respondents with a complete survey	0·011 (-0·026 to 0·049)	0·55	9281
Identified vomiting as newborn d.s.	Knowledge and attitudes	All respondents with a complete survey	0·013 (-0·017 to 0·042)	0·39	9281
Identified poor appetite as newborn d.s.	Knowledge and attitudes	All respondents with a complete survey	-0·0039 (-0·014 to 0·0067)	0·47	9281
Identified redness/bleeding around cord as newborn d.s.	Knowledge and attitudes	All respondents with a complete survey	0·017 (-0·0038 to 0·039)	0·11	9281
Identified pus in cord as newborn d.s.	Knowledge and attitudes	All respondents with a complete survey	0·015 (-0·0014 to 0·031)	0·074	9281
Identified cold skin as newborn d.s.	Knowledge and attitudes	All respondents with a complete survey	0·0018 (-0·0076 to 0·011)	0·70	9281

For peer review only - http://bmjopen.bmj.com/site/about/guidelines.xhtml

Identified cough as newborn d.s.	Knowledge and attitudes	All respondents with a complete survey	-0·018 (-0·066 to 0·031)	0·48	9281
Identified cold as newborn d.s.	Knowledge and attitudes	All respondents with a complete survey	-0·025 (-0·080 to 0·030)	0·37	9281
Identified problems latching as newborn d.s.	Knowledge and attitudes	All respondents with a complete survey	-0·0025 (-0·017 to 0·012)	0·72	9279
Identified pneumonia as newborn d.s.	Knowledge and attitudes	All respondents with a complete survey	NA	NA	NA
Did not use harmful substances to treat cord stump	Practice	All respondents with a complete survey and a child born since 2018-09-01 (after the end of the intervention)	0·099 (-0·082 to 0·28)	0·29	289
Did not wrap fajero around newborn in first 7 days after birth	Practice	All respondents with a complete survey and a child born since 2018-09-01 (after the end of the intervention)	0·25 (0·043 to 0·46)	0·018	298
Identified proper cord care methods[b]	Knowledge and attitudes	All respondents with a complete survey	0·094 (0·047 to 0·14)	0·0001	9281
Correctly answered proper cord care riddle	Intervention knowledge	All respondents with a complete survey	NA	NA	NA
Kept newborn wrapped first 7 days after birth	Practice	All respondents with a complete survey and a child born since 2018-09-01 (after the end of the intervention)	-0·041 (-0·12 to 0·038)	0·31	293
Held newborn skin-to-skin during first month after birth	Practice	All respondents with a complete survey and a child born since 2018-09-01 (after the end of the intervention)	0·21 (-0·099 to 0·51)	0·19	72
**Breastfeeding**					
Exclusively breastfed child first 6 months	Practice	All respondents with a complete survey and a child born since 2018-09-01 (after the end of the intervention)	-0·15 (-0·33 to 0·035)	0·11	298
Exclusively breastfed child first 6 months without giving chupón	Practice	All respondents with a complete survey and a child born since 2018-09-01 (after the end of the intervention)	-0·079 (-0·29 to 0·13)	0·46	299
Breastfed immediately after birth	Practice	All respondents with a complete survey and a child born since 2018-09-01 (after the end of the intervention) who reported a natural birth	-0·017 (-0·15 to 0·11)	0·80	230
Identifies that newborn should be breastfed immediately after birth	Knowledge and attitudes	All respondents with a complete survey	0·0088 (-0·027 to 0·044)	0·63	9282
Identifies that newborns should only be given breast milk during first 6 months	Knowledge and attitudes	All respondents with a complete survey	0·038 (0·0086 to 0·068)	0·012	9282
Believes newborns should not be given chupón during first 6 months	Knowledge and attitudes	All respondents with a complete survey	0·14 (0·079 to 0·19)	<0·0001	9282
**Paternal involvement**					
Father accompanied mother to clinic for prenatal care visit at least once	Practice	All respondents with a complete survey and a child born since 2018-09-01 (after the end of the intervention) who received prenatal care	0·16 (-0·049 to 0·36)	0·14	276
Father accompanied mother to seek medical care for pregnancy danger sign	Practice	All respondents with a complete survey and a child born since 2018-09-01 (after the end of the intervention) who experienced a prenatal care danger sign and sought care	-0·017 (-0·71 to 0·67)	0·96	25

For peer review only - http://bmjopen.bmj.com/site/about/guidelines.xhtml

Father waited at birthplace during labor	Practice	All respondents with a complete survey and a child born since 2018-09-01 (after the end of the intervention)	-0·013 (-0·17 to 0·14)	0·87	299
Father accompanied mother to seek medical care for postnatal danger sign	Practice	All respondents with a complete survey and a child born since 2018-09-01 (after the end of the intervention) and sought care for postpartum problem	0·28 (-0·061 to 0·63)	0·11	14
Father sought medical care for newborn for postnatal danger sign	Practice	All respondents with a complete survey and a child born since 2018-09-01 (after the end of the intervention) and sought care for a postnatal problem	-0·062 (-0·64 to 0·51)	0·83	16
Father held child	Practice	All respondents with a complete survey and a child born since 2018-09-01 (after the end of the intervention)	-0·0032 (-0·12 to 0·11)	0·96	269
Father played with child	Practice	All respondents with a complete survey and a child born since 2018-09-01 (after the end of the intervention)	0·036 (-0·10 to 0·17)	0·61	275
Father took child to clinic when sick	Practice	All respondents with a complete survey and a child born since 2018-09-01 (after the end of the intervention)	0·068 (-0·11 to 0·25)	0·46	275
Believes father should accompany mother to prenatal care visits	Knowledge and attitudes	All respondents with a complete survey	0·043 (0·020 to 0·066)	0·00020	9214
Believes father should wait at birth location while mother gives birth	Knowledge and attitudes	All respondents with a complete survey	0·014 (-0·0049 to 0·032)	0·15	9225
Believes father should care for children when sick	Knowledge and attitudes	All respondents with a complete survey	-0·0010 (-0·0082 to 0·0061)	0·78	9266
**Diarrhea management**					
Did not report diarrhea in past 4 weeks	Practice	All respondents with a complete survey	-0·0099 (-0·026 to 0·0065)	0·24	9282
Used ORT to treat diarrhea in past 4 weeks	Practice	All respondents with a complete survey who had diarrhea in past 4 weeks	0·21 (-0·28 to 0·70)	0·40	23
Used appropriate treatment for diarrhea in past 4 weeks[c]	Practice	All respondents with a complete survey who had diarrhea in past 4 weeks	-0·24 (-0·48 to 0·0082)	0·058	23
Child did not experience diarrhea past 4 weeks	Practice	All respondents with a complete survey and a new child <= 5 years old reported at wave 3 who had diarrhea in past 4 weeks	-0·011 (-0·053 to 0·031)	0·60	2377
Child experienced diarrhea past 4 weeks, was given appropriate amount of liquids	Practice	All respondents with a complete survey and a new child <= 5 years old reported at wave 3 who had diarrhea in past 4 weeks	1·9 (1·5 to 2·3)	<0·0001	49
Child experienced diarrhea past 4 weeks, was given appropriate amount of food	Practice	All respondents with a complete survey and a new child <= 5 years old reported at wave 3 who had diarrhea in past 4 weeks	1·2 (0·67 to 1·7)	<0·0001	50
Child experienced diarrhea past 4 weeks, was given ORT	Practice	All respondents with a complete survey and a new child <= 5 years old reported at wave 3 who had diarrhea in past 4 weeks	-0·029 (-0·58 to 0·52)	0·92	50
Child experienced diarrhea past 4 weeks, was given appropriate treatment	Practice	All respondents with a complete survey and a new child <= 5 years old reported at wave 3 who had diarrhea in past 4 weeks	1·1 (0·60 to 1·5)	<0·0001	55
Child experienced diarrhea past 4 weeks, was given zinc 10-14 days	Practice	All respondents with a complete survey and a new child <= 5 years old reported at wave 3 who had diarrhea in past 4 weeks	..	..	..

For peer review only - http://bmjopen.bmj.com/site/about/guidelines.xhtml

Identified appropriate diarrhea treatment methods[d]	Knowledge and attitudes	All respondents with a complete survey	0·093 (0·038 to 0·15)	0·0009	9280
Identified zinc 10-14 days as diarrhea treatment	Knowledge and attitudes	All respondents with a complete survey	0·034 (0·010 to 0·057)	0·0046	9257
Identified zinc supplement as way to prevent diarrhea	Knowledge and attitudes	All respondents with a complete survey	0·070 (0·0004 to 0·14)	0·049	9282
Identified breastfeeding as way to prevent diarrhea	Knowledge and attitudes	All respondents with a complete survey	0·041 (-0·026 to 0·11)	0·23	9282
Correctly answered diarrhea treatment with zinc riddle	Intervention knowledge	All respondents with a complete survey	NA	NA	NA
**Respiratory illness**					
Did not report respiratory illness (coughing) for 2 weeks	Practice	All respondents with a complete survey	-0·021 (-0·057 to 0·014)	0·24	9283
Child did not have cough past 4 weeks	Practice	All respondents with a complete survey	-0·033 (-0·097 to 0·031)	0·31	2379
Child had cough past 4 weeks, care was sought	Practice	All respondents with a complete survey and a new child <= 5 years old reported at wave 3 who had an illness with a cough in past 4 weeks	0·0043 (-0·24 to 0·24)	0·97	196
Identified vaccination as way to prevent respiratory illness	Knowledge and attitudes	All respondents with a complete survey	-0·0075 (-0·047 to 0·032)	0·71	9282
Identified breastfeeding as way to prevent respiratory illness	Knowledge and attitudes	All respondents with a complete survey	0·027 (-0·028 to 0·081)	0·34	9282
Identified fever as d.s. for children with respiratory illness	Knowledge and attitudes	All respondents with a complete survey	0·0090 (-0·021 to 0·039)	0·56	9282
Identified difficulty breathing as d.s. for children with respiratory illness	Knowledge and attitudes	All respondents with a complete survey	0·0066 (-0·0098 to 0·023)	0·43	9282
Identified rapid breathing as d.s. for children with respiratory illness	Knowledge and attitudes	All respondents with a complete survey	0·0069 (-0·0044 to 0·018)	0·23	9282
**Reproductive health**					
Reported ever using birth control to delay or avoid pregnancy	Practice	All respondents with a complete survey	0·011 (-0·038 to 0·060)	0·67	8529
Reported currently using birth control	Practice	All respondents with a complete survey who are not pregnant and have ever used birth control	0·060 (-0·038 to 0·16)	0·23	1948
Believes woman should be at least 18 years of age to have her first child	Knowledge and attitudes	All respondents with a complete survey	0·012 (0·0029 to 0·020)	0·0087	8675
**Gender/reproductive norms**					
Birth location chosen either jointly or by woman	Practice	All respondents with a complete survey and a child born since 2018-09-01 (after the end of the intervention)	0·042 (-0·10 to 0·19)	0·57	299

For peer review only - http://bmjopen.bmj.com/site/about/guidelines.xhtml

Results based on sub-sample that completed baseline survey, N=9285 (household targeted n=2919, household not targeted n=6366). Models included covariate for the related outcome assessed at baseline survey. d.s.=Danger sign, NA=Not applicable, ORT=Oral rehydration therapy. ··Model did not converge. Results with NA could not be assessed because outcome was not included in baseline survey. Standard errors are clustered at the village level. Robust 95% CIs in parentheses.

[a] 'Identified correct ways to provide newborn care' outcome was coded as correct if the respondent identified holding baby skin to skin, getting their health checked by a professional, immediately putting them to the breast, or making sure their diaper is clean and dry, and did not identify giving purgante, wrapping a fajero, giving a chupon, or bathing them in water right away.
[b] 'Identified proper cord care' outcome was coded as correct if respondent identified applying alcohol, applying iodine, keeping cord clean/dry, or applying peroxide, and did not identify applying oil, applying coffee, applying quina water, wrapping a fajero, applying powder, burning, applying ashes, applying a clamp, or applying thiomersal.
[c] 'Used appropriate treatment for diarrhea in past 4 weeks' outcome was coded as correct if respondent did any of the following: antibiotic, zinc, give extra food, give extra liquid, and did not do any of the following: anti-diarrhea medication, home remedy, laxative, deworming medication, chupon, massage, stop eating foods, stop taking liquids.
[d] 'Identified appropriate diarrhea treatment methods' outcome was coded as correct if respondent identified any of the following: antibiotic, zinc, ORS or Litrosol, give extra food, give extra liquid, get medical attention, and did not identify any of the following: anti-diarrhea medication, laxative, deworming medication, home remedy, chupon, massage, stop giving foods, stop giving liquids, or do nothing.

For peer review only - http://bmjopen.bmj.com/site/about/guidelines.xhtml

Table S6. Pooled total effect estimates of intervention on maternal, child, and neonatal outcomes, with adjustment for baseline demographic characteristics and baseline outcome

	Outcome type	Respondent denominator	Pooled total effect estimate		N
			Effect size (95% CI)	p value	
**Prenatal care**					
Currently taking folic acid tablets (daily in past 7 days)	Practice	All women 15+ with a complete survey	-0·0013 (-0·041 to 0·039)	0·95	5492
Made a birth plan in preparation for birth	Practice	All respondents with a complete survey and a child born since 2018-09-01 (after the end of the intervention)	-0·083 (-0·22 to 0·051)	0·22	291
Saved money in preparation for birth	Practice	All respondents with a complete survey and a child born since 2018-09-01 (after the end of the intervention)	-0·0003 (-0·13 to 0·13)	1·00	295
Sold animal in preparation for birth	Practice	All respondents with a complete survey and a child born since 2018-09-01 (after the end of the intervention)	NA	NA	NA
Sought prenatal care within 12 weeks	Practice	All respondents with a complete survey and a child born since 2018-09-01 (after the end of the intervention)	0·030 (-0·11 to 0·17)	0·67	277
Identifies that women should take folic acid before pregnancy	Knowledge and attitudes	All respondents with a complete survey	0·058 (0·026 to 0·091)	0·0005	9174
Identifies that women should seek prenatal care first 12 weeks of pregnancy	Knowledge and attitudes	All respondents with a complete survey	0·031 (0·0090 to 0·054)	0·0059	9173
Identifies accompanying woman to prenatal care visits as method of support during pregnancy	Knowledge and attitudes	All respondents with a complete survey	NA	NA	NA
Identifies ensuring that woman eats well as method of support during pregnancy	Knowledge and attitudes	All respondents with a complete survey	NA	NA	NA
Identifies ensuring that woman rests 1 hour per day well as method of support during pregnancy	Knowledge and attitudes	All respondents with a complete survey	NA	NA	NA
Identifies avoiding violence as method of support during pregnancy	Knowledge and attitudes	All respondents with a complete survey	NA	NA	NA
Identifies encouraging women to take vitamins as method of support during pregnancy	Knowledge and attitudes	All respondents with a complete survey	NA	NA	NA
Identifies helping woman with house work/child care as method of support during pregnancy	Knowledge and attitudes	All respondents with a complete survey	NA	NA	NA
Identifies saving animals to sell as method of preparing for birth expenses	Knowledge and attitudes	All respondents with a complete survey	NA	NA	NA

For peer review only

For peer review only - http://bmjopen.bmj.com/site/about/guidelines.xhtml

Identifies knowing cost of trip to maternal clinic as method of preparing for birth expenses	Knowledge and attitudes	All respondents with a complete survey	NA	NA	NA
Identifies having a savings plan as method of preparing for birth expenses	Knowledge and attitudes	All respondents with a complete survey	NA	NA	NA
Correctly answered prenatal care knowledge riddle	Intervention knowledge	All respondents with a complete survey	NA	NA	NA
**Facility based birth**					
Gave birth in health facility	Practice	All respondents with a complete survey and a child born since 2018-09-01 (after the end of the intervention)	0·0030 (-0·13 to 0·14)	0·97	295
Believes health facility is best place to give birth	Knowledge and attitudes	All respondents with a complete survey	0·018 (-0·0034 to 0·040)	0·099	9165
**Pregnancy danger signs**					
Woman sought medical care for pregnancy danger sign	Practice	All respondents with a complete survey and a child born since 2018-09-01 (after the end of the intervention) who experienced a prenatal care danger sign	-0·26 (-0·76 to 0·24)	0·30	55
Identified bleeding as pregnancy d.s.	Knowledge and attitudes	All respondents with a complete survey	0·051 (0·012 to 0·090)	0·0099	9170
Identified seizure as pregnancy d.s.	Knowledge and attitudes	All respondents with a complete survey	0·0020 (-0·0077 to 0·012)	0·69	9161
Identified headache as pregnancy d.s.	Knowledge and attitudes	All respondents with a complete survey	0·038 (-0·0085 to 0·084)	0·11	9170
Identified ringing in ears as pregnancy d.s.	Knowledge and attitudes	All respondents with a complete survey	0·0031 (-0·0064 to 0·013)	0·52	9146
Identified dizziness as pregnancy d.s.	Knowledge and attitudes	All respondents with a complete survey	-0·0030 (-0·050 to 0·044)	0·90	9170
Identified difficulty urinating as pregnancy d.s.	Knowledge and attitudes	All respondents with a complete survey	-0·0018 (-0·0094 to 0·0059)	0·65	9146
Identified reduced or absent fetal movement as pregnancy d.s.	Knowledge and attitudes	All respondents with a complete survey	0·014 (-0·0060 to 0·034)	0·17	9146
Identified water breaking as pregnancy d.s.	Knowledge and attitudes	All respondents with a complete survey	-0·0006 (-0·0079 to 0·0066)	0·87	9146
Identified fever as pregnancy d.s.	Knowledge and attitudes	All respondents with a complete survey	0·041 (0·0031 to 0·079)	0·034	9170
Identified swelling of face/hands/feet as pregnancy d.s.	Knowledge and attitudes	All respondents with a complete survey	0·032 (0·0030 to 0·061)	0·031	9170
**Postnatal care for mother**					
Mother had health checked by professional within 3 days of birth	Practice	All respondents with a complete survey and a child born since 2018-09-01 (after the end of the intervention)	-0·035 (-0·22 to 0·15)	0·70	289

For peer review only - http://bmjopen.bmj.com/site/about/guidelines.xhtml

For peer review only

Mother had health checked by professional within 7 days of birth	Practice	All respondents with a complete survey and a child born since 2018-09-01 (after the end of the intervention)	-0·032 (-0·23 to 0·17)	0·75	289
Mother sought medical care for postnatal danger sign	Practice	All respondents with a complete survey and a child born since 2018-09-01 (after the end of the intervention)	0·38 (0·062 to 0·69)	0·019	33
Identifies that mother should receive postnatal medical check-up within 3 days of birth	Knowledge and attitudes	All respondents with a complete survey	0·072 (0·030 to 0·11)	0·0007	9170
Identifies that mother should receive postnatal medical check-up within 7 days of birth	Knowledge and attitudes	All respondents with a complete survey	0·037 (-0·011 to 0·085)	0·13	9170
Identified heavy vaginal bleeding as postnatal d.s.	Knowledge and attitudes	All respondents with a complete survey	0·066 (0·028 to 0·10)	0·0006	9170
Identified fever as postnatal d.s.	Knowledge and attitudes	All respondents with a complete survey	0·060 (0·011 to 0·11)	0·017	9170
Identified vaginal discharge as postnatal d.s.	Knowledge and attitudes	All respondents with a complete survey	0·0045 (-0·010 to 0·019)	0·55	9155
Identified headache as postnatal d.s.	Knowledge and attitudes	All respondents with a complete survey	0·029 (-0·0085 to 0·066)	0·13	9170
Identified convulsions or fits as postnatal d.s.	Knowledge and attitudes	All respondents with a complete survey	0·00 (-0·0056 to 0·0056)	0·99	8958
**Postnatal care for newborn**					
Newborn had health checked by professional within 3 days of birth	Practice	All respondents with a complete survey and a child born since 2018-09-01 (after the end of the intervention)	0·028 (-0·13 to 0·18)	0·72	293
Newborn had health checked by professional within 7 days of birth	Practice	All respondents with a complete survey and a child born since 2018-09-01 (after the end of the intervention)	0·022 (-0·16 to 0·20)	0·81	293
Newborn experienced health problem in first month, care was sought	Practice	All respondents with a complete survey and a child born since 2018-09-01 (after the end of the intervention)	-0·014 (-0·014 to -0·014)	<0·0001	23
Identified correct ways to provide newborn care[a]	Knowledge and attitudes	All respondents with a complete survey	0·023 (0·0099 to 0·037)	0·0006	9149
Identified fever as newborn d.s.	Knowledge and attitudes	All respondents with a complete survey	0·0033 (-0·043 to 0·049)	0·89	9172
Identified diarrhea as newborn d.s.	Knowledge and attitudes	All respondents with a complete survey	0·0039 (-0·036 to 0·043)	0·85	9172
Identified difficulty breathing as newborn d.s.	Knowledge and attitudes	All respondents with a complete survey	0·0097 (-0·025 to 0·045)	0·59	9148
Identified vomiting as newborn d.s.	Knowledge and attitudes	All respondents with a complete survey	0·0085 (-0·021 to 0·038)	0·58	9163
Identified poor appetite as newborn d.s.	Knowledge and attitudes	All respondents with a complete survey	-0·0049 (-0·015 to 0·0048)	0·32	9148
Identified redness/bleeding around cord as newborn d.s.	Knowledge and attitudes	All respondents with a complete survey	0·020 (-0·0008 to 0·042)	0·059	9172
Identified pus in cord as newborn d.s.	Knowledge and attitudes	All respondents with a complete survey	0·015 (-0·0014 to 0·031)	0·073	9163
Identified cold skin as newborn d.s.	Knowledge and attitudes	All respondents with a complete survey	0·0023 (-0·0069 to 0·011)	0·63	9148

For peer review only - http://bmjopen.bmj.com/site/about/guidelines.xhtml

Identified cough as newborn d.s.	Knowledge and attitudes	All respondents with a complete survey	-0·019 (-0·069 to 0·031)	0·45	9172
Identified cold as newborn d.s.	Knowledge and attitudes	All respondents with a complete survey	-0·016 (-0·067 to 0·035)	0·55	9172
Identified problems latching as newborn d.s.	Knowledge and attitudes	All respondents with a complete survey	-0·0013 (-0·015 to 0·012)	0·85	9146
Identified pneumonia as newborn d.s.	Knowledge and attitudes	All respondents with a complete survey	NA	NA	NA
Did not use harmful substances to treat cord stump	Practice	All respondents with a complete survey and a child born since 2018-09-01 (after the end of the intervention)	0·11 (-0·061 to 0·29)	0·20	285
Did not wrap fajero around newborn in first 7 days after birth	Practice	All respondents with a complete survey and a child born since 2018-09-01 (after the end of the intervention)	0·30 (0·11 to 0·48)	0·0018	290
Identified proper cord care methods[b]	Knowledge and attitudes	All respondents with a complete survey	0·094 (0·048 to 0·14)	0·0001	9172
Correctly answered proper cord care riddle	Intervention knowledge	All respondents with a complete survey	NA	NA	NA
Kept newborn wrapped first 7 days after birth	Practice	All respondents with a complete survey and a child born since 2018-09-01 (after the end of the intervention)	..	..	..
Held newborn skin-to-skin during first month after birth	Practice	All respondents with a complete survey and a child born since 2018-09-01 (after the end of the intervention)	0·051 (-0·21 to 0·31)	0·70	70
**Breastfeeding**					
Exclusively breastfed child first 6 months	Practice	All respondents with a complete survey and a child born since 2018-09-01 (after the end of the intervention)	-0·12 (-0·26 to 0·014)	0·079	294
Exclusively breastfed child first 6 months without giving chupón	Practice	All respondents with a complete survey and a child born since 2018-09-01 (after the end of the intervention)	-0·062 (-0·23 to 0·11)	0·47	295
Breastfed immediately after birth	Practice	All respondents with a complete survey and a child born since 2018-09-01 (after the end of the intervention) who reported a natural birth	-0·049 (-0·18 to 0·085)	0·47	217
Identifies that newborn should be breastfed immediately after birth	Knowledge and attitudes	All respondents with a complete survey	0·011 (-0·023 to 0·045)	0·53	9173
Identifies that newborns should only be given breast milk during first 6 months	Knowledge and attitudes	All respondents with a complete survey	0·038 (0·0075 to 0·069)	0·015	9173
Believes newborns should not be given chupón during first 6 months	Knowledge and attitudes	All respondents with a complete survey	0·16 (0·10 to 0·21)	<0·0001	9173
**Paternal involvement**					
Father accompanied mother to clinic for prenatal care visit at least once	Practice	All respondents with a complete survey and a child born since 2018-09-01 (after the end of the intervention) who received prenatal care	0·079 (-0·10 to 0·26)	0·39	272
Father accompanied mother to seek medical care for pregnancy danger sign	Practice	All respondents with a complete survey and a child born since 2018-09-01 (after the end of the intervention) who experienced a prenatal care danger sign and sought care	..	..	..

For peer review only - http://bmjopen.bmj.com/site/about/guidelines.xhtml

Father waited at birthplace during labor	Practice	All respondents with a complete survey and a child born since 2018-09-01 (after the end of the intervention)	-0·021 (-0·18 to 0·13)	0·79	295
Father accompanied mother to seek medical care for postnatal danger sign	Practice	All respondents with a complete survey and a child born since 2018-09-01 (after the end of the intervention) and sought care for postpartum problem	..	..	..
Father sought medical care for newborn for postnatal danger sign	Practice	All respondents with a complete survey and a child born since 2018-09-01 (after the end of the intervention) and sought care for a postnatal problem	..	..	..
Father held child	Practice	All respondents with a complete survey and a child born since 2018-09-01 (after the end of the intervention)	0·058 (-0·042 to 0·16)	0·25	265
Father played with child	Practice	All respondents with a complete survey and a child born since 2018-09-01 (after the end of the intervention)	0·077 (-0·037 to 0·19)	0·18	271
Father took child to clinic when sick	Practice	All respondents with a complete survey and a child born since 2018-09-01 (after the end of the intervention)	0·093 (-0·077 to 0·26)	0·28	271
Believes father should accompany mother to prenatal care visits	Knowledge and attitudes	All respondents with a complete survey	0·039 (0·017 to 0·061)	0·0006	9105
Believes father should wait at birth location while mother gives birth	Knowledge and attitudes	All respondents with a complete survey	0·014 (-0·0044 to 0·032)	0·14	9108
Believes father should care for children when sick	Knowledge and attitudes	All respondents with a complete survey	0·0001 (-0·0059 to 0·0062)	0·97	9149
**Diarrhea management**					
Did not report diarrhea in past 4 weeks	Practice	All respondents with a complete survey	-0·0095 (-0·025 to 0·0062)	0·24	9160
Used ORT to treat diarrhea in past 4 weeks	Practice	All respondents with a complete survey who had diarrhea in past 4 weeks	0·036 (0·036 to 0·036)	<0·0001	23
Used appropriate treatment for diarrhea in past 4 weeks[c]	Practice	All respondents with a complete survey who had diarrhea in past 4 weeks	..	..	..
Child did not experience diarrhea past 4 weeks	Practice	All respondents with a complete survey and a new child <= 5 years old reported at wave 3 who had diarrhea in past 4 weeks	-0·0093 (-0·052 to 0·034)	0·67	2346
Child experienced diarrhea past 4 weeks, was given appropriate amount of liquids	Practice	All respondents with a complete survey and a new child <= 5 years old reported at wave 3 who had diarrhea in past 4 weeks	1·9 (1·2 to 2·6)	<0·0001	43
Child experienced diarrhea past 4 weeks, was given appropriate amount of food	Practice	All respondents with a complete survey and a new child <= 5 years old reported at wave 3 who had diarrhea in past 4 weeks	0·95 (0·52 to 1·4)	<0·0001	43
Child experienced diarrhea past 4 weeks, was given ORT	Practice	All respondents with a complete survey and a new child <= 5 years old reported at wave 3 who had diarrhea in past 4 weeks	1·3 (0·73 to 1·8)	<0·0001	43
Child experienced diarrhea past 4 weeks, was given appropriate treatment	Practice	All respondents with a complete survey and a new child <= 5 years old reported at wave 3 who had diarrhea in past 4 weeks	0·59 (0·19 to 0·98)	0·0034	48
Child experienced diarrhea past 4 weeks, was given zinc 10-14 days	Practice	All respondents with a complete survey and a new child <= 5 years old reported at wave 3 who had diarrhea in past 4 weeks	..	..	..

For peer review only - http://bmjopen.bmj.com/site/about/guidelines.xhtml

Identified appropriate diarrhea treatment methods[d]	Knowledge and attitudes	All respondents with a complete survey	0·11 (0·055 to 0·16)	0·0001	9171
Identified zinc 10-14 days as diarrhea treatment	Knowledge and attitudes	All respondents with a complete survey	0·032 (0·0095 to 0·055)	0·0056	9133
Identified zinc supplement as way to prevent diarrhea	Knowledge and attitudes	All respondents with a complete survey	0·073 (0·0071 to 0·14)	0·030	9173
Identified breastfeeding as way to prevent diarrhea	Knowledge and attitudes	All respondents with a complete survey	0·039 (-0·021 to 0·100)	0·20	9173
Correctly answered diarrhea treatment with zinc riddle	Intervention knowledge	All respondents with a complete survey	NA	NA	NA
**Respiratory illness**					
Did not report respiratory illness (coughing) for 2 weeks	Practice	All respondents with a complete survey	-0·017 (-0·050 to 0·017)	0·33	9167
Child did not have cough past 4 weeks	Practice	All respondents with a complete survey	-0·038 (-0·10 to 0·026)	0·25	2348
Child had cough past 4 weeks, care was sought	Practice	All respondents with a complete survey and a new child <= 5 years old reported at wave 3 who had an illness with a cough in past 4 weeks	-0·0071 (-0·24 to 0·23)	0·95	195
Identified vaccination as way to prevent respiratory illness	Knowledge and attitudes	All respondents with a complete survey	-0·0012 (-0·039 to 0·036)	0·95	9173
Identified breastfeeding as way to prevent respiratory illness	Knowledge and attitudes	All respondents with a complete survey	0·031 (-0·020 to 0·081)	0·24	9173
Identified fever as d.s. for children with respiratory illness	Knowledge and attitudes	All respondents with a complete survey	0·016 (-0·012 to 0·043)	0·26	9164
Identified difficulty breathing as d.s. for children with respiratory illness	Knowledge and attitudes	All respondents with a complete survey	0·011 (-0·0047 to 0·027)	0·17	9164
Identified rapid breathing as d.s. for children with respiratory illness	Knowledge and attitudes	All respondents with a complete survey	0·0084 (-0·0023 to 0·019)	0·12	9164
**Reproductive health**					
Reported ever using birth control to delay or avoid pregnancy	Practice	All respondents with a complete survey	0·0068 (-0·040 to 0·054)	0·78	8433
Reported currently using birth control	Practice	All respondents with a complete survey who are not pregnant and have ever used birth control	0·051 (-0·039 to 0·14)	0·27	1926
Believes woman should be at least 18 years of age to have her first child	Knowledge and attitudes	All respondents with a complete survey	0·012 (0·0046 to 0·019)	0·0015	8551
**Gender/reproductive norms**					
Birth location chosen either jointly or by woman	Practice	All respondents with a complete survey and a child born since 2018-09-01 (after the end of the intervention)	0·046 (-0·086 to 0·18)	0·49	291

Results based on sub-sample that completed baseline survey, N=9285 (household targeted n=2919, household not targeted n=6366). Covariates included baseline age, gender, education, marital status, indigenous status, household wealth index, and the related outcome. d.s.=Danger sign, NA=Not applicable, ORT=Oral

For peer review only - http://bmjopen.bmj.com/site/about/guidelines.xhtml

rehydration therapy. ··Model did not converge. Results with NA could not be assessed because outcome was not included in baseline survey. Covariates were dropped sequentially if they predicted outcome perfectly. Standard errors are clustered at the village level. Robust 95% CIs in parentheses.

[a] 'Identified correct ways to provide newborn care' outcome was coded as correct if the respondent identified holding baby skin to skin, getting their health checked by a professional, immediately putting them to the breast, or making sure their diaper is clean and dry, and did not identify giving purgante, wrapping a fajero, giving a chupon, or bathing them in water right away.
[b] 'Identified proper cord care' outcome was coded as correct if respondent identified applying alcohol, applying iodine, keeping cord clean/dry, or applying peroxide, and did not identify applying oil, applying coffee, applying quina water, wrapping a fajero, applying powder, burning, applying ashes, applying a clamp, or applying thiomersal.
[c] 'Used appropriate treatment for diarrhea in past 4 weeks' outcome was coded as correct if respondent did any of the following: antibiotic, zinc, give extra food, give extra liquid, and did not do any of the following: anti-diarrhea medication, home remedy, laxative, deworming medication, chupon, massage, stop eating foods, stop taking liquids.
[d] 'Identified appropriate diarrhea treatment methods' outcome was coded as correct if respondent identified any of the following: antibiotic, zinc, ORS or Litrosol, give extra food, give extra liquid, get medical attention, and did not identify any of the following: anti-diarrhea medication, laxative, deworming medication, home remedy, chupon, massage, stop giving foods, stop giving liquids, or do nothing.

For peer review only

Table S7. Pooled total effect estimates of intervention on maternal, child, and neonatal outcomes, with adjustment for baseline demographic characteristics and baseline outcome, estimated on sub-sample of respondents who did not move between villages during study

	Outcome type	Respondent denominator	Pooled total effect estimate		N
			Effect size (95% CI)	p value	
**Prenatal care**					
Currently taking folic acid tablets (daily in past 7 days)	Practice	All women 15+ with a complete survey	0·0014 (-0·040 to 0·043)	0·95	5284
Made a birth plan in preparation for birth	Practice	All respondents with a complete survey and a child born since 2018-09-01 (after the end of the intervention)	-0·082 (-0·22 to 0·055)	0·24	278
Saved money in preparation for birth	Practice	All respondents with a complete survey and a child born since 2018-09-01 (after the end of the intervention)	-0·0050 (-0·14 to 0·13)	0·94	281
Sold animal in preparation for birth	Practice	All respondents with a complete survey and a child born since 2018-09-01 (after the end of the intervention)	NA	NA	NA
Sought prenatal care within 12 weeks	Practice	All respondents with a complete survey and a child born since 2018-09-01 (after the end of the intervention)	0·033 (-0·11 to 0·17)	0·64	264
Identifies that women should take folic acid before pregnancy	Knowledge and attitudes	All respondents with a complete survey	0·058 (0·026 to 0·090)	0·0004	8875
Identifies that women should seek prenatal care first 12 weeks of pregnancy	Knowledge and attitudes	All respondents with a complete survey	0·030 (0·0068 to 0·052)	0·011	8875
Identifies accompanying woman to prenatal care visits as method of support during pregnancy	Knowledge and attitudes	All respondents with a complete survey	NA	NA	NA
Identifies ensuring that woman eats well as method of support during pregnancy	Knowledge and attitudes	All respondents with a complete survey	NA	NA	NA
Identifies ensuring that woman rests 1 hour per day well as method of support during pregnancy	Knowledge and attitudes	All respondents with a complete survey	NA	NA	NA
Identifies avoiding violence as method of support during pregnancy	Knowledge and attitudes	All respondents with a complete survey	NA	NA	NA
Identifies encouraging women to take vitamins as method of support during pregnancy	Knowledge and attitudes	All respondents with a complete survey	NA	NA	NA
Identifies helping woman with house work/child care as method of support during pregnancy	Knowledge and attitudes	All respondents with a complete survey	NA	NA	NA
Identifies saving animals to sell as method of preparing for birth expenses	Knowledge and attitudes	All respondents with a complete survey	NA	NA	NA

For peer review only - http://bmjopen.bmj.com/site/about/guidelines.xhtml

Identifies knowing cost of trip to maternal clinic as method of preparing for birth expenses	Knowledge and attitudes	All respondents with a complete survey	NA	NA	NA
Identifies having a savings plan as method of preparing for birth expenses	Knowledge and attitudes	All respondents with a complete survey	NA	NA	NA
Correctly answered prenatal care knowledge riddle	Intervention knowledge	All respondents with a complete survey	NA	NA	NA
**Facility based birth**					
Gave birth in health facility	Practice	All respondents with a complete survey and a child born since 2018-09-01 (after the end of the intervention)	0·029 (-0·10 to 0·16)	0·66	281
Believes health facility is best place to give birth	Knowledge and attitudes	All respondents with a complete survey	0·017 (-0·0050 to 0·039)	0·13	8866
**Pregnancy danger signs**					
Woman sought medical care for pregnancy danger sign	Practice	All respondents with a complete survey and a child born since 2018-09-01 (after the end of the intervention) who experienced a prenatal care danger sign	-0·29 (-0·82 to 0·24)	0·29	51
Identified bleeding as pregnancy d.s.	Knowledge and attitudes	All respondents with a complete survey	0·054 (0·013 to 0·095)	0·010	8872
Identified seizure as pregnancy d.s.	Knowledge and attitudes	All respondents with a complete survey	0·0022 (-0·0078 to 0·012)	0·67	8863
Identified headache as pregnancy d.s.	Knowledge and attitudes	All respondents with a complete survey	0·042 (-0·0059 to 0·090)	0·086	8872
Identified ringing in ears as pregnancy d.s.	Knowledge and attitudes	All respondents with a complete survey	0·0025 (-0·0073 to 0·012)	0·62	8848
Identified dizziness as pregnancy d.s.	Knowledge and attitudes	All respondents with a complete survey	-0·0061 (-0·055 to 0·043)	0·81	8872
Identified difficulty urinating as pregnancy d.s.	Knowledge and attitudes	All respondents with a complete survey	-0·0023 (-0·010 to 0·0055)	0·57	8848
Identified reduced or absent fetal movement as pregnancy d.s.	Knowledge and attitudes	All respondents with a complete survey	0·014 (-0·0064 to 0·034)	0·18	8848
Identified water breaking as pregnancy d.s.	Knowledge and attitudes	All respondents with a complete survey	-0·0031 (-0·0098 to 0·0035)	0·36	8848
Identified fever as pregnancy d.s.	Knowledge and attitudes	All respondents with a complete survey	0·039 (0·0002 to 0·077)	0·049	8872
Identified swelling of face/hands/feet as pregnancy d.s.	Knowledge and attitudes	All respondents with a complete survey	0·032 (0·0017 to 0·063)	0·038	8872
**Postnatal care for mother**					
Mother had health checked by professional within 3 days of birth	Practice	All respondents with a complete survey and a child born since 2018-09-01 (after the end of the intervention)	-0·058 (-0·24 to 0·13)	0·54	275

For peer review only - http://bmjopen.bmj.com/site/about/guidelines.xhtml

Mother had health checked by professional within 7 days of birth	Practice	All respondents with a complete survey and a child born since 2018-09-01 (after the end of the intervention)	-0·060 (-0·27 to 0·15)	0·57	272
Mother sought medical care for postnatal danger sign	Practice	All respondents with a complete survey and a child born since 2018-09-01 (after the end of the intervention)	0·32 (-0·015 to 0·66)	0·061	29
Identifies that mother should receive postnatal medical check-up within 3 days of birth	Knowledge and attitudes	All respondents with a complete survey	0·071 (0·028 to 0·11)	0·0011	8872
Identifies that mother should receive postnatal medical check-up within 7 days of birth	Knowledge and attitudes	All respondents with a complete survey	0·039 (-0·012 to 0·090)	0·13	8872
Identified heavy vaginal bleeding as postnatal d.s.	Knowledge and attitudes	All respondents with a complete survey	0·070 (0·032 to 0·11)	0·0003	8871
Identified fever as postnatal d.s.	Knowledge and attitudes	All respondents with a complete survey	0·057 (0·0076 to 0·11)	0·024	8871
Identified vaginal discharge as postnatal d.s.	Knowledge and attitudes	All respondents with a complete survey	0·0035 (-0·011 to 0·018)	0·62	8856
Identified headache as postnatal d.s.	Knowledge and attitudes	All respondents with a complete survey	0·031 (-0·0079 to 0·069)	0·12	8871
Identified convulsions or fits as postnatal d.s.	Knowledge and attitudes	All respondents with a complete survey	0·0004 (-0·0054 to 0·0063)	0·88	8673
**Postnatal care for newborn**					
Newborn had health checked by professional within 3 days of birth	Practice	All respondents with a complete survey and a child born since 2018-09-01 (after the end of the intervention)	0·0038 (-0·15 to 0·16)	0·96	279
Newborn had health checked by professional within 7 days of birth	Practice	All respondents with a complete survey and a child born since 2018-09-01 (after the end of the intervention)	-0·013 (-0·20 to 0·17)	0·89	276
Newborn experienced health problem in first month, care was sought	Practice	All respondents with a complete survey and a child born since 2018-09-01 (after the end of the intervention)	-0·011 (-0·011 to -0·011)	<0·0001	21
Identified correct ways to provide newborn care[a]	Knowledge and attitudes	All respondents with a complete survey	0·022 (0·0089 to 0·035)	0·0010	8851
Identified fever as newborn d.s.	Knowledge and attitudes	All respondents with a complete survey	0·0037 (-0·044 to 0·051)	0·88	8873
Identified diarrhea as newborn d.s.	Knowledge and attitudes	All respondents with a complete survey	0·0081 (-0·034 to 0·050)	0·71	8873
Identified difficulty breathing as newborn d.s.	Knowledge and attitudes	All respondents with a complete survey	0·0061 (-0·030 to 0·042)	0·74	8849
Identified vomiting as newborn d.s.	Knowledge and attitudes	All respondents with a complete survey	0·0089 (-0·021 to 0·039)	0·56	8864
Identified poor appetite as newborn d.s.	Knowledge and attitudes	All respondents with a complete survey	-0·0045 (-0·015 to 0·0054)	0·37	8849
Identified redness/bleeding around cord as newborn d.s.	Knowledge and attitudes	All respondents with a complete survey	0·021 (-0·0012 to 0·043)	0·064	8873
Identified pus in cord as newborn d.s.	Knowledge and attitudes	All respondents with a complete survey	0·015 (-0·0020 to 0·031)	0·085	8864
Identified cold skin as newborn d.s.	Knowledge and attitudes	All respondents with a complete survey	0·0024 (-0·0069 to 0·012)	0·61	8849

For peer review only - http://bmjopen.bmj.com/site/about/guidelines.xhtml

Identified cough as newborn d.s.	Knowledge and attitudes	All respondents with a complete survey	-0·020 (-0·070 to 0·031)	0·45	8873
Identified cold as newborn d.s.	Knowledge and attitudes	All respondents with a complete survey	-0·014 (-0·066 to 0·038)	0·61	8873
Identified problems latching as newborn d.s.	Knowledge and attitudes	All respondents with a complete survey	0·0001 (-0·014 to 0·014)	0·99	8847
Identified pneumonia as newborn d.s.	Knowledge and attitudes	All respondents with a complete survey	NA	NA	NA
Did not use harmful substances to treat cord stump	Practice	All respondents with a complete survey and a child born since 2018-09-01 (after the end of the intervention)	0·11 (-0·068 to 0·29)	0·23	271
Did not wrap fajero around newborn in first 7 days after birth	Practice	All respondents with a complete survey and a child born since 2018-09-01 (after the end of the intervention)	0·29 (0·099 to 0·48)	0·0029	277
Identified proper cord care methods[b]	Knowledge and attitudes	All respondents with a complete survey	0·10 (0·053 to 0·15)	<0·0001	8874
Correctly answered proper cord care riddle	Intervention knowledge	All respondents with a complete survey	NA	NA	NA
Kept newborn wrapped first 7 days after birth	Practice	All respondents with a complete survey and a child born since 2018-09-01 (after the end of the intervention)	..	..	..
Held newborn skin-to-skin during first month after birth	Practice	All respondents with a complete survey and a child born since 2018-09-01 (after the end of the intervention)	0·051 (-0·21 to 0·31)	0·70	68
**Breastfeeding**					
Exclusively breastfed child first 6 months	Practice	All respondents with a complete survey and a child born since 2018-09-01 (after the end of the intervention)	-0·12 (-0·26 to 0·012)	0·073	280
Exclusively breastfed child first 6 months without giving chupón	Practice	All respondents with a complete survey and a child born since 2018-09-01 (after the end of the intervention)	-0·060 (-0·24 to 0·12)	0·51	281
Breastfed immediately after birth	Practice	All respondents with a complete survey and a child born since 2018-09-01 (after the end of the intervention) who reported a natural birth	-0·0011 (-0·12 to 0·11)	0·98	210
Identifies that newborn should be breastfed immediately after birth	Knowledge and attitudes	All respondents with a complete survey	0·016 (-0·019 to 0·050)	0·38	8875
Identifies that newborns should only be given breast milk during first 6 months	Knowledge and attitudes	All respondents with a complete survey	0·039 (0·0080 to 0·069)	0·014	8874
Believes newborns should not be given chupón during first 6 months	Knowledge and attitudes	All respondents with a complete survey	0·16 (0·10 to 0·22)	<0·0001	8875
**Paternal involvement**					
Father accompanied mother to clinic for prenatal care visit at least once	Practice	All respondents with a complete survey and a child born since 2018-09-01 (after the end of the intervention) who received prenatal care	0·074 (-0·11 to 0·26)	0·44	259
Father accompanied mother to seek medical care for pregnancy danger sign	Practice	All respondents with a complete survey and a child born since 2018-09-01 (after the end of the intervention) who experienced a prenatal care danger sign and sought care	..	..	..

For peer review only - http://bmjopen.bmj.com/site/about/guidelines.xhtml

Father waited at birthplace during labor	Practice	All respondents with a complete survey and a child born since 2018-09-01 (after the end of the intervention)	-0·031 (-0·19 to 0·13)	0·70	281
Father accompanied mother to seek medical care for postnatal danger sign	Practice	All respondents with a complete survey and a child born since 2018-09-01 (after the end of the intervention) and sought care for postpartum problem	..	..	..
Father sought medical care for newborn for postnatal danger sign	Practice	All respondents with a complete survey and a child born since 2018-09-01 (after the end of the intervention) and sought care for a postnatal problem	..	..	..
Father held child	Practice	All respondents with a complete survey and a child born since 2018-09-01 (after the end of the intervention)	0·057 (-0·046 to 0·16)	0·28	258
Father played with child	Practice	All respondents with a complete survey and a child born since 2018-09-01 (after the end of the intervention)	0·073 (-0·043 to 0·19)	0·22	262
Father took child to clinic when sick	Practice	All respondents with a complete survey and a child born since 2018-09-01 (after the end of the intervention)	0·089 (-0·086 to 0·26)	0·32	262
Believes father should accompany mother to prenatal care visits	Knowledge and attitudes	All respondents with a complete survey	0·039 (0·017 to 0·061)	0·0006	8807
Believes father should wait at birth location while mother gives birth	Knowledge and attitudes	All respondents with a complete survey	0·013 (-0·0057 to 0·032)	0·17	8810
Believes father should care for children when sick	Knowledge and attitudes	All respondents with a complete survey	0·0004 (-0·0058 to 0·0065)	0·90	8851
**Diarrhea management**					
Did not report diarrhea in past 4 weeks	Practice	All respondents with a complete survey	-0·0095 (-0·026 to 0·0068)	0·26	8861
Used ORT to treat diarrhea in past 4 weeks	Practice	All respondents with a complete survey who had diarrhea in past 4 weeks	..	..	..
Used appropriate treatment for diarrhea in past 4 weeks[c]	Practice	All respondents with a complete survey who had diarrhea in past 4 weeks	..	..	..
Child did not experience diarrhea past 4 weeks	Practice	All respondents with a complete survey and a new child <= 5 years old reported at wave 3 who had diarrhea in past 4 weeks	-0·0094 (-0·054 to 0·035)	0·68	2257
Child experienced diarrhea past 4 weeks, was given appropriate amount of liquids	Practice	All respondents with a complete survey and a new child <= 5 years old reported at wave 3 who had diarrhea in past 4 weeks	1·9 (1·4 to 2·4)	<0·0001	41
Child experienced diarrhea past 4 weeks, was given appropriate amount of food	Practice	All respondents with a complete survey and a new child <= 5 years old reported at wave 3 who had diarrhea in past 4 weeks	1·2 (0·74 to 1·6)	<0·0001	36
Child experienced diarrhea past 4 weeks, was given ORT	Practice	All respondents with a complete survey and a new child <= 5 years old reported at wave 3 who had diarrhea in past 4 weeks	1·0 (0·62 to 1·4)	<0·0001	41
Child experienced diarrhea past 4 weeks, was given appropriate treatment	Practice	All respondents with a complete survey and a new child <= 5 years old reported at wave 3 who had diarrhea in past 4 weeks	0·58 (0·16 to 1·0)	0·0071	46
Child experienced diarrhea past 4 weeks, was given zinc 10-14 days	Practice	All respondents with a complete survey and a new child <= 5 years old reported at wave 3 who had diarrhea in past 4 weeks	..	..	..
Identified appropriate diarrhea treatment methods[d]	Knowledge and attitudes	All respondents with a complete survey	0·10 (0·051 to 0·16)	0·0001	8873
Identified zinc 10-14 days as diarrhea treatment	Knowledge and attitudes	All respondents with a complete survey	0·033 (0·0091 to 0·057)	0·0068	8835

For peer review only - http://bmjopen.bmj.com/site/about/guidelines.xhtml

For peer review only

Identified zinc supplement as way to prevent diarrhea	Knowledge and attitudes	All respondents with a complete survey	0·071 (0·0039 to 0·14)	0·038	8875
Identified breastfeeding as way to prevent diarrhea	Knowledge and attitudes	All respondents with a complete survey	0·038 (-0·024 to 0·099)	0·23	8875
Correctly answered diarrhea treatment with zinc riddle	Intervention knowledge	All respondents with a complete survey	NA	NA	NA
**Respiratory illness**					
Did not report respiratory illness (coughing) for 2 weeks	Practice	All respondents with a complete survey	-0·015 (-0·048 to 0·019)	0·39	8868
Child did not have cough past 4 weeks	Practice	All respondents with a complete survey	-0·044 (-0·11 to 0·022)	0·19	2257
Child had cough past 4 weeks, care was sought	Practice	All respondents with a complete survey and a new child <= 5 years old reported at wave 3 who had an illness with a cough in past 4 weeks	0·0005 (-0·24 to 0·24)	1·00	187
Identified vaccination as way to prevent respiratory illness	Knowledge and attitudes	All respondents with a complete survey	-0·0026 (-0·040 to 0·035)	0·89	8875
Identified breastfeeding as way to prevent respiratory illness	Knowledge and attitudes	All respondents with a complete survey	0·029 (-0·023 to 0·081)	0·27	8875
Identified fever as d.s. for children with respiratory illness	Knowledge and attitudes	All respondents with a complete survey	0·017 (-0·011 to 0·044)	0·23	8866
Identified difficulty breathing as d.s. for children with respiratory illness	Knowledge and attitudes	All respondents with a complete survey	0·011 (-0·0048 to 0·028)	0·17	8866
Identified rapid breathing as d.s. for children with respiratory illness	Knowledge and attitudes	All respondents with a complete survey	0·0086 (-0·0022 to 0·019)	0·12	8866
**Reproductive health**					
Reported ever using birth control to delay or avoid pregnancy	Practice	All respondents with a complete survey	0·0035 (-0·045 to 0·052)	0·89	8172
Reported currently using birth control	Practice	All respondents with a complete survey who are not pregnant and have ever used birth control	0·058 (-0·035 to 0·15)	0·22	1868
Believes woman should be at least 18 years of age to have her first child	Knowledge and attitudes	All respondents with a complete survey	0·012 (0·0043 to 0·019)	0·0018	8276
**Gender/reproductive norms**					
Birth location chosen either jointly or by woman	Practice	All respondents with a complete survey and a child born since 2018-09-01 (after the end of the intervention)	0·063 (-0·067 to 0·19)	0·34	277

Results based on sub-sample of respondents who didn't move villages during the study who also completed baseline survey, N=8981 (household targeted n=2831, household not targeted n=6150). Covariates included baseline age, gender, education, marital status, indigenous status, household wealth index, and the related outcome. d.s.=Danger sign, NA=Not applicable, ORT=Oral rehydration therapy. ··Model did not converge. Results with NA could not be assessed

For peer review only - http://bmjopen.bmj.com/site/about/guidelines.xhtml

because outcome was not included in baseline survey. Covariates were dropped sequentially if they predicted outcome perfectly. Standard errors are clustered at the village level. Robust 95% CIs in parentheses.

[a] 'Identified correct ways to provide newborn care' outcome was coded as correct if the respondent identified holding baby skin to skin, getting their health checked by a professional, immediately putting them to the breast, or making sure their diaper is clean and dry, and did not identify giving purgante, wrapping a fajero, giving a chupon, or bathing them in water right away.
[b] 'Identified proper cord care' outcome was coded as correct if respondent identified applying alcohol, applying iodine, keeping cord clean/dry, or applying peroxide, and did not identify applying oil, applying coffee, applying quina water, wrapping a fajero, applying powder, burning, applying ashes, applying a clamp, or applying thiomersal.
[c] 'Used appropriate treatment for diarrhea in past 4 weeks' outcome was coded as correct if respondent did any of the following: antibiotic, zinc, give extra food, give extra liquid, and did not do any of the following: anti-diarrhea medication, home remedy, laxative, deworming medication, chupon, massage, stop eating foods, stop taking liquids.
[d] 'Identified appropriate diarrhea treatment methods' outcome was coded as correct if respondent identified any of the following: antibiotic, zinc, ORS or Litrosol, give extra food, give extra liquid, get medical attention, and did not identify any of the following: anti-diarrhea medication, laxative, deworming medication, home remedy, chupon, massage, stop giving foods, stop giving liquids, or do nothing.

For peer review only - http://bmjopen.bmj.com/site/about/guidelines.xhtml

Table S8. Pooled total effect estimates of intervention on maternal, child, and neonatal outcomes, intervention module receipt defined as treatment, with adjustment for baseline demographic characteristics

	Outcome type	Respondent denominator	Intervention module(s)	Pooled total effect estimate		N
				Effect size (95% CI)	p value	
**Prenatal care**						
Currently taking folic acid tablets (daily in past 7 days)	Practice	All women 15+ with a complete survey	m11	0·017 (-0·035 to 0·070)	0·52	5922
Made a birth plan in preparation for birth	Practice	All respondents with a complete survey and a child born since 2018-09-01 (after the end of the intervention)	m2	0·021 (-0·12 to 0·16)	0·77	536
Saved money in preparation for birth	Practice	All respondents with a complete survey and a child born since 2018-09-01 (after the end of the intervention)	m1	-0·061 (-0·19 to 0·073)	0·37	540
Sold animal in preparation for birth	Practice	All respondents with a complete survey and a child born since 2018-09-01 (after the end of the intervention)	m1	-0·0070 (-0·090 to 0·076)	0·87	535
Sought prenatal care within 12 weeks	Practice	All respondents with a complete survey and a child born since 2018-09-01 (after the end of the intervention)	m1	-0·037 (-0·20 to 0·13)	0·67	518
Identifies that women should take folic acid before pregnancy	Knowledge and attitudes	All respondents with a complete survey	m11	0·097 (0·063 to 0·13)	<0·0001	9197
Identifies that women should seek prenatal care first 12 weeks of pregnancy	Knowledge and attitudes	All respondents with a complete survey	m1	0·060 (0·040 to 0·081)	<0·0001	9196
Identifies accompanying woman to prenatal care visits as method of support during pregnancy	Knowledge and attitudes	All respondents with a complete survey	m1	0·0012 (-0·057 to 0·060)	0·97	9192
Identifies ensuring that woman eats well as method of support during pregnancy	Knowledge and attitudes	All respondents with a complete survey	m1	-0·022 (-0·084 to 0·039)	0·48	9192
Identifies ensuring that woman rests 1 hour per day well as method of support during pregnancy	Knowledge and attitudes	All respondents with a complete survey	m1	0·0053 (-0·038 to 0·048)	0·81	9183
Identifies avoiding violence as method of support during pregnancy	Knowledge and attitudes	All respondents with a complete survey	m1	-0·032 (-0·054 to -0·0097)	0·0049	9192
Identifies encouraging women to take vitamins as method of support during pregnancy	Knowledge and attitudes	All respondents with a complete survey	m1	-0·038 (-0·074 to -0·0016)	0·041	9192

For peer review only - http://bmjopen.bmj.com/site/about/guidelines.xhtml

Identifies helping woman with house work/child care as method of support during pregnancy	Knowledge and attitudes	All respondents with a complete survey	m1	0·099 (0·032 to 0·17)	0·0037	9192
Identifies saving animals to sell as method of preparing for birth expenses	Knowledge and attitudes	All respondents with a complete survey	m1	0·026 (-0·0042 to 0·057)	0·091	9187
Identifies knowing cost of trip to maternal clinic as method of preparing for birth expenses	Knowledge and attitudes	All respondents with a complete survey	m1	0·0057 (-0·036 to 0·047)	0·79	9196
Identifies having a savings plan as method of preparing for birth expenses	Knowledge and attitudes	All respondents with a complete survey	m1	0·059 (0·036 to 0·083)	<0·0001	9172
Correctly answered prenatal care knowledge riddle	Intervention knowledge	All respondents with a complete survey	m1	0·12 (0·079 to 0·17)	<0·0001	9196
**Facility based birth**						
Gave birth in health facility	Practice	All respondents with a complete survey and a child born since 2018-09-01 (after the end of the intervention)	m3	-0·039 (-0·18 to 0·10)	0·58	540
Believes health facility is best place to give birth	Knowledge and attitudes	All respondents with a complete survey	m3	0·034 (0·0069 to 0·061)	0·014	9188
**Pregnancy danger signs**						
Woman sought medical care for pregnancy danger sign	Practice	All respondents with a complete survey and a child born since 2018-09-01 (after the end of the intervention) who experienced a prenatal care danger sign	m1	0·025 (-0·22 to 0·27)	0·84	209
Identified bleeding as pregnancy d.s.	Knowledge and attitudes	All respondents with a complete survey	m1	0·070 (0·023 to 0·12)	0·0035	9195
Identified seizure as pregnancy d.s.	Knowledge and attitudes	All respondents with a complete survey	m1	-0·0032 (-0·013 to 0·0066)	0·53	9186
Identified headache as pregnancy d.s.	Knowledge and attitudes	All respondents with a complete survey	m1	0·059 (0·0056 to 0·11)	0·030	9195
Identified ringing in ears as pregnancy d.s.	Knowledge and attitudes	All respondents with a complete survey	m1	0·0044 (-0·0074 to 0·016)	0·46	9171
Identified dizziness as pregnancy d.s.	Knowledge and attitudes	All respondents with a complete survey	m1	-0·025 (-0·085 to 0·036)	0·43	9195
Identified difficulty urinating as pregnancy d.s.	Knowledge and attitudes	All respondents with a complete survey	m1	-0·0066 (-0·014 to 0·0008)	0·079	9171
Identified reduced or absent fetal movement as pregnancy d.s.	Knowledge and attitudes	All respondents with a complete survey	m1	0·032 (0·0006 to 0·062)	0·046	9171
Identified water breaking as pregnancy d.s.	Knowledge and attitudes	All respondents with a complete survey	m1	0·0017 (-0·0078 to 0·011)	0·72	9171
Identified fever as pregnancy d.s.	Knowledge and attitudes	All respondents with a complete survey	m1	0·066 (0·015 to 0·12)	0·012	9195

For peer review only - http://bmjopen.bmj.com/site/about/guidelines.xhtml

Identified swelling of face/hands/feet as pregnancy d.s.	Knowledge and attitudes	All respondents with a complete survey	m1	0·064 (0·022 to 0·11)	0·0028	9195
**Postnatal care for mother**						
Mother had health checked by professional within 3 days of birth	Practice	All respondents with a complete survey and a child born since 2018-09-01 (after the end of the intervention)	m5, m6	0·22 (0·034 to 0·40)	0·020	530
Mother had health checked by professional within 7 days of birth	Practice	All respondents with a complete survey and a child born since 2018-09-01 (after the end of the intervention)	m5, m6	0·15 (-0·0047 to 0·31)	0·057	530
Mother sought medical care for postnatal danger sign	Practice	All respondents with a complete survey and a child born since 2018-09-01 (after the end of the intervention)	m5, m6	0·20 (-0·027 to 0·42)	0·084	178
Identifies that mother should receive postnatal medical check-up within 3 days of birth	Knowledge and attitudes	All respondents with a complete survey	m5, m6	0·13 (0·075 to 0·18)	<0·0001	9196
Identifies that mother should receive postnatal medical check-up within 7 days of birth	Knowledge and attitudes	All respondents with a complete survey	m5, m6	0·100 (0·048 to 0·15)	0·00010	9196
Identified heavy vaginal bleeding as postnatal d.s.	Knowledge and attitudes	All respondents with a complete survey	m5, m6	0·12 (0·068 to 0·16)	<0·0001	9195
Identified fever as postnatal d.s.	Knowledge and attitudes	All respondents with a complete survey	m5, m6	0·095 (0·036 to 0·15)	0·0016	9195
Identified vaginal discharge as postnatal d.s.	Knowledge and attitudes	All respondents with a complete survey	m5, m6	0·0062 (-0·014 to 0·026)	0·54	9180
Identified headache as postnatal d.s.	Knowledge and attitudes	All respondents with a complete survey	m5, m6	0·018 (-0·026 to 0·061)	0·42	9195
Identified convulsions or fits as postnatal d.s.	Knowledge and attitudes	All respondents with a complete survey	m5, m6	0·0050 (-0·0054 to 0·016)	0·35	8983
**Postnatal care for newborn**						
Newborn had health checked by professional within 3 days of birth	Practice	All respondents with a complete survey and a child born since 2018-09-01 (after the end of the intervention)	m5, m6	0·30 (0·13 to 0·47)	0·0006	532
Newborn had health checked by professional within 7 days of birth	Practice	All respondents with a complete survey and a child born since 2018-09-01 (after the end of the intervention)	m5, m6	0·22 (0·064 to 0·37)	0·0055	532
Newborn experienced health problem in first month, care was sought	Practice	All respondents with a complete survey and a child born since 2018-09-01 (after the end of the intervention)	m5, m6	0·25 (0·049 to 0·45)	0·015	154
Identified correct ways to provide newborn care[a]	Knowledge and attitudes	All respondents with a complete survey	m5, m6, m7	0·029 (0·010 to 0·047)	0·0021	9172
Identified fever as newborn d.s.	Knowledge and attitudes	All respondents with a complete survey	m7	-0·0012 (-0·054 to 0·052)	0·96	9195

For peer review only - http://bmjopen.bmj.com/site/about/guidelines.xhtml

Identified diarrhea as newborn d.s.	Knowledge and attitudes	All respondents with a complete survey	m7	0·0025 (-0·049 to 0·054)	0·92	9195
Identified difficulty breathing as newborn d.s.	Knowledge and attitudes	All respondents with a complete survey	m7	0·028 (-0·013 to 0·070)	0·18	9171
Identified vomiting as newborn d.s.	Knowledge and attitudes	All respondents with a complete survey	m7	0·023 (-0·018 to 0·064)	0·27	9186
Identified poor appetite as newborn d.s.	Knowledge and attitudes	All respondents with a complete survey	m7	0·0060 (-0·013 to 0·025)	0·54	9171
Identified redness/bleeding around cord as newborn d.s.	Knowledge and attitudes	All respondents with a complete survey	m7	0·032 (0·0034 to 0·060)	0·028	9195
Identified pus in cord as newborn d.s.	Knowledge and attitudes	All respondents with a complete survey	m7	0·032 (0·0051 to 0·060)	0·020	9186
Identified cold skin as newborn d.s.	Knowledge and attitudes	All respondents with a complete survey	m7	0·012 (-0·0079 to 0·033)	0·23	9171
Identified cough as newborn d.s.	Knowledge and attitudes	All respondents with a complete survey	m7	-0·033 (-0·087 to 0·021)	0·23	9195
Identified cold as newborn d.s.	Knowledge and attitudes	All respondents with a complete survey	m7	-0·029 (-0·090 to 0·031)	0·34	9195
Identified problems latching as newborn d.s.	Knowledge and attitudes	All respondents with a complete survey	m7	0·0028 (-0·016 to 0·021)	0·77	9171
Identified pneumonia as newborn d.s.	Knowledge and attitudes	All respondents with a complete survey	m7	0·028 (-0·016 to 0·072)	0·22	9171
Did not use harmful substances to treat cord stump	Practice	All respondents with a complete survey and a child born since 2018-09-01 (after the end of the intervention)	m5, m6, m7	0·11 (-0·045 to 0·26)	0·17	527
Did not wrap fajero around newborn in first 7 days after birth	Practice	All respondents with a complete survey and a child born since 2018-09-01 (after the end of the intervention)	m5, m6, m7	0·30 (0·13 to 0·47)	0·0005	535
Identified proper cord care methods[b]	Knowledge and attitudes	All respondents with a complete survey	m5, m6, m7	0·16 (0·10 to 0·22)	<0·0001	9195
Correctly answered proper cord care riddle	Intervention knowledge	All respondents with a complete survey	m5, m6, m7	0·30 (0·25 to 0·34)	<0·0001	9197
Kept newborn wrapped first 7 days after birth	Practice	All respondents with a complete survey and a child born since 2018-09-01 (after the end of the intervention)	m5, m6, m7	-0·0064 (-0·059 to 0·047)	0·81	530
Held newborn skin-to-skin during first month after birth	Practice	All respondents with a complete survey and a child born since 2018-09-01 (after the end of the intervention)	m5, m6, m7	0·22 (0·081 to 0·36)	0·0020	537
**Breastfeeding**						
Exclusively breastfed child first 6 months	Practice	All respondents with a complete survey and a child born since 2018-09-01 (after the end of the intervention)	m5, m6, m7, m8	0·11 (-0·031 to 0·25)	0·13	539
Exclusively breastfed child first 6 months without giving chupón	Practice	All respondents with a complete survey and a child born since 2018-09-01 (after the end of the intervention)	m5, m6, m7, m8	0·20 (0·058 to 0·35)	0·0060	539

For peer review only - http://bmjopen.bmj.com/site/about/guidelines.xhtml

Breastfed immediately after birth	Practice	All respondents with a complete survey and a child born since 2018-09-01 (after the end of the intervention) who reported a natural birth	m5, m6, m7, m8	0·071 (-0·033 to 0·18)	0·18	428
Identifies that newborn should be breastfed immediately after birth	Knowledge and attitudes	All respondents with a complete survey	m5, m6, m7, m8	0·037 (0·0018 to 0·072)	0·040	9196
Identifies that newborns should only be given breast milk during first 6 months	Knowledge and attitudes	All respondents with a complete survey	m5, m6, m7, m8	0·061 (0·030 to 0·092)	0·0001	9196
Believes newborns should not be given chupón during first 6 months	Knowledge and attitudes	All respondents with a complete survey	m5, m6, m7, m8	0·22 (0·16 to 0·28)	<0·0001	9196
**Paternal involvement**						
Father accompanied mother to clinic for prenatal care visit at least once	Practice	All respondents with a complete survey and a child born since 2018-09-01 (after the end of the intervention) who received prenatal care	m5, m6, m7	-0·071 (-0·30 to 0·16)	0·54	519
Father accompanied mother to seek medical care for pregnancy danger sign	Practice	All respondents with a complete survey and a child born since 2018-09-01 (after the end of the intervention) who experienced a prenatal care danger sign and sought care	m5, m6, m7	0·073 (-0·24 to 0·39)	0·65	134
Father waited at birthplace during labor	Practice	All respondents with a complete survey and a child born since 2018-09-01 (after the end of the intervention)	m5, m6, m7	-0·019 (-0·16 to 0·12)	0·79	540
Father accompanied mother to seek medical care for postnatal danger sign	Practice	All respondents with a complete survey and a child born since 2018-09-01 (after the end of the intervention) and sought care for postpartum problem	m5, m6, m7	0·070 (-0·26 to 0·40)	0·68	93
Father sought medical care for newborn for postnatal danger sign	Practice	All respondents with a complete survey and a child born since 2018-09-01 (after the end of the intervention) and sought care for a postnatal problem	m5, m6, m7	0·044 (-0·24 to 0·33)	0·76	98
Father held child	Practice	All respondents with a complete survey and a child born since 2018-09-01 (after the end of the intervention)	m5, m6, m7	-0·010 (-0·11 to 0·091)	0·85	535
Father played with child	Practice	All respondents with a complete survey and a child born since 2018-09-01 (after the end of the intervention)	m5, m6, m7	-0·045 (-0·20 to 0·11)	0·56	535
Father took child to clinic when sick	Practice	All respondents with a complete survey and a child born since 2018-09-01 (after the end of the intervention)	m5, m6, m7	-0·086 (-0·24 to 0·072)	0·29	535
Believes father should accompany mother to prenatal care visits	Knowledge and attitudes	All respondents with a complete survey	m5, m6, m7	0·044 (0·021 to 0·067)	0·0002	9158
Believes father should wait at birth location while mother gives birth	Knowledge and attitudes	All respondents with a complete survey	m5, m6, m7	0·018 (-0·0006 to 0·037)	0·058	9156

For peer review only - http://bmjopen.bmj.com/site/about/guidelines.xhtml

Believes father should care for children when sick	Knowledge and attitudes	All respondents with a complete survey	m5, m6, m7	0·0005 (-0·0077 to 0·0086)	0·91	9177
**Diarrhea management**						
Did not report diarrhea in past 4 weeks	Practice	All respondents with a complete survey	m9	-0·0069 (-0·025 to 0·011)	0·46	9182
Used ORT to treat diarrhea in past 4 weeks	Practice	All respondents with a complete survey who had diarrhea in past 4 weeks	m9	-0·098 (-0·28 to 0·087)	0·30	241
Used appropriate treatment for diarrhea in past 4 weeks[c]	Practice	All respondents with a complete survey who had diarrhea in past 4 weeks	m9	-0·021 (-0·16 to 0·12)	0·78	243
Child did not experience diarrhea past 4 weeks	Practice	All respondents with a complete survey and a new child <= 5 years old reported at wave 3 who had diarrhea in past 4 weeks	m9	-0·023 (-0·070 to 0·024)	0·34	3033
Child experienced diarrhea past 4 weeks, was given appropriate amount of liquids	Practice	All respondents with a complete survey and a new child <= 5 years old reported at wave 3 who had diarrhea in past 4 weeks	m9	-0·15 (-0·35 to 0·050)	0·14	310
Child experienced diarrhea past 4 weeks, was given appropriate amount of food	Practice	All respondents with a complete survey and a new child <= 5 years old reported at wave 3 who had diarrhea in past 4 weeks	m9	-0·12 (-0·26 to 0·015)	0·081	313
Child experienced diarrhea past 4 weeks, was given ORT	Practice	All respondents with a complete survey and a new child <= 5 years old reported at wave 3 who had diarrhea in past 4 weeks	m9	0·15 (-0·041 to 0·35)	0·12	310
Child experienced diarrhea past 4 weeks, was given appropriate treatment	Practice	All respondents with a complete survey and a new child <= 5 years old reported at wave 3 who had diarrhea in past 4 weeks	m9	0·17 (-0·020 to 0·36)	0·080	312
Child experienced diarrhea past 4 weeks, was given zinc 10-14 days	Practice	All respondents with a complete survey and a new child <= 5 years old reported at wave 3 who had diarrhea in past 4 weeks	m9	..	..	..
Identified appropriate diarrhea treatment methods[d]	Knowledge and attitudes	All respondents with a complete survey	m9	0·13 (0·069 to 0·19)	<0·0001	9195
Identified zinc 10-14 days as diarrhea treatment	Knowledge and attitudes	All respondents with a complete survey	m9	0·054 (0·021 to 0·087)	0·0013	9181
Identified zinc supplement as way to prevent diarrhea	Knowledge and attitudes	All respondents with a complete survey	m9	0·071 (0·0038 to 0·14)	0·038	9196
Identified breastfeeding as way to prevent diarrhea	Knowledge and attitudes	All respondents with a complete survey	m9	0·038 (-0·023 to 0·099)	0·23	9196
Correctly answered diarrhea treatment with zinc riddle	Intervention knowledge	All respondents with a complete survey	m9	0·35 (0·30 to 0·40)	<0·0001	9197
**Respiratory illness**						
Did not report respiratory illness (coughing) for 2 weeks	Practice	All respondents with a complete survey	m9	-0·012 (-0·051 to 0·028)	0·56	9188
Child did not have cough past 4 weeks	Practice	All respondents with a complete survey	m9	-0·058 (-0·12 to 0·0070)	0·081	3034

For peer review only - http://bmjopen.bmj.com/site/about/guidelines.xhtml

Child had cough past 4 weeks, care was sought	Practice	All respondents with a complete survey and a new child <= 5 years old reported at wave 3 who had an illness with a cough in past 4 weeks	m9	0·064 (-0·090 to 0·22)	0·41	647
Identified vaccination as way to prevent respiratory illness	Knowledge and attitudes	All respondents with a complete survey	m9	-0·0053 (-0·046 to 0·036)	0·80	9196
Identified breastfeeding as way to prevent respiratory illness	Knowledge and attitudes	All respondents with a complete survey	m9	0·013 (-0·045 to 0·070)	0·66	9196
Identified fever as d.s. for children with respiratory illness	Knowledge and attitudes	All respondents with a complete survey	m9	0·021 (-0·0036 to 0·046)	0·093	9187
Identified difficulty breathing as d.s. for children with respiratory illness	Knowledge and attitudes	All respondents with a complete survey	m9	0·011 (-0·0046 to 0·026)	0·17	9187
Identified rapid breathing as d.s. for children with respiratory illness	Knowledge and attitudes	All respondents with a complete survey	m9	0·0096 (-0·0010 to 0·020)	0·075	9187
**Reproductive health**						
Reported ever using birth control to delay or avoid pregnancy	Practice	All respondents with a complete survey	m4, m10, m12	-0·017 (-0·069 to 0·036)	0·53	9191
Reported currently using birth control	Practice	All respondents with a complete survey who are not pregnant and have ever used birth control	m4, m10, m12	0·033 (-0·035 to 0·10)	0·34	3420
Believes woman should be at least 18 years of age to have her first child	Knowledge and attitudes	All respondents with a complete survey	m4, m10, m12	0·017 (0·0098 to 0·025)	<0·0001	8713
**Gender/reproductive norms**						
Birth location chosen either jointly or by woman	Practice	All respondents with a complete survey and a child born since 2018-09-01 (after the end of the intervention)	m14	0·055 (-0·036 to 0·15)	0·24	540

Results based on sub-sample that completed baseline survey, N=9285 (household targeted n=2919, household not targeted n=6366). Baseline covariates included age, gender, education, marital status, indigenous status, and household wealth index. d.s.=Danger sign, ORT=Oral rehydration therapy. ··Model did not converge. Covariates were dropped sequentially if they predicted outcome perfectly. Standard errors are clustered at the village level. Robust 95% CIs in parentheses.

[a] ‘Identified correct ways to provide newborn care’ outcome was coded as correct if the respondent identified holding baby skin to skin, getting their health checked by a professional, immediately putting them to the breast, or making sure their diaper is clean and dry, and did not identify giving purgante, wrapping a fajero, giving a chupon, or bathing them in water right away.
[b] ‘Identified proper cord care’ outcome was coded as correct if respondent identified applying alcohol, applying iodine, keeping cord clean/dry, or applying peroxide, and did not identify applying oil, applying coffee, applying quina water, wrapping a fajero, applying powder, burning, applying ashes, applying a clamp, or applying thiomersal.

For peer review only - http://bmjopen.bmj.com/site/about/guidelines.xhtml

[c] 'Used appropriate treatment for diarrhea in past 4 weeks' outcome was coded as correct if respondent did any of the following: antibiotic, zinc, give extra food, give extra liquid, and did not do any of the following: anti-diarrhea medication, home remedy, laxative, deworming medication, chupon, massage, stop eating foods, stop taking liquids.

[d] 'Identified appropriate diarrhea treatment methods' outcome was coded as correct if respondent identified any of the following: antibiotic, zinc, ORS or Litrosol, give extra food, give extra liquid, get medical attention, and did not identify any of the following: anti-diarrhea medication, laxative, deworming medication, home remedy, chupon, massage, stop giving foods, stop giving liquids, or do nothing.

For peer review only

References

1. Baird S, Bohren JA, McIntosh C, Özler B. Optimal Design of Experiments in the Presence of Interference. *https://doiorg/101162/rest_a_00716* 2018.
2. Hudgens M, Halloran M. Toward Causal Inference With Interference. *Journal of the American Statistical Association* 2008; **103**(482).

For peer review only

For peer review only - http://bmjopen.bmj.com/site/about/guidelines.xhtml

CONSORT 2010 checklist of information to include when reporting a randomised trial*

Section/Topic	Item No	Checklist item	Reported on page No
**Title and abstract**			
	1a	Identification as a randomised trial in the title	1
	1b	Structured summary of trial design, methods, results, and conclusions (for specific guidance see CONSORT for abstracts)	2
**Introduction**			
Background and objectives	2a	Scientific background and explanation of rationale	4-6
	2b	Specific objectives or hypotheses	6
**Methods**			
Trial design	3a	Description of trial design (such as parallel, factorial) including allocation ratio	6-11
	3b	Important changes to methods after trial commencement (such as eligibility criteria), with reasons	11
Participants	4a	Eligibility criteria for participants	6-7
	4b	Settings and locations where the data were collected	6-7
Interventions	5	The interventions for each group with sufficient details to allow replication, including how and when they were actually administered	8-11 & supplement
Outcomes	6a	Completely defined pre-specified primary and secondary outcome measures, including how and when they were assessed	8-11 & supplement
	6b	Any changes to trial outcomes after the trial commenced, with reasons	NA
Sample size	7a	How sample size was determined	4-5 in supplement
	7b	When applicable, explanation of any interim analyses and stopping guidelines	NA
Randomisation:			
Sequence generation	8a	Method used to generate the random allocation sequence	8-9 & supplement
	8b	Type of randomisation; details of any restriction (such as blocking and block size)	8-9 & supplement
Allocation concealment mechanism	9	Mechanism used to implement the random allocation sequence (such as sequentially numbered containers), describing any steps taken to conceal the sequence until interventions were assigned	8-9 & supplement

For peer review only

Implementation	10	Who generated the random allocation sequence, who enrolled participants, and who assigned participants to interventions	8-9 & supplement
Blinding	11a	If done, who was blinded after assignment to interventions (for example, participants, care providers, those assessing outcomes) and how	NA
	11b	If relevant, description of the similarity of interventions	NA
Statistical methods	12a	Statistical methods used to compare groups for primary and secondary outcomes	12-13 & supplement
	12b	Methods for additional analyses, such as subgroup analyses and adjusted analyses	12-13 & supplement
**Results**			
Participant flow (a diagram is strongly recommended)	13a	For each group, the numbers of participants who were randomly assigned, received intended treatment, and were analysed for the primary outcome	14-20 & supplement
	13b	For each group, losses and exclusions after randomisation, together with reasons	Figure 1, supplement
Recruitment	14a	Dates defining the periods of recruitment and follow-up	9
	14b	Why the trial ended or was stopped	NA
Baseline data	15	A table showing baseline demographic and clinical characteristics for each group	Table 1, 16
Numbers analysed	16	For each group, number of participants (denominator) included in each analysis and whether the analysis was by original assigned groups	Supplement
Outcomes and estimation	17a	For each primary and secondary outcome, results for each group, and the estimated effect size and its precision (such as 95% confidence interval)	Supplement
	17b	For binary outcomes, presentation of both absolute and relative effect sizes is recommended	Supplement
Ancillary analyses	18	Results of any other analyses performed, including subgroup analyses and adjusted analyses, distinguishing pre-specified from exploratory	Figure 2, & supplement
Harms	19	All important harms or unintended effects in each group (for specific guidance see CONSORT for harms)	NA
**Discussion**			
Limitations	20	Trial limitations, addressing sources of potential bias, imprecision, and, if relevant, multiplicity of analyses	22-27
Generalisability	21	Generalisability (external validity, applicability) of the trial findings	20-22, 27
Interpretation	22	Interpretation consistent with results, balancing benefits and harms, and considering other relevant evidence	20-22, 27
**Other information**			
Registration	23	Registration number and name of trial registry	2,13
Protocol	24	Where the full trial protocol can be accessed, if available	13,32

For peer review only - http://bmjopen.bmj.com/site/about/guidelines.xhtml

Funding	25	Sources of funding and other support (such as supply of drugs), role of funders	30

*We strongly recommend reading this statement in conjunction with the CONSORT 2010 Explanation and Elaboration for important clarifications on all the items. If relevant, we also recommend reading CONSORT extensions for cluster randomised trials, non-inferiority and equivalence trials, non-pharmacological treatments, herbal interventions, and pragmatic trials. Additional extensions are forthcoming: for those and for up to date references relevant to this checklist, see www.consort-statement.org.

For peer review only

For peer review only - http://bmjopen.bmj.com/site/about/guidelines.xhtml

BMJ Open

Maternal and child health intervention to promote behavior change: a population-level cluster-randomized controlled trial in Honduras

Journal:	*BMJ Open*
Manuscript ID	bmjopen-2022-060784.R1
Article Type:	Original research
Date Submitted by the Author:	28-Sep-2023

Complete List of Authors:	Oles, William; Yale University, Yale Institute for Network Science<br>Alexander, Marcus; Yale University, Yale Institute for Network Science<br>Negron , Rennie; Yale University, Yale Institute for Network Science<br>Nelson , Jennifer ; Inter-American Development Bank<br>Iriarte, Emma; Inter-American Development Bank<br>Airoldi, E; Temple University Fox School of Business and Management<br>Christakis, Nicholas; Yale University, Yale Institute for Network Science; Yale University, Departments of Sociology and Medicine<br>Forastiere, Laura; Yale University, Yale Institute for Network Science; Yale University, Department of Biostatistics

**Primary Subject Heading**:	Public health
Secondary Subject Heading:	Global health
Keywords:	Community child health < PAEDIATRICS, STATISTICS & RESEARCH METHODS, PUBLIC HEALTH, Maternal medicine < OBSTETRICS

SCHOLARONE™ Manuscripts

I, the Submitting Author has the right to grant and does grant on behalf of all authors of the Work (as defined in the below author licence), an exclusive licence and/or a non-exclusive licence for contributions from authors who are: i) UK Crown employees; ii) where BMJ has agreed a CC-BY licence shall apply, and/or iii) in accordance with the terms applicable for US Federal Government officers or employees acting as part of their official duties; on a worldwide, perpetual, irrevocable, royalty-free basis to BMJ Publishing Group Ltd ("BMJ") its licensees and where the relevant Journal is co-owned by BMJ to the co-owners of the Journal, to publish the Work in this journal and any other BMJ products and to exploit all rights, as set out in our licence.

The Submitting Author accepts and understands that any supply made under these terms is made by BMJ to the Submitting Author unless you are acting as an employee on behalf of your employer or a postgraduate student of an affiliated institution which is paying any applicable article publishing charge ("APC") for Open Access articles. Where the Submitting Author wishes to make the Work available on an Open Access basis (and intends to pay the relevant APC), the terms of reuse of such Open Access shall be governed by a Creative Commons licence – details of these licences and which Creative Commons licence will apply to this Work are set out in our licence referred to above.

Other than as permitted in any relevant BMJ Author's Self Archiving Policies, I confirm this Work has not been accepted for publication elsewhere, is not being considered for publication elsewhere and does not duplicate material already published. I confirm all authors consent to publication of this Work and authorise the granting of this licence.

For peer review only

Maternal and child health intervention to promote behavior change: a population-level cluster-randomized controlled trial in Honduras

William Oles,[1†] Marcus Alexander,[1†] Rennie Negron,[1] Jennifer Nelson,[2] Emma Iriarte,[2] E. M. Airoldi,[3] Nicholas A. Christakis,[1,4] Laura Forastiere[1,5*]

[1]Yale Institute for Network Science, Yale University, New Haven, CT, USA;
[2]Inter-American Development Bank, Washington, DC, USA;
[3]Fox School of Business, Temple University, Philadelphia, PA, USA;
[4]Departments of Sociology and Medicine, Yale University, New Haven, CT, USA; and
[5]Department of Biostatistics, Yale School of Public Health, Yale University, New Haven, CT, USA

[†]These two authors contributed equally to this work.

*Correspondence to: Professor Laura Forastiere, Department of Biostatistics, Yale School of Public Health, 350 George St, New Haven, CT, laura.forastiere@yale.edu

For peer review only - http://bmjopen.bmj.com/site/about/guidelines.xhtml

Abstract

Objectives:

To assess the efficacy of a sustained educational intervention to affect diverse outcomes across the pregnancy and infancy timeline.

Setting:

A multi-arm cluster-randomized controlled trial in 99 villages in Honduras' Copán region, involving 16,301 people in 5,633 households from October, 2015, to December, 2019.

Participants:

Residents aged 12 and older were eligible. A photographic census involved 93% of the population, with 13,881 and 10,263 individuals completing baseline and endline surveys, respectively.

Intervention:

22-month household-based counseling intervention aiming to improve practices, knowledge, and attitudes related to maternal, neonatal, and child health.

Primary and secondary outcome measures:

Primary outcomes were prenatal/postnatal care behaviors, facility births, exclusive breastfeeding, parental involvement, treatment of diarrhea and respiratory illness, reproductive health, and gender/reproductive norms. Secondary outcomes were knowledge and attitudes related to the primary outcomes.

Results:

Parents targeted for the intervention were 16·4% (95% CI 3·1%–29·8%, p=0·016; absolute change +13·9% targeted vs +5·8% untargeted) more likely to have their newborn's health checked in a health facility within three days of birth; 19·6% (95% CI 4·2%–35·1%, p=0·013; absolute change +37·9% targeted vs +16·1% untargeted) more likely to not wrap a fajero around the umbilical cord in the first week after birth; and 8·9% (95% CI 0·3%–17·5%, p=0·043; absolute change +5·1% targeted vs -4·1% untargeted) more likely report that the mother breastfed immediately after birth. Changes in knowledge and attitudes related to these primary outcomes were also observed. We found no significant effect on various other practices, including care-seeking for pregnancy or postnatal danger signs, facility based births, using skin-to-skin contact, or exclusive breastfeeding.

Conclusion:

A sustained counseling intervention delivered in the home setting by community health workers can meaningfully change practices, knowledge, and attitudes related to proper newborn care following birth, including professional care-seeking, umbilical cord care, and breastfeeding.

Trial registration:

ClinicalTrials.gov, NCT02694679.

Strengths and limitations of this study:

For peer review only

- High-quality data acquisition over four years using a modified version of Timed and Targeted Counseling methodology included dialogue counseling methods and interactive story-telling to assess current needs and practices of intervention recipients and negotiate on progressive improvement.

- A wider array of outcomes was collected than other trials of its kind, determining the impact of the intervention on various points of the pregnancy and infancy timeline.

- Two-stage randomization and different treatment dosages allowed estimation of pooled spillover effects upon targeted individuals or households, a feature uncommon to other large-scale interventions.

- Limitations include the potential influence of co-intervention in the study area; the reliance on self-reported outcomes; and that the intervention modules were delivered at different times and frequencies, depending on household composition.

For peer review only - http://bmjopen.bmj.com/site/about/guidelines.xhtml

Introduction

Despite significant global progress in many measures of maternal and under-5 child health over the past 30 years, maternal and child morbidity and mortality continue to pose a significant burden, particularly in resource-limited settings across Sub-Saharan Africa and Latin America. Globally, neonatal deaths account for nearly 50% of all deaths in children under 5 years old, and preterm birth complications, pneumonia, and intrapartum-related complications account for the majority of these deaths.[1,2] These causes suggest that poor maternal outcomes often underlie poor infant and child outcomes; consequently, large-scale interventions to improve resources, knowledge, and practices at every point in the pregnancy and infancy timeline have become a central part of the global health agenda. Interventions targeting maternal and child health outcomes are largely founded on evidence-based WHO guidelines, which emphasize birth preparedness, complication readiness, and care-seeking for the mother, as well as immediate stimulation, skin-to-skin contact, immediate and exclusive breastfeeding, umbilical cord care, thermal care and medical check-ups for the newborn, among others.[3,4]

Although many neonatal, child, and maternal deaths can be prevented through clinical services, emerging evidence has shown that reductions in poor health outcomes in these areas are possible by complementing them with low-cost interventions in the home and community setting.[5,6] This branch of intervention packages focuses on demand-side conditions, building on the idea that improvement in behaviors related to maternal and child health outcomes not only depends on the accessibility or provision of

For peer review only

For peer review only - http://bmjopen.bmj.com/site/about/guidelines.xhtml

health services, but also on the community-level demand for services and practices. Behaviors which affect maternal and child health outcomes are often socially reinforced or otherwise influenced by community norms and therefore difficult to change, making the on-ground implementation of interventions increasingly important. There is a large body of evidence that points to the efficacy of counseling interventions which (typically) employ interpersonal interactions with community health workers during home visits and may be implemented on their own or as one of many domains in a multidimensional intervention package.[6–10]

Despite the considerable progress that Honduras has made towards its population health goals, it still lags behind many other low-to-middle-income countries in Latin America. In Honduras in 2016, the under-5 mortality rate was 19.8 per 1000 live births while the neonatal mortality rate was for 11.4 per 1000 live births.[11] Despite important progress, these rates were greater than comparable countries in the same year, including Mexico, Nicaragua, and El Salvador, with only Guatemala exceeding that of Honduras.[11] The leading causes of neonatal mortality in Honduras were perinatal disorders, congenital malformations, pneumonia, diarrhea, and malnutrition.[12] With regard to women's and reproductive health, the most recent Honduras Demographic and Health Survey (DHS) reports that the total fertility rate of women aged 15-49 is 2.9, the percent of pregnancy or motherhood among those aged 15-19 is 24%, the median age of first marriage is 19.3 years, and the percent of women who are literate is 92.7.[13] Additionally, in 2020, the World Health Organization estimated that Honduras had a maternal mortality ratio of 72 deaths per 100,000 births, which corresponded to

For peer review only - http://bmjopen.bmj.com/site/about/guidelines.xhtml

4.2% of all deaths among women aged 15-49 being attributed to maternal causes.[14] Nationwide, 74% of live births occur in a medical facility, while 57% of live births occur in rural regions which likely face higher barriers to perinatal care resource access, given that Honduras already has the lowest physician density in Latin America.[15–17]

Few studies have combined an at-scale counseling intervention with randomized assessment methods in Latin America. Here, we completed a randomized trial in the western highlands of Honduras in which households received a two-year intervention package that consisted of household visits by community health workers (CHWs). CHWs were trained to use validated counseling methods to promote practices, knowledges, and attitudes related to desired maternal and child health outcomes. We assessed a wide range of outcomes across the pregnancy and infancy timeline that aligned with the intervention counseling topics, including prenatal care, facility-based births, postnatal care for mother and child, danger signs and care-seeking, breastfeeding, paternal involvement, diarrhea and respiratory illness prevention and management in children 5 and under, and reproductive health. Pertinently, the underlying framework of the intervention here also differed from similar efforts in other settings in that it targeted the broader community in addition to parents themselves.

The aim of our randomized assessment is thus to evaluate the effects of a large-scale, household-based counseling intervention on outcomes related to maternal, neonatal, and child health in rural villages in Honduras.

For peer review only - http://bmjopen.bmj.com/site/about/guidelines.xhtml

Methods

Study design and participants

We conducted a multi-arm cluster-randomized controlled trial in the Department of Copán in western Honduras comprising the municipalities of Copán Ruinas, Santa Rita, Cabañas, and San Jerónimo. Copán is a predominantly rural, mountainous, coffee-growing region and is characterized by high rates of neonatal and maternal morbidity and mortality, with many rural villages facing barriers to healthcare which often leave perinatal care insufficient or unsafe.[12,16] The trial was a partnership between the Yale Human Nature Lab (HNL), the Inter-American Development Bank (IDB), and the Ministry of Health of Honduras, with implementing partners World Vision Honduras and Dimagi. Of the 238 villages in the Copán region, 176 were selected for a parent trial evaluating a novel social network targeting technique; of these, 99 were included in the current assessment (villages in which participants were assigned to treatment based on a non-random, network-based algorithm were excluded). The parent trial was formally registered (NCT02694679) prior to implementation, and the full details of the parent trial design and methods are published in our 2017 study protocol.[17] Factors such as population size, geographic diversity, accessibility, and safety were considered when selecting villages, and power testing using conservative treatment-effect simulations following initial enrollment found recruitment to be adequate to detect the desired effects. The village selection process, study area profiling, and power testing are described further in our study protocol.[17]

For peer review only

All individuals aged 12 years or older and who lived in a study village were eligible to participate. We conducted a complete photographic census among individuals who agreed to enroll in the study which covered 93% of the eligible population. Any household with at least one eligible individual who agreed to enroll in the study and who completed the baseline survey was eligible for randomization.

Randomization and masking

The trial used a two-stage factorial design with a first-stage randomization at the village level and a second-stage randomization at the household level. In the first stage, we randomly assigned villages to a dosage, or proportion of households targeted for intervention per village (0, 0.05, 0.1, 0.2, 0.3, 0.5, 0.75, 1). In the second stage, households were randomly assigned to the intervention according to the village's dosage. We used a covariate-constrained randomization procedure than ensured balance at the village and household levels between arms. Our full randomization procedure is described in our published protocol.[17]

The current randomized assessment focuses on the 99 villages that were assigned to the random targeting strategy with a proportion of targeted households greater than 0 or the control arm with a proportion equal to 0. In other words, we had two kinds of control subjects: 1) untreated households in untreated villages, and 2) untreated households in treated villages. This is important because, when assessing whether subjects respond to receiving an educational intervention, one should adjust for whether, by chance, other members of their village also got the same intervention, thereby potentially reinforcing

For peer review only - http://bmjopen.bmj.com/site/about/guidelines.xhtml

the impact of the educational intervention. We note that this important detail of possible reinforcement of treatment response due to treatment of other community members is typically overlooked in most field trials of such interventions. Households in the 22 villages with a proportion of households targeted equal to 0 did not receive any intervention and were used as our pure control, or comparison, villages.

The intervention assessment reported here establishes if there were changes in practices, knowledge, and attitudes related to target outcomes among those actually receiving the intervention. Due to the nature of the intervention, masking of participants and community health workers, termed Community Change Agents (CCAs) in the current study, who delivered the intervention was not possible. However, both CCAs and surveyors were blinded to the methods used to select the intervention households assigned to them.

Procedures

We and our local partners introduced the project to village leaders, secured local approvals, and managed local implementation of the study and intervention. There was extensive local involvement in setting the agenda for this RCT. CCAs delivered up to 22 1–2 hour counseling sessions across 15 modules to targeted households at monthly intervals between November, 2016, and August, 2018. The intervention was designed by World Vision Honduras (who also hired, paid and trained CCAs) and was named Proyecto Redes: Con Amor y Cuidados Madres y Bebés Sanos (With Love and Care,

For peer review only

Healthy Moms and Babies).[18,19] Intervention delivery and outcomes assessments were conducted by two different, independent teams of people.

CCAs made home visits and spoke to families regarding several health topics tailored to the family's current circumstances and based on a modified version of the Timed and Targeted Counseling (ttC) methodology, which has been implemented in 20 countries worldwide by World Vision.[20] Additionally, the behavior-change communication strategy was designed with the P Process tool, which uses narrative and negotiation to reach agreements with families to try new practices and has been used for more than 30 years for planning health-communication programs.[21] The educational modules delivered during home visits were designed to address maternal, neonatal, and child health, and included topics such as preparation of birth plan, facility-based birth, mother and newborn care, and folic acid importance (see appendix for full list of educational modules). Sessions were also designed to include discussion of relevant regional practices that were identified during formative work in our pilot study as being potentially dangerous to newborns. Two such practices were the use of chupones during feeding (cloth materials dampened with saliva, honey, or plant waters) and the placement of a fajero (abdominal cloth wrap) around a newborn's umbilical cord site.[17,18] Additional information regarding the planned protocol for each session and details of ttC method integration is available in the appendix and is additionally published elsewhere, including the supplementary material of our study protocol.[17,19]

For peer review only - http://bmjopen.bmj.com/site/about/guidelines.xhtml

CCAs used tablets during intervention delivery, which served as a visual and audio aid for educational materials (i.e., families could see stories in video and listen to songs/riddles); as a prompt for CCAs to standardize intervention implementation; and as a medium to collect data.[22] Intervention delivery differed from other community-based approaches in that it was randomized to any household regardless of household composition (not just delivered to expectant mothers); it was comprised of long home visits and did not include additional outside messaging (i.e., pamphlets, radio messages); and it did not include the use of community action groups. However, it should be noted that both intervention and control villages may also have received other interventions simultaneously, including both traditional behavior change interventions through the Government of Honduras and supply-side interventions through IDB and the Salud Mesoamerica Initiative.[23]

We developed a structured survey instrument comprised of both validated scales and internally developed items based on extensive literature review, expert consultation, input from the Ministry of Health of Honduras, and qualitative research with local residents and village leaders. Our survey instrument was primarily designed to capture target outcomes related to maternal, neonatal, and child health, in addition to demographic information. Upon study enrollment, participants completed a photographic census and baseline survey from June, 2015, to June, 2016, using our publicly-available social network data collection software “Trellis”.[24] An additional photographic census was conducted from January, 2019, to December, 2019, at which point participants also completed the endline survey. For more details on our pilot work

For peer review only

and field operations see the study's published protocol.[17] No important changes to methods were made after trial commencement.

Outcomes

The primary outcomes can be grouped into the following practices: (1) prenatal care for the mother, including folic acid use, creating a birth plan, and receipt of prenatal care in first trimester; (2) giving birth in a health facility; (3) care-seeking for danger signs experienced by both mother and newborn; (4) postnatal care for mother, including preventative check-ups with health professionals; (5) postnatal care for newborn, including preventative check-ups with health professionals, proper thermal care, and proper cord care (including withholding use of fajero); (6) immediate and exclusive breastfeeding for infants under 6 months (including withholding use of chupones); (7) paternal involvement in pregnancy and child care; (8) proper treatment of diarrhea and respiratory illness in children 5 and under; (9) reproductive health (including contraceptive use); and (10) gender/reproductive norms (see appendix for full list of outcomes). Secondary outcomes were knowledge or attitudes related to the primary outcomes. All outcomes were measured via our endline survey instrument. The inclusion of certain responses to our survey questions was determined by eligibility encoded into the survey design, which was termed "denominator" and can be found in the appendix for each outcome (table S1, appendix). For example, only mothers and fathers who had a child since the end of the intervention (September 1, 2018) were eligible to have a response included for the endline survey question about whether their child was exclusively breastfed for the first 6 months.

For peer review only - http://bmjopen.bmj.com/site/about/guidelines.xhtml

Statistical analysis

Given that our intervention was designed to be delivered at the household-level and that its behavior change strategies are embedded in ideas of addressing community norms, we cannot rule out the presence of between-participant interference within villages. In particular, we assume that there could be interference within villages but not between villages (partial interference), and that a respondent's outcome may be affected by the proportion of treated household in the village in addition to their own treatment status (stratified interference).[25,26] This assumption is substantiated by an analysis in prior work in a different part of Honduras that showed enhanced adoption of a nutritional intervention consistent with social magnification.[27]

In this analysis, we use a two-stage randomization design (in which we randomized village clusters to a treatment arm and then further randomized households to receive the intervention) in order to estimate the total effect of the intervention on the treated (the effect of receiving the treatment in an intervention village compared to a control village); the direct effects on the treated (which are effects attributable to receiving the intervention after adjusting for spillover effects upon the targeted individuals); and finally the spillover effects on the untreated who lived in the treated villages and may have been affected by others who received the intervention.[25] We focus on the first effect here.

For peer review only - http://bmjopen.bmj.com/site/about/guidelines.xhtml

Under an intent-to-treat framework, we used multivariate logistic regression fitted to individual-level data for analysis of binary outcomes. We pooled responses across all treatment arms to obtain an average effect across all village-level treatment dosages, adding sampling weights to adjust for differential probability of treatment assignment. Clustered heteroskedasticity-robust standard errors that allowed for intragroup correlation were estimated at the level of villages, which was the unit of randomization in the first stage. Further information about our analysis strategy can be found in the appendix. Significance was defined as p<0.05. All analyses were performed using R version 4.0.4 and Stata version 13. This trial is registered at ClinicalTrials.gov, NCT02694679.

Patient and Public Involvement

Community leaders of each of the villages were involved as partners in this study, and both the intervention team and the survey data collection team worked with local communities at each step of the study design process to ensure feasibility and incorporate feedback obtained through focus groups, interviews, pilot surveys, and other outreach.

Results

Trial participants 12 and older were recruited and completed a baseline survey between June, 2015, and June, 2016. A total of 16,301 individuals across 5,633 households were randomized, 13,881 of whom completed baseline surveys. And 10,263 individuals completed the endline survey between January, 2019, and December, 2019,

For peer review only - http://bmjopen.bmj.com/site/about/guidelines.xhtml

approximately 24 months after intervention delivery began. Of 6,038 individuals lost by the endline, 4% were lost to death, 50% to out-migration, 12% to refusal to continue in the study, 32% due to not being reached, and 2% to other reasons. Those lost to follow-up were more likely to be younger, male, have a primary school or greater education, be single, not identify as Maya Chorti, and have greater self-rated physical and mental health, although notably there was no difference in attrition between households randomized the intervention and those not (Table S10, appendix). A total of 77 villages with 4,410 households were randomized to the random targeting strategy and 22 villages with 1,223 households were randomized to the control arm with no treated households. Among the 77 villages assigned to the random targeting strategy, 11 villages were randomly assigned to each treatment dosage, or proportion of households targeted (figure 1, see appendix figure S1 for parent trial profile). Across all treatment dosages, a total of 1,815 households with 5,305 individuals were randomized to receive the intervention. Villages had between 48 and 571 participants each, and households assigned to treatment received the intervention's educational household visits between November, 2016, and August, 2018.

At baseline, 8,700 out of 16,301 (53%) participants were women. The mean age was 32·9 years (SD 17·2); 9,404 (58%) respondents were married or in a civil union; and 8,589 out of 13,881 (62%) participants had less than a primary education. Of the parents that completed an endline survey, 630 reported having a child after the intervention delivery period (September 1, 2018) and 1,133 reported having a child that was five or under at baseline. Descriptive statistics of the sample arranged by treatment

For peer review only - http://bmjopen.bmj.com/site/about/guidelines.xhtml

arm are reported in table 1. Village and household characteristics were balanced across treatment arms at baseline.

For peer review only

For peer review only - http://bmjopen.bmj.com/site/about/guidelines.xhtml

Table 1. Baseline characteristics of the intention-to-treat population in the pure control and random-assignment arms

	Pure control group (n=3600)	Random-assignment group (n=12701)	
		Within-cluster control (n=7396)	Targeted (n=5305)
Household characteristics			
**Wealth index**			
Quintile 1	524 (15%)	1194 (17%)	896 (17%)
Quintile 2	558 (16%)	1394 (19%)	1073 (21%)
Quintile 3	592 (17%)	1596 (22%)	983 (19%)
Quintile 4	819 (22%)	1487 (21%)	1152 (22%)
Quintile 5	1080 (30%)	1566 (21%)	1116 (21%)
**Households**	1223	2595	1815
**Household size***	4·28 (2·1)	4·35 (2·1)	4·52 (2·2)
Child characteristics			
**Youngest child's age, years***	6·04 (5·2)	5·44 (4·8)	5·51 (4·9)
**Past month illness (child 5 or under)***			
Respiratory illness	281 (30%)	674 (33%)	527 (36%)
Diarrheal illness	124 (13%)	338 (16%)	251 (17%)
Respondent characteristics			
**Sex**			
Female	1908 (53%)	3964 (54%)	2828 (53%)
Male	1692 (47%)	3432 (46%)	2477 (47%)

For peer review only - http://bmjopen.bmj.com/site/about/guidelines.xhtml

**Age**	33·2 (17·4)	32·8 (17·1)	32·8 (17·2)
**Education**			
Less than primary	1845 (60%)	3907 (62%)	2836 (63%)
Primary or greater	1206 (40%)	2438 (38%)	1680 (37%)
**Marital status**			
Single	1274 (35%)	2672 (36%)	1959 (37%)
Married or Civil Union	2120 (59%)	4254 (58%)	3030 (57%)
Separated/Divorced/Widowed	206 (6%)	470 (6%)	316 (6%)
**Indigenous status***			
No	2807 (92%)	5489 (87%)	3932 (87%)
Yes, Maya Chorti	243 (8%)	851 (13%)	573 (13%)
Yes, other indigenous group	1 (<1%)	4 (<1%)	2 (<1%)
**Health***			
Self-rated physical health, 1 (excellent)–5 (poor)†	3·21 (1·1)	3·25 (1·1)	3·24 (1·1)
Self-rated mental health, 1 (excellent)–5 (poor)†	3·08 (1·1)	3·12 (1·1)	3·14 (1·1)

Data are n, n (%), or mean (SD). *Information only available from respondents who completed baseline survey (n=13881). †Health questions from single item assessing general self-rated health (SF-1).[28]

For peer review only

Significant total effects of the intervention on several primary and secondary outcomes are displayed in figure 2. Direct, indirect, and total effects estimated from outcomes at endline as well as baseline levels for each outcome are reported in Table S1 (appendix). We focus here on total effects of the intervention pooled across all dosages, which account for both the direct effect of the intervention and any spillover effects within each village (i.e., the effect of having other treated individuals in the same village upon a person who did not receive the intervention). Although all three effects are estimated, the total effects are emphasized as the best average estimate of the intervention's impact in the study population and as the metric most analogous to treatment effects reported from other comparable educational intervention trials.

Compared to parents of children born after the intervention period who did not receive the intervention, parents of children who received it showed significant improvements in primary outcome practices related to postnatal care, proper umbilical cord care, and breastfeeding. Specifically, the total effect of the intervention led to a 16·4% (95% CI 3·1%–29·8%, p=0·016) increase in the probability that parents had their newborn's health checked by a professional in a health facility within three days of birth; a 19·6% (95% CI 4·2%–35·1%, p=0·013) increase in the probability that parents did not wrap a fajero around the umbilical cord in the first week after birth; and a 8·9% (95% CI 0·3%–17·5%, p=0·043) increase in the probability that the mother breastfed immediately after birth (figure 2; Table S1, appendix). These pooled effects corresponded to an absolute change in behavior before and after the intervention of +13·9% targeted vs +5·8% untargeted for having newborn's health checked within three days; +37·9% targeted vs

For peer review only - http://bmjopen.bmj.com/site/about/guidelines.xhtml

+16·1% untargeted for not wrapping a fajero around the umblical cord within the first week; and +5·1% targeted vs -4·1% untargeted for mother's breastfeeding immediately after birth (Table S9, appendix). Among parents of children born after the intervention period, we found no significant total effect of the intervention on practices related to preparing a birth plan, seeking care for pregnancy danger signs, facility based birth, mothers seeking postnatal check-up or care for postnatal danger signs, keeping the newborn wrapped or holding them skin-to-skin after birth, exclusively breastfeeding for six months, or father involvement in newborn care. Additionally, we found no significant total effects on certain measures of diarrhea or respiratory illness treatment in children five and under, folic acid use among women 15 and over, or contraceptive use among all respondents.

In addition to the changes in actual behaviors related to maternal and child health, we also assessed changes in the knowledge and attitudes related to those behaviors as secondary outcomes. Changes in knowledge or attitudes may precede changes in practices, and we analyzed responses of all participants regardless of whether they had a child since the intervention. We noted improvements in knowledge or attitudes related to the significant practices in the areas of postnatal care, proper umbilical cord care, and breastfeeding among the treated. Specifically, the total effect of the intervention was a 2·7% (95% CI 1·0%–4·3%, p=0·0014) increase in the probability that a participant identified correct ways to provide newborn care, which included getting their health checked by a medical professional; a 9·1% (95% CI 4·0%–14·2%, p=0·0005) increase in the probability that a participant identified proper cord care methods, which included

For peer review only - http://bmjopen.bmj.com/site/about/guidelines.xhtml

not wrapping a fajero; and a 14·0% (95% CI 8·4%–19·6%, p<0·0001) increase in the probability a participant believed newborns should not be given chupones when breastfeeding in the first six months (figure 2; Table S1, appendix).

Additionally, we found significant total effects for knowledge and attitudes related to folic acid use before pregnancy; methods of savings as part of the birth plan; certain pregnancy danger signs (i.e., fever, swelling of hands/face/feet); fathers accompanying mothers to prenatal care visits; mothers receiving postnatal check-ups within three days of birth; certain postnatal danger signs for mother (i.e., heavy vaginal bleeding, fever); identifying appropriate diarrhea treatment methods including correct use of zinc; and delaying pregnancy until 18 years or older (figure 2; Table S1, appendix). We found no significant total effect of the intervention on knowledge or attitudes related to seeking prenatal care in the first trimester; facility-based birth; the majority of the pregnancy danger signs; the majority of the postnatal and newborn danger signs; immediate breastfeeding; father waiting at birth location or caring for children when they are sick; diarrheal illness prevention; or respiratory illness prevention and danger signs (Table S1, appendix).

No adverse events associated with the intervention were found. We performed additional analyses as a check for robustness of our results, which included looking exclusively at the response of the mothers, adjusting for demographic characteristics of respondents, adjusting for baseline behaviors, and excluding those who moved between treatment arms during the study (Table S2–Table S7, appendix).

For peer review only - http://bmjopen.bmj.com/site/about/guidelines.xhtml

Finally, we incidentally note that two outcomes were associated with significant spillover effects on those who were untreated, which are effects of having others in one's village receive the intervention: father holding the child, and child with past-month diarrheal illness being treated with zinc (Table S1, appendix). Our study design, which included variation in the fraction of people treated per village and the presence of a pure control group of wholly untreated villages allowed us to perform such an analysis. A comprehensive investigation of how spillover effects may depend on treatment dosage and effects specific to a village's social network structure are the subject of other work.

Quality assessment of intervention delivery

Monitoring data on session implementation showed that the intervention was delivered as intended, with up to 22 monthly sessions planned per intervention village. Of the 1,815 households initially targeted for intervention at baseline, 1,225 (67%) households received modules for all 15 intervention topics as designed. Of the targeted households that completed endline surveys, the average number of sessions delivered per household was 15·2 (SD 7·4), with an average group size of 1·1 (SD 0·7) in attendance. Of the parents of children born after the intervention period who completed endline surveys, mothers were present for an average of 9·0 educational modules (SD 7·8) while fathers were present for an average of 2·4 educational modules (SD 2·8). Finally, as a method of assessing intervention delivery quality, we administered three survey questions designed as riddles for which the answer was based specifically on

For peer review only

information covered in a session visit. We found significant ($p<0{\cdot}0001$) effects of the intervention on answers to all quality-check questions (Table S8, appendix).

Discussion

In this multi-arm cluster-randomized controlled trial, we implemented an intervention targeting health outcomes for mothers, neonates, and children which featured validated counseling and negotiation techniques delivered by community health workers to households in rural Honduras over the course of 22 months. Our results show improvements in many, but not all, practices related to postnatal care, proper umbilical cord care, and breastfeeding. Parents with children born after receiving the intervention were 16% more likely to report that their newborn's health was checked by a professional in a health facility within three days of birth; 20% more likely to report not using a fajero on the newborn's umbilical cord; and 9% more likely to report their child was breastfed immediately. Importantly, whenever we noted a significant effect on behavior, we also saw an effect on the corresponding knowledge or attitude. We generally noted that our intervention seemed to have more impact on knowledge and attitudes than on practices, which is consistent with the theory that changes in knowledge and attitudes may be required first in order to seed changes in behavior and that such changes are easier to affect.

Although our study design randomized villages to different dosages, or proportions of households treated, we estimate pooled effects across all dosages. The primary contribution of our findings, therefore, is that we provide estimates of average treatment

For peer review only - http://bmjopen.bmj.com/site/about/guidelines.xhtml

effects. Such estimates represent the expectation policymakers can have on average in a setting where different proportions of the community are targeted for intervention (including observational settings in which the intervention is delivered non-randomly). Overall, however, the majority of pooled spillover effect estimates are negligible. Spillover effects may be heterogenous across dosage of households treated, social network structure, or individual treatment status. Further investigation of spillover effects in this study remains the subject of other work.

The design and implementation of this trial had several strengths. The experimental design was robust and involved a two-stage randomization in which we assigned village clusters to treatment dosages and then further assigned households within those clusters to treatment. Similar groups were attained through balancing across a number of village- and household-level covariates captured at the baseline stages of the project. In varying the dosage of households treated and randomizing some villages to a pure control design in which no households are treated, we were able to estimate pooled spillover effects, a feature uncommon to other large-scale public health intervention evaluation. Careful monitoring and training of CCAs through partnerships with IDB resulted in unbiased, high-quality data acquisition across four years from baseline to endline. The selected behavior change strategy was a modified version of the Timed and Targeted Counseling methodology with the P Process tool, which together allowed CCAs to use dialogue counseling methods and interactive story-telling to assess current needs and practices of intervention recipients and negotiate on progressive improvement.[20,21] The approach has been documented as effective in changing a

For peer review only - http://bmjopen.bmj.com/site/about/guidelines.xhtml

variety of behaviors aligned with the UN's Sustainable Development Goals and was successfully tailored to the conditions of the current study.[7,8,29] Finally, this study assessed a wider array of outcomes than other trials of its kind, which helped to determine the impact of the intervention on various points of the pregnancy and infancy timeline.

The study also had a number of limitations. In a traditional behavior change communication intervention, households would be targeted based on where pregnant women or young children live, and community members would be exposed to the intervention messages as many times as possible (e.g., in-person meetings, radio messages, flyers, etc.). Neither of these were possible given our randomization scheme (and the parent trial's larger goal of measuring spread of information and behavior via social networks). Although we made significant attempts to capture a diversity of villages in our study, some were excluded on the basis of accessibility and safety. An additional consideration limiting generalizability was that respondents who were lost to follow-up were more likely to be younger, male, single, have a higher education, not identify with an indigenous group, and have greater self-ratings of physical and mental health. While we attempted to use objective measures whenever possible, both primary and secondary outcomes were assessed using self-report. Additionally, some practices targeted by our intervention were already performed at high rates in Honduras prior to our study, introducing ceiling effects to our intervention (e.g., both DHS and our baseline survey show 86% of women began breastfeeding immediately after birth; Table S1, appendix).[13] Finally, our study area was subject to co-intervention, the most

For peer review only - http://bmjopen.bmj.com/site/about/guidelines.xhtml

notable of which was the Salud Mesoamérica Initiative (SMI). SMI comprised both the Ministry of Health of Honduras and IDB and included implementation of both demand and supply-side interventions to improve maternal and child health within the Department of Copán.[23] All municipalities, and therefore villages, within Copán that were included in the current study were also included in SMI, and all were randomized to SMI's intervention arm. This suggests that both treated and untreated households in our trial experienced equal impact from SMI's co-intervention, and also that this trial's interaction with SMI within treated households may have slightly reduced the effect of our intervention. We took care to ensure that our intervention had unique and specific messaging that was evaluated through our outcome measures, and we note that the presence of co-intervention improves our study's generalizability, as it is rare that large-scale public health interventions occur in isolation.

Many of our findings are consistent with similar home-based intervention packages targeting maternal, neonatal, and child health through behavior change communication strategies. Our review of the literature suggests that this is one of the largest RCTs of a maternal and child health intervention covering a full range of outcomes to take place in the Americas. Three studies reporting interventions across rural communities in Southern Ethiopia, India, Cambodia, Kenya, and Zambia which used the ttC counseling strategy found a 10% improvement in immediate breastfeeding, a 25% improvement in exclusive breastfeeding within first six months, a doubling in family planning, and a 7.5-fold increase in skilled birth attendance.[7–9] One intervention also used Community Scorecards as a mechanism of social accountability, which was not used in the present

For peer review only

study, and all used fewer home visits overall.[9] The evaluation of breastfeeding in the current trial was largely based on the Alive & Thrive initiative, which relies on interpersonal communication and community mobilization for home-visit counseling to improve breastfeeding and complementary infant feeding practices in low-income regions.[10] Alive & Thrive's most recent trial in rural Burkina Faso used messaging about feeding through both conversation and physical materials at health center consultations, women's support groups, home visits, and public community events, and targeted pregnant or breastfeeding women and their family members. Our findings of impacts on knowledge and attitudes about exclusive breastfeeding are in agreement with their trial results, with their trial showing additional effects on exclusive breastfeeding practices up to 39%.[10]

Even among trials like Alive & Thrive targeting similar outcomes with similar behavior change communication strategies, almost no other study (to our knowledge) had exclusive messaging by CHWs during in-person home visits, nor a targeting strategy that selected households regardless of current or recently pregnant mother status, like the current trial. Two quasi-experimental interventions in rural Indian and Ugandan villages—the Integrated Nutrition and Health Programme (INHP) and MANIFEST intervention, respectively—which used home visits by CHWs to promote prenatal, delivery, and newborn care practices, each reported significant improvements in outcomes related to prenatal check-ups, facility delivery, umbilical cord care, thermal care, and breastfeeding.[30,31] However, the INHP was designed as an non-governmental organization facilitation of a government program with the primary

For peer review only - http://bmjopen.bmj.com/site/about/guidelines.xhtml

outcome being the socioeconomic equity of changes in the targeted behaviors, while the MANIFEST intervention involved media communication and health care capacity-building components.[30,31] Additionally, almost no interventions included discussion of regional practices that may have cultural significance but be potentially harmful to the mother or newborn. Our study suggested that inclusion of such practices in our communication strategy was effective in impacting knowledge, attitudes, and practices related to fajero use, as well as knowledge and attitudes about the use of chupones.

While our randomization scheme and large sample size increased our confidence that observed differences in outcomes, or lack thereof, would be due to the intervention, further investigation into why change may or may not occur is warranted. For example, many of the primary outcomes were prevalent at baseline, including immediate breastfeeding (86%), avoiding harmful substances around umbilical cords (83%), and keeping the newborn warm and clothed after birth (99%) (Table S1, appendix). An even larger swath of secondary knowledge and attitudes outcomes were prevalent at the 80% level or above at baseline (Table S1, appendix). High baseline rates of certain practices, knowledge, and attitudes may limit the generalizability of the current study, and more work is needed to further clarify how a similar intervention may have a different impact in other communities, as well as how educational intervention packages in general might be tailored to account for the regional prevalence of behaviors which impact maternal and child health.

For peer review only

Additionally, the intervention was designed for behavior change and was therefore aimed at the demand-side conditions of health behaviors. In many cases, however, said behaviors may not only rely on prior practices, knowledge, and attitudes, but also on supply-side conditions such as the accessibility of healthcare resources or the quality of local health centers. For example, outcomes evaluated in the current study but which depend on supply-side conditions include folic acid use, preventative check-ups, care-seeking from medical professionals due to the experience of either postnatal or newborn danger signs, delivery in a health facility, and zinc treatment for diarrheal illness. Other outcomes were not as dependent on supply-side conditions and were therefore considered in the household's control and potentially more able to be impacted by a counseling intervention, such as preparing a birth plan, immediate and exclusive breastfeeding, withholding chupones and fajeros, keeping the newborn warm and clothed, and skin-to-skin contact with newborn.

Overall, a novel large-scale household-based counseling intervention package randomized at the village and household levels and delivered in a rural setting resulted in meaningful change in practices, knowledge, and attitudes related to preventative care-seeking for newborns, proper umbilical cord care, and immediate breastfeeding. Additional studies are needed to understand the underlying reasons for change in some but not all outcomes. In our opinion, the results of the trial are generalizable to similar communities in this region. Further research is also needed to assess the cost-effectiveness of this approach and its generalizability to other areas or practices. Finally, our two-stage randomization allowed us to identify distinct treatment effects, suggesting

For peer review only - http://bmjopen.bmj.com/site/about/guidelines.xhtml

that there are factors such as social network ties and community structure which can influence the uptake of behavior change. Further evaluation of the role of social network ties is needed to understand how, when people are given public health education, their response depends on how others around them are coping with similar challenges.

Contributors

Marcus Alexander, Nicholas A. Christakis, and Laura Forastiere formulated the hypothesis and conceptualized this study. World Vision Honduras directed the design of the intervention content, supervised by IDB, and the content was then reviewed by HNL to ensure that it met study criteria. Nicholas A. Christakis, Rennie Negron, Jennifer Nelson, and Emma Iriarte were involved in survey design. Rennie Negron trained data collection teams and managed study implementation, and Jennifer Nelson and Emma Iriarte oversaw intervention delivery. Nicholas A. Christakis and Rennie Negron managed data collection. William Oles, Marcus Alexander and Laura Forastiere analyzed the data, and Marcus Alexander, William Oles, Laura Forastiere and Nicholas A. Christakis interpreted the analyses. William Oles did the literature search. William Oles, Marcus Alexander and Laura Forastiere produced the figures and tables, and, with Rennie Negron and Nicholas A. Christakis wrote the first draft. William Oles, Laura Forastiere, and Rennie Negron wrote the supplementary appendix. All authors reviewed and provided input to the final draft. Nicholas A. Christakis and Laura Forastiere had final responsibility for the decision to submit for publication.

Declaration of interests

For peer review only - http://bmjopen.bmj.com/site/about/guidelines.xhtml

For peer review only

Nicholas A. Christakis reports grants from the Bill and Melinda Gates Foundation and the Tata-Yale Alliance. The Bill and Melinda Gates Foundation funded the intervention through a grant to IDB supervised by Jennifer Nelson and Emma Iriarte. All authors declare no competing interests.

Data sharing

The de-identified datasets generated during the study along with the statistical plan and analytic code will be available from the corresponding author on reasonable request at the end of the project after all planned manuscripts have been accepted for publication. We will make the data without identifiers available to users only under a data-sharing agreement that provides for: (1) a commitment to using the data only for research purposes and not to make the attempt of identifying any individual participant; (2) a commitment to securing the data in case there are still some sensitive variables after the identifiers have been removed, by using appropriate computer technology; (3) a commitment to destroying or returning the data after analyses are complete; and (4) a commitment to not publish any information that is not treated at the aggregate level so that no specific characteristics can be linked to small communities.

For peer review only

Acknowledgements

We acknowledge our partners at the Bill and Melinda Gates Foundation and all staff of the Human Nature Lab at Yale University, including: HNL Lab and Project Director Thomas Keegan; Data Scientist Liza Nicoll; software developers Mark McKnight and Wyatt Israel; research coordinators Jai Broome, Catherine Henry, Kevin Garcia,

For peer review only - http://bmjopen.bmj.com/site/about/guidelines.xhtml

Petergaye Murray, Samy Galvez, Maria Vassimon de Assis; as well as research assistants John Sang Won Lee, Selena Lee, and Drew Prinster. We acknowledge our staff at IDB including Dr. Hugo Godoy, IDB Health Specialist in Honduras, the Salud Mesoamerica Initiative Team, especially Maria Paola Zuniga, Mauricio Perez Calvo, Jose Ignacio Mata, and María Elena Ordóñez, IDB consultant, and partners from the World Vision Honduras Team including Karen Ramos, Lesbia Garcia, Gertrudis Medrano, and the Dimagi Team who supported the development of the community health application used during the intervention. We also acknowledge our partners at the Ministry of Health Honduras and all our local partners and field data collection team, and we thank all study participants.

Role of the funding source

The trial was an investigator-initiated study supported by grants from the Bill & Melinda Gates Foundation and the Tata Group. The funding sources did not have any role in the design, conduct, or analysis of the study, the writing of the report, or the decision to submit it for publication. All authors had full access to all the data in the study. NAC and LF had final responsibility for the decision to submit for publication.

Ethics Approval

All participants provided informed consent at the time of data collection before randomization. Ethics approval was obtained from the Institutional Review Board (Protocol #1506016012) at Yale University in New Haven, Connecticut, USA and the Ministry of Health of Honduras.

For peer review only

References

1 GBD 2015 Child Mortality Collaborators. Global, regional, national, and selected subnational levels of stillbirths, neonatal, infant, and under-5 mortality, 1980–2015: a systematic analysis for the Global Burden of Disease Study 2015. Lancet 2016;388:1725–74. doi: 10.1016/S0140-6736(16)31575-6

2 Liu L, Oza S, Hogan D, et al. Global, regional, and national causes of child mortality in 2000–13, with projections to inform post-2015 priorities: an updated systematic analysis. Lancet 2015;385:430–40. doi: 10.1016/S0140-6736(14)61698-6

3 World Health Organization (WHO). WHO recommendations on health promotion interventions for maternal and newborn health [online]. 2015. https://www.who.int/publications-detail-redirect/9789241508742 (accessed 13 Aug 2023).

4 World Health Organization (WHO). WHO recommendations on newborn health: guidelines approved by the WHO Guidelines Review Committee [online]. 2017. https://www.who.int/publications-detail-redirect/WHO-MCA-17.07 (accessed 13 Aug 2023).

5 Kumar V, Mohanty S, Kumar A, et al. Effect of community-based behaviour change management on neonatal mortality in Shivgarh, Uttar Pradesh, India: a cluster-randomised controlled trial. Lancet 2008;372:1151–62. doi: 10.1016/S0140-6736(08)61483-X

6 Gilmore B, McAuliffe E. Effectiveness of community health workers delivering preventive interventions for maternal and child health in low- and middle-income countries: a systematic review. BMC Public Health 2013;13:847. doi: 10.1186/1471-2458-13-847

7 Beyero M, Reider K, Mekonnen Y. Effect of timed and targeted counseling (ttC) by peer mothers in changing infant and young child feeding practices among mothers of children 0 to 18 months in four districts in Southern Ethiopia. The FASEB Journal 2013;27. doi: 10.1096/fasebj.27.1_supplement.620.9

8 World Vision International. Timed and Targeted Counselling (TTC): A Service Package of the CHW Project Model [online]. 2020. https://www.wvi.org/sites/default/files/2020-06/TTC%20CPM%20Brief%20-%20June%202020.pdf (accessed 13 Aug 2023).

9 Edward A, Krishnan A, Ettyang G, et al. Can people-centered community-oriented interventions improve skilled birth attendance? Evidence from a quasi-

For peer review only

For peer review only - http://bmjopen.bmj.com/site/about/guidelines.xhtml

experimental study in rural communities of Cambodia, Kenya, and Zambia. BMC Pregnancy and Childbirth 2020;20:1–514. doi: 10.1186/s12884-020-03223-0

10 Cresswell JA, Ganaba R, Sarrassat S, et al. The effect of the Alive & Thrive initiative on exclusive breastfeeding in rural Burkina Faso: a repeated cross-sectional cluster randomised controlled trial. Lancet Global Health 2019;7:e357–65. doi: 10.1016/S2214-109X(18)30494-7

11 United Nations Inter-Agency Group for Child Mortality Estimation (UN IGME). Under-five mortality rate/Neonatal mortality rate - Total. UN Inter-agency Group for Child Mortality Estimation [dataset]. 2023. https://childmortality.org/data (accessed 13 Aug 2023).

12 Pan American Health Organization (PAHO). Health in the Americas – Honduras [online]. 2017. https://www.paho.org/salud-en-las-americas-2017/wp-content/uploads/2017/09/Print-Version-English.pdf (accessed 20 Aug 2023).

13 US Agency for International Development (USAID). Honduras. DHS Program: STATcompiler [dataset]. 2012. https://www.statcompiler.com/en/ (accessed 13 Aug 2023).

14 United Nations Maternal Mortality Estimation Inter-Agency Group (MMEIG). Trends in maternal mortality 2000 to 2020 [online]. 2023. https://www.who.int/publications/i/item/9789240068759 (accessed 20 Aug 2023).

15 Pan American Health Organization (PAHO). Health Situation in the Americas: Core Indicators [online]. 2018. https://iris.paho.org/bitstream/handle/10665.2/49511/CoreIndicators2018_eng.pdf?sequence=1&isAllowed=y (accessed 20 Aug 2023).

16 Griffiths M, McGuire JS. A New Dimension for Health Reform–The Integrated Community Child Health Program in Honduras. In: La Forgia GM, eds. Health System Innovations in Central America: Lessons and Impact of New Approaches. Washington, DC: The International Bank for Reconstruction and Development / The World Bank 2005:173–96.

17 Shakya HB, Stafford D, Hughes DA, et al. Exploiting social influence to magnify population-level behaviour change in maternal and child health: study protocol for a randomised controlled trial of network targeting algorithms in rural Honduras. BMJ Open 2017;7:e012996. doi: 10.1136/bmjopen-2016-012996

18 World Vision International, Inter-American Development Bank. Project REDES: Qualitative Evaluation [online]. 2019. https://socialdigital.iadb.org/sites/default/files/2019-10/Evaluaci%C3%B3n%20Cualitativa%20EV_0.pdf (accessed 20 Aug 2023).

For peer review only - http://bmjopen.bmj.com/site/about/guidelines.xhtml

19 World Vision International, Inter-American Development Bank (IDB). Project REDES: Final Execution Report. 2019. https://socialdigital.iadb.org/sites/default/files/2019-10/Final%20Report%20REDES%20260919.pdf (accessed 23 Aug 2023).

20 World Vision International. Timed and Targeted Counseling (ttC) [online]. 2016. https://www.wvi.org/health/timed-and-targeted-counseling-ttc (accessed 20 Aug 2023).

21 Health Communication Capacity Collaborative. The P Process: Five Steps to Strategic Communication [online]. 2013. https://www.healthcommcapacity.org/wp-content/uploads/2014/04/P-Process-Brochure.pdf (accessed 20 Aug 2023).

22 Inter-American Development Bank (IDB). ttC: Timed and Targeted Counseling APP for Community Health Workers [online]. 2019. https://socialdigital.iadb.org/en/sph/solutions/ttc-timed-and-targeted-counseling-app-community-health-workers (accessed 20 Aug 2023).

23 Mokdad AH, Palmisano EB, Zuniga-Brenes P, et al. Supply-side interventions to improve health: Findings from the Salud Mesoamerica Initiative. PloS One 2018;13:e0195292–e0195292. doi: 10.1371/journal.pone.0195292

24 Lungeanu A, McKnight M, Negron R, et al. Using Trellis software to enhance high-quality large-scale network data collection in the field. Social Networks 2021;66:171–84. doi: 10.1016/j.socnet.2021.02.007

25 Baird S, Bohren JA, McIntosh C, et al. Optimal Design of Experiments in the Presence of Interference. The Review of Economics and Statistics 2018;100:844–60. doi: 10.1162/rest_a_00716

26 Hudgens MG, Halloran ME. Toward Causal Inference With Interference. Journal of the American Statistical Association 2008;103:832–42. doi: 10.1198/016214508000000292

27 Kim DA, Hwong AR, Stafford D, et al. Social network targeting to maximise population behaviour change: a cluster randomised controlled trial. Lancet 2015;386:145–53. doi: 10.1016/S0140-6736(15)60095-2

28 DeSalvo KB, Bloser N, Reynolds K, et al. Mortality prediction with a single general self-rated health question: A meta-analysis. Journal of General Internal Medicine 2006;21:267–75. doi: 10.1111/j.1525-1497.2005.00291.x

29 Yun Y. Family Member Participation In Community Health Worker Intervention and Maternal Health Behavior: a Study of World Vision's Timed and Targeted Counseling from Cambodia, Guatemala, Kenya, and Zambia [online]. 2018. https://ecommons.cornell.edu/handle/1813/57397 (accessed 23 Aug 2023).

For peer review only - http://bmjopen.bmj.com/site/about/guidelines.xhtml

30 Baqui AH, Rosecrans AM, Williams EK, et al. NGO facilitation of a government community-based maternal and neonatal health programme in rural India: improvements in equity. Health Policy and Planning 2008;23:234–43. doi: 10.1093/heapol/czn012

31 Ekirapa-Kiracho E, Muhumuza Kananura R, Tetui M, et al. Effect of a participatory multisectoral maternal and newborn intervention on maternal health service utilization and newborn care practices: a quasi-experimental study in three rural Ugandan districts. Global Health Action 2017;10:1363506–28. doi: 10.1080/16549716.2017.1363506

For peer review only

For peer review only - http://bmjopen.bmj.com/site/about/guidelines.xhtml

Figure captions:

Figure 1: Trial profile: A population-level cluster-randomized controlled trial of maternal and child health intervention to promote behavior change in Honduras.

Figure 2: Significant total, direct, and indirect effects of the RCT of maternal and child health intervention on prenatal/postnatal care, breastfeeding, and diarrhea management.

For peer review only

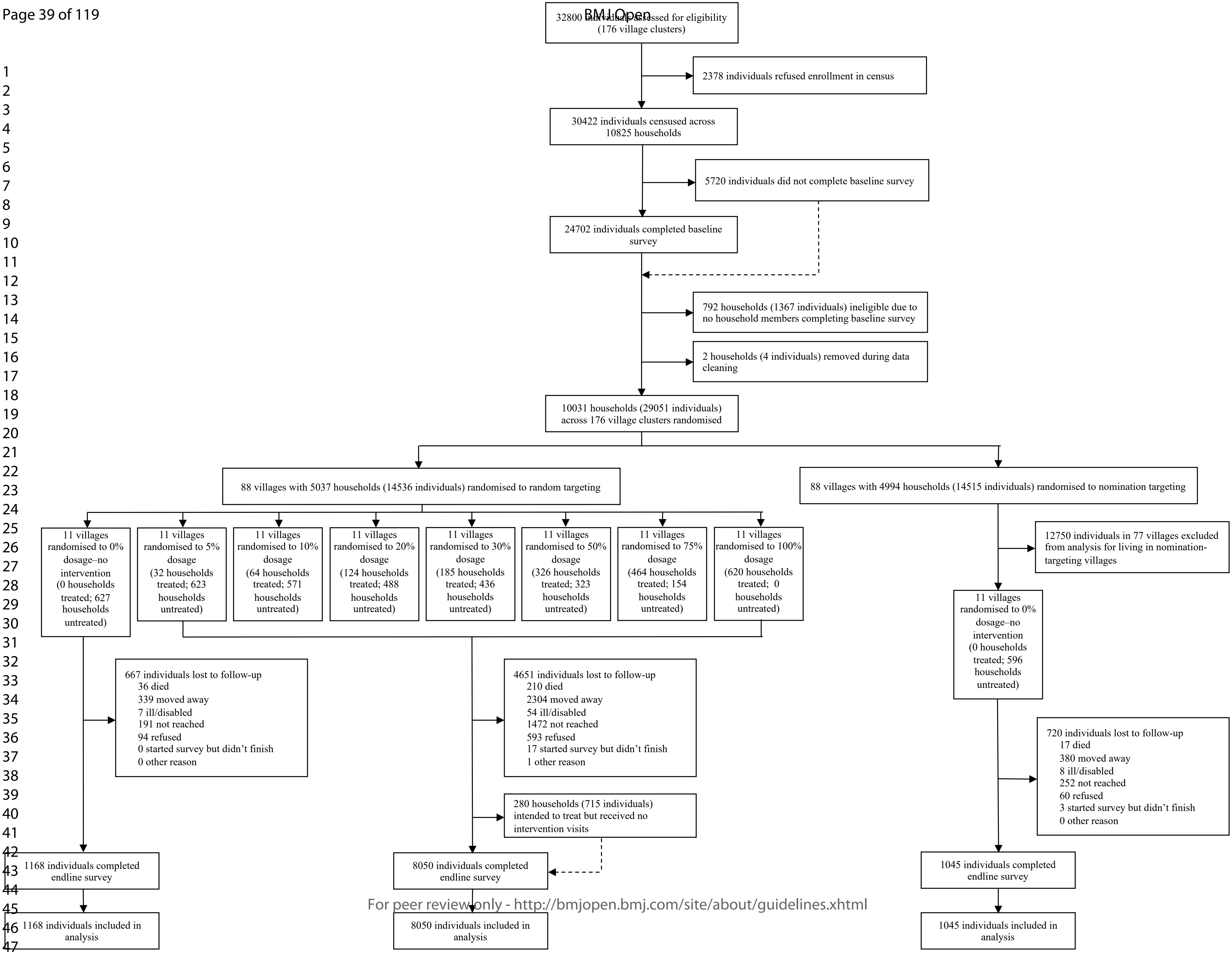
32800 individuals assessed for eligibility (176 village clusters)
2378 individuals refused enrollment in census
30422 individuals censused across 10825 households
5720 individuals did not complete baseline survey
24702 individuals completed baseline survey
792 households (1367 individuals) ineligible due to no household members completing baseline survey
2 households (4 individuals) removed during data cleaning
10031 households (29051 individuals) across 176 village clusters randomised
88 villages with 5037 households (14536 individuals) randomised to random targeting
88 villages with 4994 households (14515 individuals) randomised to nomination targeting
11 villages randomised to 0% dosage–no intervention (0 households treated; 627 households untreated)
11 villages randomised to 5% dosage (32 households treated; 623 households untreated)
11 villages randomised to 10% dosage (64 households treated; 571 households untreated)
11 villages randomised to 20% dosage (124 households treated; 488 households untreated)
11 villages randomised to 30% dosage (185 households treated; 436 households untreated)
11 villages randomised to 50% dosage (326 households treated; 323 households untreated)
11 villages randomised to 75% dosage (464 households treated; 154 households untreated)
11 villages randomised to 100% dosage (620 households treated; 0 households untreated)
12750 individuals in 77 villages excluded from analysis for living in nomination-targeting villages
11 villages randomised to 0% dosage–no intervention (0 households treated; 596 households untreated)
667 individuals lost to follow-up
36 died
339 moved away
7 ill/disabled
191 not reached
94 refused
0 started survey but didn’t finish
0 other reason
4651 individuals lost to follow-up
210 died
2304 moved away
54 ill/disabled
1472 not reached
593 refused
17 started survey but didn’t finish
1 other reason
280 households (715 individuals) intended to treat but received no intervention visits
720 individuals lost to follow-up
17 died
380 moved away
8 ill/disabled
252 not reached
60 refused
3 started survey but didn’t finish
0 other reason
1168 individuals completed endline survey
8050 individuals completed endline survey
1045 individuals completed endline survey
1168 individuals included in analysis
8050 individuals included in analysis
1045 individuals included in analysis

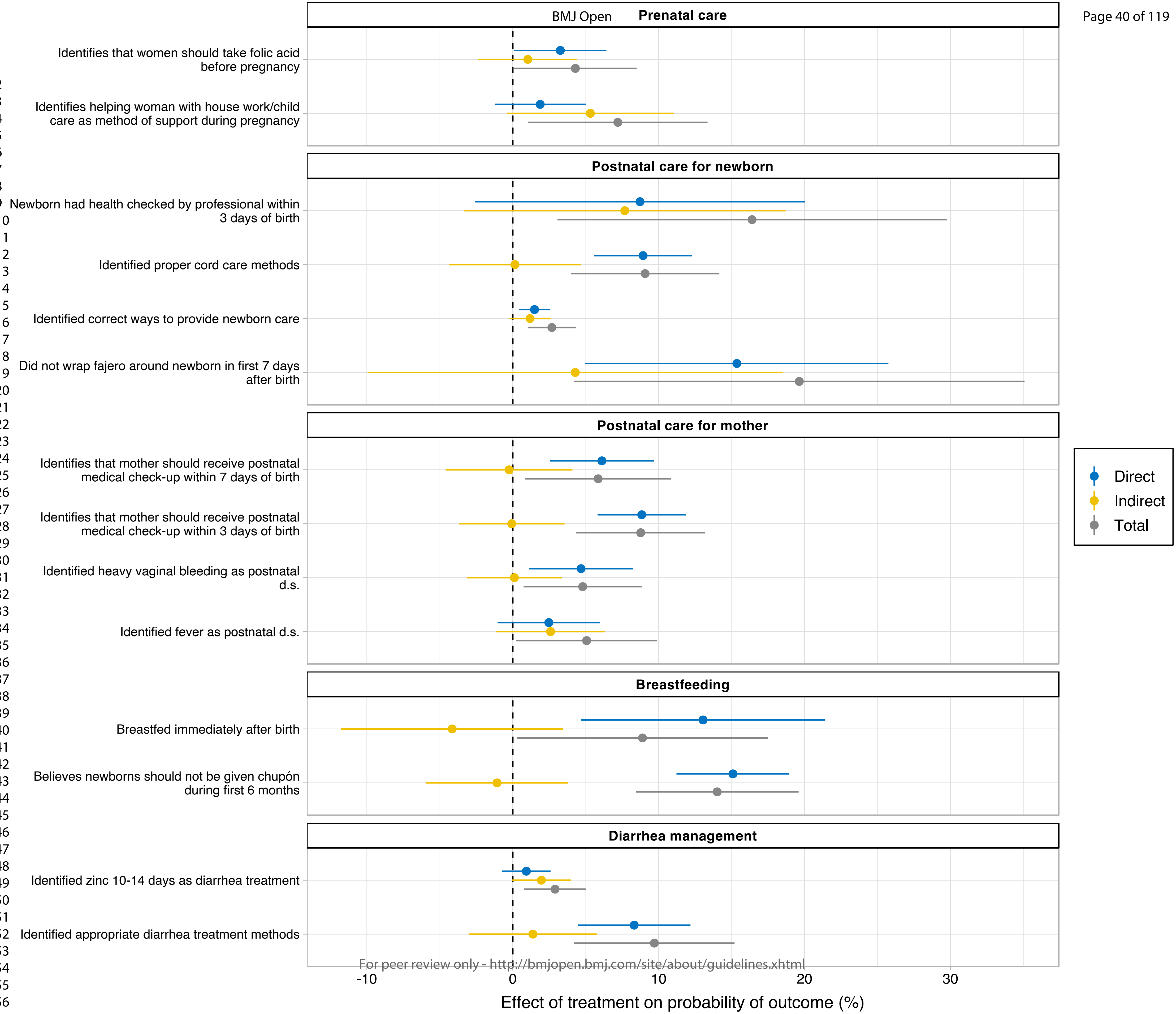

For peer review only - http://bmjopen.bmj.com/site/about/guidelines.xhtml

ONLINE SUPPLEMENTARY APPENDIX

Maternal and child health intervention to promote behavior change: a population-level cluster-randomized controlled trial in Honduras

William Oles, BS

Marcus Alexander, PhD

Rennie Negron, MPH

Jennifer Nelson, MPH

Emma Iriarte, MD

E. M. Airoldi, PhD

Nicholas A. Christakis, MD, PhD, MPH

Laura Forastiere, PhD

For peer review only

For peer review only - http://bmjopen.bmj.com/site/about/guidelines.xhtml

Table of Contents

Pilot work **3**

Field operations **3**

Community engagement **3**

Missing data and attrition **3**

Blocking of villages and randomisation **3**

Assignment of households to treatment/control in each village **4**

Intervention sessions **5**

Statistical methods **5**

Additional analysis **7**

Tables **9**

Table S1. Pooled direct, indirect, and total effect estimates of intervention on maternal, child, and neonatal outcomes 9

Table S2. Pooled total effect estimates of intervention on maternal, child, and neonatal outcomes, mothers responses for practice outcomes only 20

Table S3. Pooled total effect estimates of intervention on maternal, child, and neonatal outcomes, estimated on sub-sample of respondents who did not move between villages during study 22

Table S4. Pooled total effect estimates of intervention on maternal, child, and neonatal outcomes, with adjustment for baseline demographic characteristics 29

Table S5. Pooled total effect estimates of intervention on maternal, child, and neonatal outcomes, with adjustment for baseline outcome 36

Table S6. Pooled total effect estimates of intervention on maternal, child, and neonatal outcomes, with adjustment for baseline demographic characteristics and baseline outcome 43

Table S7. Pooled total effect estimates of intervention on maternal, child, and neonatal outcomes, with adjustment for baseline demographic characteristics and baseline outcome, estimated on sub-sample of respondents who did not move between villages during study 50

Table S8. Pooled total effect estimates of intervention on maternal, child, and neonatal outcomes, intervention module receipt defined as treatment, with adjustment for baseline demographic characteristics 57

Table S9. Absolute changes in primary and secondary outcomes from baseline to endline survey 65

Table S10. Analysis of differences in attrition between baseline and endline surveys 76

References **77**

For peer review only - http://bmjopen.bmj.com/site/about/guidelines.xhtml

Pilot work

Prior to the rollout of our community census and baseline survey efforts, we conducted 3 separate pilot surveys. The first pilot survey, conducted in the summer of 2014, was given to 1018 individuals in 2 towns in the Lempira Department, Honduras, of whom we were able to collect network, demographic, and social normative measures from 831. The second pilot survey was conducted in February 2015, and covered 4 small villages in the Copan region, and included 165 individuals with questions on networks, demographics, as well as reproductive, maternal, child and neonatal health knowledge, attitudes, norms, and behaviors. Our final pilot survey was conducted in May 2015, for which we enrolled 577 individuals. After each pilot we carefully analyzed the data for anomalies, including large number of missing responses, no variation in item responses, or responses that seemed irregular in ways suggesting response bias or misunderstanding of questions. The survey was then revised accordingly.

Our final survey instrument included both internally-derived scales revised using aforementioned pilot work as well as additional scales with well-described external validity and reliability. Examples of such scales include Self-Rated Health (SF-1), Patient Health Questionnaire-2 (PHQ-2), Alcohol Use Disorders Identification Test (AUDIT-C), Perceived Neighborhood Safety, and the Latin American and Caribbean Household Food Security Measurement Scale (ELCSA), among others.[1-4]

Field operations

We recruited and trained over 100 local surveyors to perform preliminary data collection and infrastructure development, complete participant recruitment and census enumeration, and conduct the survey interviews. We also established 4 field offices in geographically strategic locations to minimize travel time to and from the study villages. All offices were fully equipped with data collection tablets, netbook computers, printers, high speed internet, and a local server to insure secure download, synchronization and transfer of research data. All surveyors were extensively trained in the use of our survey instruments and software by US-based project managers and supervised by Honduran project coordinators with whom we had daily contact in relation to data collection and other implementation activities.

Community engagement

We developed valuable relationships with local government and health officials, participating in staff meetings for health center personnel and community health workers. We presented project plans and obtained approval of our field operations and data collection procedures from the Honduras Ministry of Health (MOH). The MOH reviewed our study protocols, consents and survey instruments and provided feedback. Our field teams have also met with local community leaders and indigenous council members to present study objectives in all study villages prior to beginning recruitment and data collection.

Missing data and attrition

Of the 32800 estimated eligible individuals in the study villages, 93% agreed to participate in the study, so bias due to failure to recruit participants is likely to be very low. At the follow-up and final stages, we made our best efforts to track study participants enrolled at baseline who discontinued for any reason, and we attempted to continue surveying participants who moved from one study village to another. Follow-up data and attrition are reported for each treatment arm in our comprehensive trial profile shown in supplemental figure S1. Of the 5633 households initially randomized in the trial that were a part of the treatment arms included in the current analysis, 4861 households (86%) had at least one respondent who completed the endline survey. A chi-square test of independence showed that there was no significant association between household treatment status and attrition, X^2 (1, N = 5633) = 0.80, p = .37. Further analysis of differences in attrition is reported in Table S10.

Blocking of villages and randomisation

Villages were assigned to treatment arms in an 8x2 factorial design—8 dosages of households treated per village (0, 0.05, 0.1, 0.2, 0.3, 0.5, 0.75, 1), and 2 targeting strategies (random target, friend nomination target). We ran a re-

For peer review only - http://bmjopen.bmj.com/site/about/guidelines.xhtml

randomization procedure that generated 10000 assignments of villages to treatment arms. This procedure generates assignments while controlling balance on the following variables: number of respondents in the village, average number of respondents in the household, latitude, longitude, elevation, time to health center, and time to maternity clinic.

This procedure prioritized matching the means and standard deviations of the 16 treatment arms, but also weights the third moments and cross-moments (to get at covariances). The procedure also assigns a score that summarizes balance. We checked how similar these assignments were. Of the 100 assignments with a better balance summary score, no two contained a pair of treatment arms that overlap in more than 8 elements. We also measured the average maximum overlap, defined as the maximum number of items that any pair of treatment arms have in common. On average, over the 100 assignments with better balance summary score, the maximum overlap was 3.985, indicating that the top 100 assignments of villages to treatment arms were are all quite different.

Focusing on the 100 assignments with better balance summary score, as candidate re-randomizations, we then ran a battery of statistical tests check for statistically significant imbalances. We focused on the following variables and tests quoted in parenthesis: number of respondents in the village (t test), average number of respondents in the household (t test), latitude (t test), longitude (t test), elevation (t test), time to health center (t test), time to maternity clinic (t test), number of households (t test), empirical distribution of household sizes (Kolmogorov-Smirnov test), village can be accessed in when raining (proportions), proportion of indigenous population (t test), time in minutes to main road (t test), village is a coffee producer (proportions), average number of women of reproductive age (t test), average age (t test), and percent male (t test).

For each of the 100 assignments we produced a table with these tests for each of 147 contrasts: 120 contrasts reported the p-values for imbalance in pairs of treatment arms for each of the covariates; 26 contrasts reported the p-values for marginal imbalance in pairs of levels of treatment; 1 contrast reported the p-values for marginal imbalance in the two nomination schemes. We selected assignment number 73, which had only one imbalance at significance 5%. The imbalance was for average age between treatment arms 3 (random target, 10% treated households) and 14 (friend nomination target, 50% treated households), which is an unimportant contrast for the purpose of conducting the analyses proposed in this study.

Assignment of households to treatment/control in each village
Dosage is to be understood as proportion of households treated per village, which was applied to the number of households in each village and the rounded to the nearest whole integer number. We built a social network where a tie meant that there was a nomination in any of the following name generators: personal private matters; spent free time; closest friend. For each village, we generated 10000 assignments of households to treatment in villages in the friend nomination arm. For each of these assignments we then computed balance for the following covariates: number of census respondents, number of women of reproductive age, number of children under the age of 12 who live in this household, existence of handwashing location observed by enumerator, household electricity, separate room in the house that is used as the kitchen, self-reported health status, and network degree centrality of the household. We also considered the following individual variables averaged at the household level: age, sex, and baseline self-reported health status of the individuals in the households.

Here, for each of the 144 villages assigned to a treatment arm in which the percentage of household treated differs from 0% and 100%, we only considered the contrast: treatment vs. control. We then selected the set of assignments of household to treatment and control in each of the 144 villages that minimized the number of tests for imbalance failed at 5%. These balanced sets of assignments constituted the final assignments of households to treatment in villages in the random target arm, but only the initial assignment of households to treatment in villages in the friend nomination arm. For villages in the friend nomination arm, we picked each of the households initially assigned to treatment, in turn, then picked a person in the household, then picked a tie at random among this person’s ties. Whenever we ran into a duplicate, we restarted the choice. This procedure generated a final set of assignments of households to treatment in villages in the friend nomination arm. For the purposes of analyses of direct effects of the intervention, households in villages in the friend nomination arm were excluded since they were targeted nonrandomly to receive the intervention.

For peer review only - http://bmjopen.bmj.com/site/about/guidelines.xhtml

Intervention sessions

Our community-based behavioral intervention took course over 22 months (November 2016–August 2018). It was comprised of a series of household-level counseling sessions targeting maternal, child, and neonatal practices using 15 distinct educational modules delivered over a maximum of 22 sessions. Each session was designed to last two hours and was centered around the timed and targeted counseling (ttC) method, which is a bottom-up counseling strategy in which family units are introduced to information about health practices and behaviors through storytelling. Throughout each story, families encounter "problem situations" and "positive stories," after which they reflect on the presentation and negotiate agreements about practices that are appropriate or necessary to improve health outcomes. Sessions also incorporated other forms of media including educational posters, songs, and physical illustrations. In each sessions, members of the family also complete standardized questionnaires for data collection which range from providing information about their home to specific questions related to knowledge, attitudes, and practices of targeted health behaviors.

The 15 educational modules along with the percentage of targeted households randomized to treatment in random-target villages which receiving the module are listed below. Table S8 contains additional analysis which incorporates specific module receipt into our estimation of treatment effects and also describes which modules were paired with primary and secondary outcomes.

Module	Description of module	% targeted households receiving the module (at least one session)
m1	Pregnancy care and prenatal care in first 12 weeks	77.1
m2	Preparing a birth plan	76.3
m3	Facility-based birth	72.9
m4	Importance of family planning	75.2
m5	Care of woman during childbirth and postpartum period and newborn care	74.0
m6	Care of woman during childbirth and postpartum period and newborn care	72.2
m7	Newborn care and danger signs	72.9
m8	Infant care: caring for children between 1–6 months	78.1
m9	Danger signs and seeking medical attention for acute respiratory infections and diarrheal illness	77.5
m10	Reproductive life plan	80.7
m11	Importance of folic acid for mom and newborn	80.1
m12	Importance of preventing teen pregnancy (<18 years old)	78.7
m13	Self-esteem, values, life goals	78.5
m14	Prevention of inter-partner violence; non-violent communication	79.6
m15	Zika prevention	77.3

Statistical methods

The opportunity to answer certain questions on our survey was determined by eligibility encoded into the survey design. For example, only mothers and fathers that had a child since the end of the intervention (September 1, 2018) were eligible to have a response included for the question about whether their child was exclusively breastfed for the first 6 months. For each outcome, we restricted the analysis to the respondents eligible to answer the related question. The respondent denominator for all outcomes analyzed can be found in Table S1. Each outcome variable was coded as binary: 0 if the response was the undesired outcome related to the intervention, and 1 if the response was the desired outcome related to the intervention. Responses of 'Refusal' for any survey question were excluded for the purposes of our analysis. Responses of 'Don't know' were coded as an undesired for outcomes related to knowledge but were otherwise excluded for outcomes about practices or attitudes. The unit of analysis was the individual respondent.

For peer review only - http://bmjopen.bmj.com/site/about/guidelines.xhtml

In our randomized experiment, first villages were either randomly assigned to different dosages, or proportion of treated households, or to no intervention, and then household were randomly assigned to the intervention according to the dosage assigned to their village. Here we only consider the villages assigned to random targeting. Because of this, three groups of respondents exist that can be compared: pure controls (those not targeted living in villages where no one is targeted); within-cluster controls (those not targeted living in villages where a nonzero dosage of people is targeted); and treated (those targeted). Due to the presence of interference among households, in order to estimate the effect of receiving the intervention, we needed to correct for the indirect effect affecting the untreated households in a village assigned to random targeting. We make the assumption that interference could be present within villages but not between them (also known as the partial interference assumption), and that a respondent's outcome depends on the proportion of treated household in the village in addition to its own treatment (stratified interference).[5,6] Under this assumption, we denote by Yic(Tic, Pc) the potential outcome of individual i in village c under individual treatment Tic and a proportion of treated households in the village c equal to Pc. We define the following causal effects:

$$TE(p): E[Y(1,p)-Y(0,0)]$$

$$DE(p): E[Y(1,p)-Y(0,p)]$$

$$SE(p): E[Y(0,p)-Y(0,0)]$$

TE(p) is the total effect of receiving the treatment while a proportion of p households is also treated in the village compare to the control condition without any intervention in the village, DE(p) is the average direct effect of receiving the treatment versus not in a village where a proportion of p households are treated, and SE(p) is the indirect (or spillover) effect of having a proportion of p households treated in the village for the untreated individuals. We have that for each p, the total effect TE(p) is the sum of the spillover effect on the untreated SE(p) and the direct effect DE(p). Here we focus on the total and direct effects, while accounting for spillover effects. To this end, we estimate the pooled total effect TE and the pooled direct effect DE averaging across dosages p. We estimated pooled effects by combining observations from all dosages in order to measure an average effect for the entire study population. The pooled total effect can be seen as the effect on the treated due to the intervention, while the pooled direct effect is the effect of directly receiving the treatment controlling for spillover effects.

To estimate these pooled causal effects in presence of interference, we used the following regression model:

$$\text{probit}(Y_{ic}) = \beta_0 + \beta_{total} T_{ic} + \beta_{indirect} S_{ic}$$

where Y_{ic} is the observed outcome of individual i in village c ,β_0 is the population average outcome, β_{total} is the pooled total effect, T_{ic} is the treated status of individual i in village c, $\beta_{indirect}$ is the pooled indirect effect, and S_{ic} is the within-village control status of individual i in village c.[5] Given the estimated coefficients we predicted the pooled potential outcomes for the treated individual, for the within-village control individuals, and for the pure controls. Total and spillover effects were estimated subtracting the corresponding potential outcomes.

Since the pooled total effect is the sum of the pooled indirect effect and the pooled direct effect, the pooled direct effect was computed by subtracting the estimated effects:

$$DE=TE-SE$$

In addition, this estimation of pooled effects required the addition of saturation weights, since targeted individuals are given disproportionately high weight in high-dosage villages and disproportionately low weight in low-dosage villages when observations are pooled. Saturation weights were calculated on the village-level based on the empirical proportion of respondents targeted. The indirect and total effect estimates used robust standard errors clustered on village, and standard errors for the direct effect estimates were calculated using the delta method.

We performed additional sensitivity analysis to test the robustness of our primary findings. Results for our additional analyses are reported in the supplemental tables below. Table S1 reports direct and total effect estimates for all primary and secondary outcomes, and also details the percentage of respondents who reported the desired outcome at baseline. Table S2 reports the direct and total effect estimates for a sub-sample of respondents that was limited to mother of children born after the intervention period, and effects are reported only for the primary outcomes. Table S3 reports the direct and total effect estimates for all primary and secondary outcomes for a sub-sample of respondents that was limited to those who did not move between villages, and therefore treatment arms, during the

For peer review only - http://bmjopen.bmj.com/site/about/guidelines.xhtml

study period. Table S4 reports the direct and total effect estimates for all primary and secondary outcomes, adjusted for baseline demographic characteristics (including age, gender, marital status, indigenous status, and household wealth index). Table S5 reports the direct and total effect estimates for all primary and secondary outcomes, adjusted for outcomes at baseline. Table S6 reports the direct and total effect estimates, adjusted for baseline demographic characteristics and baseline outcomes. Table S7 reports the direct and total effect estimates for a sub-sample of respondents that was limited to those who did not move between villages, adjusted for baseline demographic characteristics and baseline outcomes. Table S8 reports the direct and total effect estimates using relevant intervention module receipt as indicators of treatment, adjusted for baseline demographic characteristics.

Additional analysis

When analysis of practices was limited to the responses of mothers, the probability of having the newborn's health checked by a professional in a health facility within three days and of not using a fajero on the umbilical cord were even greater among the targeted, and, additionally, a significant effect on the likelihood that mothers reported that the father was involved with bathing their child emerged (Table S2, appendix). We performed additional sensitivity analyses by excluding respondents that moved between treatment arms during the study, adjusting for the respondent's baseline responses for each outcome, and adjusting for demographic factors including gender, age, education, marital status, indigenous status, and household wealth index (Table S7, appendix). When all of the aforementioned covariates were included, we noted that there were no significant total effects for seeking a professional check-up for a newborn within three days of birth or for immediate breastfeeding, while the total effect estimate for not using a fajero increased. In these analyses, additional significant total effects also emerged for mothers seeking medical care after experiencing a postnatal danger sign and parents providing their children with proper treatment (defined as providing zinc, antibiotics, additional food, or oral rehydration therapy) after their child experienced past-month diarrheal illness. The variation in effects with individual covariate adjustment suggests that the impact of the intervention is heterogenous and that, while we are able to report pooled effects across the study population, it is expected that some individuals will be more likely to respond to the interventions than others.

For peer review only - http://bmjopen.bmj.com/site/about/guidelines.xhtml

Figures

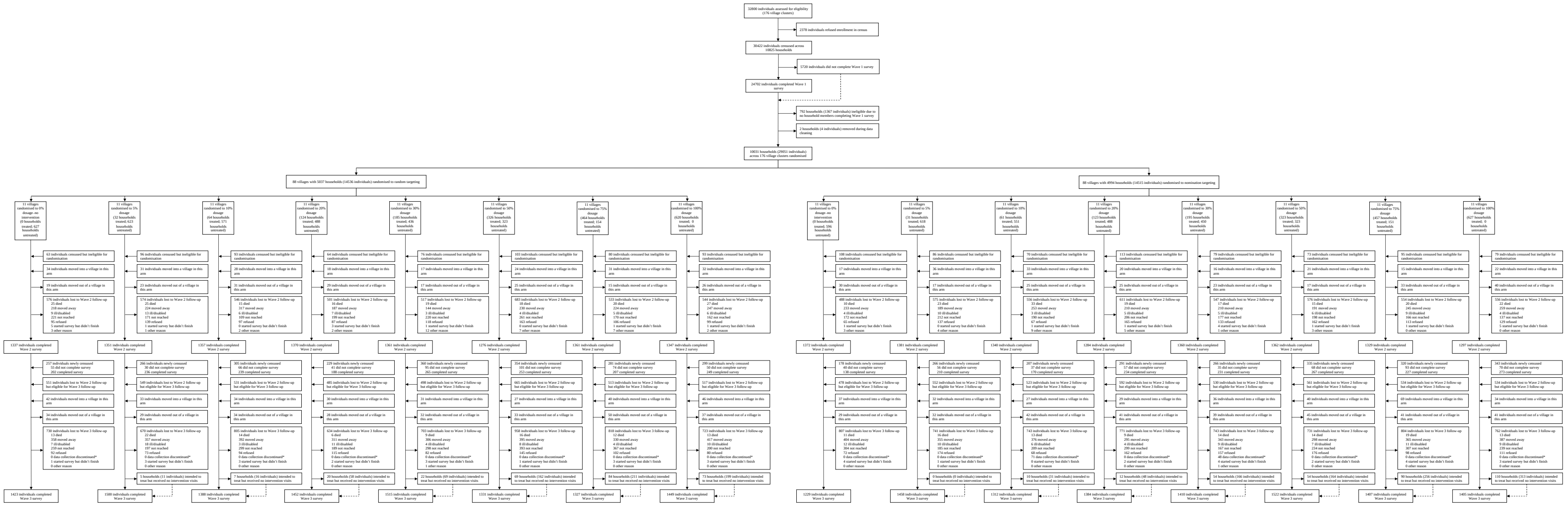

Figure S1. Trial profile for parent study

For peer review only - http://bmjopen.bmj.com/site/about/guidelines.xhtml

Tables

Table S1. Pooled direct, indirect, and total effect estimates of intervention on maternal, child, and neonatal outcomes

	Outcome type	Respondent denominator	Pooled direct effect estimate		Pooled indirect effect estimate		Pooled total effect estimate		N	Outcome at baseline survey (%)
			Effect size (95% CI)	p value	Effect size (95% CI)	p value	Effect size (95% CI)	p value		
**Prenatal care**										
Currently taking folic acid tablets (daily in past 7 days)	Practice	All women 15+ with a complete survey	-0·0049 (-0·034 to 0·024)	0·74	0·0053 (-0·036 to 0·047)	0·80	0·0004 (-0·043 to 0·044)	0·98	6311	32
Made a birth plan in preparation for birth	Practice	All respondents with a complete survey and a child born since 2018-09-01 (after the end of the intervention)	0·020 (-0·071 to 0·11)	0·67	0·054 (-0·020 to 0·13)	0·15	0·074 (-0·015 to 0·16)	0·10	619	61
Saved money in preparation for birth	Practice	All respondents with a complete survey and a child born since 2018-09-01 (after the end of the intervention)	0·046 (-0·061 to 0·15)	0·40	-0·065 (-0·15 to 0·023)	0·14	-0·020 (-0·14 to 0·098)	0·74	620	63
Sold animal in preparation for birth	Practice	All respondents with a complete survey and a child born since 2018-09-01 (after the end of the intervention)	-0·011 (-0·079 to 0·058)	0·76	-0·0044 (-0·055 to 0·046)	0·86	-0·015 (-0·080 to 0·049)	0·65	619	NA
Sought prenatal care within 12 weeks	Practice	All respondents with a complete survey and a child born since 2018-09-01 (after the end of the intervention)	-0·042 (-0·17 to 0·090)	0·54	-0·011 (-0·12 to 0·095)	0·84	-0·053 (-0·19 to 0·089)	0·46	597	73
Identifies that women should take folic acid before pregnancy	Knowledge and attitudes	All respondents with a complete survey	0·033 (0·0010 to 0·064)	0·044	0·010 (-0·024 to 0·044)	0·55	0·043 (0·0008 to 0·085)	0·046	10262	73
Identifies that women should seek prenatal care first 12 weeks of pregnancy	Knowledge and attitudes	All respondents with a complete survey	0·019 (-0·0061 to 0·044)	0·14	0·0038 (-0·018 to 0·026)	0·73	0·023 (-0·0046 to 0·050)	0·10	10261	87

For peer review only

For peer review only - http://bmjopen.bmj.com/site/about/guidelines.xhtml

Identifies accompanying woman to prenatal care visits as method of support during pregnancy	Knowledge and attitudes	All respondents with a complete survey	0·0051 (-0·025 to 0·036)	0·74	-0·011 (-0·060 to 0·038)	0·66	-0·0058 (-0·059 to 0·048)	0·83	10257	NA
Identifies ensuring that woman eats well as method of support during pregnancy	Knowledge and attitudes	All respondents with a complete survey	-0·0072 (-0·040 to 0·025)	0·66	-0·012 (-0·057 to 0·034)	0·61	-0·019 (-0·071 to 0·032)	0·47	10257	NA
Identifies ensuring that woman rests 1 hour per day well as method of support during pregnancy	Knowledge and attitudes	All respondents with a complete survey	0·0046 (-0·018 to 0·028)	0·69	-0·0012 (-0·034 to 0·031)	0·94	0·0034 (-0·028 to 0·035)	0·83	10257	NA
Identifies avoiding violence as method of support during pregnancy	Knowledge and attitudes	All respondents with a complete survey	-0·014 (-0·032 to 0·0037)	0·12	-0·018 (-0·036 to 0·0003)	0·054	-0·032 (-0·053 to -0·011)	0·0023	10257	NA
Identifies encouraging women to take vitamins as method of support during pregnancy	Knowledge and attitudes	All respondents with a complete survey	-0·0046 (-0·027 to 0·018)	0·68	-0·019 (-0·053 to 0·016)	0·29	-0·023 (-0·060 to 0·013)	0·21	10257	NA
Identifies helping woman with house work/child care as method of support during pregnancy	Knowledge and attitudes	All respondents with a complete survey	0·019 (-0·012 to 0·050)	0·24	0·053 (-0·0039 to 0·11)	0·068	0·072 (0·010 to 0·13)	0·022	10257	NA
Identifies saving animals to sell as method of preparing for birth expenses	Knowledge and attitudes	All respondents with a complete survey	0·017 (-0·0027 to 0·036)	0·093	0·016 (-0·0098 to 0·043)	0·22	0·033 (0·0025 to 0·063)	0·034	10261	NA
Identifies knowing cost of trip to maternal clinic as method of preparing for birth expenses	Knowledge and attitudes	All respondents with a complete survey	-0·0049 (-0·023 to 0·014)	0·61	-0·0019 (-0·031 to 0·027)	0·90	-0·0068 (-0·038 to 0·024)	0·67	10261	NA
Identifies having a savings plan as method of preparing for birth expenses	Knowledge and attitudes	All respondents with a complete survey	0·030 (0·013 to 0·047)	0·0004	0·0013 (-0·025 to 0·027)	0·92	0·031 (0·0022 to 0·060)	0·035	10261	NA
Correctly answered prenatal care knowledge riddle	Intervention knowledge	All respondents with a complete survey	0·044 (0·015 to 0·073)	0·0030	0·032 (0 to 0·065)	0·050	0·076 (0·040 to 0·11)	<0·0001	10261	NA
**Facility based birth**										
Gave birth in health facility	Practice	All respondents with a complete survey and a child born since 2018-09-01 (after the end of the intervention)	0·018 (-0·075 to 0·11)	0·70	-0·031 (-0·17 to 0·11)	0·67	-0·013 (-0·15 to 0·13)	0·86	620	72
Believes health facility is best place to give birth	Knowledge and attitudes	All respondents with a complete survey	0·021 (0·0036 to 0·039)	0·018	-0·013 (-0·050 to 0·024)	0·48	0·0080 (-0·028 to 0·044)	0·66	10262	90
**Pregnancy danger signs**										

For peer review only - http://bmjopen.bmj.com/site/about/guidelines.xhtml

Woman sought medical care for pregnancy danger sign	Practice	All respondents with a complete survey and a child born since 2018-09-01 (after the end of the intervention) who experienced a prenatal care danger sign	-0·026 (-0·23 to 0·18)	0·80	-0·081 (-0·24 to 0·076)	0·31	-0·11 (-0·33 to 0·12)	0·35	235	64
Identified bleeding as pregnancy d.s.	Knowledge and attitudes	All respondents with a complete survey	0·018 (-0·015 to 0·052)	0·28	0·021 (-0·017 to 0·058)	0·28	0·039 (-0·0039 to 0·082)	0·075	10260	41
Identified seizure as pregnancy d.s.	Knowledge and attitudes	All respondents with a complete survey	0·0048 (-0·0047 to 0·014)	0·32	-0·0020 (-0·0094 to 0·0054)	0·60	0·0028 (-0·0063 to 0·012)	0·54	10260	6
Identified headache as pregnancy d.s.	Knowledge and attitudes	All respondents with a complete survey	0·027 (-0·012 to 0·066)	0·18	0·0079 (-0·031 to 0·046)	0·69	0·035 (-0·017 to 0·086)	0·18	10260	39
Identified ringing in ears as pregnancy d.s.	Knowledge and attitudes	All respondents with a complete survey	0·0040 (-0·0053 to 0·013)	0·40	0·0010 (-0·0055 to 0·0074)	0·77	0·0050 (-0·0052 to 0·015)	0·34	10260	4
Identified dizziness as pregnancy d.s.	Knowledge and attitudes	All respondents with a complete survey	-0·014 (-0·048 to 0·020)	0·42	0·0031 (-0·042 to 0·048)	0·89	-0·011 (-0·060 to 0·038)	0·66	10260	37
Identified difficulty urinating as pregnancy d.s.	Knowledge and attitudes	All respondents with a complete survey	0·0024 (-0·0033 to 0·0081)	0·41	-0·0025 (-0·0095 to 0·0046)	0·49	-0·0001 (-0·0078 to 0·0077)	0·99	10260	2
Identified reduced or absent fetal movement as pregnancy d.s.	Knowledge and attitudes	All respondents with a complete survey	0·0087 (-0·0087 to 0·026)	0·33	0·0096 (-0·0044 to 0·024)	0·18	0·018 (-0·0048 to 0·042)	0·12	10260	6
Identified water breaking as pregnancy d.s.	Knowledge and attitudes	All respondents with a complete survey	-0·0025 (-0·0088 to 0·0037)	0·43	0·0024 (-0·0037 to 0·0084)	0·45	-0·0002 (-0·0074 to 0·0070)	0·96	10260	2
Identified fever as pregnancy d.s.	Knowledge and attitudes	All respondents with a complete survey	0·012 (-0·012 to 0·036)	0·34	0·027 (-0·0056 to 0·059)	0·11	0·039 (0·0013 to 0·076)	0·042	10260	14
Identified swelling of face/hands/feet as pregnancy d.s.	Knowledge and attitudes	All respondents with a complete survey	0·017 (-0·0095 to 0·044)	0·21	0·017 (-0·0061 to 0·040)	0·15	0·034 (0·0032 to 0·065)	0·030	10260	10
**Postnatal care for mother**										
Mother had health checked by professional within 3 days of birth	Practice	All respondents with a complete survey and a child born since 2018-09-01 (after the end of the intervention)	0·051 (-0·060 to 0·16)	0·37	0·088 (-0·055 to 0·23)	0·23	0·14 (-0·024 to 0·30)	0·094	605	14
Mother had health checked by professional within 7 days of birth	Practice	All respondents with a complete survey and a child born since 2018-09-01 (after the	0·00020 (-0·13 to 0·13)	1·00	0·12 (-0·0002 to 0·25)	0·050	0·12 (-0·037 to 0·28)	0·13	605	27

For peer review only

		end of the intervention)								
Mother sought medical care for postnatal danger sign	Practice	All respondents with a complete survey and a child born since 2018-09-01 (after the end of the intervention)	0·063 (-0·14 to 0·26)	0·54	0·16 (-0·020 to 0·33)	0·082	0·22 (-0·0038 to 0·44)	0·054	194	48
Identifies that mother should receive postnatal medical check-up within 3 days of birth	Knowledge and attitudes	All respondents with a complete survey	0·088 (0·058 to 0·12)	<0·0001	-0·0007 (-0·037 to 0·036)	0·97	0·088 (0·043 to 0·13)	0·0001	10261	9
Identifies that mother should receive postnatal medical check-up within 7 days of birth	Knowledge and attitudes	All respondents with a complete survey	0·061 (0·026 to 0·097)	0·0008	-0·0025 (-0·046 to 0·041)	0·91	0·059 (0·0086 to 0·11)	0·022	10261	24
Identified heavy vaginal bleeding as postnatal d.s.	Knowledge and attitudes	All respondents with a complete survey	0·047 (0·011 to 0·083)	0·010	0·0011 (-0·032 to 0·034)	0·95	0·048 (0·0074 to 0·088)	0·020	10260	43
Identified fever as postnatal d.s.	Knowledge and attitudes	All respondents with a complete survey	0·025 (-0·010 to 0·060)	0·17	0·026 (-0·012 to 0·063)	0·18	0·051 (0·0024 to 0·099)	0·040	10260	38
Identified vaginal discharge as postnatal d.s.	Knowledge and attitudes	All respondents with a complete survey	0·0083 (-0·0022 to 0·019)	0·12	-0·0055 (-0·017 to 0·0060)	0·35	0·0029 (-0·010 to 0·016)	0·67	10260	7
Identified headache as postnatal d.s.	Knowledge and attitudes	All respondents with a complete survey	0·019 (-0·0070 to 0·044)	0·15	0·0073 (-0·027 to 0·041)	0·67	0·026 (-0·013 to 0·065)	0·20	10260	22
Identified convulsions or fits as postnatal d.s.	Knowledge and attitudes	All respondents with a complete survey	0·0021 (-0·0021 to 0·0062)	0·33	-0·0019 (-0·0066 to 0·0028)	0·44	0·0002 (-0·0051 to 0·0056)	0·94	10260	1
**Postnatal care for newborn**										
Newborn had health checked by professional within 3 days of birth	Practice	All respondents with a complete survey and a child born since 2018-09-01 (after the end of the intervention)	0·087 (-0·026 to 0·20)	0·13	0·077 (-0·033 to 0·19)	0·17	0·16 (0·031 to 0·30)	0·016	610	15
Newborn had health checked by professional within 7 days of birth	Practice	All respondents with a complete survey and a child born since 2018-09-01 (after the end of the intervention)	0·079 (-0·050 to 0·21)	0·23	0·049 (-0·047 to 0·14)	0·32	0·13 (-0·014 to 0·27)	0·077	610	29
Newborn experienced health problem in first month, care was sought	Practice	All respondents with a complete survey and a child born since 2018-09-01 (after the	-0·081 (-0·34 to 0·18)	0·54	0·11 (-0·070 to 0·29)	0·23	0·030 (-0·22 to 0·28)	0·81	177	74

For peer review only - http://bmjopen.bmj.com/site/about/guidelines.xhtml

		end of the intervention)								
Identified correct ways to provide newborn care[a]	Knowledge and attitudes	All respondents with a complete survey	0·015 (0·0044 to 0·026)	0·0055	0·012 (-0·0023 to 0·026)	0·10	0·027 (0·010 to 0·043)	0·0014	10261	5
Identified fever as newborn d.s.	Knowledge and attitudes	All respondents with a complete survey	-0·023 (-0·062 to 0·016)	0·25	0·028 (-0·011 to 0·067)	0·15	0·0055 (-0·044 to 0·055)	0·83	10260	69
Identified diarrhea as newborn d.s.	Knowledge and attitudes	All respondents with a complete survey	0·0096 (-0·019 to 0·038)	0·51	-0·0002 (-0·038 to 0·037)	0·99	0·0093 (-0·032 to 0·050)	0·65	10260	49
Identified difficulty breathing as newborn d.s.	Knowledge and attitudes	All respondents with a complete survey	0·0099 (-0·010 to 0·030)	0·34	0·0033 (-0·028 to 0·035)	0·84	0·013 (-0·024 to 0·050)	0·48	10260	36
Identified vomiting as newborn d.s.	Knowledge and attitudes	All respondents with a complete survey	0·0018 (-0·018 to 0·021)	0·86	0·0084 (-0·017 to 0·034)	0·52	0·010 (-0·017 to 0·038)	0·47	10260	27
Identified poor appetite as newborn d.s.	Knowledge and attitudes	All respondents with a complete survey	-0·0001 (-0·011 to 0·011)	0·99	-0·0064 (-0·014 to 0·0012)	0·097	-0·0065 (-0·016 to 0·0032)	0·19	10260	6
Identified redness/bleeding around cord as newborn d.s.	Knowledge and attitudes	All respondents with a complete survey	0·0099 (-0·0027 to 0·023)	0·13	0·0047 (-0·014 to 0·024)	0·63	0·015 (-0·0056 to 0·035)	0·16	10260	5
Identified pus in cord as newborn d.s.	Knowledge and attitudes	All respondents with a complete survey	0·013 (0·0016 to 0·024)	0·025	0·0006 (-0·012 to 0·013)	0·93	0·013 (-0·0021 to 0·028)	0·092	10260	5
Identified cold skin as newborn d.s.	Knowledge and attitudes	All respondents with a complete survey	0·0042 (-0·0048 to 0·013)	0·36	-0·0025 (-0·0086 to 0·0037)	0·43	0·0017 (-0·0067 to 0·010)	0·69	10260	2
Identified cough as newborn d.s.	Knowledge and attitudes	All respondents with a complete survey	-0·034 (-0·063 to -0·0054)	0·020	0·015 (-0·026 to 0·057)	0·47	-0·019 (-0·065 to 0·027)	0·42	10260	2
Identified cold as newborn d.s.	Knowledge and attitudes	All respondents with a complete survey	-0·022 (-0·052 to 0·0085)	0·16	0·0028 (-0·045 to 0·050)	0·91	-0·019 (-0·074 to 0·036)	0·50	10260	1
Identified problems latching as newborn d.s.	Knowledge and attitudes	All respondents with a complete survey	0·0009 (-0·0080 to 0·0098)	0·84	-0·0051 (-0·015 to 0·0053)	0·34	-0·0041 (-0·017 to 0·0082)	0·51	10260	0
Identified pneumonia as newborn d.s.	Knowledge and attitudes	All respondents with a complete survey	0·0027 (-0·025 to 0·031)	0·85	-0·0037 (-0·037 to 0·029)	0·82	-0·0010 (-0·037 to 0·035)	0·95	10260	NA
Did not use harmful substances to treat cord stump	Practice	All respondents with a complete survey and a child born since 2018-09-01 (after the end of the intervention)	0·070 (-0·052 to 0·19)	0·26	0·022 (-0·098 to 0·14)	0·72	0·092 (-0·042 to 0·23)	0·18	598	83
Did not wrap fajero around newborn in first 7 days after birth	Practice	All respondents with a complete survey and a child born since 2018-09-01 (after the end of the intervention)	0·15 (0·050 to 0·26)	0·0038	0·043 (-0·100 to 0·19)	0·56	0·20 (0·042 to 0·35)	0·013	616	6

Identified proper cord care methods[b]	Knowledge and attitudes	All respondents with a complete survey	0·089 (0·056 to 0·12)	<0·0001	0·0015 (-0·044 to 0·047)	0·95	0·091 (0·040 to 0·14)	0·0005	10260	47
Correctly answered proper cord care riddle	Intervention knowledge	All respondents with a complete survey	0·17 (0·13 to 0·21)	<0·0001	0·019 (-0·027 to 0·066)	0·42	0·19 (0·14 to 0·24)	<0·0001	10262	NA
Kept newborn wrapped first 7 days after birth	Practice	All respondents with a complete survey and a child born since 2018-09-01 (after the end of the intervention)	-0·044 (-0·095 to 0·0080)	0·098	0·013 (-0·036 to 0·063)	0·59	-0·030 (-0·086 to 0·026)	0·29	619	99
Held newborn skin-to-skin during first month after birth	Practice	All respondents with a complete survey and a child born since 2018-09-01 (after the end of the intervention)	0·095 (-0·041 to 0·23)	0·17	0·032 (-0·080 to 0·14)	0·57	0·13 (-0·014 to 0·27)	0·077	617	100
**Breastfeeding**										
Exclusively breastfed child first 6 months	Practice	All respondents with a complete survey and a child born since 2018-09-01 (after the end of the intervention)	0·053 (-0·085 to 0·19)	0·45	-0·032 (-0·16 to 0·091)	0·61	0·021 (-0·14 to 0·18)	0·80	619	59
Exclusively breastfed child first 6 months without giving chupón	Practice	All respondents with a complete survey and a child born since 2018-09-01 (after the end of the intervention)	0·086 (-0·046 to 0·22)	0·20	0·012 (-0·10 to 0·12)	0·84	0·097 (-0·048 to 0·24)	0·19	619	34
Breastfed immediately after birth	Practice	All respondents with a complete survey and a child born since 2018-09-01 (after the end of the intervention) who reported a natural birth	0·13 (0·047 to 0·21)	0·0023	-0·042 (-0·12 to 0·035)	0·29	0·089 (0·0028 to 0·17)	0·043	493	86
Identifies that newborn should be breastfed immediately after birth	Knowledge and attitudes	All respondents with a complete survey	0·0045 (-0·019 to 0·028)	0·70	0·010 (-0·016 to 0·037)	0·45	0·015 (-0·018 to 0·048)	0·38	10261	82
Identifies that newborns should only be given breast milk during first 6 months	Knowledge and attitudes	All respondents with a complete survey	0·042 (0·017 to 0·067)	0·0008	-0·015 (-0·041 to 0·011)	0·25	0·027 (-0·0048 to 0·059)	0·096	10261	80

For peer review only - http://bmjopen.bmj.com/site/about/guidelines.xhtml

For peer review only

Believes newborns should not be given chupón during first 6 months	Knowledge and attitudes	All respondents with a complete survey	0·15 (0·11 to 0·19)	<0·0001	-0·011 (-0·060 to 0·038)	0·67	0·14 (0·084 to 0·20)	<0·0001	10261	23
**Paternal involvement**										
Father accompanied mother to clinic for prenatal care visit at least once	Practice	All respondents with a complete survey and a child born since 2018-09-01 (after the end of the intervention) who received prenatal care	0·018 (-0·12 to 0·15)	0·79	-0·064 (-0·21 to 0·087)	0·41	-0·045 (-0·22 to 0·13)	0·62	595	52
Father accompanied mother to seek medical care for pregnancy danger sign	Practice	All respondents with a complete survey and a child born since 2018-09-01 (after the end of the intervention) who experienced a prenatal care danger sign and sought care	0·010 (-0·15 to 0·17)	0·90	-0·0013 (-0·18 to 0·18)	0·99	0·0091 (-0·21 to 0·23)	0·94	149	65
Father waited at birthplace during labor	Practice	All respondents with a complete survey and a child born since 2018-09-01 (after the end of the intervention)	-0·031 (-0·14 to 0·075)	0·56	0·0083 (-0·12 to 0·13)	0·90	-0·023 (-0·16 to 0·11)	0·74	620	78
Father accompanied mother to seek medical care for postnatal danger sign	Practice	All respondents with a complete survey and a child born since 2018-09-01 (after the end of the intervention) and sought care for postpartum problem	0·18 (-0·032 to 0·39)	0·096	-0·037 (-0·32 to 0·24)	0·80	0·14 (-0·17 to 0·45)	0·37	97	67
Father sought medical care for newborn for postnatal danger sign	Practice	All respondents with a complete survey and a child born since 2018-09-01 (after the end of the intervention) and sought care for a postnatal problem	0·0059 (-0·33 to 0·34)	0·97	-0·099 (-0·37 to 0·17)	0·48	-0·093 (-0·44 to 0·25)	0·60	116	73
Father held child	Practice	All respondents with a complete survey and a child born since 2018-	-0·046 (-0·11 to 0·022)	0·19	0·078 (0·013 to 0·14)	0·020	0·032 (-0·040 to 0·10)	0·38	614	97

For peer review only - http://bmjopen.bmj.com/site/about/guidelines.xhtml

		09-01 (after the end of the intervention)								
Father played with child	Practice	All respondents with a complete survey and a child born since 2018-09-01 (after the end of the intervention)	-0·091 (-0·19 to 0·0099)	0·077	0·076 (-0·030 to 0·18)	0·16	-0·015 (-0·13 to 0·10)	0·80	614	90
Father took child to clinic when sick	Practice	All respondents with a complete survey and a child born since 2018-09-01 (after the end of the intervention)	-0·053 (-0·19 to 0·080)	0·44	0·084 (-0·011 to 0·18)	0·082	0·031 (-0·11 to 0·17)	0·66	614	71
Believes father should accompany mother to prenatal care visits	Knowledge and attitudes	All respondents with a complete survey	0·041 (0·022 to 0·060)	<0·0001	0·0024 (-0·022 to 0·027)	0·85	0·043 (0·019 to 0·068)	0·0005	10220	93
Believes father should wait at birth location while mother gives birth	Knowledge and attitudes	All respondents with a complete survey	0·0044 (-0·0078 to 0·017)	0·48	0·0070 (-0·0079 to 0·022)	0·36	0·011 (-0·0062 to 0·029)	0·20	10225	94
Believes father should care for children when sick	Knowledge and attitudes	All respondents with a complete survey	-0·0012 (-0·0069 to 0·0044)	0·67	-0·0015 (-0·0074 to 0·0043)	0·60	-0·0028 (-0·010 to 0·0047)	0·47	10250	99
**Diarrhea management**										
Did not report diarrhea in past 4 weeks	Practice	All respondents with a complete survey	-0·0010 (-0·014 to 0·012)	0·87	-0·0086 (-0·019 to 0·0021)	0·12	-0·0096 (-0·025 to 0·0060)	0·23	10263	97
Used ORT to treat diarrhea in past 4 weeks	Practice	All respondents with a complete survey who had diarrhea in past 4 weeks	-0·14 (-0·30 to 0·022)	0·091	-0·044 (-0·20 to 0·12)	0·59	-0·19 (-0·38 to 0·011)	0·064	266	52
Used appropriate treatment for diarrhea in past 4 weeks[c]	Practice	All respondents with a complete survey who had diarrhea in past 4 weeks	-0·015 (-0·16 to 0·13)	0·84	0·012 (-0·12 to 0·15)	0·86	-0·0027 (-0·16 to 0·16)	0·97	268	28
Child did not experience diarrhea past 4 weeks	Practice	All respondents with a complete survey and a new child <= 5 years old reported at wave 3 who had diarrhea in past 4 weeks	0·0042 (-0·025 to 0·034)	0·78	-0·011 (-0·044 to 0·023)	0·53	-0·0064 (-0·043 to 0·030)	0·73	3333	84
Child experienced diarrhea past 4 weeks, was given appropriate amount of liquids	Practice	All respondents with a complete survey and a new child <= 5 years old reported at	-0·100 (-0·25 to 0·046)	0·18	-0·031 (-0·20 to 0·14)	0·73	-0·13 (-0·31 to 0·050)	0·16	350	50

For peer review only - http://bmjopen.bmj.com/site/about/guidelines.xhtml

		wave 3 who had diarrhea in past 4 weeks								
Child experienced diarrhea past 4 weeks, was given appropriate amount of food	Practice	All respondents with a complete survey and a new child <= 5 years old reported at wave 3 who had diarrhea in past 4 weeks	-0·088 (-0·20 to 0·028)	0·14	-0·024 (-0·15 to 0·099)	0·70	-0·11 (-0·25 to 0·024)	0·11	350	35
Child experienced diarrhea past 4 weeks, was given ORT	Practice	All respondents with a complete survey and a new child <= 5 years old reported at wave 3 who had diarrhea in past 4 weeks	0·082 (-0·069 to 0·23)	0·29	0·056 (-0·089 to 0·20)	0·45	0·14 (-0·032 to 0·31)	0·11	347	60
Child experienced diarrhea past 4 weeks, was given appropriate treatment	Practice	All respondents with a complete survey and a new child <= 5 years old reported at wave 3 who had diarrhea in past 4 weeks	0·027 (-0·10 to 0·15)	0·68	0·076 (-0·056 to 0·21)	0·26	0·10 (-0·059 to 0·27)	0·21	348	31
Child experienced diarrhea past 4 weeks, was given zinc 10-14 days	Practice	All respondents with a complete survey and a new child <= 5 years old reported at wave 3 who had diarrhea in past 4 weeks	0·0080 (-0·030 to 0·046)	0·68	0·15 (0·0026 to 0·29)	0·046	0·16 (-0·014 to 0·33)	0·072	311	1
Identified appropriate diarrhea treatment methods[d]	Knowledge and attitudes	All respondents with a complete survey	0·083 (0·045 to 0·12)	<0·0001	0·014 (-0·030 to 0·058)	0·54	0·097 (0·042 to 0·15)	0·0005	10260	41
Identified zinc 10-14 days as diarrhea treatment	Knowledge and attitudes	All respondents with a complete survey	0·0093 (-0·0073 to 0·026)	0·27	0·020 (-0·0005 to 0·040)	0·056	0·029 (0·0079 to 0·050)	0·0072	10261	4
Identified zinc supplement as way to prevent diarrhea	Knowledge and attitudes	All respondents with a complete survey	0·063 (0·035 to 0·090)	<0·0001	-0·0004 (-0·066 to 0·065)	0·99	0·062 (-0·0063 to 0·13)	0·075	10261	45
Identified breastfeeding as way to prevent diarrhea	Knowledge and attitudes	All respondents with a complete survey	0·031 (0·0028 to 0·060)	0·032	0·0070 (-0·058 to 0·072)	0·83	0·038 (-0·028 to 0·10)	0·26	10261	26
Correctly answered diarrhea treatment with zinc riddle	Intervention knowledge	All respondents with a complete survey	0·20 (0·16 to 0·24)	<0·0001	0·019 (-0·035 to 0·074)	0·49	0·22 (0·16 to 0·28)	<0·0001	10261	NA
**Respiratory illness**										

For peer review only - http://bmjopen.bmj.com/site/about/guidelines.xhtml

Did not report respiratory illness (coughing) for 2 weeks	Practice	All respondents with a complete survey	0·0005 (-0·022 to 0·023)	0·97	-0·017 (-0·045 to 0·011)	0·23	-0·017 (-0·050 to 0·017)	0·33	10263	79	
Child did not have cough past 4 weeks	Practice	All respondents with a complete survey	0·0076 (-0·037 to 0·052)	0·74	-0·070 (-0·11 to -0·029)	0·0009	-0·063 (-0·12 to -0·0051)	0·033	3334	67	
Child had cough past 4 weeks, care was sought	Practice	All respondents with a complete survey and a new child <= 5 years old reported at wave 3 who had an illness with a cough in past 4 weeks	-0·041 (-0·14 to 0·055)	0·40	0·070 (-0·033 to 0·17)	0·18	0·029 (-0·092 to 0·15)	0·64	700	4	
Identified vaccination as way to prevent respiratory illness	Knowledge and attitudes	All respondents with a complete survey	0·021 (-0·00010 to 0·042)	0·051	-0·042 (-0·080 to -0·0035)	0·033	-0·021 (-0·059 to 0·017)	0·28	10261	51	
Identified breastfeeding as way to prevent respiratory illness	Knowledge and attitudes	All respondents with a complete survey	0·015 (-0·013 to 0·042)	0·29	-0·0010 (-0·052 to 0·050)	0·97	0·014 (-0·040 to 0·068)	0·62	10261	34	
Identified fever as d.s. for children with respiratory illness	Knowledge and attitudes	All respondents with a complete survey	0·0067 (-0·0067 to 0·020)	0·33	-0·0023 (-0·032 to 0·027)	0·88	0·0044 (-0·028 to 0·037)	0·79	10261	82	
Identified difficulty breathing as d.s. for children with respiratory illness	Knowledge and attitudes	All respondents with a complete survey	0·011 (0·0032 to 0·019)	0·0057	-0·0064 (-0·023 to 0·011)	0·46	0·0046 (-0·014 to 0·023)	0·62	10261	89	
Identified rapid breathing as d.s. for children with respiratory illness	Knowledge and attitudes	All respondents with a complete survey	0·0073 (0·0012 to 0·013)	0·019	-0·0025 (-0·014 to 0·0087)	0·67	0·0048 (-0·0070 to 0·017)	0·42	10261	85	
**Reproductive health**											
Reported ever using birth control to delay or avoid pregnancy	Practice	All respondents with a complete survey	0·015 (-0·021 to 0·052)	0·41	-0·033 (-0·084 to 0·019)	0·21	-0·018 (-0·073 to 0·038)	0·53	10257	33	
Reported currently using birth control	Practice	All respondents with a complete survey who are not pregnant and have ever used birth control	0·014 (-0·039 to 0·066)	0·61	0·035 (-0·025 to 0·095)	0·26	0·048 (-0·023 to 0·12)	0·19	3793	55	
Believes woman should be at least 18 years of age to have her first child	Knowledge and attitudes	All respondents with a complete survey	0·0054 (-0·0010 to 0·012)	0·096	0·0056 (-0·0022 to 0·014)	0·16	0·011 (0·0021 to 0·020)	0·015	9733	98	
**Gender/reproductive norms**											
Birth location chosen either jointly or by woman	Practice	All respondents with a complete survey and a child born since 2018-09-01 (after the	0·0093 (-0·061 to 0·080)	0·80	0·039 (-0·045 to 0·12)	0·36	0·048 (-0·054 to 0·15)	0·36	620	93	

For peer review only - http://bmjopen.bmj.com/site/about/guidelines.xhtml

		end of the intervention)								

Results based on final sample of N=10263 (household targeted n=3238, household not targeted n=7025). d.s.=Danger sign, NA=Not applicable, ORT=Oral rehydration therapy. Proportions with NA could not be assessed because the outcome was not part of the baseline instrument. Standard errors are clustered at the village level. Robust 95% CIs in parentheses.

[a] 'Identified correct ways to provide newborn care' outcome was coded as correct if the respondent identified holding baby skin to skin, getting their health checked by a professional, immediately putting them to the breast, or making sure their diaper is clean and dry, and did not identify giving purgante, wrapping a fajero, giving a chupon, or bathing them in water right away.
[b] 'Identified proper cord care' outcome was coded as correct if respondent identified applying alcohol, applying iodine, keeping cord clean/dry, or applying peroxide, and did not identify applying oil, applying coffee, applying quina water, wrapping a fajero, applying powder, burning, applying ashes, applying a clamp, or applying thiomersal.
[c] 'Used appropriate treatment for diarrhea in past 4 weeks' outcome was coded as correct if respondent did any of the following: antibiotic, zinc, give extra food, give extra liquid, and did not do any of the following: anti-diarrhea medication, home remedy, laxative, deworming medication, chupon, massage, stop eating foods, stop taking liquids.
[d] 'Identified appropriate diarrhea treatment methods' outcome was coded as correct if respondent identified any of the following: antibiotic, zinc, ORS or Litrosol, give extra food, give extra liquid, get medical attention, and did not identify any of the following: anti-diarrhea medication, laxative, deworming medication, home remedy, chupon, massage, stop giving foods, stop giving liquids, or do nothing.

For peer review only

For peer review only - http://bmjopen.bmj.com/site/about/guidelines.xhtml

Table S2. Pooled total effect estimates of intervention on maternal, child, and neonatal outcomes, mothers responses for practice outcomes only

	Pooled total effect estimate		N
	Effect size (95% CI)	p value	
**Prenatal care**			
Currently taking folic acid tablets (daily in past 7 days)	0·0004 (-0·043 to 0·044)	0·98	6311
Made a birth plan in preparation for birth	0·031 (-0·11 to 0·17)	0·66	412
Saved money in preparation for birth	-0·039 (-0·17 to 0·091)	0·56	412
Sold animal in preparation for birth	-0·014 (-0·097 to 0·070)	0·75	412
Sought prenatal care within 12 weeks	-0·031 (-0·19 to 0·12)	0·69	407
**Facility based birth**			
Gave birth in health facility	-0·049 (-0·19 to 0·094)	0·50	412
**Pregnancy danger signs**			
Woman sought medical care for pregnancy danger sign	-0·12 (-0·41 to 0·16)	0·39	152
**Postnatal care for mother**			
Mother had health checked by professional within 3 days of birth	0·14 (-0·038 to 0·32)	0·12	411
Mother had health checked by professional within 7 days of birth	0·11 (-0·058 to 0·27)	0·20	411
Mother sought medical care for postnatal danger sign	0·22 (-0·014 to 0·46)	0·066	131
**Postnatal care for newborn**			
Newborn had health checked by professional within 3 days of birth	0·18 (0·015 to 0·34)	0·032	411
Newborn had health checked by professional within 7 days of birth	0·11 (-0·049 to 0·27)	0·17	411
Newborn experienced health problem in first month, care was sought	-0·0022 (-0·29 to 0·29)	0·99	129
Did not use harmful substances to treat cord stump	0·054 (-0·10 to 0·21)	0·50	410
Did not wrap fajero around newborn in first 7 days after birth	0·24 (0·069 to 0·41)	0·0060	411
Kept newborn wrapped first 7 days after birth	-0·0026 (-0·059 to 0·054)	0·93	412
Held newborn skin-to-skin during first month after birth	0·15 (-0·013 to 0·31)	0·073	411
**Breastfeeding**			
Exclusively breastfed child first 6 months	0·061 (-0·10 to 0·22)	0·47	412
Exclusively breastfed child first 6 months without giving chupón	0·14 (-0·016 to 0·29)	0·079	412
Breastfed immediately after birth	0·10 (-0·0032 to 0·20)	0·057	335

For peer review only - http://bmjopen.bmj.com/site/about/guidelines.xhtml

For peer review only

**Paternal involvement**			
Father accompanied mother to clinic for prenatal care visit at least once	-0·060 (-0·26 to 0·14)	0·55	394
Father accompanied mother to seek medical care for pregnancy danger sign	0·11 (-0·15 to 0·38)	0·41	94
Father waited at birthplace during labor	-0·017 (-0·17 to 0·14)	0·83	412
Father accompanied mother to seek medical care for postnatal danger sign	0·22 (-0·10 to 0·54)	0·18	66
Father sought medical care for newborn for postnatal danger sign	-0·028 (-0·42 to 0·37)	0·89	83
Father held child	0·054 (-0·038 to 0·14)	0·25	406
Father played with child	0·025 (-0·13 to 0·18)	0·75	406
Father took child to clinic when sick	0·059 (-0·11 to 0·23)	0·51	406
**Diarrhea management**			
Did not report diarrhea in past 4 weeks	-0·013 (-0·034 to 0·0070)	0·20	6318
Used ORT to treat diarrhea in past 4 weeks	-0·14 (-0·39 to 0·12)	0·29	181
Used appropriate treatment for diarrhea in past 4 weeks[a]	0·069 (-0·16 to 0·30)	0·55	183
Child did not experience diarrhea past 4 weeks	-0·0035 (-0·040 to 0·033)	0·85	2891
Child experienced diarrhea past 4 weeks, was given appropriate amount of liquids	-0·13 (-0·32 to 0·064)	0·19	291
Child experienced diarrhea past 4 weeks, was given appropriate amount of food	-0·11 (-0·25 to 0·032)	0·13	291
Child experienced diarrhea past 4 weeks, was given ORT	0·13 (-0·052 to 0·32)	0·16	289
Child experienced diarrhea past 4 weeks, was given appropriate treatment	0·090 (-0·081 to 0·26)	0·30	291
Child experienced diarrhea past 4 weeks, was given zinc 10-14 days	0·16 (-0·033 to 0·35)	0·10	260
**Respiratory illness**			
Did not report respiratory illness (coughing) for 2 weeks	-0·040 (-0·082 to 0·0022)	0·063	6318
Child did not have cough past 4 weeks	-0·061 (-0·12 to 0·0012)	0·055	2892
Child had cough past 4 weeks, care was sought	0·055 (-0·068 to 0·18)	0·38	616
**Reproductive health**			
Reported ever using birth control to delay or avoid pregnancy	0·020 (-0·041 to 0·081)	0·52	6316
Reported currently using birth control	0·030 (-0·061 to 0·12)	0·52	2570
**Gender/reproductive norms**			
Birth location chosen either jointly or by woman	0·032 (-0·092 to 0·16)	0·62	412

ORT=Oral rehydration therapy. Standard errors are clustered at the village level. Robust 95% CIs in parentheses.

[a] ‘Used appropriate treatment for diarrhea in past 4 weeks’ outcome was coded as correct if respondent did any of the following: antibiotic, zinc, give extra food, give extra liquid, and did not do any of the following: anti-diarrhea medication, home remedy, laxative, deworming medication, chupon, massage, stop eating foods, stop taking liquids.

For peer review only

Table S3. Pooled total effect estimates of intervention on maternal, child, and neonatal outcomes, estimated on sub-sample of respondents who did not move between villages during study

	Outcome type	Respondent denominator	Pooled total effect estimate		N
			Effect size (95% CI)	p value	
**Prenatal care**					
Currently taking folic acid tablets (daily in past 7 days)	Practice	All women 15+ with a complete survey	-0·0029 (-0·048 to 0·042)	0·90	6035
Made a birth plan in preparation for birth	Practice	All respondents with a complete survey and a child born since 2018-09-01 (after the end of the intervention)	0·070 (-0·022 to 0·16)	0·13	567
Saved money in preparation for birth	Practice	All respondents with a complete survey and a child born since 2018-09-01 (after the end of the intervention)	-0·012 (-0·14 to 0·11)	0·85	568
Sold animal in preparation for birth	Practice	All respondents with a complete survey and a child born since 2018-09-01 (after the end of the intervention)	-0·017 (-0·086 to 0·052)	0·62	567
Sought prenatal care within 12 weeks	Practice	All respondents with a complete survey and a child born since 2018-09-01 (after the end of the intervention)	-0·052 (-0·20 to 0·097)	0·50	547
Identifies that women should take folic acid before pregnancy	Knowledge and attitudes	All respondents with a complete survey	0·042 (-0·0008 to 0·084)	0·054	9917
Identifies that women should seek prenatal care first 12 weeks of pregnancy	Knowledge and attitudes	All respondents with a complete survey	0·020 (-0·0077 to 0·048)	0·16	9917
Identifies accompanying woman to prenatal care visits as method of support during pregnancy	Knowledge and attitudes	All respondents with a complete survey	-0·0039 (-0·058 to 0·051)	0·89	9913
Identifies ensuring that woman eats well as method of support during pregnancy	Knowledge and attitudes	All respondents with a complete survey	-0·016 (-0·069 to 0·037)	0·56	9913
Identifies ensuring that woman rests 1 hour per day well as method of support during pregnancy	Knowledge and attitudes	All respondents with a complete survey	0·0091 (-0·024 to 0·042)	0·59	9913
Identifies avoiding violence as method of support during pregnancy	Knowledge and attitudes	All respondents with a complete survey	-0·034 (-0·055 to -0·013)	0·0018	9913
Identifies encouraging women to take vitamins as method of support during pregnancy	Knowledge and attitudes	All respondents with a complete survey	-0·022 (-0·060 to 0·017)	0·27	9913
Identifies helping woman with house work/child care as method of support during pregnancy	Knowledge and attitudes	All respondents with a complete survey	0·074 (0·0092 to 0·14)	0·025	9913
Identifies saving animals to sell as method of preparing for birth expenses	Knowledge and attitudes	All respondents with a complete survey	0·032 (0·0013 to 0·063)	0·041	9917

For peer review only - http://bmjopen.bmj.com/site/about/guidelines.xhtml

Identifies knowing cost of trip to maternal clinic as method of preparing for birth expenses	Knowledge and attitudes	All respondents with a complete survey	-0·0050 (-0·037 to 0·027)	0·76	9917
Identifies having a savings plan as method of preparing for birth expenses	Knowledge and attitudes	All respondents with a complete survey	0·033 (0·0025 to 0·063)	0·034	9917
Correctly answered prenatal care knowledge riddle	Intervention knowledge	All respondents with a complete survey	0·078 (0·039 to 0·12)	0·0001	9916
**Facility based birth**					
Gave birth in health facility	Practice	All respondents with a complete survey and a child born since 2018-09-01 (after the end of the intervention)	-0·0002 (-0·15 to 0·15)	1·00	568
Believes health facility is best place to give birth	Knowledge and attitudes	All respondents with a complete survey	0·0074 (-0·030 to 0·044)	0·69	9917
**Pregnancy danger signs**					
Woman sought medical care for pregnancy danger sign	Practice	All respondents with a complete survey and a child born since 2018-09-01 (after the end of the intervention) who experienced a prenatal care danger sign	-0·12 (-0·36 to 0·12)	0·33	210
Identified bleeding as pregnancy d.s.	Knowledge and attitudes	All respondents with a complete survey	0·040 (-0·0049 to 0·084)	0·081	9916
Identified seizure as pregnancy d.s.	Knowledge and attitudes	All respondents with a complete survey	0·0030 (-0·0064 to 0·012)	0·53	9916
Identified headache as pregnancy d.s.	Knowledge and attitudes	All respondents with a complete survey	0·040 (-0·014 to 0·093)	0·14	9916
Identified ringing in ears as pregnancy d.s.	Knowledge and attitudes	All respondents with a complete survey	0·0053 (-0·0054 to 0·016)	0·33	9916
Identified dizziness as pregnancy d.s.	Knowledge and attitudes	All respondents with a complete survey	-0·013 (-0·064 to 0·038)	0·61	9916
Identified difficulty urinating as pregnancy d.s.	Knowledge and attitudes	All respondents with a complete survey	-0·0002 (-0·0082 to 0·0077)	0·95	9916
Identified reduced or absent fetal movement as pregnancy d.s.	Knowledge and attitudes	All respondents with a complete survey	0·019 (-0·0052 to 0·043)	0·13	9916
Identified water breaking as pregnancy d.s.	Knowledge and attitudes	All respondents with a complete survey	-0·0022 (-0·0091 to 0·0047)	0·53	9916
Identified fever as pregnancy d.s.	Knowledge and attitudes	All respondents with a complete survey	0·036 (-0·0024 to 0·075)	0·066	9916
Identified swelling of face/hands/feet as pregnancy d.s.	Knowledge and attitudes	All respondents with a complete survey	0·035 (0·0030 to 0·067)	0·032	9916
**Postnatal care for mother**					
Mother had health checked by professional within 3 days of birth	Practice	All respondents with a complete survey and a child born since 2018-09-01 (after the end of the intervention)	0·12 (-0·047 to 0·30)	0·15	555

For peer review only - http://bmjopen.bmj.com/site/about/guidelines.xhtml

For peer review only

Mother had health checked by professional within 7 days of birth	Practice	All respondents with a complete survey and a child born since 2018-09-01 (after the end of the intervention)	0·10 (-0·072 to 0·27)	0·25	555
Mother sought medical care for postnatal danger sign	Practice	All respondents with a complete survey and a child born since 2018-09-01 (after the end of the intervention)	0·21 (-0·019 to 0·45)	0·071	178
Identifies that mother should receive postnatal medical check-up within 3 days of birth	Knowledge and attitudes	All respondents with a complete survey	0·087 (0·041 to 0·13)	0·0002	9917
Identifies that mother should receive postnatal medical check-up within 7 days of birth	Knowledge and attitudes	All respondents with a complete survey	0·060 (0·0079 to 0·11)	0·024	9917
Identified heavy vaginal bleeding as postnatal d.s.	Knowledge and attitudes	All respondents with a complete survey	0·053 (0·012 to 0·094)	0·011	9915
Identified fever as postnatal d.s.	Knowledge and attitudes	All respondents with a complete survey	0·049 (0·0007 to 0·098)	0·047	9915
Identified vaginal discharge as postnatal d.s.	Knowledge and attitudes	All respondents with a complete survey	0·0026 (-0·010 to 0·015)	0·68	9915
Identified headache as postnatal d.s.	Knowledge and attitudes	All respondents with a complete survey	0·029 (-0·011 to 0·069)	0·16	9915
Identified convulsions or fits as postnatal d.s.	Knowledge and attitudes	All respondents with a complete survey	0·0006 (-0·0050 to 0·0062)	0·84	9915
**Postnatal care for newborn**					
Newborn had health checked by professional within 3 days of birth	Practice	All respondents with a complete survey and a child born since 2018-09-01 (after the end of the intervention)	0·15 (0·016 to 0·29)	0·029	560
Newborn had health checked by professional within 7 days of birth	Practice	All respondents with a complete survey and a child born since 2018-09-01 (after the end of the intervention)	0·11 (-0·036 to 0·26)	0·14	560
Newborn experienced health problem in first month, care was sought	Practice	All respondents with a complete survey and a child born since 2018-09-01 (after the end of the intervention)	0·067 (-0·20 to 0·33)	0·62	160
Identified correct ways to provide newborn care[a]	Knowledge and attitudes	All respondents with a complete survey	0·026 (0·0096 to 0·043)	0·0019	9917
Identified fever as newborn d.s.	Knowledge and attitudes	All respondents with a complete survey	0·0054 (-0·046 to 0·057)	0·84	9915
Identified diarrhea as newborn d.s.	Knowledge and attitudes	All respondents with a complete survey	0·015 (-0·028 to 0·057)	0·50	9915
Identified difficulty breathing as newborn d.s.	Knowledge and attitudes	All respondents with a complete survey	0·0085 (-0·029 to 0·046)	0·66	9915
Identified vomiting as newborn d.s.	Knowledge and attitudes	All respondents with a complete survey	0·011 (-0·017 to 0·038)	0·45	9915
Identified poor appetite as newborn d.s.	Knowledge and attitudes	All respondents with a complete survey	-0·0062 (-0·016 to 0·0039)	0·23	9915
Identified redness/bleeding around cord as newborn d.s.	Knowledge and attitudes	All respondents with a complete survey	0·016 (-0·0053 to 0·037)	0·14	9915
Identified pus in cord as newborn d.s.	Knowledge and attitudes	All respondents with a complete survey	0·013 (-0·0024 to 0·029)	0·097	9915
Identified cold skin as newborn d.s.	Knowledge and attitudes	All respondents with a complete survey	0·0018 (-0·0069 to 0·011)	0·68	9915

For peer review only - http://bmjopen.bmj.com/site/about/guidelines.xhtml

Identified cough as newborn d.s.	Knowledge and attitudes	All respondents with a complete survey	-0·018 (-0·065 to 0·029)	0·45	9915
Identified cold as newborn d.s.	Knowledge and attitudes	All respondents with a complete survey	-0·016 (-0·072 to 0·040)	0·58	9915
Identified problems latching as newborn d.s.	Knowledge and attitudes	All respondents with a complete survey	-0·0029 (-0·016 to 0·010)	0·67	9915
Identified pneumonia as newborn d.s.	Knowledge and attitudes	All respondents with a complete survey	-0·0009 (-0·039 to 0·037)	0·96	9915
Did not use harmful substances to treat cord stump	Practice	All respondents with a complete survey and a child born since 2018-09-01 (after the end of the intervention)	0·098 (-0·043 to 0·24)	0·17	546
Did not wrap fajero around newborn in first 7 days after birth	Practice	All respondents with a complete survey and a child born since 2018-09-01 (after the end of the intervention)	0·20 (0·038 to 0·37)	0·016	565
Identified proper cord care methods[b]	Knowledge and attitudes	All respondents with a complete survey	0·097 (0·046 to 0·15)	0·0002	9916
Correctly answered proper cord care riddle	Intervention knowledge	All respondents with a complete survey	0·19 (0·13 to 0·24)	<0·0001	9917
Kept newborn wrapped first 7 days after birth	Practice	All respondents with a complete survey and a child born since 2018-09-01 (after the end of the intervention)	-0·040 (-0·10 to 0·022)	0·21	567
Held newborn skin-to-skin during first month after birth	Practice	All respondents with a complete survey and a child born since 2018-09-01 (after the end of the intervention)	0·15 (-0·0050 to 0·30)	0·058	565
**Breastfeeding**					
Exclusively breastfed child first 6 months	Practice	All respondents with a complete survey and a child born since 2018-09-01 (after the end of the intervention)	0·0034 (-0·16 to 0·17)	0·97	567
Exclusively breastfed child first 6 months without giving chupón	Practice	All respondents with a complete survey and a child born since 2018-09-01 (after the end of the intervention)	0·10 (-0·049 to 0·26)	0·18	567
Breastfed immediately after birth	Practice	All respondents with a complete survey and a child born since 2018-09-01 (after the end of the intervention) who reported a natural birth	0·12 (0·035 to 0·21)	0·0055	452
Identifies that newborn should be breastfed immediately after birth	Knowledge and attitudes	All respondents with a complete survey	0·018 (-0·015 to 0·051)	0·28	9917
Identifies that newborns should only be given breast milk during first 6 months	Knowledge and attitudes	All respondents with a complete survey	0·028 (-0·0042 to 0·061)	0·088	9916
Believes newborns should not be given chupón during first 6 months	Knowledge and attitudes	All respondents with a complete survey	0·14 (0·082 to 0·20)	<0·0001	9917
**Paternal involvement**					
Father accompanied mother to clinic for prenatal care visit at least once	Practice	All respondents with a complete survey and a child born since 2018-09-01 (after the end of the intervention) who received prenatal care	-0·053 (-0·25 to 0·14)	0·60	544
Father accompanied mother to seek medical care for pregnancy danger sign	Practice	All respondents with a complete survey and a child born since 2018-09-01 (after the end of the intervention) who experienced a prenatal care danger sign and sought care	-0·073 (-0·30 to 0·16)	0·53	134

For peer review only - http://bmjopen.bmj.com/site/about/guidelines.xhtml

Father waited at birthplace during labor	Practice	All respondents with a complete survey and a child born since 2018-09-01 (after the end of the intervention)	-0·036 (-0·18 to 0·11)	0·63	568
Father accompanied mother to seek medical care for postnatal danger sign	Practice	All respondents with a complete survey and a child born since 2018-09-01 (after the end of the intervention) and sought care for postpartum problem	0·13 (-0·19 to 0·45)	0·43	90
Father sought medical care for newborn for postnatal danger sign	Practice	All respondents with a complete survey and a child born since 2018-09-01 (after the end of the intervention) and sought care for a postnatal problem	-0·060 (-0·43 to 0·31)	0·75	103
Father held child	Practice	All respondents with a complete survey and a child born since 2018-09-01 (after the end of the intervention)	0·033 (-0·044 to 0·11)	0·40	563
Father played with child	Practice	All respondents with a complete survey and a child born since 2018-09-01 (after the end of the intervention)	-0·023 (-0·15 to 0·11)	0·73	563
Father took child to clinic when sick	Practice	All respondents with a complete survey and a child born since 2018-09-01 (after the end of the intervention)	0·014 (-0·13 to 0·16)	0·85	563
Believes father should accompany mother to prenatal care visits	Knowledge and attitudes	All respondents with a complete survey	0·044 (0·019 to 0·069)	0·0006	9876
Believes father should wait at birth location while mother gives birth	Knowledge and attitudes	All respondents with a complete survey	0·011 (-0·0068 to 0·029)	0·22	9881
Believes father should care for children when sick	Knowledge and attitudes	All respondents with a complete survey	-0·0025 (-0·010 to 0·0051)	0·52	9906
**Diarrhea management**					
Did not report diarrhea in past 4 weeks	Practice	All respondents with a complete survey	-0·0097 (-0·026 to 0·0066)	0·24	9918
Used ORT to treat diarrhea in past 4 weeks	Practice	All respondents with a complete survey who had diarrhea in past 4 weeks	-0·19 (-0·39 to 0·0012)	0·051	263
Used appropriate treatment for diarrhea in past 4 weeks[c]	Practice	All respondents with a complete survey who had diarrhea in past 4 weeks	-0·0006 (-0·16 to 0·16)	0·99	265
Child did not experience diarrhea past 4 weeks	Practice	All respondents with a complete survey and a new child <= 5 years old reported at wave 3 who had diarrhea in past 4 weeks	-0·0045 (-0·042 to 0·033)	0·82	3150
Child experienced diarrhea past 4 weeks, was given appropriate amount of liquids	Practice	All respondents with a complete survey and a new child <= 5 years old reported at wave 3 who had diarrhea in past 4 weeks	-0·15 (-0·34 to 0·037)	0·12	327
Child experienced diarrhea past 4 weeks, was given appropriate amount of food	Practice	All respondents with a complete survey and a new child <= 5 years old reported at wave 3 who had diarrhea in past 4 weeks	-0·12 (-0·26 to 0·015)	0·081	327
Child experienced diarrhea past 4 weeks, was given ORT	Practice	All respondents with a complete survey and a new child <= 5 years old reported at wave 3 who had diarrhea in past 4 weeks	0·12 (-0·054 to 0·30)	0·17	324
Child experienced diarrhea past 4 weeks, was given appropriate treatment	Practice	All respondents with a complete survey and a new child <= 5 years old reported at wave 3 who had diarrhea in past 4 weeks	0·12 (-0·056 to 0·29)	0·19	326
Child experienced diarrhea past 4 weeks, was given zinc 10-14 days	Practice	All respondents with a complete survey and a new child <= 5 years old reported at wave 3 who had diarrhea in past 4 weeks	0·17 (-0·014 to 0·35)	0·071	291

For peer review only - http://bmjopen.bmj.com/site/about/guidelines.xhtml

Identified appropriate diarrhea treatment methods[d]	Knowledge and attitudes	All respondents with a complete survey	0·097 (0·040 to 0·15)	0·0008	9916
Identified zinc 10-14 days as diarrhea treatment	Knowledge and attitudes	All respondents with a complete survey	0·030 (0·0077 to 0·052)	0·0081	9917
Identified zinc supplement as way to prevent diarrhea	Knowledge and attitudes	All respondents with a complete survey	0·061 (-0·0089 to 0·13)	0·087	9917
Identified breastfeeding as way to prevent diarrhea	Knowledge and attitudes	All respondents with a complete survey	0·036 (-0·032 to 0·10)	0·30	9917
Correctly answered diarrhea treatment with zinc riddle	Intervention knowledge	All respondents with a complete survey	0·23 (0·16 to 0·29)	<0·0001	9916
**Respiratory illness**					
Did not report respiratory illness (coughing) for 2 weeks	Practice	All respondents with a complete survey	-0·015 (-0·049 to 0·019)	0·39	9918
Child did not have cough past 4 weeks	Practice	All respondents with a complete survey	-0·067 (-0·13 to -0·0079)	0·027	3150
Child had cough past 4 weeks, care was sought	Practice	All respondents with a complete survey and a new child <= 5 years old reported at wave 3 who had an illness with a cough in past 4 weeks	0·035 (-0·091 to 0·16)	0·58	661
Identified vaccination as way to prevent respiratory illness	Knowledge and attitudes	All respondents with a complete survey	-0·022 (-0·060 to 0·017)	0·27	9917
Identified breastfeeding as way to prevent respiratory illness	Knowledge and attitudes	All respondents with a complete survey	0·013 (-0·042 to 0·068)	0·65	9917
Identified fever as d.s. for children with respiratory illness	Knowledge and attitudes	All respondents with a complete survey	0·0052 (-0·027 to 0·037)	0·75	9917
Identified difficulty breathing as d.s. for children with respiratory illness	Knowledge and attitudes	All respondents with a complete survey	0·0048 (-0·014 to 0·023)	0·61	9917
Identified rapid breathing as d.s. for children with respiratory illness	Knowledge and attitudes	All respondents with a complete survey	0·0050 (-0·0070 to 0·017)	0·41	9917
**Reproductive health**					
Reported ever using birth control to delay or avoid pregnancy	Practice	All respondents with a complete survey	-0·022 (-0·081 to 0·036)	0·46	9913
Reported currently using birth control	Practice	All respondents with a complete survey who are not pregnant and have ever used birth control	0·047 (-0·028 to 0·12)	0·22	3604
Believes woman should be at least 18 years of age to have her first child	Knowledge and attitudes	All respondents with a complete survey	0·011 (0·0023 to 0·020)	0·014	9407
**Gender/reproductive norms**					

For peer review only - http://bmjopen.bmj.com/site/about/guidelines.xhtml

Birth location chosen either jointly or by woman	Practice	All respondents with a complete survey and a child born since 2018-09-01 (after the end of the intervention)	0·048 (-0·054 to 0·15)	0·36	568

Results based on sub-sample of respondents who didn't move villages during the study N=9918 (household targeted n=3129, household not targeted n=6789). d.s.=Danger sign, ORT=Oral rehydration therapy. Standard errors are clustered at the village level. Robust 95% CIs in parentheses.

[a] 'Identified correct ways to provide newborn care' outcome was coded as correct if the respondent identified holding baby skin to skin, getting their health checked by a professional, immediately putting them to the breast, or making sure their diaper is clean and dry, and did not identify giving purgante, wrapping a fajero, giving a chupon, or bathing them in water right away.
[b] 'Identified proper cord care' outcome was coded as correct if respondent identified applying alcohol, applying iodine, keeping cord clean/dry, or applying peroxide, and did not identify applying oil, applying coffee, applying quina water, wrapping a fajero, applying powder, burning, applying ashes, applying a clamp, or applying thiomersal.
[c] 'Used appropriate treatment for diarrhea in past 4 weeks' outcome was coded as correct if respondent did any of the following: antibiotic, zinc, give extra food, give extra liquid, and did not do any of the following: anti-diarrhea medication, home remedy, laxative, deworming medication, chupon, massage, stop eating foods, stop taking liquids.
[d] 'Identified appropriate diarrhea treatment methods' outcome was coded as correct if respondent identified any of the following: antibiotic, zinc, ORS or Litrosol, give extra food, give extra liquid, get medical attention, and did not identify any of the following: anti-diarrhea medication, laxative, deworming medication, home remedy, chupon, massage, stop giving foods, stop giving liquids, or do nothing.

For peer review only

For peer review only - http://bmjopen.bmj.com/site/about/guidelines.xhtml

Table S4. Pooled total effect estimates of intervention on maternal, child, and neonatal outcomes, with adjustment for baseline demographic characteristics

	Outcome type	Respondent denominator	Pooled total effect estimate		N
			Effect size (95% CI)	p value	
**Prenatal care**					
Currently taking folic acid tablets (daily in past 7 days)	Practice	All women 15+ with a complete survey	-0·0059 (-0·049 to 0·037)	0·79	5922
Made a birth plan in preparation for birth	Practice	All respondents with a complete survey and a child born since 2018-09-01 (after the end of the intervention)	0·026 (-0·080 to 0·13)	0·63	536
Saved money in preparation for birth	Practice	All respondents with a complete survey and a child born since 2018-09-01 (after the end of the intervention)	-0·090 (-0·22 to 0·039)	0·17	540
Sold animal in preparation for birth	Practice	All respondents with a complete survey and a child born since 2018-09-01 (after the end of the intervention)	-0·013 (-0·078 to 0·051)	0·69	535
Sought prenatal care within 12 weeks	Practice	All respondents with a complete survey and a child born since 2018-09-01 (after the end of the intervention)	-0·0044 (-0·13 to 0·12)	0·94	518
Identifies that women should take folic acid before pregnancy	Knowledge and attitudes	All respondents with a complete survey	0·060 (0·028 to 0·093)	0·0003	9197
Identifies that women should seek prenatal care first 12 weeks of pregnancy	Knowledge and attitudes	All respondents with a complete survey	0·032 (0·011 to 0·053)	0·0033	9196
Identifies accompanying woman to prenatal care visits as method of support during pregnancy	Knowledge and attitudes	All respondents with a complete survey	0·0020 (-0·048 to 0·052)	0·94	9192
Identifies ensuring that woman eats well as method of support during pregnancy	Knowledge and attitudes	All respondents with a complete survey	-0·027 (-0·077 to 0·023)	0·29	9192
Identifies ensuring that woman rests 1 hour per day well as method of support during pregnancy	Knowledge and attitudes	All respondents with a complete survey	0·0095 (-0·022 to 0·041)	0·56	9183
Identifies avoiding violence as method of support during pregnancy	Knowledge and attitudes	All respondents with a complete survey	-0·029 (-0·051 to -0·0079)	0·0073	9192
Identifies encouraging women to take vitamins as method of support during pregnancy	Knowledge and attitudes	All respondents with a complete survey	-0·019 (-0·054 to 0·017)	0·30	9192
Identifies helping woman with house work/child care as method of support during pregnancy	Knowledge and attitudes	All respondents with a complete survey	0·085 (0·030 to 0·14)	0·0022	9192
Identifies saving animals to sell as method of preparing for birth expenses	Knowledge and attitudes	All respondents with a complete survey	0·023 (-0·0023 to 0·048)	0·075	9187

For peer review only

For peer review only - http://bmjopen.bmj.com/site/about/guidelines.xhtml

Identifies knowing cost of trip to maternal clinic as method of preparing for birth expenses	Knowledge and attitudes	All respondents with a complete survey	-0·0044 (-0·035 to 0·026)	0·78	9196
Identifies having a savings plan as method of preparing for birth expenses	Knowledge and attitudes	All respondents with a complete survey	0·039 (0·012 to 0·067)	0·0047	9172
Correctly answered prenatal care knowledge riddle	Intervention knowledge	All respondents with a complete survey	0·090 (0·054 to 0·13)	<0·0001	9196
**Facility based birth**					
Gave birth in health facility	Practice	All respondents with a complete survey and a child born since 2018-09-01 (after the end of the intervention)	0·026 (-0·088 to 0·14)	0·66	540
Believes health facility is best place to give birth	Knowledge and attitudes	All respondents with a complete survey	0·024 (-0·00060 to 0·049)	0·056	9188
**Pregnancy danger signs**					
Woman sought medical care for pregnancy danger sign	Practice	All respondents with a complete survey and a child born since 2018-09-01 (after the end of the intervention) who experienced a prenatal care danger sign	-0·040 (-0·25 to 0·17)	0·71	209
Identified bleeding as pregnancy d.s.	Knowledge and attitudes	All respondents with a complete survey	0·051 (0·013 to 0·090)	0·0090	9195
Identified seizure as pregnancy d.s.	Knowledge and attitudes	All respondents with a complete survey	0·0020 (-0·0076 to 0·012)	0·68	9186
Identified headache as pregnancy d.s.	Knowledge and attitudes	All respondents with a complete survey	0·036 (-0·010 to 0·083)	0·13	9195
Identified ringing in ears as pregnancy d.s.	Knowledge and attitudes	All respondents with a complete survey	0·0031 (-0·0064 to 0·013)	0·52	9171
Identified dizziness as pregnancy d.s.	Knowledge and attitudes	All respondents with a complete survey	-0·0026 (-0·049 to 0·044)	0·91	9195
Identified difficulty urinating as pregnancy d.s.	Knowledge and attitudes	All respondents with a complete survey	-0·0018 (-0·0094 to 0·0059)	0·65	9171
Identified reduced or absent fetal movement as pregnancy d.s.	Knowledge and attitudes	All respondents with a complete survey	0·014 (-0·0060 to 0·034)	0·17	9171
Identified water breaking as pregnancy d.s.	Knowledge and attitudes	All respondents with a complete survey	-0·0007 (-0·0079 to 0·0066)	0·86	9171
Identified fever as pregnancy d.s.	Knowledge and attitudes	All respondents with a complete survey	0·040 (0·0012 to 0·079)	0·043	9195
Identified swelling of face/hands/feet as pregnancy d.s.	Knowledge and attitudes	All respondents with a complete survey	0·034 (0·0050 to 0·064)	0·022	9195
**Postnatal care for mother**					
Mother had health checked by professional within 3 days of birth	Practice	All respondents with a complete survey and a child born since 2018-09-01 (after the end of the intervention)	0·13 (-0·024 to 0·29)	0·096	530

For peer review only - http://bmjopen.bmj.com/site/about/guidelines.xhtml

Mother had health checked by professional within 7 days of birth	Practice	All respondents with a complete survey and a child born since 2018-09-01 (after the end of the intervention)	0·075 (-0·065 to 0·22)	0·30	530
Mother sought medical care for postnatal danger sign	Practice	All respondents with a complete survey and a child born since 2018-09-01 (after the end of the intervention)	0·12 (-0·072 to 0·31)	0·23	178
Identifies that mother should receive postnatal medical check-up within 3 days of birth	Knowledge and attitudes	All respondents with a complete survey	0·073 (0·031 to 0·11)	0·0006	9196
Identifies that mother should receive postnatal medical check-up within 7 days of birth	Knowledge and attitudes	All respondents with a complete survey	0·040 (-0·0085 to 0·089)	0·11	9196
Identified heavy vaginal bleeding as postnatal d.s.	Knowledge and attitudes	All respondents with a complete survey	0·067 (0·029 to 0·11)	0·0006	9195
Identified fever as postnatal d.s.	Knowledge and attitudes	All respondents with a complete survey	0·060 (0·0098 to 0·11)	0·019	9195
Identified vaginal discharge as postnatal d.s.	Knowledge and attitudes	All respondents with a complete survey	0·0046 (-0·010 to 0·019)	0·54	9180
Identified headache as postnatal d.s.	Knowledge and attitudes	All respondents with a complete survey	0·029 (-0·0086 to 0·066)	0·13	9195
Identified convulsions or fits as postnatal d.s.	Knowledge and attitudes	All respondents with a complete survey	0·00 (-0·0056 to 0·0056)	1·00	8983
**Postnatal care for newborn**					
Newborn had health checked by professional within 3 days of birth	Practice	All respondents with a complete survey and a child born since 2018-09-01 (after the end of the intervention)	0·16 (0·029 to 0·30)	0·017	532
Newborn had health checked by professional within 7 days of birth	Practice	All respondents with a complete survey and a child born since 2018-09-01 (after the end of the intervention)	0·085 (-0·041 to 0·21)	0·19	532
Newborn experienced health problem in first month, care was sought	Practice	All respondents with a complete survey and a child born since 2018-09-01 (after the end of the intervention)	0·0030 (-0·23 to 0·23)	0·98	154
Identified correct ways to provide newborn care[a]	Knowledge and attitudes	All respondents with a complete survey	0·024 (0·010 to 0·037)	0·0005	9172
Identified fever as newborn d.s.	Knowledge and attitudes	All respondents with a complete survey	0·0022 (-0·045 to 0·049)	0·93	9195
Identified diarrhea as newborn d.s.	Knowledge and attitudes	All respondents with a complete survey	0·0035 (-0·037 to 0·044)	0·86	9195
Identified difficulty breathing as newborn d.s.	Knowledge and attitudes	All respondents with a complete survey	0·0099 (-0·025 to 0·045)	0·58	9171
Identified vomiting as newborn d.s.	Knowledge and attitudes	All respondents with a complete survey	0·0090 (-0·021 to 0·039)	0·56	9186
Identified poor appetite as newborn d.s.	Knowledge and attitudes	All respondents with a complete survey	-0·0049 (-0·015 to 0·0047)	0·32	9171
Identified redness/bleeding around cord as newborn d.s.	Knowledge and attitudes	All respondents with a complete survey	0·020 (-0·0017 to 0·041)	0·072	9195
Identified pus in cord as newborn d.s.	Knowledge and attitudes	All respondents with a complete survey	0·015 (-0·0014 to 0·031)	0·074	9186
Identified cold skin as newborn d.s.	Knowledge and attitudes	All respondents with a complete survey	0·0023 (-0·0068 to 0·012)	0·61	9171

For peer review only - http://bmjopen.bmj.com/site/about/guidelines.xhtml

Identified cough as newborn d.s.	Knowledge and attitudes	All respondents with a complete survey	-0·019 (-0·069 to 0·031)	0·45	9195
Identified cold as newborn d.s.	Knowledge and attitudes	All respondents with a complete survey	-0·017 (-0·067 to 0·034)	0·52	9195
Identified problems latching as newborn d.s.	Knowledge and attitudes	All respondents with a complete survey	-0·0013 (-0·015 to 0·012)	0·85	9171
Identified pneumonia as newborn d.s.	Knowledge and attitudes	All respondents with a complete survey	0·0061 (-0·030 to 0·042)	0·74	9171
Did not use harmful substances to treat cord stump	Practice	All respondents with a complete survey and a child born since 2018-09-01 (after the end of the intervention)	0·089 (-0·042 to 0·22)	0·18	527
Did not wrap fajero around newborn in first 7 days after birth	Practice	All respondents with a complete survey and a child born since 2018-09-01 (after the end of the intervention)	0·27 (0·11 to 0·42)	0·0006	535
Identified proper cord care methods[b]	Knowledge and attitudes	All respondents with a complete survey	0·095 (0·046 to 0·14)	0·0001	9195
Correctly answered proper cord care riddle	Intervention knowledge	All respondents with a complete survey	0·21 (0·16 to 0·25)	<0·0001	9197
Kept newborn wrapped first 7 days after birth	Practice	All respondents with a complete survey and a child born since 2018-09-01 (after the end of the intervention)	-0·050 (-0·11 to 0·012)	0·12	530
Held newborn skin-to-skin during first month after birth	Practice	All respondents with a complete survey and a child born since 2018-09-01 (after the end of the intervention)	0·11 (-0·019 to 0·24)	0·095	537
**Breastfeeding**					
Exclusively breastfed child first 6 months	Practice	All respondents with a complete survey and a child born since 2018-09-01 (after the end of the intervention)	-0·0046 (-0·14 to 0·14)	0·95	539
Exclusively breastfed child first 6 months without giving chupón	Practice	All respondents with a complete survey and a child born since 2018-09-01 (after the end of the intervention)	0·061 (-0·082 to 0·20)	0·40	539
Breastfed immediately after birth	Practice	All respondents with a complete survey and a child born since 2018-09-01 (after the end of the intervention) who reported a natural birth	0·090 (-0·0078 to 0·19)	0·071	428
Identifies that newborn should be breastfed immediately after birth	Knowledge and attitudes	All respondents with a complete survey	0·016 (-0·018 to 0·050)	0·35	9196
Identifies that newborns should only be given breast milk during first 6 months	Knowledge and attitudes	All respondents with a complete survey	0·039 (0·0073 to 0·070)	0·015	9196
Believes newborns should not be given chupón during first 6 months	Knowledge and attitudes	All respondents with a complete survey	0·16 (0·10 to 0·21)	<0·0001	9196
**Paternal involvement**					
Father accompanied mother to clinic for prenatal care visit at least once	Practice	All respondents with a complete survey and a child born since 2018-09-01 (after the end of the intervention) who received prenatal care	-0·016 (-0·20 to 0·17)	0·87	519
Father accompanied mother to seek medical care for pregnancy danger sign	Practice	All respondents with a complete survey and a child born since 2018-09-01 (after the end of the intervention) who experienced a prenatal care danger sign and sought care	0·16 (-0·081 to 0·41)	0·19	134

For peer review only - http://bmjopen.bmj.com/site/about/guidelines.xhtml

Father waited at birthplace during labor	Practice	All respondents with a complete survey and a child born since 2018-09-01 (after the end of the intervention)	-0·022 (-0·15 to 0·11)	0·74	540
Father accompanied mother to seek medical care for postnatal danger sign	Practice	All respondents with a complete survey and a child born since 2018-09-01 (after the end of the intervention) and sought care for postpartum problem	0·099 (-0·16 to 0·36)	0·46	93
Father sought medical care for newborn for postnatal danger sign	Practice	All respondents with a complete survey and a child born since 2018-09-01 (after the end of the intervention) and sought care for a postnatal problem	-0·010 (-0·30 to 0·28)	0·95	98
Father held child	Practice	All respondents with a complete survey and a child born since 2018-09-01 (after the end of the intervention)	0·045 (-0·028 to 0·12)	0·23	535
Father played with child	Practice	All respondents with a complete survey and a child born since 2018-09-01 (after the end of the intervention)	0·013 (-0·10 to 0·13)	0·83	535
Father took child to clinic when sick	Practice	All respondents with a complete survey and a child born since 2018-09-01 (after the end of the intervention)	0·091 (-0·040 to 0·22)	0·17	535
Believes father should accompany mother to prenatal care visits	Knowledge and attitudes	All respondents with a complete survey	0·038 (0·015 to 0·061)	0·0014	9158
Believes father should wait at birth location while mother gives birth	Knowledge and attitudes	All respondents with a complete survey	0·014 (-0·0044 to 0·032)	0·14	9156
Believes father should care for children when sick	Knowledge and attitudes	All respondents with a complete survey	0·0001 (-0·0059 to 0·0062)	0·97	9177
**Diarrhea management**					
Did not report diarrhea in past 4 weeks	Practice	All respondents with a complete survey	-0·0095 (-0·025 to 0·0063)	0·24	9182
Used ORT to treat diarrhea in past 4 weeks	Practice	All respondents with a complete survey who had diarrhea in past 4 weeks	-0·17 (-0·35 to 0·0073)	0·060	241
Used appropriate treatment for diarrhea in past 4 weeks[c]	Practice	All respondents with a complete survey who had diarrhea in past 4 weeks	-0·0029 (-0·14 to 0·14)	0·97	243
Child did not experience diarrhea past 4 weeks	Practice	All respondents with a complete survey and a new child <= 5 years old reported at wave 3 who had diarrhea in past 4 weeks	-0·0049 (-0·042 to 0·032)	0·80	3033
Child experienced diarrhea past 4 weeks, was given appropriate amount of liquids	Practice	All respondents with a complete survey and a new child <= 5 years old reported at wave 3 who had diarrhea in past 4 weeks	-0·16 (-0·32 to 0·0046)	0·057	310
Child experienced diarrhea past 4 weeks, was given appropriate amount of food	Practice	All respondents with a complete survey and a new child <= 5 years old reported at wave 3 who had diarrhea in past 4 weeks	-0·14 (-0·27 to -0·0099)	0·035	313
Child experienced diarrhea past 4 weeks, was given ORT	Practice	All respondents with a complete survey and a new child <= 5 years old reported at wave 3 who had diarrhea in past 4 weeks	0·14 (-0·035 to 0·32)	0·12	310
Child experienced diarrhea past 4 weeks, was given appropriate treatment	Practice	All respondents with a complete survey and a new child <= 5 years old reported at wave 3 who had diarrhea in past 4 weeks	0·13 (-0·038 to 0·29)	0·13	312
Child experienced diarrhea past 4 weeks, was given zinc 10-14 days	Practice	All respondents with a complete survey and a new child <= 5 years old reported at wave 3 who had diarrhea in past 4 weeks	..	..	..

For peer review only - http://bmjopen.bmj.com/site/about/guidelines.xhtml

Identified appropriate diarrhea treatment methods[d]	Knowledge and attitudes	All respondents with a complete survey	0·11 (0·056 to 0·16)	<0·0001	9195
Identified zinc 10-14 days as diarrhea treatment	Knowledge and attitudes	All respondents with a complete survey	0·033 (0·0097 to 0·056)	0·0053	9181
Identified zinc supplement as way to prevent diarrhea	Knowledge and attitudes	All respondents with a complete survey	0·073 (0·0073 to 0·14)	0·029	9196
Identified breastfeeding as way to prevent diarrhea	Knowledge and attitudes	All respondents with a complete survey	0·040 (-0·021 to 0·100)	0·20	9196
Correctly answered diarrhea treatment with zinc riddle	Intervention knowledge	All respondents with a complete survey	0·24 (0·19 to 0·30)	<0·0001	9197
**Respiratory illness**					
Did not report respiratory illness (coughing) for 2 weeks	Practice	All respondents with a complete survey	-0·013 (-0·046 to 0·020)	0·44	9188
Child did not have cough past 4 weeks	Practice	All respondents with a complete survey	-0·060 (-0·12 to -0·0046)	0·034	3034
Child had cough past 4 weeks, care was sought	Practice	All respondents with a complete survey and a new child <= 5 years old reported at wave 3 who had an illness with a cough in past 4 weeks	0·042 (-0·085 to 0·17)	0·52	647
Identified vaccination as way to prevent respiratory illness	Knowledge and attitudes	All respondents with a complete survey	-0·0017 (-0·039 to 0·036)	0·93	9196
Identified breastfeeding as way to prevent respiratory illness	Knowledge and attitudes	All respondents with a complete survey	0·030 (-0·021 to 0·081)	0·25	9196
Identified fever as d.s. for children with respiratory illness	Knowledge and attitudes	All respondents with a complete survey	0·015 (-0·012 to 0·043)	0·27	9187
Identified difficulty breathing as d.s. for children with respiratory illness	Knowledge and attitudes	All respondents with a complete survey	0·011 (-0·0049 to 0·027)	0·18	9187
Identified rapid breathing as d.s. for children with respiratory illness	Knowledge and attitudes	All respondents with a complete survey	0·0082 (-0·0024 to 0·019)	0·13	9187
**Reproductive health**					
Reported ever using birth control to delay or avoid pregnancy	Practice	All respondents with a complete survey	-0·0030 (-0·055 to 0·049)	0·91	9191
Reported currently using birth control	Practice	All respondents with a complete survey who are not pregnant and have ever used birth control	0·035 (-0·027 to 0·097)	0·27	3420
Believes woman should be at least 18 years of age to have her first child	Knowledge and attitudes	All respondents with a complete survey	0·012 (0·0048 to 0·019)	0·0011	8713
**Gender/reproductive norms**					
Birth location chosen either jointly or by woman	Practice	All respondents with a complete survey and a child born since 2018-09-01 (after the end of the intervention)	0·049 (-0·040 to 0·14)	0·28	540

For peer review only - http://bmjopen.bmj.com/site/about/guidelines.xhtml

Results based on sub-sample that completed baseline survey, N=9285 (household targeted n=2919, household not targeted n=6366). Baseline covariates included age, gender, education, marital status, indigenous status, and household wealth index. d.s.=Danger sign, ORT=Oral rehydration therapy. ··Model did not converge. Covariates were dropped sequentially if they predicted outcome perfectly. Standard errors are clustered at the village level. Robust 95% CIs in parentheses.

[a] 'Identified correct ways to provide newborn care' outcome was coded as correct if the respondent identified holding baby skin to skin, getting their health checked by a professional, immediately putting them to the breast, or making sure their diaper is clean and dry, and did not identify giving purgante, wrapping a fajero, giving a chupon, or bathing them in water right away.
[b] 'Identified proper cord care' outcome was coded as correct if respondent identified applying alcohol, applying iodine, keeping cord clean/dry, or applying peroxide, and did not identify applying oil, applying coffee, applying quina water, wrapping a fajero, applying powder, burning, applying ashes, applying a clamp, or applying thiomersal.
[c] 'Used appropriate treatment for diarrhea in past 4 weeks' outcome was coded as correct if respondent did any of the following: antibiotic, zinc, give extra food, give extra liquid, and did not do any of the following: anti-diarrhea medication, home remedy, laxative, deworming medication, chupon, massage, stop eating foods, stop taking liquids.
[d] 'Identified appropriate diarrhea treatment methods' outcome was coded as correct if respondent identified any of the following: antibiotic, zinc, ORS or Litrosol, give extra food, give extra liquid, get medical attention, and did not identify any of the following: anti-diarrhea medication, laxative, deworming medication, home remedy, chupon, massage, stop giving foods, stop giving liquids, or do nothing.

For peer review only - http://bmjopen.bmj.com/site/about/guidelines.xhtml

Table S5. Pooled total effect estimates of intervention on maternal, child, and neonatal outcomes, with adjustment for baseline outcome

	Outcome type	Respondent denominator	Pooled total effect estimate		N
			Effect size (95% CI)	p value	
**Prenatal care**					
Currently taking folic acid tablets (daily in past 7 days)	Practice	All women 15+ with a complete survey	0·0086 (-0·033 to 0·050)	0·69	5552
Made a birth plan in preparation for birth	Practice	All respondents with a complete survey and a child born since 2018-09-01 (after the end of the intervention)	-0·075 (-0·22 to 0·074)	0·32	299
Saved money in preparation for birth	Practice	All respondents with a complete survey and a child born since 2018-09-01 (after the end of the intervention)	0·044 (-0·092 to 0·18)	0·53	299
Sold animal in preparation for birth	Practice	All respondents with a complete survey and a child born since 2018-09-01 (after the end of the intervention)	NA	NA	NA
Sought prenatal care within 12 weeks	Practice	All respondents with a complete survey and a child born since 2018-09-01 (after the end of the intervention)	0·0028 (-0·16 to 0·16)	0·97	285
Identifies that women should take folic acid before pregnancy	Knowledge and attitudes	All respondents with a complete survey	0·045 (0·0062 to 0·083)	0·023	9283
Identifies that women should seek prenatal care first 12 weeks of pregnancy	Knowledge and attitudes	All respondents with a complete survey	0·024 (-0·0017 to 0·050)	0·067	9282
Identifies accompanying woman to prenatal care visits as method of support during pregnancy	Knowledge and attitudes	All respondents with a complete survey	NA	NA	NA
Identifies ensuring that woman eats well as method of support during pregnancy	Knowledge and attitudes	All respondents with a complete survey	NA	NA	NA
Identifies ensuring that woman rests 1 hour per day well as method of support during pregnancy	Knowledge and attitudes	All respondents with a complete survey	NA	NA	NA
Identifies avoiding violence as method of support during pregnancy	Knowledge and attitudes	All respondents with a complete survey	NA	NA	NA
Identifies encouraging women to take vitamins as method of support during pregnancy	Knowledge and attitudes	All respondents with a complete survey	NA	NA	NA
Identifies helping woman with house work/child care as method of support during pregnancy	Knowledge and attitudes	All respondents with a complete survey	NA	NA	NA
Identifies saving animals to sell as method of preparing for birth expenses	Knowledge and attitudes	All respondents with a complete survey	NA	NA	NA

For peer review only - http://bmjopen.bmj.com/site/about/guidelines.xhtml

Identifies knowing cost of trip to maternal clinic as method of preparing for birth expenses	Knowledge and attitudes	All respondents with a complete survey	NA	NA	NA
Identifies having a savings plan as method of preparing for birth expenses	Knowledge and attitudes	All respondents with a complete survey	NA	NA	NA
Correctly answered prenatal care knowledge riddle	Intervention knowledge	All respondents with a complete survey	NA	NA	NA
**Facility based birth**					
Gave birth in health facility	Practice	All respondents with a complete survey and a child born since 2018-09-01 (after the end of the intervention)	-0·055 (-0·20 to 0·092)	0·46	299
Believes health facility is best place to give birth	Knowledge and attitudes	All respondents with a complete survey	0·0065 (-0·023 to 0·036)	0·66	9283
**Pregnancy danger signs**					
Woman sought medical care for pregnancy danger sign	Practice	All respondents with a complete survey and a child born since 2018-09-01 (after the end of the intervention) who experienced a prenatal care danger sign	-0·34 (-0·81 to 0·13)	0·15	57
Identified bleeding as pregnancy d.s.	Knowledge and attitudes	All respondents with a complete survey	0·048 (0·0048 to 0·091)	0·030	9279
Identified seizure as pregnancy d.s.	Knowledge and attitudes	All respondents with a complete survey	0·0026 (-0·0071 to 0·012)	0·60	9279
Identified headache as pregnancy d.s.	Knowledge and attitudes	All respondents with a complete survey	0·045 (-0·0051 to 0·094)	0·079	9279
Identified ringing in ears as pregnancy d.s.	Knowledge and attitudes	All respondents with a complete survey	0·0050 (-0·0062 to 0·016)	0·38	9279
Identified dizziness as pregnancy d.s.	Knowledge and attitudes	All respondents with a complete survey	-0·0068 (-0·056 to 0·043)	0·79	9279
Identified difficulty urinating as pregnancy d.s.	Knowledge and attitudes	All respondents with a complete survey	-0·0001 (-0·0085 to 0·0084)	0·99	9279
Identified reduced or absent fetal movement as pregnancy d.s.	Knowledge and attitudes	All respondents with a complete survey	0·014 (-0·0055 to 0·034)	0·16	9279
Identified water breaking as pregnancy d.s.	Knowledge and attitudes	All respondents with a complete survey	-0·0006 (-0·0083 to 0·0071)	0·89	9279
Identified fever as pregnancy d.s.	Knowledge and attitudes	All respondents with a complete survey	0·047 (0·010 to 0·084)	0·013	9279
Identified swelling of face/hands/feet as pregnancy d.s.	Knowledge and attitudes	All respondents with a complete survey	0·037 (0·0039 to 0·070)	0·028	9279
**Postnatal care for mother**					
Mother had health checked by professional within 3 days of birth	Practice	All respondents with a complete survey and a child born since 2018-09-01 (after the end of the intervention)	0·024 (-0·18 to 0·23)	0·82	293

For peer review only - http://bmjopen.bmj.com/site/about/guidelines.xhtml

For peer review only

Mother had health checked by professional within 7 days of birth	Practice	All respondents with a complete survey and a child born since 2018-09-01 (after the end of the intervention)	0·0051 (-0·22 to 0·24)	0·97	293
Mother sought medical care for postnatal danger sign	Practice	All respondents with a complete survey and a child born since 2018-09-01 (after the end of the intervention)	0·21 (-0·15 to 0·57)	0·25	34
Identifies that mother should receive postnatal medical check-up within 3 days of birth	Knowledge and attitudes	All respondents with a complete survey	0·092 (0·045 to 0·14)	0·0001	9279
Identifies that mother should receive postnatal medical check-up within 7 days of birth	Knowledge and attitudes	All respondents with a complete survey	0·054 (0·0031 to 0·11)	0·038	9279
Identified heavy vaginal bleeding as postnatal d.s.	Knowledge and attitudes	All respondents with a complete survey	0·060 (0·021 to 0·098)	0·0024	9279
Identified fever as postnatal d.s.	Knowledge and attitudes	All respondents with a complete survey	0·059 (0·0096 to 0·11)	0·019	9279
Identified vaginal discharge as postnatal d.s.	Knowledge and attitudes	All respondents with a complete survey	0·0032 (-0·012 to 0·018)	0·67	9279
Identified headache as postnatal d.s.	Knowledge and attitudes	All respondents with a complete survey	0·031 (-0·0088 to 0·071)	0·13	9279
Identified convulsions or fits as postnatal d.s.	Knowledge and attitudes	All respondents with a complete survey	0·0003 (-0·0055 to 0·0062)	0·91	9279
**Postnatal care for newborn**					
Newborn had health checked by professional within 3 days of birth	Practice	All respondents with a complete survey and a child born since 2018-09-01 (after the end of the intervention)	0·091 (-0·095 to 0·28)	0·34	297
Newborn had health checked by professional within 7 days of birth	Practice	All respondents with a complete survey and a child born since 2018-09-01 (after the end of the intervention)	0·043 (-0·18 to 0·27)	0·71	297
Newborn experienced health problem in first month, care was sought	Practice	All respondents with a complete survey and a child born since 2018-09-01 (after the end of the intervention)	-0·031 (-0·57 to 0·51)	0·91	25
Identified correct ways to provide newborn care[a]	Knowledge and attitudes	All respondents with a complete survey	0·026 (0·010 to 0·042)	0·0011	9282
Identified fever as newborn d.s.	Knowledge and attitudes	All respondents with a complete survey	0·015 (-0·033 to 0·062)	0·54	9281
Identified diarrhea as newborn d.s.	Knowledge and attitudes	All respondents with a complete survey	0·020 (-0·023 to 0·062)	0·36	9281
Identified difficulty breathing as newborn d.s.	Knowledge and attitudes	All respondents with a complete survey	0·011 (-0·026 to 0·049)	0·55	9281
Identified vomiting as newborn d.s.	Knowledge and attitudes	All respondents with a complete survey	0·013 (-0·017 to 0·042)	0·39	9281
Identified poor appetite as newborn d.s.	Knowledge and attitudes	All respondents with a complete survey	-0·0039 (-0·014 to 0·0067)	0·47	9281
Identified redness/bleeding around cord as newborn d.s.	Knowledge and attitudes	All respondents with a complete survey	0·017 (-0·0038 to 0·039)	0·11	9281
Identified pus in cord as newborn d.s.	Knowledge and attitudes	All respondents with a complete survey	0·015 (-0·0014 to 0·031)	0·074	9281
Identified cold skin as newborn d.s.	Knowledge and attitudes	All respondents with a complete survey	0·0018 (-0·0076 to 0·011)	0·70	9281

For peer review only - http://bmjopen.bmj.com/site/about/guidelines.xhtml

Identified cough as newborn d.s.	Knowledge and attitudes	All respondents with a complete survey	-0·018 (-0·066 to 0·031)	0·48	9281
Identified cold as newborn d.s.	Knowledge and attitudes	All respondents with a complete survey	-0·025 (-0·080 to 0·030)	0·37	9281
Identified problems latching as newborn d.s.	Knowledge and attitudes	All respondents with a complete survey	-0·0025 (-0·017 to 0·012)	0·72	9279
Identified pneumonia as newborn d.s.	Knowledge and attitudes	All respondents with a complete survey	NA	NA	NA
Did not use harmful substances to treat cord stump	Practice	All respondents with a complete survey and a child born since 2018-09-01 (after the end of the intervention)	0·099 (-0·082 to 0·28)	0·29	289
Did not wrap fajero around newborn in first 7 days after birth	Practice	All respondents with a complete survey and a child born since 2018-09-01 (after the end of the intervention)	0·25 (0·043 to 0·46)	0·018	298
Identified proper cord care methods[b]	Knowledge and attitudes	All respondents with a complete survey	0·094 (0·047 to 0·14)	0·0001	9281
Correctly answered proper cord care riddle	Intervention knowledge	All respondents with a complete survey	NA	NA	NA
Kept newborn wrapped first 7 days after birth	Practice	All respondents with a complete survey and a child born since 2018-09-01 (after the end of the intervention)	-0·041 (-0·12 to 0·038)	0·31	293
Held newborn skin-to-skin during first month after birth	Practice	All respondents with a complete survey and a child born since 2018-09-01 (after the end of the intervention)	0·21 (-0·099 to 0·51)	0·19	72
**Breastfeeding**					
Exclusively breastfed child first 6 months	Practice	All respondents with a complete survey and a child born since 2018-09-01 (after the end of the intervention)	-0·15 (-0·33 to 0·035)	0·11	298
Exclusively breastfed child first 6 months without giving chupón	Practice	All respondents with a complete survey and a child born since 2018-09-01 (after the end of the intervention)	-0·079 (-0·29 to 0·13)	0·46	299
Breastfed immediately after birth	Practice	All respondents with a complete survey and a child born since 2018-09-01 (after the end of the intervention) who reported a natural birth	-0·017 (-0·15 to 0·11)	0·80	230
Identifies that newborn should be breastfed immediately after birth	Knowledge and attitudes	All respondents with a complete survey	0·0088 (-0·027 to 0·044)	0·63	9282
Identifies that newborns should only be given breast milk during first 6 months	Knowledge and attitudes	All respondents with a complete survey	0·038 (0·0086 to 0·068)	0·012	9282
Believes newborns should not be given chupón during first 6 months	Knowledge and attitudes	All respondents with a complete survey	0·14 (0·079 to 0·19)	<0·0001	9282
**Paternal involvement**					
Father accompanied mother to clinic for prenatal care visit at least once	Practice	All respondents with a complete survey and a child born since 2018-09-01 (after the end of the intervention) who received prenatal care	0·16 (-0·049 to 0·36)	0·14	276
Father accompanied mother to seek medical care for pregnancy danger sign	Practice	All respondents with a complete survey and a child born since 2018-09-01 (after the end of the intervention) who experienced a prenatal care danger sign and sought care	-0·017 (-0·71 to 0·67)	0·96	25

For peer review only - http://bmjopen.bmj.com/site/about/guidelines.xhtml

Father waited at birthplace during labor	Practice	All respondents with a complete survey and a child born since 2018-09-01 (after the end of the intervention)	-0·013 (-0·17 to 0·14)	0·87	299
Father accompanied mother to seek medical care for postnatal danger sign	Practice	All respondents with a complete survey and a child born since 2018-09-01 (after the end of the intervention) and sought care for postpartum problem	0·28 (-0·061 to 0·63)	0·11	14
Father sought medical care for newborn for postnatal danger sign	Practice	All respondents with a complete survey and a child born since 2018-09-01 (after the end of the intervention) and sought care for a postnatal problem	-0·062 (-0·64 to 0·51)	0·83	16
Father held child	Practice	All respondents with a complete survey and a child born since 2018-09-01 (after the end of the intervention)	-0·0032 (-0·12 to 0·11)	0·96	269
Father played with child	Practice	All respondents with a complete survey and a child born since 2018-09-01 (after the end of the intervention)	0·036 (-0·10 to 0·17)	0·61	275
Father took child to clinic when sick	Practice	All respondents with a complete survey and a child born since 2018-09-01 (after the end of the intervention)	0·068 (-0·11 to 0·25)	0·46	275
Believes father should accompany mother to prenatal care visits	Knowledge and attitudes	All respondents with a complete survey	0·043 (0·020 to 0·066)	0·00020	9214
Believes father should wait at birth location while mother gives birth	Knowledge and attitudes	All respondents with a complete survey	0·014 (-0·0049 to 0·032)	0·15	9225
Believes father should care for children when sick	Knowledge and attitudes	All respondents with a complete survey	-0·0010 (-0·0082 to 0·0061)	0·78	9266
**Diarrhea management**					
Did not report diarrhea in past 4 weeks	Practice	All respondents with a complete survey	-0·0099 (-0·026 to 0·0065)	0·24	9282
Used ORT to treat diarrhea in past 4 weeks	Practice	All respondents with a complete survey who had diarrhea in past 4 weeks	0·21 (-0·28 to 0·70)	0·40	23
Used appropriate treatment for diarrhea in past 4 weeks[c]	Practice	All respondents with a complete survey who had diarrhea in past 4 weeks	-0·24 (-0·48 to 0·0082)	0·058	23
Child did not experience diarrhea past 4 weeks	Practice	All respondents with a complete survey and a new child <= 5 years old reported at wave 3 who had diarrhea in past 4 weeks	-0·011 (-0·053 to 0·031)	0·60	2377
Child experienced diarrhea past 4 weeks, was given appropriate amount of liquids	Practice	All respondents with a complete survey and a new child <= 5 years old reported at wave 3 who had diarrhea in past 4 weeks	1·9 (1·5 to 2·3)	<0·0001	49
Child experienced diarrhea past 4 weeks, was given appropriate amount of food	Practice	All respondents with a complete survey and a new child <= 5 years old reported at wave 3 who had diarrhea in past 4 weeks	1·2 (0·67 to 1·7)	<0·0001	50
Child experienced diarrhea past 4 weeks, was given ORT	Practice	All respondents with a complete survey and a new child <= 5 years old reported at wave 3 who had diarrhea in past 4 weeks	-0·029 (-0·58 to 0·52)	0·92	50
Child experienced diarrhea past 4 weeks, was given appropriate treatment	Practice	All respondents with a complete survey and a new child <= 5 years old reported at wave 3 who had diarrhea in past 4 weeks	1·1 (0·60 to 1·5)	<0·0001	55
Child experienced diarrhea past 4 weeks, was given zinc 10-14 days	Practice	All respondents with a complete survey and a new child <= 5 years old reported at wave 3 who had diarrhea in past 4 weeks	..	..	..

For peer review only - http://bmjopen.bmj.com/site/about/guidelines.xhtml

Identified appropriate diarrhea treatment methods[d]	Knowledge and attitudes	All respondents with a complete survey	0·093 (0·038 to 0·15)	0·0009	9280
Identified zinc 10-14 days as diarrhea treatment	Knowledge and attitudes	All respondents with a complete survey	0·034 (0·010 to 0·057)	0·0046	9257
Identified zinc supplement as way to prevent diarrhea	Knowledge and attitudes	All respondents with a complete survey	0·070 (0·0004 to 0·14)	0·049	9282
Identified breastfeeding as way to prevent diarrhea	Knowledge and attitudes	All respondents with a complete survey	0·041 (-0·026 to 0·11)	0·23	9282
Correctly answered diarrhea treatment with zinc riddle	Intervention knowledge	All respondents with a complete survey	NA	NA	NA
**Respiratory illness**					
Did not report respiratory illness (coughing) for 2 weeks	Practice	All respondents with a complete survey	-0·021 (-0·057 to 0·014)	0·24	9283
Child did not have cough past 4 weeks	Practice	All respondents with a complete survey	-0·033 (-0·097 to 0·031)	0·31	2379
Child had cough past 4 weeks, care was sought	Practice	All respondents with a complete survey and a new child <= 5 years old reported at wave 3 who had an illness with a cough in past 4 weeks	0·0043 (-0·24 to 0·24)	0·97	196
Identified vaccination as way to prevent respiratory illness	Knowledge and attitudes	All respondents with a complete survey	-0·0075 (-0·047 to 0·032)	0·71	9282
Identified breastfeeding as way to prevent respiratory illness	Knowledge and attitudes	All respondents with a complete survey	0·027 (-0·028 to 0·081)	0·34	9282
Identified fever as d.s. for children with respiratory illness	Knowledge and attitudes	All respondents with a complete survey	0·0090 (-0·021 to 0·039)	0·56	9282
Identified difficulty breathing as d.s. for children with respiratory illness	Knowledge and attitudes	All respondents with a complete survey	0·0066 (-0·0098 to 0·023)	0·43	9282
Identified rapid breathing as d.s. for children with respiratory illness	Knowledge and attitudes	All respondents with a complete survey	0·0069 (-0·0044 to 0·018)	0·23	9282
**Reproductive health**					
Reported ever using birth control to delay or avoid pregnancy	Practice	All respondents with a complete survey	0·011 (-0·038 to 0·060)	0·67	8529
Reported currently using birth control	Practice	All respondents with a complete survey who are not pregnant and have ever used birth control	0·060 (-0·038 to 0·16)	0·23	1948
Believes woman should be at least 18 years of age to have her first child	Knowledge and attitudes	All respondents with a complete survey	0·012 (0·0029 to 0·020)	0·0087	8675
**Gender/reproductive norms**					
Birth location chosen either jointly or by woman	Practice	All respondents with a complete survey and a child born since 2018-09-01 (after the end of the intervention)	0·042 (-0·10 to 0·19)	0·57	299

For peer review only - http://bmjopen.bmj.com/site/about/guidelines.xhtml

Results based on sub-sample that completed baseline survey, N=9285 (household targeted n=2919, household not targeted n=6366). Models included covariate for the related outcome assessed at baseline survey. d.s.=Danger sign, NA=Not applicable, ORT=Oral rehydration therapy. ··Model did not converge. Results with NA could not be assessed because outcome was not included in baseline survey. Standard errors are clustered at the village level. Robust 95% CIs in parentheses.

[a] ‘Identified correct ways to provide newborn care’ outcome was coded as correct if the respondent identified holding baby skin to skin, getting their health checked by a professional, immediately putting them to the breast, or making sure their diaper is clean and dry, and did not identify giving purgante, wrapping a fajero, giving a chupon, or bathing them in water right away.
[b] ‘Identified proper cord care’ outcome was coded as correct if respondent identified applying alcohol, applying iodine, keeping cord clean/dry, or applying peroxide, and did not identify applying oil, applying coffee, applying quina water, wrapping a fajero, applying powder, burning, applying ashes, applying a clamp, or applying thiomersal.
[c] ‘Used appropriate treatment for diarrhea in past 4 weeks’ outcome was coded as correct if respondent did any of the following: antibiotic, zinc, give extra food, give extra liquid, and did not do any of the following: anti-diarrhea medication, home remedy, laxative, deworming medication, chupon, massage, stop eating foods, stop taking liquids.
[d] ‘Identified appropriate diarrhea treatment methods’ outcome was coded as correct if respondent identified any of the following: antibiotic, zinc, ORS or Litrosol, give extra food, give extra liquid, get medical attention, and did not identify any of the following: anti-diarrhea medication, laxative, deworming medication, home remedy, chupon, massage, stop giving foods, stop giving liquids, or do nothing.

For peer review only - http://bmjopen.bmj.com/site/about/guidelines.xhtml

Table S6. Pooled total effect estimates of intervention on maternal, child, and neonatal outcomes, with adjustment for baseline demographic characteristics and baseline outcome

	Outcome type	Respondent denominator	Pooled total effect estimate		N
			Effect size (95% CI)	p value	
**Prenatal care**					
Currently taking folic acid tablets (daily in past 7 days)	Practice	All women 15+ with a complete survey	-0·0013 (-0·041 to 0·039)	0·95	5492
Made a birth plan in preparation for birth	Practice	All respondents with a complete survey and a child born since 2018-09-01 (after the end of the intervention)	-0·083 (-0·22 to 0·051)	0·22	291
Saved money in preparation for birth	Practice	All respondents with a complete survey and a child born since 2018-09-01 (after the end of the intervention)	-0·0003 (-0·13 to 0·13)	1·00	295
Sold animal in preparation for birth	Practice	All respondents with a complete survey and a child born since 2018-09-01 (after the end of the intervention)	NA	NA	NA
Sought prenatal care within 12 weeks	Practice	All respondents with a complete survey and a child born since 2018-09-01 (after the end of the intervention)	0·030 (-0·11 to 0·17)	0·67	277
Identifies that women should take folic acid before pregnancy	Knowledge and attitudes	All respondents with a complete survey	0·058 (0·026 to 0·091)	0·0005	9174
Identifies that women should seek prenatal care first 12 weeks of pregnancy	Knowledge and attitudes	All respondents with a complete survey	0·031 (0·0090 to 0·054)	0·0059	9173
Identifies accompanying woman to prenatal care visits as method of support during pregnancy	Knowledge and attitudes	All respondents with a complete survey	NA	NA	NA
Identifies ensuring that woman eats well as method of support during pregnancy	Knowledge and attitudes	All respondents with a complete survey	NA	NA	NA
Identifies ensuring that woman rests 1 hour per day well as method of support during pregnancy	Knowledge and attitudes	All respondents with a complete survey	NA	NA	NA
Identifies avoiding violence as method of support during pregnancy	Knowledge and attitudes	All respondents with a complete survey	NA	NA	NA
Identifies encouraging women to take vitamins as method of support during pregnancy	Knowledge and attitudes	All respondents with a complete survey	NA	NA	NA
Identifies helping woman with house work/child care as method of support during pregnancy	Knowledge and attitudes	All respondents with a complete survey	NA	NA	NA
Identifies saving animals to sell as method of preparing for birth expenses	Knowledge and attitudes	All respondents with a complete survey	NA	NA	NA

For peer review only - http://bmjopen.bmj.com/site/about/guidelines.xhtml

Identifies knowing cost of trip to maternal clinic as method of preparing for birth expenses	Knowledge and attitudes	All respondents with a complete survey	NA	NA	NA
Identifies having a savings plan as method of preparing for birth expenses	Knowledge and attitudes	All respondents with a complete survey	NA	NA	NA
Correctly answered prenatal care knowledge riddle	Intervention knowledge	All respondents with a complete survey	NA	NA	NA
**Facility based birth**					
Gave birth in health facility	Practice	All respondents with a complete survey and a child born since 2018-09-01 (after the end of the intervention)	0·0030 (-0·13 to 0·14)	0·97	295
Believes health facility is best place to give birth	Knowledge and attitudes	All respondents with a complete survey	0·018 (-0·0034 to 0·040)	0·099	9165
**Pregnancy danger signs**					
Woman sought medical care for pregnancy danger sign	Practice	All respondents with a complete survey and a child born since 2018-09-01 (after the end of the intervention) who experienced a prenatal care danger sign	-0·26 (-0·76 to 0·24)	0·30	55
Identified bleeding as pregnancy d.s.	Knowledge and attitudes	All respondents with a complete survey	0·051 (0·012 to 0·090)	0·0099	9170
Identified seizure as pregnancy d.s.	Knowledge and attitudes	All respondents with a complete survey	0·0020 (-0·0077 to 0·012)	0·69	9161
Identified headache as pregnancy d.s.	Knowledge and attitudes	All respondents with a complete survey	0·038 (-0·0085 to 0·084)	0·11	9170
Identified ringing in ears as pregnancy d.s.	Knowledge and attitudes	All respondents with a complete survey	0·0031 (-0·0064 to 0·013)	0·52	9146
Identified dizziness as pregnancy d.s.	Knowledge and attitudes	All respondents with a complete survey	-0·0030 (-0·050 to 0·044)	0·90	9170
Identified difficulty urinating as pregnancy d.s.	Knowledge and attitudes	All respondents with a complete survey	-0·0018 (-0·0094 to 0·0059)	0·65	9146
Identified reduced or absent fetal movement as pregnancy d.s.	Knowledge and attitudes	All respondents with a complete survey	0·014 (-0·0060 to 0·034)	0·17	9146
Identified water breaking as pregnancy d.s.	Knowledge and attitudes	All respondents with a complete survey	-0·0006 (-0·0079 to 0·0066)	0·87	9146
Identified fever as pregnancy d.s.	Knowledge and attitudes	All respondents with a complete survey	0·041 (0·0031 to 0·079)	0·034	9170
Identified swelling of face/hands/feet as pregnancy d.s.	Knowledge and attitudes	All respondents with a complete survey	0·032 (0·0030 to 0·061)	0·031	9170
**Postnatal care for mother**					
Mother had health checked by professional within 3 days of birth	Practice	All respondents with a complete survey and a child born since 2018-09-01 (after the end of the intervention)	-0·035 (-0·22 to 0·15)	0·70	289

For peer review only

For peer review only - http://bmjopen.bmj.com/site/about/guidelines.xhtml

Mother had health checked by professional within 7 days of birth	Practice	All respondents with a complete survey and a child born since 2018-09-01 (after the end of the intervention)	-0·032 (-0·23 to 0·17)	0·75	289
Mother sought medical care for postnatal danger sign	Practice	All respondents with a complete survey and a child born since 2018-09-01 (after the end of the intervention)	0·38 (0·062 to 0·69)	0·019	33
Identifies that mother should receive postnatal medical check-up within 3 days of birth	Knowledge and attitudes	All respondents with a complete survey	0·072 (0·030 to 0·11)	0·0007	9170
Identifies that mother should receive postnatal medical check-up within 7 days of birth	Knowledge and attitudes	All respondents with a complete survey	0·037 (-0·011 to 0·085)	0·13	9170
Identified heavy vaginal bleeding as postnatal d.s.	Knowledge and attitudes	All respondents with a complete survey	0·066 (0·028 to 0·10)	0·0006	9170
Identified fever as postnatal d.s.	Knowledge and attitudes	All respondents with a complete survey	0·060 (0·011 to 0·11)	0·017	9170
Identified vaginal discharge as postnatal d.s.	Knowledge and attitudes	All respondents with a complete survey	0·0045 (-0·010 to 0·019)	0·55	9155
Identified headache as postnatal d.s.	Knowledge and attitudes	All respondents with a complete survey	0·029 (-0·0085 to 0·066)	0·13	9170
Identified convulsions or fits as postnatal d.s.	Knowledge and attitudes	All respondents with a complete survey	0·00 (-0·0056 to 0·0056)	0·99	8958
**Postnatal care for newborn**					
Newborn had health checked by professional within 3 days of birth	Practice	All respondents with a complete survey and a child born since 2018-09-01 (after the end of the intervention)	0·028 (-0·13 to 0·18)	0·72	293
Newborn had health checked by professional within 7 days of birth	Practice	All respondents with a complete survey and a child born since 2018-09-01 (after the end of the intervention)	0·022 (-0·16 to 0·20)	0·81	293
Newborn experienced health problem in first month, care was sought	Practice	All respondents with a complete survey and a child born since 2018-09-01 (after the end of the intervention)	-0·014 (-0·014 to -0·014)	<0·0001	23
Identified correct ways to provide newborn care[a]	Knowledge and attitudes	All respondents with a complete survey	0·023 (0·0099 to 0·037)	0·0006	9149
Identified fever as newborn d.s.	Knowledge and attitudes	All respondents with a complete survey	0·0033 (-0·043 to 0·049)	0·89	9172
Identified diarrhea as newborn d.s.	Knowledge and attitudes	All respondents with a complete survey	0·0039 (-0·036 to 0·043)	0·85	9172
Identified difficulty breathing as newborn d.s.	Knowledge and attitudes	All respondents with a complete survey	0·0097 (-0·025 to 0·045)	0·59	9148
Identified vomiting as newborn d.s.	Knowledge and attitudes	All respondents with a complete survey	0·0085 (-0·021 to 0·038)	0·58	9163
Identified poor appetite as newborn d.s.	Knowledge and attitudes	All respondents with a complete survey	-0·0049 (-0·015 to 0·0048)	0·32	9148
Identified redness/bleeding around cord as newborn d.s.	Knowledge and attitudes	All respondents with a complete survey	0·020 (-0·0008 to 0·042)	0·059	9172
Identified pus in cord as newborn d.s.	Knowledge and attitudes	All respondents with a complete survey	0·015 (-0·0014 to 0·031)	0·073	9163
Identified cold skin as newborn d.s.	Knowledge and attitudes	All respondents with a complete survey	0·0023 (-0·0069 to 0·011)	0·63	9148

For peer review only - http://bmjopen.bmj.com/site/about/guidelines.xhtml

Identified cough as newborn d.s.	Knowledge and attitudes	All respondents with a complete survey	-0·019 (-0·069 to 0·031)	0·45	9172
Identified cold as newborn d.s.	Knowledge and attitudes	All respondents with a complete survey	-0·016 (-0·067 to 0·035)	0·55	9172
Identified problems latching as newborn d.s.	Knowledge and attitudes	All respondents with a complete survey	-0·0013 (-0·015 to 0·012)	0·85	9146
Identified pneumonia as newborn d.s.	Knowledge and attitudes	All respondents with a complete survey	NA	NA	NA
Did not use harmful substances to treat cord stump	Practice	All respondents with a complete survey and a child born since 2018-09-01 (after the end of the intervention)	0·11 (-0·061 to 0·29)	0·20	285
Did not wrap fajero around newborn in first 7 days after birth	Practice	All respondents with a complete survey and a child born since 2018-09-01 (after the end of the intervention)	0·30 (0·11 to 0·48)	0·0018	290
Identified proper cord care methods[b]	Knowledge and attitudes	All respondents with a complete survey	0·094 (0·048 to 0·14)	0·0001	9172
Correctly answered proper cord care riddle	Intervention knowledge	All respondents with a complete survey	NA	NA	NA
Kept newborn wrapped first 7 days after birth	Practice	All respondents with a complete survey and a child born since 2018-09-01 (after the end of the intervention)	..	..	..
Held newborn skin-to-skin during first month after birth	Practice	All respondents with a complete survey and a child born since 2018-09-01 (after the end of the intervention)	0·051 (-0·21 to 0·31)	0·70	70
**Breastfeeding**					
Exclusively breastfed child first 6 months	Practice	All respondents with a complete survey and a child born since 2018-09-01 (after the end of the intervention)	-0·12 (-0·26 to 0·014)	0·079	294
Exclusively breastfed child first 6 months without giving chupón	Practice	All respondents with a complete survey and a child born since 2018-09-01 (after the end of the intervention)	-0·062 (-0·23 to 0·11)	0·47	295
Breastfed immediately after birth	Practice	All respondents with a complete survey and a child born since 2018-09-01 (after the end of the intervention) who reported a natural birth	-0·049 (-0·18 to 0·085)	0·47	217
Identifies that newborn should be breastfed immediately after birth	Knowledge and attitudes	All respondents with a complete survey	0·011 (-0·023 to 0·045)	0·53	9173
Identifies that newborns should only be given breast milk during first 6 months	Knowledge and attitudes	All respondents with a complete survey	0·038 (0·0075 to 0·069)	0·015	9173
Believes newborns should not be given chupón during first 6 months	Knowledge and attitudes	All respondents with a complete survey	0·16 (0·10 to 0·21)	<0·0001	9173
**Paternal involvement**					
Father accompanied mother to clinic for prenatal care visit at least once	Practice	All respondents with a complete survey and a child born since 2018-09-01 (after the end of the intervention) who received prenatal care	0·079 (-0·10 to 0·26)	0·39	272
Father accompanied mother to seek medical care for pregnancy danger sign	Practice	All respondents with a complete survey and a child born since 2018-09-01 (after the end of the intervention) who experienced a prenatal care danger sign and sought care	..	..	..

For peer review only

For peer review only - http://bmjopen.bmj.com/site/about/guidelines.xhtml

Father waited at birthplace during labor	Practice	All respondents with a complete survey and a child born since 2018-09-01 (after the end of the intervention)	-0·021 (-0·18 to 0·13)	0·79	295
Father accompanied mother to seek medical care for postnatal danger sign	Practice	All respondents with a complete survey and a child born since 2018-09-01 (after the end of the intervention) and sought care for postpartum problem	..	..	..
Father sought medical care for newborn for postnatal danger sign	Practice	All respondents with a complete survey and a child born since 2018-09-01 (after the end of the intervention) and sought care for a postnatal problem	..	..	..
Father held child	Practice	All respondents with a complete survey and a child born since 2018-09-01 (after the end of the intervention)	0·058 (-0·042 to 0·16)	0·25	265
Father played with child	Practice	All respondents with a complete survey and a child born since 2018-09-01 (after the end of the intervention)	0·077 (-0·037 to 0·19)	0·18	271
Father took child to clinic when sick	Practice	All respondents with a complete survey and a child born since 2018-09-01 (after the end of the intervention)	0·093 (-0·077 to 0·26)	0·28	271
Believes father should accompany mother to prenatal care visits	Knowledge and attitudes	All respondents with a complete survey	0·039 (0·017 to 0·061)	0·0006	9105
Believes father should wait at birth location while mother gives birth	Knowledge and attitudes	All respondents with a complete survey	0·014 (-0·0044 to 0·032)	0·14	9108
Believes father should care for children when sick	Knowledge and attitudes	All respondents with a complete survey	0·0001 (-0·0059 to 0·0062)	0·97	9149
**Diarrhea management**					
Did not report diarrhea in past 4 weeks	Practice	All respondents with a complete survey	-0·0095 (-0·025 to 0·0062)	0·24	9160
Used ORT to treat diarrhea in past 4 weeks	Practice	All respondents with a complete survey who had diarrhea in past 4 weeks	0·036 (0·036 to 0·036)	<0·0001	23
Used appropriate treatment for diarrhea in past 4 weeks[c]	Practice	All respondents with a complete survey who had diarrhea in past 4 weeks	..	..	..
Child did not experience diarrhea past 4 weeks	Practice	All respondents with a complete survey and a new child <= 5 years old reported at wave 3 who had diarrhea in past 4 weeks	-0·0093 (-0·052 to 0·034)	0·67	2346
Child experienced diarrhea past 4 weeks, was given appropriate amount of liquids	Practice	All respondents with a complete survey and a new child <= 5 years old reported at wave 3 who had diarrhea in past 4 weeks	1·9 (1·2 to 2·6)	<0·0001	43
Child experienced diarrhea past 4 weeks, was given appropriate amount of food	Practice	All respondents with a complete survey and a new child <= 5 years old reported at wave 3 who had diarrhea in past 4 weeks	0·95 (0·52 to 1·4)	<0·0001	43
Child experienced diarrhea past 4 weeks, was given ORT	Practice	All respondents with a complete survey and a new child <= 5 years old reported at wave 3 who had diarrhea in past 4 weeks	1·3 (0·73 to 1·8)	<0·0001	43
Child experienced diarrhea past 4 weeks, was given appropriate treatment	Practice	All respondents with a complete survey and a new child <= 5 years old reported at wave 3 who had diarrhea in past 4 weeks	0·59 (0·19 to 0·98)	0·0034	48
Child experienced diarrhea past 4 weeks, was given zinc 10-14 days	Practice	All respondents with a complete survey and a new child <= 5 years old reported at wave 3 who had diarrhea in past 4 weeks	..	..	..

For peer review only - http://bmjopen.bmj.com/site/about/guidelines.xhtml

Identified appropriate diarrhea treatment methods[d]	Knowledge and attitudes	All respondents with a complete survey	0·11 (0·055 to 0·16)	0·0001	9171
Identified zinc 10-14 days as diarrhea treatment	Knowledge and attitudes	All respondents with a complete survey	0·032 (0·0095 to 0·055)	0·0056	9133
Identified zinc supplement as way to prevent diarrhea	Knowledge and attitudes	All respondents with a complete survey	0·073 (0·0071 to 0·14)	0·030	9173
Identified breastfeeding as way to prevent diarrhea	Knowledge and attitudes	All respondents with a complete survey	0·039 (-0·021 to 0·100)	0·20	9173
Correctly answered diarrhea treatment with zinc riddle	Intervention knowledge	All respondents with a complete survey	NA	NA	NA
**Respiratory illness**					
Did not report respiratory illness (coughing) for 2 weeks	Practice	All respondents with a complete survey	-0·017 (-0·050 to 0·017)	0·33	9167
Child did not have cough past 4 weeks	Practice	All respondents with a complete survey	-0·038 (-0·10 to 0·026)	0·25	2348
Child had cough past 4 weeks, care was sought	Practice	All respondents with a complete survey and a new child <= 5 years old reported at wave 3 who had an illness with a cough in past 4 weeks	-0·0071 (-0·24 to 0·23)	0·95	195
Identified vaccination as way to prevent respiratory illness	Knowledge and attitudes	All respondents with a complete survey	-0·0012 (-0·039 to 0·036)	0·95	9173
Identified breastfeeding as way to prevent respiratory illness	Knowledge and attitudes	All respondents with a complete survey	0·031 (-0·020 to 0·081)	0·24	9173
Identified fever as d.s. for children with respiratory illness	Knowledge and attitudes	All respondents with a complete survey	0·016 (-0·012 to 0·043)	0·26	9164
Identified difficulty breathing as d.s. for children with respiratory illness	Knowledge and attitudes	All respondents with a complete survey	0·011 (-0·0047 to 0·027)	0·17	9164
Identified rapid breathing as d.s. for children with respiratory illness	Knowledge and attitudes	All respondents with a complete survey	0·0084 (-0·0023 to 0·019)	0·12	9164
**Reproductive health**					
Reported ever using birth control to delay or avoid pregnancy	Practice	All respondents with a complete survey	0·0068 (-0·040 to 0·054)	0·78	8433
Reported currently using birth control	Practice	All respondents with a complete survey who are not pregnant and have ever used birth control	0·051 (-0·039 to 0·14)	0·27	1926
Believes woman should be at least 18 years of age to have her first child	Knowledge and attitudes	All respondents with a complete survey	0·012 (0·0046 to 0·019)	0·0015	8551
**Gender/reproductive norms**					
Birth location chosen either jointly or by woman	Practice	All respondents with a complete survey and a child born since 2018-09-01 (after the end of the intervention)	0·046 (-0·086 to 0·18)	0·49	291

Results based on sub-sample that completed baseline survey, N=9285 (household targeted n=2919, household not targeted n=6366). Covariates included baseline age, gender, education, marital status, indigenous status, household wealth index, and the related outcome. d.s.=Danger sign, NA=Not applicable, ORT=Oral

For peer review only - http://bmjopen.bmj.com/site/about/guidelines.xhtml

rehydration therapy. ··Model did not converge. Results with NA could not be assessed because outcome was not included in baseline survey. Covariates were dropped sequentially if they predicted outcome perfectly. Standard errors are clustered at the village level. Robust 95% CIs in parentheses.

[a] 'Identified correct ways to provide newborn care' outcome was coded as correct if the respondent identified holding baby skin to skin, getting their health checked by a professional, immediately putting them to the breast, or making sure their diaper is clean and dry, and did not identify giving purgante, wrapping a fajero, giving a chupon, or bathing them in water right away.
[b] 'Identified proper cord care' outcome was coded as correct if respondent identified applying alcohol, applying iodine, keeping cord clean/dry, or applying peroxide, and did not identify applying oil, applying coffee, applying quina water, wrapping a fajero, applying powder, burning, applying ashes, applying a clamp, or applying thiomersal.
[c] 'Used appropriate treatment for diarrhea in past 4 weeks' outcome was coded as correct if respondent did any of the following: antibiotic, zinc, give extra food, give extra liquid, and did not do any of the following: anti-diarrhea medication, home remedy, laxative, deworming medication, chupon, massage, stop eating foods, stop taking liquids.
[d] 'Identified appropriate diarrhea treatment methods' outcome was coded as correct if respondent identified any of the following: antibiotic, zinc, ORS or Litrosol, give extra food, give extra liquid, get medical attention, and did not identify any of the following: anti-diarrhea medication, laxative, deworming medication, home remedy, chupon, massage, stop giving foods, stop giving liquids, or do nothing.

For peer review only

Table S7. Pooled total effect estimates of intervention on maternal, child, and neonatal outcomes, with adjustment for baseline demographic characteristics and baseline outcome, estimated on sub-sample of respondents who did not move between villages during study

	Outcome type	Respondent denominator	Pooled total effect estimate		N
			Effect size (95% CI)	p value	
**Prenatal care**					
Currently taking folic acid tablets (daily in past 7 days)	Practice	All women 15+ with a complete survey	0·0014 (-0·040 to 0·043)	0·95	5284
Made a birth plan in preparation for birth	Practice	All respondents with a complete survey and a child born since 2018-09-01 (after the end of the intervention)	-0·082 (-0·22 to 0·055)	0·24	278
Saved money in preparation for birth	Practice	All respondents with a complete survey and a child born since 2018-09-01 (after the end of the intervention)	-0·0050 (-0·14 to 0·13)	0·94	281
Sold animal in preparation for birth	Practice	All respondents with a complete survey and a child born since 2018-09-01 (after the end of the intervention)	NA	NA	NA
Sought prenatal care within 12 weeks	Practice	All respondents with a complete survey and a child born since 2018-09-01 (after the end of the intervention)	0·033 (-0·11 to 0·17)	0·64	264
Identifies that women should take folic acid before pregnancy	Knowledge and attitudes	All respondents with a complete survey	0·058 (0·026 to 0·090)	0·0004	8875
Identifies that women should seek prenatal care first 12 weeks of pregnancy	Knowledge and attitudes	All respondents with a complete survey	0·030 (0·0068 to 0·052)	0·011	8875
Identifies accompanying woman to prenatal care visits as method of support during pregnancy	Knowledge and attitudes	All respondents with a complete survey	NA	NA	NA
Identifies ensuring that woman eats well as method of support during pregnancy	Knowledge and attitudes	All respondents with a complete survey	NA	NA	NA
Identifies ensuring that woman rests 1 hour per day well as method of support during pregnancy	Knowledge and attitudes	All respondents with a complete survey	NA	NA	NA
Identifies avoiding violence as method of support during pregnancy	Knowledge and attitudes	All respondents with a complete survey	NA	NA	NA
Identifies encouraging women to take vitamins as method of support during pregnancy	Knowledge and attitudes	All respondents with a complete survey	NA	NA	NA
Identifies helping woman with house work/child care as method of support during pregnancy	Knowledge and attitudes	All respondents with a complete survey	NA	NA	NA
Identifies saving animals to sell as method of preparing for birth expenses	Knowledge and attitudes	All respondents with a complete survey	NA	NA	NA

For peer review only - http://bmjopen.bmj.com/site/about/guidelines.xhtml

Identifies knowing cost of trip to maternal clinic as method of preparing for birth expenses	Knowledge and attitudes	All respondents with a complete survey	NA	NA	NA
Identifies having a savings plan as method of preparing for birth expenses	Knowledge and attitudes	All respondents with a complete survey	NA	NA	NA
Correctly answered prenatal care knowledge riddle	Intervention knowledge	All respondents with a complete survey	NA	NA	NA
**Facility based birth**					
Gave birth in health facility	Practice	All respondents with a complete survey and a child born since 2018-09-01 (after the end of the intervention)	0·029 (-0·10 to 0·16)	0·66	281
Believes health facility is best place to give birth	Knowledge and attitudes	All respondents with a complete survey	0·017 (-0·0050 to 0·039)	0·13	8866
**Pregnancy danger signs**					
Woman sought medical care for pregnancy danger sign	Practice	All respondents with a complete survey and a child born since 2018-09-01 (after the end of the intervention) who experienced a prenatal care danger sign	-0·29 (-0·82 to 0·24)	0·29	51
Identified bleeding as pregnancy d.s.	Knowledge and attitudes	All respondents with a complete survey	0·054 (0·013 to 0·095)	0·010	8872
Identified seizure as pregnancy d.s.	Knowledge and attitudes	All respondents with a complete survey	0·0022 (-0·0078 to 0·012)	0·67	8863
Identified headache as pregnancy d.s.	Knowledge and attitudes	All respondents with a complete survey	0·042 (-0·0059 to 0·090)	0·086	8872
Identified ringing in ears as pregnancy d.s.	Knowledge and attitudes	All respondents with a complete survey	0·0025 (-0·0073 to 0·012)	0·62	8848
Identified dizziness as pregnancy d.s.	Knowledge and attitudes	All respondents with a complete survey	-0·0061 (-0·055 to 0·043)	0·81	8872
Identified difficulty urinating as pregnancy d.s.	Knowledge and attitudes	All respondents with a complete survey	-0·0023 (-0·010 to 0·0055)	0·57	8848
Identified reduced or absent fetal movement as pregnancy d.s.	Knowledge and attitudes	All respondents with a complete survey	0·014 (-0·0064 to 0·034)	0·18	8848
Identified water breaking as pregnancy d.s.	Knowledge and attitudes	All respondents with a complete survey	-0·0031 (-0·0098 to 0·0035)	0·36	8848
Identified fever as pregnancy d.s.	Knowledge and attitudes	All respondents with a complete survey	0·039 (0·0002 to 0·077)	0·049	8872
Identified swelling of face/hands/feet as pregnancy d.s.	Knowledge and attitudes	All respondents with a complete survey	0·032 (0·0017 to 0·063)	0·038	8872
**Postnatal care for mother**					
Mother had health checked by professional within 3 days of birth	Practice	All respondents with a complete survey and a child born since 2018-09-01 (after the end of the intervention)	-0·058 (-0·24 to 0·13)	0·54	275

For peer review only - http://bmjopen.bmj.com/site/about/guidelines.xhtml

Mother had health checked by professional within 7 days of birth	Practice	All respondents with a complete survey and a child born since 2018-09-01 (after the end of the intervention)	-0·060 (-0·27 to 0·15)	0·57	272
Mother sought medical care for postnatal danger sign	Practice	All respondents with a complete survey and a child born since 2018-09-01 (after the end of the intervention)	0·32 (-0·015 to 0·66)	0·061	29
Identifies that mother should receive postnatal medical check-up within 3 days of birth	Knowledge and attitudes	All respondents with a complete survey	0·071 (0·028 to 0·11)	0·0011	8872
Identifies that mother should receive postnatal medical check-up within 7 days of birth	Knowledge and attitudes	All respondents with a complete survey	0·039 (-0·012 to 0·090)	0·13	8872
Identified heavy vaginal bleeding as postnatal d.s.	Knowledge and attitudes	All respondents with a complete survey	0·070 (0·032 to 0·11)	0·0003	8871
Identified fever as postnatal d.s.	Knowledge and attitudes	All respondents with a complete survey	0·057 (0·0076 to 0·11)	0·024	8871
Identified vaginal discharge as postnatal d.s.	Knowledge and attitudes	All respondents with a complete survey	0·0035 (-0·011 to 0·018)	0·62	8856
Identified headache as postnatal d.s.	Knowledge and attitudes	All respondents with a complete survey	0·031 (-0·0079 to 0·069)	0·12	8871
Identified convulsions or fits as postnatal d.s.	Knowledge and attitudes	All respondents with a complete survey	0·0004 (-0·0054 to 0·0063)	0·88	8673
**Postnatal care for newborn**					
Newborn had health checked by professional within 3 days of birth	Practice	All respondents with a complete survey and a child born since 2018-09-01 (after the end of the intervention)	0·0038 (-0·15 to 0·16)	0·96	279
Newborn had health checked by professional within 7 days of birth	Practice	All respondents with a complete survey and a child born since 2018-09-01 (after the end of the intervention)	-0·013 (-0·20 to 0·17)	0·89	276
Newborn experienced health problem in first month, care was sought	Practice	All respondents with a complete survey and a child born since 2018-09-01 (after the end of the intervention)	-0·011 (-0·011 to -0·011)	<0·0001	21
Identified correct ways to provide newborn care[a]	Knowledge and attitudes	All respondents with a complete survey	0·022 (0·0089 to 0·035)	0·0010	8851
Identified fever as newborn d.s.	Knowledge and attitudes	All respondents with a complete survey	0·0037 (-0·044 to 0·051)	0·88	8873
Identified diarrhea as newborn d.s.	Knowledge and attitudes	All respondents with a complete survey	0·0081 (-0·034 to 0·050)	0·71	8873
Identified difficulty breathing as newborn d.s.	Knowledge and attitudes	All respondents with a complete survey	0·0061 (-0·030 to 0·042)	0·74	8849
Identified vomiting as newborn d.s.	Knowledge and attitudes	All respondents with a complete survey	0·0089 (-0·021 to 0·039)	0·56	8864
Identified poor appetite as newborn d.s.	Knowledge and attitudes	All respondents with a complete survey	-0·0045 (-0·015 to 0·0054)	0·37	8849
Identified redness/bleeding around cord as newborn d.s.	Knowledge and attitudes	All respondents with a complete survey	0·021 (-0·0012 to 0·043)	0·064	8873
Identified pus in cord as newborn d.s.	Knowledge and attitudes	All respondents with a complete survey	0·015 (-0·0020 to 0·031)	0·085	8864
Identified cold skin as newborn d.s.	Knowledge and attitudes	All respondents with a complete survey	0·0024 (-0·0069 to 0·012)	0·61	8849

For peer review only

For peer review only - http://bmjopen.bmj.com/site/about/guidelines.xhtml

Identified cough as newborn d.s.	Knowledge and attitudes	All respondents with a complete survey	-0·020 (-0·070 to 0·031)	0·45	8873
Identified cold as newborn d.s.	Knowledge and attitudes	All respondents with a complete survey	-0·014 (-0·066 to 0·038)	0·61	8873
Identified problems latching as newborn d.s.	Knowledge and attitudes	All respondents with a complete survey	0·0001 (-0·014 to 0·014)	0·99	8847
Identified pneumonia as newborn d.s.	Knowledge and attitudes	All respondents with a complete survey	NA	NA	NA
Did not use harmful substances to treat cord stump	Practice	All respondents with a complete survey and a child born since 2018-09-01 (after the end of the intervention)	0·11 (-0·068 to 0·29)	0·23	271
Did not wrap fajero around newborn in first 7 days after birth	Practice	All respondents with a complete survey and a child born since 2018-09-01 (after the end of the intervention)	0·29 (0·099 to 0·48)	0·0029	277
Identified proper cord care methods[b]	Knowledge and attitudes	All respondents with a complete survey	0·10 (0·053 to 0·15)	<0·0001	8874
Correctly answered proper cord care riddle	Intervention knowledge	All respondents with a complete survey	NA	NA	NA
Kept newborn wrapped first 7 days after birth	Practice	All respondents with a complete survey and a child born since 2018-09-01 (after the end of the intervention)	··	··	··
Held newborn skin-to-skin during first month after birth	Practice	All respondents with a complete survey and a child born since 2018-09-01 (after the end of the intervention)	0·051 (-0·21 to 0·31)	0·70	68
**Breastfeeding**					
Exclusively breastfed child first 6 months	Practice	All respondents with a complete survey and a child born since 2018-09-01 (after the end of the intervention)	-0·12 (-0·26 to 0·012)	0·073	280
Exclusively breastfed child first 6 months without giving chupón	Practice	All respondents with a complete survey and a child born since 2018-09-01 (after the end of the intervention)	-0·060 (-0·24 to 0·12)	0·51	281
Breastfed immediately after birth	Practice	All respondents with a complete survey and a child born since 2018-09-01 (after the end of the intervention) who reported a natural birth	-0·0011 (-0·12 to 0·11)	0·98	210
Identifies that newborn should be breastfed immediately after birth	Knowledge and attitudes	All respondents with a complete survey	0·016 (-0·019 to 0·050)	0·38	8875
Identifies that newborns should only be given breast milk during first 6 months	Knowledge and attitudes	All respondents with a complete survey	0·039 (0·0080 to 0·069)	0·014	8874
Believes newborns should not be given chupón during first 6 months	Knowledge and attitudes	All respondents with a complete survey	0·16 (0·10 to 0·22)	<0·0001	8875
**Paternal involvement**					
Father accompanied mother to clinic for prenatal care visit at least once	Practice	All respondents with a complete survey and a child born since 2018-09-01 (after the end of the intervention) who received prenatal care	0·074 (-0·11 to 0·26)	0·44	259
Father accompanied mother to seek medical care for pregnancy danger sign	Practice	All respondents with a complete survey and a child born since 2018-09-01 (after the end of the intervention) who experienced a prenatal care danger sign and sought care	··	··	··

For peer review only - http://bmjopen.bmj.com/site/about/guidelines.xhtml

Father waited at birthplace during labor	Practice	All respondents with a complete survey and a child born since 2018-09-01 (after the end of the intervention)	-0·031 (-0·19 to 0·13)	0·70	281
Father accompanied mother to seek medical care for postnatal danger sign	Practice	All respondents with a complete survey and a child born since 2018-09-01 (after the end of the intervention) and sought care for postpartum problem	..	..	..
Father sought medical care for newborn for postnatal danger sign	Practice	All respondents with a complete survey and a child born since 2018-09-01 (after the end of the intervention) and sought care for a postnatal problem	..	..	..
Father held child	Practice	All respondents with a complete survey and a child born since 2018-09-01 (after the end of the intervention)	0·057 (-0·046 to 0·16)	0·28	258
Father played with child	Practice	All respondents with a complete survey and a child born since 2018-09-01 (after the end of the intervention)	0·073 (-0·043 to 0·19)	0·22	262
Father took child to clinic when sick	Practice	All respondents with a complete survey and a child born since 2018-09-01 (after the end of the intervention)	0·089 (-0·086 to 0·26)	0·32	262
Believes father should accompany mother to prenatal care visits	Knowledge and attitudes	All respondents with a complete survey	0·039 (0·017 to 0·061)	0·0006	8807
Believes father should wait at birth location while mother gives birth	Knowledge and attitudes	All respondents with a complete survey	0·013 (-0·0057 to 0·032)	0·17	8810
Believes father should care for children when sick	Knowledge and attitudes	All respondents with a complete survey	0·0004 (-0·0058 to 0·0065)	0·90	8851
**Diarrhea management**					
Did not report diarrhea in past 4 weeks	Practice	All respondents with a complete survey	-0·0095 (-0·026 to 0·0068)	0·26	8861
Used ORT to treat diarrhea in past 4 weeks	Practice	All respondents with a complete survey who had diarrhea in past 4 weeks	..	..	..
Used appropriate treatment for diarrhea in past 4 weeks[c]	Practice	All respondents with a complete survey who had diarrhea in past 4 weeks	..	..	..
Child did not experience diarrhea past 4 weeks	Practice	All respondents with a complete survey and a new child <= 5 years old reported at wave 3 who had diarrhea in past 4 weeks	-0·0094 (-0·054 to 0·035)	0·68	2257
Child experienced diarrhea past 4 weeks, was given appropriate amount of liquids	Practice	All respondents with a complete survey and a new child <= 5 years old reported at wave 3 who had diarrhea in past 4 weeks	1·9 (1·4 to 2·4)	<0·0001	41
Child experienced diarrhea past 4 weeks, was given appropriate amount of food	Practice	All respondents with a complete survey and a new child <= 5 years old reported at wave 3 who had diarrhea in past 4 weeks	1·2 (0·74 to 1·6)	<0·0001	36
Child experienced diarrhea past 4 weeks, was given ORT	Practice	All respondents with a complete survey and a new child <= 5 years old reported at wave 3 who had diarrhea in past 4 weeks	1·0 (0·62 to 1·4)	<0·0001	41
Child experienced diarrhea past 4 weeks, was given appropriate treatment	Practice	All respondents with a complete survey and a new child <= 5 years old reported at wave 3 who had diarrhea in past 4 weeks	0·58 (0·16 to 1·0)	0·0071	46
Child experienced diarrhea past 4 weeks, was given zinc 10-14 days	Practice	All respondents with a complete survey and a new child <= 5 years old reported at wave 3 who had diarrhea in past 4 weeks	..	..	..
Identified appropriate diarrhea treatment methods[d]	Knowledge and attitudes	All respondents with a complete survey	0·10 (0·051 to 0·16)	0·0001	8873
Identified zinc 10-14 days as diarrhea treatment	Knowledge and attitudes	All respondents with a complete survey	0·033 (0·0091 to 0·057)	0·0068	8835

For peer review only - http://bmjopen.bmj.com/site/about/guidelines.xhtml

Identified zinc supplement as way to prevent diarrhea	Knowledge and attitudes	All respondents with a complete survey	0·071 (0·0039 to 0·14)	0·038	8875
Identified breastfeeding as way to prevent diarrhea	Knowledge and attitudes	All respondents with a complete survey	0·038 (-0·024 to 0·099)	0·23	8875
Correctly answered diarrhea treatment with zinc riddle	Intervention knowledge	All respondents with a complete survey	NA	NA	NA
**Respiratory illness**					
Did not report respiratory illness (coughing) for 2 weeks	Practice	All respondents with a complete survey	-0·015 (-0·048 to 0·019)	0·39	8868
Child did not have cough past 4 weeks	Practice	All respondents with a complete survey	-0·044 (-0·11 to 0·022)	0·19	2257
Child had cough past 4 weeks, care was sought	Practice	All respondents with a complete survey and a new child <= 5 years old reported at wave 3 who had an illness with a cough in past 4 weeks	0·0005 (-0·24 to 0·24)	1·00	187
Identified vaccination as way to prevent respiratory illness	Knowledge and attitudes	All respondents with a complete survey	-0·0026 (-0·040 to 0·035)	0·89	8875
Identified breastfeeding as way to prevent respiratory illness	Knowledge and attitudes	All respondents with a complete survey	0·029 (-0·023 to 0·081)	0·27	8875
Identified fever as d.s. for children with respiratory illness	Knowledge and attitudes	All respondents with a complete survey	0·017 (-0·011 to 0·044)	0·23	8866
Identified difficulty breathing as d.s. for children with respiratory illness	Knowledge and attitudes	All respondents with a complete survey	0·011 (-0·0048 to 0·028)	0·17	8866
Identified rapid breathing as d.s. for children with respiratory illness	Knowledge and attitudes	All respondents with a complete survey	0·0086 (-0·0022 to 0·019)	0·12	8866
**Reproductive health**					
Reported ever using birth control to delay or avoid pregnancy	Practice	All respondents with a complete survey	0·0035 (-0·045 to 0·052)	0·89	8172
Reported currently using birth control	Practice	All respondents with a complete survey who are not pregnant and have ever used birth control	0·058 (-0·035 to 0·15)	0·22	1868
Believes woman should be at least 18 years of age to have her first child	Knowledge and attitudes	All respondents with a complete survey	0·012 (0·0043 to 0·019)	0·0018	8276
**Gender/reproductive norms**					
Birth location chosen either jointly or by woman	Practice	All respondents with a complete survey and a child born since 2018-09-01 (after the end of the intervention)	0·063 (-0·067 to 0·19)	0·34	277

Results based on sub-sample of respondents who didn't move villages during the study who also completed baseline survey, N=8981 (household targeted n=2831, household not targeted n=6150). Covariates included baseline age, gender, education, marital status, indigenous status, household wealth index, and the related outcome. d.s.=Danger sign, NA=Not applicable, ORT=Oral rehydration therapy. ··Model did not converge. Results with NA could not be assessed

For peer review only - http://bmjopen.bmj.com/site/about/guidelines.xhtml

because outcome was not included in baseline survey. Covariates were dropped sequentially if they predicted outcome perfectly. Standard errors are clustered at the village level. Robust 95% CIs in parentheses.

[a] 'Identified correct ways to provide newborn care' outcome was coded as correct if the respondent identified holding baby skin to skin, getting their health checked by a professional, immediately putting them to the breast, or making sure their diaper is clean and dry, and did not identify giving purgante, wrapping a fajero, giving a chupon, or bathing them in water right away.
[b] 'Identified proper cord care' outcome was coded as correct if respondent identified applying alcohol, applying iodine, keeping cord clean/dry, or applying peroxide, and did not identify applying oil, applying coffee, applying quina water, wrapping a fajero, applying powder, burning, applying ashes, applying a clamp, or applying thiomersal.
[c] 'Used appropriate treatment for diarrhea in past 4 weeks' outcome was coded as correct if respondent did any of the following: antibiotic, zinc, give extra food, give extra liquid, and did not do any of the following: anti-diarrhea medication, home remedy, laxative, deworming medication, chupon, massage, stop eating foods, stop taking liquids.
[d] 'Identified appropriate diarrhea treatment methods' outcome was coded as correct if respondent identified any of the following: antibiotic, zinc, ORS or Litrosol, give extra food, give extra liquid, get medical attention, and did not identify any of the following: anti-diarrhea medication, laxative, deworming medication, home remedy, chupon, massage, stop giving foods, stop giving liquids, or do nothing.

For peer review only - http://bmjopen.bmj.com/site/about/guidelines.xhtml

Table S8. Pooled total effect estimates of intervention on maternal, child, and neonatal outcomes, intervention module receipt defined as treatment, with adjustment for baseline demographic characteristics

	Outcome type	Respondent denominator	Intervention module(s)	Pooled total effect estimate		N
				Effect size (95% CI)	p value	
**Prenatal care**						
Currently taking folic acid tablets (daily in past 7 days)	Practice	All women 15+ with a complete survey	m11	0·017 (-0·035 to 0·070)	0·52	5922
Made a birth plan in preparation for birth	Practice	All respondents with a complete survey and a child born since 2018-09-01 (after the end of the intervention)	m2	0·021 (-0·12 to 0·16)	0·77	536
Saved money in preparation for birth	Practice	All respondents with a complete survey and a child born since 2018-09-01 (after the end of the intervention)	m1	-0·061 (-0·19 to 0·073)	0·37	540
Sold animal in preparation for birth	Practice	All respondents with a complete survey and a child born since 2018-09-01 (after the end of the intervention)	m1	-0·0070 (-0·090 to 0·076)	0·87	535
Sought prenatal care within 12 weeks	Practice	All respondents with a complete survey and a child born since 2018-09-01 (after the end of the intervention)	m1	-0·037 (-0·20 to 0·13)	0·67	518
Identifies that women should take folic acid before pregnancy	Knowledge and attitudes	All respondents with a complete survey	m11	0·097 (0·063 to 0·13)	<0·0001	9197
Identifies that women should seek prenatal care first 12 weeks of pregnancy	Knowledge and attitudes	All respondents with a complete survey	m1	0·060 (0·040 to 0·081)	<0·0001	9196
Identifies accompanying woman to prenatal care visits as method of support during pregnancy	Knowledge and attitudes	All respondents with a complete survey	m1	0·0012 (-0·057 to 0·060)	0·97	9192
Identifies ensuring that woman eats well as method of support during pregnancy	Knowledge and attitudes	All respondents with a complete survey	m1	-0·022 (-0·084 to 0·039)	0·48	9192
Identifies ensuring that woman rests 1 hour per day well as method of support during pregnancy	Knowledge and attitudes	All respondents with a complete survey	m1	0·0053 (-0·038 to 0·048)	0·81	9183
Identifies avoiding violence as method of support during pregnancy	Knowledge and attitudes	All respondents with a complete survey	m1	-0·032 (-0·054 to -0·0097)	0·0049	9192
Identifies encouraging women to take vitamins as method of support during pregnancy	Knowledge and attitudes	All respondents with a complete survey	m1	-0·038 (-0·074 to -0·0016)	0·041	9192

For peer review only - http://bmjopen.bmj.com/site/about/guidelines.xhtml

Identifies helping woman with house work/child care as method of support during pregnancy	Knowledge and attitudes	All respondents with a complete survey	m1	0·099 (0·032 to 0·17)	0·0037	9192
Identifies saving animals to sell as method of preparing for birth expenses	Knowledge and attitudes	All respondents with a complete survey	m1	0·026 (-0·0042 to 0·057)	0·091	9187
Identifies knowing cost of trip to maternal clinic as method of preparing for birth expenses	Knowledge and attitudes	All respondents with a complete survey	m1	0·0057 (-0·036 to 0·047)	0·79	9196
Identifies having a savings plan as method of preparing for birth expenses	Knowledge and attitudes	All respondents with a complete survey	m1	0·059 (0·036 to 0·083)	<0·0001	9172
Correctly answered prenatal care knowledge riddle	Intervention knowledge	All respondents with a complete survey	m1	0·12 (0·079 to 0·17)	<0·0001	9196
**Facility based birth**						
Gave birth in health facility	Practice	All respondents with a complete survey and a child born since 2018-09-01 (after the end of the intervention)	m3	-0·039 (-0·18 to 0·10)	0·58	540
Believes health facility is best place to give birth	Knowledge and attitudes	All respondents with a complete survey	m3	0·034 (0·0069 to 0·061)	0·014	9188
**Pregnancy danger signs**						
Woman sought medical care for pregnancy danger sign	Practice	All respondents with a complete survey and a child born since 2018-09-01 (after the end of the intervention) who experienced a prenatal care danger sign	m1	0·025 (-0·22 to 0·27)	0·84	209
Identified bleeding as pregnancy d.s.	Knowledge and attitudes	All respondents with a complete survey	m1	0·070 (0·023 to 0·12)	0·0035	9195
Identified seizure as pregnancy d.s.	Knowledge and attitudes	All respondents with a complete survey	m1	-0·0032 (-0·013 to 0·0066)	0·53	9186
Identified headache as pregnancy d.s.	Knowledge and attitudes	All respondents with a complete survey	m1	0·059 (0·0056 to 0·11)	0·030	9195
Identified ringing in ears as pregnancy d.s.	Knowledge and attitudes	All respondents with a complete survey	m1	0·0044 (-0·0074 to 0·016)	0·46	9171
Identified dizziness as pregnancy d.s.	Knowledge and attitudes	All respondents with a complete survey	m1	-0·025 (-0·085 to 0·036)	0·43	9195
Identified difficulty urinating as pregnancy d.s.	Knowledge and attitudes	All respondents with a complete survey	m1	-0·0066 (-0·014 to 0·0008)	0·079	9171
Identified reduced or absent fetal movement as pregnancy d.s.	Knowledge and attitudes	All respondents with a complete survey	m1	0·032 (0·0006 to 0·062)	0·046	9171
Identified water breaking as pregnancy d.s.	Knowledge and attitudes	All respondents with a complete survey	m1	0·0017 (-0·0078 to 0·011)	0·72	9171
Identified fever as pregnancy d.s.	Knowledge and attitudes	All respondents with a complete survey	m1	0·066 (0·015 to 0·12)	0·012	9195

For peer review only - http://bmjopen.bmj.com/site/about/guidelines.xhtml

Identified swelling of face/hands/feet as pregnancy d.s.	Knowledge and attitudes	All respondents with a complete survey	m1	0·064 (0·022 to 0·11)	0·0028	9195
**Postnatal care for mother**						
Mother had health checked by professional within 3 days of birth	Practice	All respondents with a complete survey and a child born since 2018-09-01 (after the end of the intervention)	m5, m6	0·22 (0·034 to 0·40)	0·020	530
Mother had health checked by professional within 7 days of birth	Practice	All respondents with a complete survey and a child born since 2018-09-01 (after the end of the intervention)	m5, m6	0·15 (-0·0047 to 0·31)	0·057	530
Mother sought medical care for postnatal danger sign	Practice	All respondents with a complete survey and a child born since 2018-09-01 (after the end of the intervention)	m5, m6	0·20 (-0·027 to 0·42)	0·084	178
Identifies that mother should receive postnatal medical check-up within 3 days of birth	Knowledge and attitudes	All respondents with a complete survey	m5, m6	0·13 (0·075 to 0·18)	<0·0001	9196
Identifies that mother should receive postnatal medical check-up within 7 days of birth	Knowledge and attitudes	All respondents with a complete survey	m5, m6	0·100 (0·048 to 0·15)	0·00010	9196
Identified heavy vaginal bleeding as postnatal d.s.	Knowledge and attitudes	All respondents with a complete survey	m5, m6	0·12 (0·068 to 0·16)	<0·0001	9195
Identified fever as postnatal d.s.	Knowledge and attitudes	All respondents with a complete survey	m5, m6	0·095 (0·036 to 0·15)	0·0016	9195
Identified vaginal discharge as postnatal d.s.	Knowledge and attitudes	All respondents with a complete survey	m5, m6	0·0062 (-0·014 to 0·026)	0·54	9180
Identified headache as postnatal d.s.	Knowledge and attitudes	All respondents with a complete survey	m5, m6	0·018 (-0·026 to 0·061)	0·42	9195
Identified convulsions or fits as postnatal d.s.	Knowledge and attitudes	All respondents with a complete survey	m5, m6	0·0050 (-0·0054 to 0·016)	0·35	8983
**Postnatal care for newborn**						
Newborn had health checked by professional within 3 days of birth	Practice	All respondents with a complete survey and a child born since 2018-09-01 (after the end of the intervention)	m5, m6	0·30 (0·13 to 0·47)	0·0006	532
Newborn had health checked by professional within 7 days of birth	Practice	All respondents with a complete survey and a child born since 2018-09-01 (after the end of the intervention)	m5, m6	0·22 (0·064 to 0·37)	0·0055	532
Newborn experienced health problem in first month, care was sought	Practice	All respondents with a complete survey and a child born since 2018-09-01 (after the end of the intervention)	m5, m6	0·25 (0·049 to 0·45)	0·015	154
Identified correct ways to provide newborn care[a]	Knowledge and attitudes	All respondents with a complete survey	m5, m6, m7	0·029 (0·010 to 0·047)	0·0021	9172
Identified fever as newborn d.s.	Knowledge and attitudes	All respondents with a complete survey	m7	-0·0012 (-0·054 to 0·052)	0·96	9195

For peer review only

For peer review only - http://bmjopen.bmj.com/site/about/guidelines.xhtml

Identified diarrhea as newborn d.s.	Knowledge and attitudes	All respondents with a complete survey	m7	0·0025 (-0·049 to 0·054)	0·92	9195
Identified difficulty breathing as newborn d.s.	Knowledge and attitudes	All respondents with a complete survey	m7	0·028 (-0·013 to 0·070)	0·18	9171
Identified vomiting as newborn d.s.	Knowledge and attitudes	All respondents with a complete survey	m7	0·023 (-0·018 to 0·064)	0·27	9186
Identified poor appetite as newborn d.s.	Knowledge and attitudes	All respondents with a complete survey	m7	0·0060 (-0·013 to 0·025)	0·54	9171
Identified redness/bleeding around cord as newborn d.s.	Knowledge and attitudes	All respondents with a complete survey	m7	0·032 (0·0034 to 0·060)	0·028	9195
Identified pus in cord as newborn d.s.	Knowledge and attitudes	All respondents with a complete survey	m7	0·032 (0·0051 to 0·060)	0·020	9186
Identified cold skin as newborn d.s.	Knowledge and attitudes	All respondents with a complete survey	m7	0·012 (-0·0079 to 0·033)	0·23	9171
Identified cough as newborn d.s.	Knowledge and attitudes	All respondents with a complete survey	m7	-0·033 (-0·087 to 0·021)	0·23	9195
Identified cold as newborn d.s.	Knowledge and attitudes	All respondents with a complete survey	m7	-0·029 (-0·090 to 0·031)	0·34	9195
Identified problems latching as newborn d.s.	Knowledge and attitudes	All respondents with a complete survey	m7	0·0028 (-0·016 to 0·021)	0·77	9171
Identified pneumonia as newborn d.s.	Knowledge and attitudes	All respondents with a complete survey	m7	0·028 (-0·016 to 0·072)	0·22	9171
Did not use harmful substances to treat cord stump	Practice	All respondents with a complete survey and a child born since 2018-09-01 (after the end of the intervention)	m5, m6, m7	0·11 (-0·045 to 0·26)	0·17	527
Did not wrap fajero around newborn in first 7 days after birth	Practice	All respondents with a complete survey and a child born since 2018-09-01 (after the end of the intervention)	m5, m6, m7	0·30 (0·13 to 0·47)	0·0005	535
Identified proper cord care methods[b]	Knowledge and attitudes	All respondents with a complete survey	m5, m6, m7	0·16 (0·10 to 0·22)	<0·0001	9195
Correctly answered proper cord care riddle	Intervention knowledge	All respondents with a complete survey	m5, m6, m7	0·30 (0·25 to 0·34)	<0·0001	9197
Kept newborn wrapped first 7 days after birth	Practice	All respondents with a complete survey and a child born since 2018-09-01 (after the end of the intervention)	m5, m6, m7	-0·0064 (-0·059 to 0·047)	0·81	530
Held newborn skin-to-skin during first month after birth	Practice	All respondents with a complete survey and a child born since 2018-09-01 (after the end of the intervention)	m5, m6, m7	0·22 (0·081 to 0·36)	0·0020	537
**Breastfeeding**						
Exclusively breastfed child first 6 months	Practice	All respondents with a complete survey and a child born since 2018-09-01 (after the end of the intervention)	m5, m6, m7, m8	0·11 (-0·031 to 0·25)	0·13	539
Exclusively breastfed child first 6 months without giving chupón	Practice	All respondents with a complete survey and a child born since 2018-09-01 (after the end of the intervention)	m5, m6, m7, m8	0·20 (0·058 to 0·35)	0·0060	539

For peer review only - http://bmjopen.bmj.com/site/about/guidelines.xhtml

Breastfed immediately after birth	Practice	All respondents with a complete survey and a child born since 2018-09-01 (after the end of the intervention) who reported a natural birth	m5, m6, m7, m8	0·071 (-0·033 to 0·18)	0·18	428
Identifies that newborn should be breastfed immediately after birth	Knowledge and attitudes	All respondents with a complete survey	m5, m6, m7, m8	0·037 (0·0018 to 0·072)	0·040	9196
Identifies that newborns should only be given breast milk during first 6 months	Knowledge and attitudes	All respondents with a complete survey	m5, m6, m7, m8	0·061 (0·030 to 0·092)	0·0001	9196
Believes newborns should not be given chupón during first 6 months	Knowledge and attitudes	All respondents with a complete survey	m5, m6, m7, m8	0·22 (0·16 to 0·28)	<0·0001	9196
**Paternal involvement**						
Father accompanied mother to clinic for prenatal care visit at least once	Practice	All respondents with a complete survey and a child born since 2018-09-01 (after the end of the intervention) who received prenatal care	m5, m6, m7	-0·071 (-0·30 to 0·16)	0·54	519
Father accompanied mother to seek medical care for pregnancy danger sign	Practice	All respondents with a complete survey and a child born since 2018-09-01 (after the end of the intervention) who experienced a prenatal care danger sign and sought care	m5, m6, m7	0·073 (-0·24 to 0·39)	0·65	134
Father waited at birthplace during labor	Practice	All respondents with a complete survey and a child born since 2018-09-01 (after the end of the intervention)	m5, m6, m7	-0·019 (-0·16 to 0·12)	0·79	540
Father accompanied mother to seek medical care for postnatal danger sign	Practice	All respondents with a complete survey and a child born since 2018-09-01 (after the end of the intervention) and sought care for postpartum problem	m5, m6, m7	0·070 (-0·26 to 0·40)	0·68	93
Father sought medical care for newborn for postnatal danger sign	Practice	All respondents with a complete survey and a child born since 2018-09-01 (after the end of the intervention) and sought care for a postnatal problem	m5, m6, m7	0·044 (-0·24 to 0·33)	0·76	98
Father held child	Practice	All respondents with a complete survey and a child born since 2018-09-01 (after the end of the intervention)	m5, m6, m7	-0·010 (-0·11 to 0·091)	0·85	535
Father played with child	Practice	All respondents with a complete survey and a child born since 2018-09-01 (after the end of the intervention)	m5, m6, m7	-0·045 (-0·20 to 0·11)	0·56	535
Father took child to clinic when sick	Practice	All respondents with a complete survey and a child born since 2018-09-01 (after the end of the intervention)	m5, m6, m7	-0·086 (-0·24 to 0·072)	0·29	535
Believes father should accompany mother to prenatal care visits	Knowledge and attitudes	All respondents with a complete survey	m5, m6, m7	0·044 (0·021 to 0·067)	0·0002	9158
Believes father should wait at birth location while mother gives birth	Knowledge and attitudes	All respondents with a complete survey	m5, m6, m7	0·018 (-0·0006 to 0·037)	0·058	9156

For peer review only - http://bmjopen.bmj.com/site/about/guidelines.xhtml

Believes father should care for children when sick	Knowledge and attitudes	All respondents with a complete survey	m5, m6, m7	0·0005 (-0·0077 to 0·0086)	0·91	9177
**Diarrhea management**						
Did not report diarrhea in past 4 weeks	Practice	All respondents with a complete survey	m9	-0·0069 (-0·025 to 0·011)	0·46	9182
Used ORT to treat diarrhea in past 4 weeks	Practice	All respondents with a complete survey who had diarrhea in past 4 weeks	m9	-0·098 (-0·28 to 0·087)	0·30	241
Used appropriate treatment for diarrhea in past 4 weeks[c]	Practice	All respondents with a complete survey who had diarrhea in past 4 weeks	m9	-0·021 (-0·16 to 0·12)	0·78	243
Child did not experience diarrhea past 4 weeks	Practice	All respondents with a complete survey and a new child <= 5 years old reported at wave 3 who had diarrhea in past 4 weeks	m9	-0·023 (-0·070 to 0·024)	0·34	3033
Child experienced diarrhea past 4 weeks, was given appropriate amount of liquids	Practice	All respondents with a complete survey and a new child <= 5 years old reported at wave 3 who had diarrhea in past 4 weeks	m9	-0·15 (-0·35 to 0·050)	0·14	310
Child experienced diarrhea past 4 weeks, was given appropriate amount of food	Practice	All respondents with a complete survey and a new child <= 5 years old reported at wave 3 who had diarrhea in past 4 weeks	m9	-0·12 (-0·26 to 0·015)	0·081	313
Child experienced diarrhea past 4 weeks, was given ORT	Practice	All respondents with a complete survey and a new child <= 5 years old reported at wave 3 who had diarrhea in past 4 weeks	m9	0·15 (-0·041 to 0·35)	0·12	310
Child experienced diarrhea past 4 weeks, was given appropriate treatment	Practice	All respondents with a complete survey and a new child <= 5 years old reported at wave 3 who had diarrhea in past 4 weeks	m9	0·17 (-0·020 to 0·36)	0·080	312
Child experienced diarrhea past 4 weeks, was given zinc 10-14 days	Practice	All respondents with a complete survey and a new child <= 5 years old reported at wave 3 who had diarrhea in past 4 weeks	m9	··	··	··
Identified appropriate diarrhea treatment methods[d]	Knowledge and attitudes	All respondents with a complete survey	m9	0·13 (0·069 to 0·19)	<0·0001	9195
Identified zinc 10-14 days as diarrhea treatment	Knowledge and attitudes	All respondents with a complete survey	m9	0·054 (0·021 to 0·087)	0·0013	9181
Identified zinc supplement as way to prevent diarrhea	Knowledge and attitudes	All respondents with a complete survey	m9	0·071 (0·0038 to 0·14)	0·038	9196
Identified breastfeeding as way to prevent diarrhea	Knowledge and attitudes	All respondents with a complete survey	m9	0·038 (-0·023 to 0·099)	0·23	9196
Correctly answered diarrhea treatment with zinc riddle	Intervention knowledge	All respondents with a complete survey	m9	0·35 (0·30 to 0·40)	<0·0001	9197
**Respiratory illness**						
Did not report respiratory illness (coughing) for 2 weeks	Practice	All respondents with a complete survey	m9	-0·012 (-0·051 to 0·028)	0·56	9188
Child did not have cough past 4 weeks	Practice	All respondents with a complete survey	m9	-0·058 (-0·12 to 0·0070)	0·081	3034

For peer review only - http://bmjopen.bmj.com/site/about/guidelines.xhtml

Child had cough past 4 weeks, care was sought	Practice	All respondents with a complete survey and a new child <= 5 years old reported at wave 3 who had an illness with a cough in past 4 weeks	m9	0·064 (-0·090 to 0·22)	0·41	647
Identified vaccination as way to prevent respiratory illness	Knowledge and attitudes	All respondents with a complete survey	m9	-0·0053 (-0·046 to 0·036)	0·80	9196
Identified breastfeeding as way to prevent respiratory illness	Knowledge and attitudes	All respondents with a complete survey	m9	0·013 (-0·045 to 0·070)	0·66	9196
Identified fever as d.s. for children with respiratory illness	Knowledge and attitudes	All respondents with a complete survey	m9	0·021 (-0·0036 to 0·046)	0·093	9187
Identified difficulty breathing as d.s. for children with respiratory illness	Knowledge and attitudes	All respondents with a complete survey	m9	0·011 (-0·0046 to 0·026)	0·17	9187
Identified rapid breathing as d.s. for children with respiratory illness	Knowledge and attitudes	All respondents with a complete survey	m9	0·0096 (-0·0010 to 0·020)	0·075	9187
**Reproductive health**						
Reported ever using birth control to delay or avoid pregnancy	Practice	All respondents with a complete survey	m4, m10, m12	-0·017 (-0·069 to 0·036)	0·53	9191
Reported currently using birth control	Practice	All respondents with a complete survey who are not pregnant and have ever used birth control	m4, m10, m12	0·033 (-0·035 to 0·10)	0·34	3420
Believes woman should be at least 18 years of age to have her first child	Knowledge and attitudes	All respondents with a complete survey	m4, m10, m12	0·017 (0·0098 to 0·025)	<0·0001	8713
**Gender/reproductive norms**						
Birth location chosen either jointly or by woman	Practice	All respondents with a complete survey and a child born since 2018-09-01 (after the end of the intervention)	m14	0·055 (-0·036 to 0·15)	0·24	540

Results based on sub-sample that completed baseline survey, N=9285 (household targeted n=2919, household not targeted n=6366). Baseline covariates included age, gender, education, marital status, indigenous status, and household wealth index. d.s.=Danger sign, ORT=Oral rehydration therapy. ··Model did not converge. Covariates were dropped sequentially if they predicted outcome perfectly. Standard errors are clustered at the village level. Robust 95% CIs in parentheses.

[a] 'Identified correct ways to provide newborn care' outcome was coded as correct if the respondent identified holding baby skin to skin, getting their health checked by a professional, immediately putting them to the breast, or making sure their diaper is clean and dry, and did not identify giving purgante, wrapping a fajero, giving a chupon, or bathing them in water right away.
[b] 'Identified proper cord care' outcome was coded as correct if respondent identified applying alcohol, applying iodine, keeping cord clean/dry, or applying peroxide, and did not identify applying oil, applying coffee, applying quina water, wrapping a fajero, applying powder, burning, applying ashes, applying a clamp, or applying thiomersal.

For peer review only - http://bmjopen.bmj.com/site/about/guidelines.xhtml

[c] 'Used appropriate treatment for diarrhea in past 4 weeks' outcome was coded as correct if respondent did any of the following: antibiotic, zinc, give extra food, give extra liquid, and did not do any of the following: anti-diarrhea medication, home remedy, laxative, deworming medication, chupon, massage, stop eating foods, stop taking liquids.
[d] 'Identified appropriate diarrhea treatment methods' outcome was coded as correct if respondent identified any of the following: antibiotic, zinc, ORS or Litrosol, give extra food, give extra liquid, get medical attention, and did not identify any of the following: anti-diarrhea medication, laxative, deworming medication, home remedy, chupon, massage, stop giving foods, stop giving liquids, or do nothing.

For peer review only

For peer review only - http://bmjopen.bmj.com/site/about/guidelines.xhtml

Table S9. Absolute changes in primary and secondary outcomes from baseline to endline survey

	Outcome type	Respondent denominator	Baseline			Endline			Change in outcome %, untargeted	Change in outcome %, targeted
			Outcome %, untargeted	Outcome %, targeted	p value	Outcome %, untargeted	Outcome %, targeted	p value		
**Prenatal care**										
Currently taking folic acid tablets (daily in past 7 days)	Practice	All women 15+ with a complete survey	30·7	32·3	0·255	20·3	21·0	0·562	-10·4	-11·3
Made a birth plan in preparation for birth	Practice	All respondents with a complete survey and a child born since 2018-09-01 (after the end of the intervention)	63·7	58·9	0·011	78·2	77·1	0·834	+14·5	+18·2
Saved money in preparation for birth	Practice	All respondents with a complete survey and a child born since 2018-09-01 (after the end of the intervention)	64·5	62·9	0·391	79·7	77·3	0·545	+15·2	+14·4
Sold animal in preparation for birth	Practice	All respondents with a complete survey and a child born since 2018-09-01 (after the end of the intervention)	NA	NA	NA	9·6	9·0	0·937	NA	NA
Sought prenatal care within 12 weeks	Practice	All respondents with a complete survey and a child born since 2018-09-01 (after the end of the intervention)	73·0	73·8	0·666	78·2	75·4	0·502	+5·2	+1·6
Identifies that women should take folic acid before pregnancy	Knowledge and attitudes	All respondents with a complete survey	73·7	73·3	0·728	76·0	82·0	<0·001	+2·3	+8·7
Identifies that women should seek prenatal care first 12 weeks of pregnancy	Knowledge and attitudes	All respondents with a complete survey	86·1	86·5	0·665	85·4	88·0	<0·001	-0·7	+1·5
Identifies accompanying woman to prenatal care visits as method of support during pregnancy	Knowledge and attitudes	All respondents with a complete survey	NA	NA	NA	30·5	33·0	0·013	NA	NA

For peer review only

For peer review only - http://bmjopen.bmj.com/site/about/guidelines.xhtml

Identifies ensuring that woman eats well as method of support during pregnancy	Knowledge and attitudes	All respondents with a complete survey	NA	NA	NA	29·4	26·6	0·004	NA	NA
Identifies ensuring that woman rests 1 hour per day well as method of support during pregnancy	Knowledge and attitudes	All respondents with a complete survey	NA	NA	NA	11·3	11·3	0·998	NA	NA
Identifies avoiding violence as method of support during pregnancy	Knowledge and attitudes	All respondents with a complete survey	NA	NA	NA	7·2	5·8	0·011	NA	NA
Identifies encouraging women to take vitamins as method of support during pregnancy	Knowledge and attitudes	All respondents with a complete survey	NA	NA	NA	12·2	10·5	0·016	NA	NA
Identifies helping woman with house work/child care as method of support during pregnancy	Knowledge and attitudes	All respondents with a complete survey	NA	NA	NA	55·2	56·8	0·124	NA	NA
Identifies saving animals to sell as method of preparing for birth expenses	Knowledge and attitudes	All respondents with a complete survey	NA	NA	NA	3·7	5·2	<0·001	NA	NA
Identifies knowing cost of trip to maternal clinic as method of preparing for birth expenses	Knowledge and attitudes	All respondents with a complete survey	NA	NA	NA	8·0	7·1	0·125	NA	NA
Identifies having a savings plan as method of preparing for birth expenses	Knowledge and attitudes	All respondents with a complete survey	NA	NA	NA	89·4	92·2	<0·001	NA	NA
Correctly answered prenatal care knowledge riddle	Intervention knowledge	All respondents with a complete survey	NA	NA	NA	11·2	16·1	<0·001	NA	NA
**Facility based birth**										
Gave birth in health facility	Practice	All respondents with a complete survey and a child born since 2018-09-01 (after the end of the intervention)	74·8	72·3	0·142	84·4	81·0	0·352	+9·6	+8·7
Believes health facility is best place to give birth	Knowledge and attitudes	All respondents with a complete survey	91·1	90·2	0·19	92·8	95·0	<0·001	+1·7	+4·8
**Pregnancy danger signs**										
Woman sought medical care for pregnancy danger sign	Practice	All respondents with a complete survey and a child born since 2018-	64·7	63·9	0·852	63·2	62·7	1	-1·5	-1·2

For peer review only - http://bmjopen.bmj.com/site/about/guidelines.xhtml

		09-01 (after the end of the intervention) who experienced a prenatal care danger sign								
Identified bleeding as pregnancy d.s.	Knowledge and attitudes	All respondents with a complete survey	41·5	41·4	0·952	27·5	29·5	0·034	-14·0	-11·9
Identified seizure as pregnancy d.s.	Knowledge and attitudes	All respondents with a complete survey	6·3	5·4	0·103	1·5	1·9	0·229	-4·8	-3·5
Identified headache as pregnancy d.s.	Knowledge and attitudes	All respondents with a complete survey	38·1	39·2	0·32	37·0	41·4	<0·001	-1·1	+2·2
Identified ringing in ears as pregnancy d.s.	Knowledge and attitudes	All respondents with a complete survey	3·9	3·8	0·789	1·5	1·5	1	-2·4	-2·3
Identified dizziness as pregnancy d.s.	Knowledge and attitudes	All respondents with a complete survey	36·2	36·9	0·521	37·1	36·6	0·623	+0·9	-0·3
Identified difficulty urinating as pregnancy d.s.	Knowledge and attitudes	All respondents with a complete survey	1·9	2·2	0·555	1·0	1·1	0·668	-0·9	-1·1
Identified reduced or absent fetal movement as pregnancy d.s.	Knowledge and attitudes	All respondents with a complete survey	5·9	6·1	0·662	4·6	4·5	0·906	-1·3	-1·6
Identified water breaking as pregnancy d.s.	Knowledge and attitudes	All respondents with a complete survey	2·1	2·4	0·415	1·2	1·1	0·779	-0·9	-1·3
Identified fever as pregnancy d.s.	Knowledge and attitudes	All respondents with a complete survey	13·7	13·6	0·907	17·1	19·6	0·002	+3·4	+6·0
Identified swelling of face/hands/feet as pregnancy d.s.	Knowledge and attitudes	All respondents with a complete survey	9·3	9·7	0·489	14·0	16·5	0·001	+4·7	+6·8
**Postnatal care for mother**										
Mother had health checked by professional within 3 days of birth	Practice	All respondents with a complete survey and a child born since 2018-09-01 (after the end of the intervention)	14·6	14·1	0·744	22·9	27·4	0·263	+8·3	+13·3
Mother had health checked by professional within 7 days of birth	Practice	All respondents with a complete survey and a child born since 2018-09-01 (after the end of the intervention)	27·2	27·2	1	43·1	43·8	0·942	+15·9	+16·6

For peer review only - http://bmjopen.bmj.com/site/about/guidelines.xhtml

Mother sought medical care for postnatal danger sign	Practice	All respondents with a complete survey and a child born since 2018-09-01 (after the end of the intervention)	51·2	46·6	0·214	47·5	51·3	0·706	-3·7	+4·7
Identifies that mother should receive postnatal medical check-up within 3 days of birth	Knowledge and attitudes	All respondents with a complete survey	9·1	10·0	0·17	16·0	24·4	<0·001	+6·9	+14·4
Identifies that mother should receive postnatal medical check-up within 7 days of birth	Knowledge and attitudes	All respondents with a complete survey	23·9	24·5	0·542	33·8	40·8	<0·001	+9·9	+16·3
Identified heavy vaginal bleeding as postnatal d.s.	Knowledge and attitudes	All respondents with a complete survey	42·4	43·6	0·312	37·6	41·4	<0·001	-4·8	-2·2
Identified fever as postnatal d.s.	Knowledge and attitudes	All respondents with a complete survey	37·4	36·2	0·283	26·1	28·5	0·013	-11·3	-7·7
Identified vaginal discharge as postnatal d.s.	Knowledge and attitudes	All respondents with a complete survey	6·8	6·8	1	3·6	3·7	0·695	-3·2	-3·1
Identified headache as postnatal d.s.	Knowledge and attitudes	All respondents with a complete survey	22·1	23·5	0·141	17·0	19·5	0·002	-5·1	-4·0
Identified convulsions or fits as postnatal d.s.	Knowledge and attitudes	All respondents with a complete survey	1·0	1·1	0·944	0·7	0·8	0·591	-0·3	-0·3
**Postnatal care for newborn**										
Newborn had health checked by professional within 3 days of birth	Practice	All respondents with a complete survey and a child born since 2018-09-01 (after the end of the intervention)	16·6	15·9	0·65	22·4	29·8	0·056	+5·8	+13·9
Newborn had health checked by professional within 7 days of birth	Practice	All respondents with a complete survey and a child born since 2018-09-01 (after the end of the intervention)	29·7	28·4	0·456	43·0	48·1	0·271	+13·3	+19·7
Newborn experienced health problem in first month, care was sought	Practice	All respondents with a complete survey and a child born since 2018-09-01 (after the end of the intervention)	74·5	70·0	0·175	65·5	55·2	0·242	-9·0	-14·8

For peer review only - http://bmjopen.bmj.com/site/about/guidelines.xhtml

Identified correct ways to provide newborn care[a]	Knowledge and attitudes	All respondents with a complete survey	4·6	6·1	0·003	2·1	3·5	<0·001	-2·5	-2·6
Identified fever as newborn d.s.	Knowledge and attitudes	All respondents with a complete survey	68·2	66·8	0·201	57·7	56·8	0·379	-10·5	-10·0
Identified diarrhea as newborn d.s.	Knowledge and attitudes	All respondents with a complete survey	48·7	49·2	0·694	32·5	35·3	0·005	-16·2	-13·9
Identified difficulty breathing as newborn d.s.	Knowledge and attitudes	All respondents with a complete survey	35·9	36·3	0·721	9·8	11·0	0·062	-26·1	-25·3
Identified vomiting as newborn d.s.	Knowledge and attitudes	All respondents with a complete survey	27·6	28·2	0·588	11·5	11·8	0·7	-16·1	-16·4
Identified poor appetite as newborn d.s.	Knowledge and attitudes	All respondents with a complete survey	5·5	6·8	0·018	2·2	2·0	0·509	-3·3	-4·8
Identified redness/bleeding around cord as newborn d.s.	Knowledge and attitudes	All respondents with a complete survey	4·9	5·0	0·823	4·4	5·9	0·001	-0·5	+0·9
Identified pus in cord as newborn d.s.	Knowledge and attitudes	All respondents with a complete survey	4·5	5·6	0·02	2·9	4·5	<0·001	-1·6	-1·1
Identified cold skin as newborn d.s.	Knowledge and attitudes	All respondents with a complete survey	1·2	1·7	0·036	0·8	0·6	0·283	-0·4	-1·1
Identified cough as newborn d.s.	Knowledge and attitudes	All respondents with a complete survey	1·6	0·9	0·009	37·5	35·7	0·084	+35·9	+34·8
Identified cold as newborn d.s.	Knowledge and attitudes	All respondents with a complete survey	1·3	1·1	0·679	36·2	32·5	<0·001	+34·9	+31·4
Identified problems latching as newborn d.s.	Knowledge and attitudes	All respondents with a complete survey	0·0	0·0	0·844	2·6	2·7	1	+2·6	+2·7
Identified pneumonia as newborn d.s.	Knowledge and attitudes	All respondents with a complete survey	NA	NA	NA	14·0	14·4	0·62	NA	NA
Did not use harmful substances to treat cord stump	Practice	All respondents with a complete survey and a child born since 2018-09-01 (after the end of the intervention)	84·4	81·7	0·066	44·1	53·2	0·042	-40·3	-28·5
Did not wrap fajero around newborn in first 7 days after birth	Practice	All respondents with a complete survey and a child born since 2018-09-01 (after the end of the intervention)	6·1	5·2	0·376	22·2	43·1	<0·001	+16·1	+37·9
Identified proper cord care methods[b]	Knowledge and attitudes	All respondents with a complete survey	46·0	46·4	0·731	38·7	49·0	<0·001	-7·3	+2·6

For peer review only - http://bmjopen.bmj.com/site/about/guidelines.xhtml

Correctly answered proper cord care riddle	Intervention knowledge	All respondents with a complete survey	NA	NA	NA	21·5	39·4	<0·001	NA	NA
Kept newborn wrapped first 7 days after birth	Practice	All respondents with a complete survey and a child born since 2018-09-01 (after the end of the intervention)	98·7	98·0	0·182	97·5	92·9	0·01	-1·2	-5·1
Held newborn skin-to-skin during first month after birth	Practice	All respondents with a complete survey and a child born since 2018-09-01 (after the end of the intervention)	100·0	100·0	NA	31·0	40·5	0·023	-69·0	-59·5
**Breastfeeding**										
Exclusively breastfed child first 6 months	Practice	All respondents with a complete survey and a child born since 2018-09-01 (after the end of the intervention)	57·8	57·9	0·99	56·4	65·9	0·028	-1·4	+8·0
Exclusively breastfed child first 6 months without giving chupón	Practice	All respondents with a complete survey and a child born since 2018-09-01 (after the end of the intervention)	35·1	31·4	0·042	41·7	52·1	0·017	+6·6	+20·7
Breastfed immediately after birth	Practice	All respondents with a complete survey and a child born since 2018-09-01 (after the end of the intervention) who reported a natural birth	86·5	83·8	0·071	82·4	88·9	0·073	-4·1	+5·1
Identifies that newborn should be breastfed immediately after birth	Knowledge and attitudes	All respondents with a complete survey	81·0	81·7	0·469	80·5	82·0	0·096	-0·5	+0·3
Identifies that newborns should only be given breast milk during first 6 months	Knowledge and attitudes	All respondents with a complete survey	80·5	81·2	0·495	81·9	85·4	<0·001	+1·4	+4·2
Believes newborns should not be given chupón during first 6 months	Knowledge and attitudes	All respondents with a complete survey	24·3	22·4	0·048	26·7	44·9	<0·001	+2·4	+22·5

For peer review only - http://bmjopen.bmj.com/site/about/guidelines.xhtml

Paternal involvement										
Father accompanied mother to clinic for prenatal care visit at least once	Practice	All respondents with a complete survey and a child born since 2018-09-01 (after the end of the intervention) who received prenatal care	52·5	52·8	0·906	58·8	55·9	0·565	+6·3	+3·1
Father accompanied mother to seek medical care for pregnancy danger sign	Practice	All respondents with a complete survey and a child born since 2018-09-01 (after the end of the intervention) who experienced a prenatal care danger sign and sought care	69·7	60·9	0·019	67·7	71·7	0·749	-2·0	+10·8
Father waited at birthplace during labor	Practice	All respondents with a complete survey and a child born since 2018-09-01 (after the end of the intervention)	76·6	76·7	0·987	80·7	73·5	0·05	+4·1	-3·2
Father accompanied mother to seek medical care for postnatal danger sign	Practice	All respondents with a complete survey and a child born since 2018-09-01 (after the end of the intervention) and sought care for postpartum problem	68·8	69·7	0·923	66·1	76·3	0·398	-2·7	+6·6
Father sought medical care for newborn for postnatal danger sign	Practice	All respondents with a complete survey and a child born since 2018-09-01 (after the end of the intervention) and sought care for a postnatal problem	76·2	72·5	0·352	67·5	66·7	1	-8·7	-5·8
Father held child	Practice	All respondents with a complete survey and a child born since 2018-09-01 (after the end of the intervention)	97·3	96·9	0·61	90·1	88·6	0·654	-7·2	-8·3

For peer review only - http://bmjopen.bmj.com/site/about/guidelines.xhtml

Father played with child	Practice	All respondents with a complete survey and a child born since 2018-09-01 (after the end of the intervention)	90·6	91·0	0·793	84·7	79·0	0·103	-5·9	-12·0
Father took child to clinic when sick	Practice	All respondents with a complete survey and a child born since 2018-09-01 (after the end of the intervention)	73·2	69·6	0·049	63·9	62·9	0·875	-9·3	-6·7
Believes father should accompany mother to prenatal care visits	Knowledge and attitudes	All respondents with a complete survey	92·3	92·0	0·569	92·4	96·0	<0·001	+0·1	+4·0
Believes father should wait at birth location while mother gives birth	Knowledge and attitudes	All respondents with a complete survey	93·9	94·2	0·522	94·3	94·8	0·387	+0·4	+0·6
Believes father should care for children when sick	Knowledge and attitudes	All respondents with a complete survey	99·3	99·2	0·91	99·1	99·0	0·687	-0·2	-0·2
**Diarrhea management**										
Did not report diarrhea in past 4 weeks	Practice	All respondents with a complete survey	97·6	97·4	0·516	97·5	97·2	0·355	-0·1	-0·2
Used ORT to treat diarrhea in past 4 weeks	Practice	All respondents with a complete survey who had diarrhea in past 4 weeks	61·6	42·9	0·011	41·5	37·8	0·653	-20·1	-5·1
Used appropriate treatment for diarrhea in past 4 weeks[c]	Practice	All respondents with a complete survey who had diarrhea in past 4 weeks	25·7	26·0	1	19·9	19·6	1	-5·8	-6·4
Child did not experience diarrhea past 4 weeks	Practice	All respondents with a complete survey and a new child <= 5 years old reported at wave 3 who had diarrhea in past 4 weeks	84·2	82·3	0·189	89·5	89·5	1	+5·3	+7·2
Child experienced diarrhea past 4 weeks, was given appropriate amount of liquids	Practice	All respondents with a complete survey and a new child <= 5 years old reported at wave 3 who had diarrhea in past 4 weeks	52·4	54·7	0·685	51·1	41·6	0·123	-1·3	-13·1

For peer review only - http://bmjopen.bmj.com/site/about/guidelines.xhtml

Child experienced diarrhea past 4 weeks, was given appropriate amount of food	Practice	All respondents with a complete survey and a new child <= 5 years old reported at wave 3 who had diarrhea in past 4 weeks	36·8	36·1	0·958	30·0	21·2	0·113	-6·8	-14·9
Child experienced diarrhea past 4 weeks, was given ORT	Practice	All respondents with a complete survey and a new child <= 5 years old reported at wave 3 who had diarrhea in past 4 weeks	66·3	58·0	0·079	49·2	55·0	0·372	-17·1	-3·0
Child experienced diarrhea past 4 weeks, was given appropriate treatment	Practice	All respondents with a complete survey and a new child <= 5 years old reported at wave 3 who had diarrhea in past 4 weeks	27·3	29·7	0·614	20·3	28·6	0·117	-7·0	-1·1
Child experienced diarrhea past 4 weeks, was given zinc 10-14 days	Practice	All respondents with a complete survey and a new child <= 5 years old reported at wave 3 who had diarrhea in past 4 weeks	1·6	0·6	0·581	1·4	2·0	1	-0·2	+1·4
Identified appropriate diarrhea treatment methods[d]	Knowledge and attitudes	All respondents with a complete survey	41·3	41·2	0·963	35·4	45·0	<0·001	-5·9	+3·8
Identified zinc 10-14 days as diarrhea treatment	Knowledge and attitudes	All respondents with a complete survey	4·1	4·2	0·727	7·3	8·3	0·084	+3·2	+4·1
Identified zinc supplement as way to prevent diarrhea	Knowledge and attitudes	All respondents with a complete survey	45·4	45·5	0·994	67·2	73·5	<0·001	+21·8	+28·0
Identified breastfeeding as way to prevent diarrhea	Knowledge and attitudes	All respondents with a complete survey	26·2	26·0	0·837	65·3	67·5	0·028	+39·1	+41·5
Correctly answered diarrhea treatment with zinc riddle	Intervention knowledge	All respondents with a complete survey	NA	NA	NA	18·9	41·0	<0·001	NA	NA
**Respiratory illness**										
Did not report respiratory illness (coughing) for 2 weeks	Practice	All respondents with a complete survey	79·2	79·6	0·633	85·5	83·5	0·011	+6·3	+3·9

For peer review only - http://bmjopen.bmj.com/site/about/guidelines.xhtml

Child did not have cough past 4 weeks	Practice	All respondents with a complete survey	68·0	63·2	0·008	79·2	78·6	0·741	+11·2	+15·4
Child had cough past 4 weeks, care was sought	Practice	All respondents with a complete survey and a new child <= 5 years old reported at wave 3 who had an illness with a cough in past 4 weeks	4·1	2·9	0·447	34·3	33·5	0·905	+30·2	+30·6
Identified vaccination as way to prevent respiratory illness	Knowledge and attitudes	All respondents with a complete survey	50·7	50·8	0·939	83·9	84·8	0·236	+33·2	+34·0
Identified breastfeeding as way to prevent respiratory illness	Knowledge and attitudes	All respondents with a complete survey	34·0	33·6	0·728	70·9	72·0	0·262	+36·9	+38·4
Identified fever as d.s. for children with respiratory illness	Knowledge and attitudes	All respondents with a complete survey	81·6	80·8	0·392	94·1	95·3	0·022	+12·5	+14·5
Identified difficulty breathing as d.s. for children with respiratory illness	Knowledge and attitudes	All respondents with a complete survey	88·6	88·3	0·691	96·7	97·6	0·018	+8·1	+9·3
Identified rapid breathing as d.s. for children with respiratory illness	Knowledge and attitudes	All respondents with a complete survey	85·0	85·2	0·853	97·5	98·1	0·079	+12·5	+12·9
**Reproductive health**										
Reported ever using birth control to delay or avoid pregnancy	Practice	All respondents with a complete survey	33·9	31·7	0·056	38·2	40·0	0·082	+4·3	+8·3
Reported currently using birth control	Practice	All respondents with a complete survey who are not pregnant and have ever used birth control	56·0	57·2	0·584	54·7	59·4	0·007	-1·3	+2·2
Believes woman should be at least 18 years of age to have her first child	Knowledge and attitudes	All respondents with a complete survey	98·4	97·9	0·112	98·3	99·0	0·02	-0·1	+1·1
**Gender/reproductive norms**										
Birth location chosen either jointly or by woman	Practice	All respondents with a complete survey and a child born since 2018-09-01 (after the end of the intervention)	92·4	91·8	0·593	84·6	85·8	0·785	-7·8	-6·0

For peer review only - http://bmjopen.bmj.com/site/about/guidelines.xhtml

Results based on final sample of N=10263 (household targeted n=3238, household not targeted n=7025). d.s.=Danger sign, NA=Not applicable, ORT=Oral rehydration therapy. Proportions with NA could not be assessed because the outcome was not part of the baseline instrument. Proportion comparisons against a null hypothesis that there is no difference in outcomes between those targeted and untargeted were performed using a two-sided t-test.

[a] 'Identified correct ways to provide newborn care' outcome was coded as correct if the respondent identified holding baby skin to skin, getting their health checked by a professional, immediately putting them to the breast, or making sure their diaper is clean and dry, and did not identify giving purgante, wrapping a fajero, giving a chupon, or bathing them in water right away.
[b] 'Identified proper cord care' outcome was coded as correct if respondent identified applying alcohol, applying iodine, keeping cord clean/dry, or applying peroxide, and did not identify applying oil, applying coffee, applying quina water, wrapping a fajero, applying powder, burning, applying ashes, applying a clamp, or applying thiomersal.
[c] 'Used appropriate treatment for diarrhea in past 4 weeks' outcome was coded as correct if respondent did any of the following: antibiotic, zinc, give extra food, give extra liquid, and did not do any of the following: anti-diarrhea medication, home remedy, laxative, deworming medication, chupon, massage, stop eating foods, stop taking liquids.
[d] 'Identified appropriate diarrhea treatment methods' outcome was coded as correct if respondent identified any of the following: antibiotic, zinc, ORS or Litrosol, give extra food, give extra liquid, get medical attention, and did not identify any of the following: anti-diarrhea medication, laxative, deworming medication, home remedy, chupon, massage, stop giving foods, stop giving liquids, or do nothing.

For peer review only - http://bmjopen.bmj.com/site/about/guidelines.xhtml

Table S10. Analysis of differences in attrition between baseline and endline surveys

	Lost to follow-up (n=6038)	Completed endline survey (n=10263)	Test statistic	p value
Target status (households), *n (%)*				
Not targeted	3971 (66%)	7025 (68%)	0·87	0·351
Targeted	2067 (34%)	3238 (32%)		
Age, *mean (SD)*	28·7 (15·9)	35·0 (17·5)	-21·49	<0·001
Sex, *n (%)*				
Female	2382 (39%)	6318 (62%)	746·79	<0·001
Male	3656 (61%)	3945 (38%)		
Education, *n (%)*				
Less than primary	2625 (57%)	5964 (64%)	67·02	<0·001
Primary or greater	1985 (43%)	3339 (36%)		
Marital status, *n (%)*				
Single	2719 (45%)	3186 (31%)	335·74	<0·001
Married or Civil Union	3056 (51%)	6348 (62%)		
Separated/Divorced/Widowed	263 (4%)	729 (7%)		
Indigenous status, *n (%)*				
No	4168 (90%)	8061 (87%)	40·36	<0·001
Yes, Maya Chorti	438 (10%)	1229 (13%)		
Yes, other indigenous group	3 (<1%)	6 (<1%)		
Self-rated physical health, *mean (SD)*	3·1 (1·1)	3·3 (1·0)	-8·99	<0·001
Self-rated mental health, *mean (SD)*	3·0 (1·1)	3·2 (1·1)	-8·70	<0·001
Household wealth index, *n (%)*				
Quintile 1	940 (17%)	1674 (17%)	4·10	0·393
Quintile 2	1094 (18%)	1931 (19%)		
Quintile 3	1191 (20%)	1980 (19%)		
Quintile 4	1264 (21%)	2194 (22%)		
Quintile 5	1426 (24%)	2336 (23%)		

Analysis based on subgroup of households/respondents who were randomized in the trial (intention to treat analysis, n=16301). Pearson's chi-squared test was used to assess differences in target status, sex, education, marital status, indigenous status, and household wealth index. Welch two-sample t-test was used to assess age, self-rated physical health, and self-rated mental health.

For peer review only

For peer review only - http://bmjopen.bmj.com/site/about/guidelines.xhtml

References

1. DeSalvo KB, Bloser N, Reynolds K, et al. Mortality prediction with a single general self-rated health question: A meta-analysis. Journal of General Internal Medicine 2006;21:267–75. doi: 10.1111/j.1525-1497.2005.00291.x

2. Kroenke K, Spitzer RL, Williams JB. The Patient Health Questionnaire-2: Validity of a Two-Item Depression Screener. Medical Care 2003;41:1284–92. doi: 10.1097/01.MLR.0000093487.78664.3C

3. Bush K, Kivlahan DR, McDonell MB, et al. The AUDIT alcohol consumption questions (AUDIT-C): an effective brief screening test for problem drinking. Arch Intern Med 1998;158(16):1789–95. doi: 10.1001/archinte.158.16.1789.

4. Pérez-Escamilla R, Dessalines M, Finnigan M, et al. Household food insecurity is associated with childhood malaria in rural Haiti. Journal of Nutrition 2009;139(11):2132–8. doi: 10.3945/jn.109.108852

5. Baird S, Bohren JA, McIntosh C, *et al*. Optimal Design of Experiments in the Presence of Interference. The Review of Economics and Statistics 2018;100:844–60. doi: 10.1162/rest_a_00716

6. Hudgens MG, Halloran ME. Toward Causal Inference With Interference. Journal of the American Statistical Association 2008;103:832–42. doi: 10.1198/016214508000000292

For peer review only - http://bmjopen.bmj.com/site/about/guidelines.xhtml

CONSORT 2010 checklist of information to include when reporting a randomised trial*

Section/Topic	Item No	Checklist item	Reported on page No
**Title and abstract**			
	1a	Identification as a randomised trial in the title	1
	1b	Structured summary of trial design, methods, results, and conclusions (for specific guidance see CONSORT for abstracts)	2
**Introduction**			
Background and objectives	2a	Scientific background and explanation of rationale	4-6
	2b	Specific objectives or hypotheses	6
**Methods**			
Trial design	3a	Description of trial design (such as parallel, factorial) including allocation ratio	7-11
	3b	Important changes to methods after trial commencement (such as eligibility criteria), with reasons	12
Participants	4a	Eligibility criteria for participants	7-8
	4b	Settings and locations where the data were collected	7-8
Interventions	5	The interventions for each group with sufficient details to allow replication, including how and when they were actually administered	9-12 & supplement
Outcomes	6a	Completely defined pre-specified primary and secondary outcome measures, including how and when they were assessed	9-12 & supplement
	6b	Any changes to trial outcomes after the trial commenced, with reasons	NA
Sample size	7a	How sample size was determined	7 & supplement
	7b	When applicable, explanation of any interim analyses and stopping guidelines	NA
Randomisation:			
Sequence generation	8a	Method used to generate the random allocation sequence	8-9 & supplement
	8b	Type of randomisation; details of any restriction (such as blocking and block size)	8-9 & supplement
Allocation concealment mechanism	9	Mechanism used to implement the random allocation sequence (such as sequentially numbered containers), describing any steps taken to conceal the sequence until interventions were assigned	8-9 & supplement

For peer review only - http://bmjopen.bmj.com/site/about/guidelines.xhtml

Implementation	10	Who generated the random allocation sequence, who enrolled participants, and who assigned participants to interventions	8-9 & supplement
Blinding	11a	If done, who was blinded after assignment to interventions (for example, participants, care providers, those assessing outcomes) and how	NA
	11b	If relevant, description of the similarity of interventions	NA
Statistical methods	12a	Statistical methods used to compare groups for primary and secondary outcomes	13-14 & supplement
	12b	Methods for additional analyses, such as subgroup analyses and adjusted analyses	13-14 & supplement
**Results**			
Participant flow (a diagram is strongly recommended)	13a	For each group, the numbers of participants who were randomly assigned, received intended treatment, and were analysed for the primary outcome	14-22 & supplement
	13b	For each group, losses and exclusions after randomisation, together with reasons	15, Figure 1 & supplement
Recruitment	14a	Dates defining the periods of recruitment and follow-up	9
	14b	Why the trial ended or was stopped	NA
Baseline data	15	A table showing baseline demographic and clinical characteristics for each group	Table 1
Numbers analysed	16	For each group, number of participants (denominator) included in each analysis and whether the analysis was by original assigned groups	Supplemental Table S1
Outcomes and estimation	17a	For each primary and secondary outcome, results for each group, and the estimated effect size and its precision (such as 95% confidence interval)	Supplemental Table S1
	17b	For binary outcomes, presentation of both absolute and relative effect sizes is recommended	Supplemental Table S1 & S9
Ancillary analyses	18	Results of any other analyses performed, including subgroup analyses and adjusted analyses, distinguishing pre-specified from exploratory	Figure 2, & supplement
Harms	19	All important harms or unintended effects in each group (for specific guidance see CONSORT for harms)	NA
**Discussion**			
Limitations	20	Trial limitations, addressing sources of potential bias, imprecision, and, if relevant, multiplicity of analyses	25-26, 28
Generalisability	21	Generalisability (external validity, applicability) of the trial findings	25-26, 28
Interpretation	22	Interpretation consistent with results, balancing benefits and harms, and considering other relevant evidence	25-29
**Other information**			

For peer review only - http://bmjopen.bmj.com/site/about/guidelines.xhtml

Registration	23	Registration number and name of trial registry	2,14
Protocol	24	Where the full trial protocol can be accessed, if available	14,32
Funding	25	Sources of funding and other support (such as supply of drugs), role of funders	32

*We strongly recommend reading this statement in conjunction with the CONSORT 2010 Explanation and Elaboration for important clarifications on all the items. If relevant, we also recommend reading CONSORT extensions for cluster randomised trials, non-inferiority and equivalence trials, non-pharmacological treatments, herbal interventions, and pragmatic trials. Additional extensions are forthcoming: for those and for up to date references relevant to this checklist, see www.consort-statement.org.

For peer review only

For peer review only - http://bmjopen.bmj.com/site/about/guidelines.xhtml
